# Influence of 1,2,4-Tri-*tert*-butylcyclopentadienyl Ligand on the Reactivity of the Thorium Bipyridyl Metallocene [η^5^-1,2,4-(Me_3_C)_3_C_5_H_2_]_2_Th(bipy)]

**DOI:** 10.1021/acs.inorgchem.4c02782

**Published:** 2024-10-03

**Authors:** Dongwei Wang, Yi Heng, Tongyu Li, Wanjian Ding, Guohua Hou, Guofu Zi, Marc D. Walter

**Affiliations:** † Department of Chemistry, Beijing Normal University, Beijing 100875, China; ‡ Technische Universität Braunschweig, Institut für Anorganische und Analytische Chemie, Hagenring 30, Braunschweig 38106, Germany

## Abstract

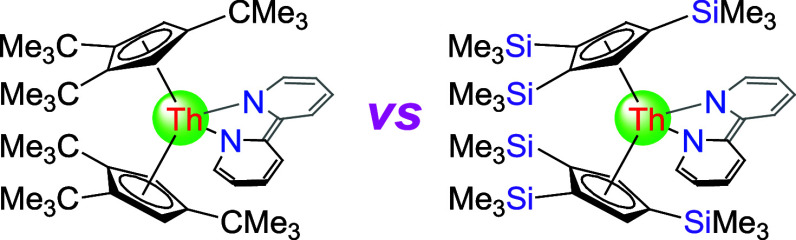

The thorium bipyridyl metallocene (Cp^3*t*Bu^)_2_Th(bipy) (**1**; Cp^3*t*Bu^ = η^5^-1,2,4-(Me_3_C)_3_C_5_H_2_) shows a rich reactivity toward a series of small molecules. For example, complex **1** may act as a synthon for the (Cp^3*t*Bu^)_2_Th(II) fragment as illustrated by its reactivity toward to CuI, hydrazine derivative (PhNH)_2_, Ph_2_*E*_2_ (*E* = S, Se), elemental sulfur (S_8_) and selenium (Se), organic azides, CS_2_, and isothiocyanates. Moreover, in the presence of polar multiple bonds, such as those in ketones Ph_2_CO and (CH_2_)_5_CO, aldehydes *p*-MePhCHO and *p*-ClPhCHO, seleno-ketone (*p*-MeOPh)_2_CSe, nitriles PhCN, Ph_2_CHCN, C_6_H_11_CN, and *p*-(NC)_2_Ph, and benzoyl cyanide PhCOCN, C–C coupling occurs to furnish (Cp^3*t*Bu^)_2_Th[(bipy)(Ph_2_CO)] (**10**), (Cp^3*t*Bu^)_2_Th[(bipy)((CH_2_)_5_CO)] (**11**), (Cp^3*t*Bu^)_2_Th[(bipy)(*p*-MePhCHO)] (**12**), (Cp^3*t*Bu^)_2_Th[(bipy)(*p*-ClPhCHO)] (**13**), (Cp^3*t*Bu^)_2_Th[(bipy){(*p*-MeOPh)_2_CSe}] (**14**), (Cp^3*t*Bu^)_2_Th[(bipy)(PhCN)] (**16**), (Cp^3*t*Bu^)_2_Th[(bipy)(Ph_2_CHCN)] (**17**), (Cp^3*t*Bu^)_2_Th[(bipy)(C_6_H_11_CN)] (**18**), [(Cp^3*t*Bu^)_2_Th]_2_{μ-(bipy)[*p*-Ph(CN)_2_](bipy)} (**20**), and (Cp^3*t*Bu^)_2_Th{(bipy)[PhC(CN)O]} (**21**), respectively. Nevertheless, ketazine (PhCH=N)_2_ or benzyl nitrile PhCH_2_CN forms the dimeric complexes [(Cp^3*t*Bu^)Th]_2_[μ-NC(Ph)(bipy)]_2_ (**15**) and (Cp^3*t*Bu^)_2_Th[(bipy){C(=CHPh)NH}] (**19**), respectively. In contrast, C–N bond cleavage and C–C coupling processes occur upon addition of isonitriles Me_3_CNC and C_6_H_11_NC to **1** to yield the thorium isocyanido amido complexes (Cp^3*t*Bu^)_2_Th[4-(Me_3_C)bipy](NC) (**22**) and (Cp^3*t*Bu^)_2_Th[4-(C_6_H_11_)bipy](NC) (**23**), respectively. Furthermore, a single-electron transfer (SET) process ensues when 1 equiv of CuI is added to **1** to yield the Th(VI) bipyridyl iodide complex (Cp^3*t*Bu^)_2_Th(I)(bipy) (**3**).

## Abstract 2

The thoriumbipyridyl metallocene[η^5^-1,2,4-(Me_3_C)_3_C_5_H_2_]_2_Th(bipy)(**1**) shows a rich reactivity towards a series of small molecules. Moreover, **1** shows a distinctively different reactivity fromits uranium analogue [η^5^-1,2,4-(Me_3_C)_3_C_5_H_2_]_2_U(bipy),which can be rationalized based on the different electronic structure withinthe [η^5^-1,2,4-(Me_3_C)_3_C_5_H_2_]_2_An and [bipy] fragments, as well as steric considerations. Furthermore, steric and electronic effectsmediated by the coordinated cyclopentadienyl ligands significantlyinfluence the reactivity of the thorium bipyridyl metallocenes.

## Introduction

Harnessing the ability of redox-active ligands to accomplish (multi)electron transfer processes have been successfully employed throughout the periodic table for small molecule activation and catalysis.^1−7^ This ability is especially appealing when the metal atom itself cannot readily engage in multielectron process, and in these cases the electrons required for the chemical transformation can either originate jointly from the ligand and the metal atom or exclusively from the ligand.^1−7^ Hence these complexes might also act as synthetic equivalents for low-valent metal complexes.^1−7^ The latter aspect also interested actinide chemists, since low-valent actinide compounds in general are capable to activate a wide range of small molecules.^8−35^ Although several trivalent actinide complexes are known, the number of well-characterized divalent representatives is limited to few selected examples.^36−55^ This can be attributed to very unfavorable redox-potential rendering, which requires well-adapted ligand scaffolds to tame their reactivity. In contrast *in situ* reduction of An(IV) derivatives is a viable route to access highly reactive low-valent intermediates, giving rise to poorly controlled reactivity.^56−62^ To overcome these limitations, actinide complexes incorporating redox-active ligands have been designed^63−78^ that contain for example 2,2′-bipyridine,^79−105^ 1,4-diazabutadiene,^106−111^ pyridine diimine,^112−117^ or arene ligands.^118−137^ We entered this field nearly two decades ago and since then prepared a series of well-behaved bipy actinide metallocenes, (Cp^3tms^)_2_An(bipy) (An = Th (**1′**), U; Cp^3tms^ = η^5^-1,2,4-(Me_3_Si)_3_C_5_H_2_), (Cp^3*t*Bu^)_2_An(bipy) (An = Th (**1**), U (**1″**)), Cp*_2_An(bipy) (An = Th, U; Cp* = η^5^-C_5_Me_5_), (Cp^2tms^)_2_U(bipy) (Cp^2tms^ = η^5^-1,3-(Me_3_Si)_2_C_5_H_3_), and (Cp^2*t*Bu^)_2_An(bipy) (An = Th, U; Cp^2*t*Bu^ = η^5^-1,3-(Me_3_C)_2_C_5_H_3_).^138−149^ This allowed us to investigate the reactivity of the transient Cp_2_An(II) fragments toward various small molecules.^138−149^ For each metal these complexes exhibit related reactivity patterns, but individual differences arise due to the steric and electronic influence exerted by the cyclopentadienyl ligand. This can be illustrated by comparing the reactivity of Ph_2_C*E* (*E* = O, S) toward for two uranium derivatives (Cp^3*t*Bu^)_2_U(bipy) (**1″**; Table 1)^147^ and (Cp^3tms^)_2_U(bipy),^145^ that only differ in the substituents on the cyclopentadienyl, i.e., *tert*-butyl vs trimethylsilyl. Whereas, in the former case, the bipyridyl ligand is displaced,^147^ while an insertion reaction occurs in the latter one.^145^ This led to the question whether similar reactivity differences might also emerge between the related thorium complexes (Cp^3*t*Bu^)_2_Th(bipy) (**1**)^139^ and (Cp^3tms^)_2_Th(bipy) (**1′**).^148^ The reactivity of **1′** was previously probed in great detail (Table 1), and we decided to perform a similar study on its counterpart **1**. This also allows us to contextualize and to compare this reactivity to those of Cp*_2_Th(bipy), (Cp^2*t*Bu^)_2_Th(bipy), and (Cp^3tms^)_2_Th(bipy) (**1′**) (Table 1).^140,142,148,149^ For completeness, we also discuss similarities and differences between the reactivity of the thorium bipy complex **1** and its uranium counterpart (Cp^3*t*Bu^)_2_U(bipy) (**1″**; Table 1)^147^ in this contribution.

**Table 1:**
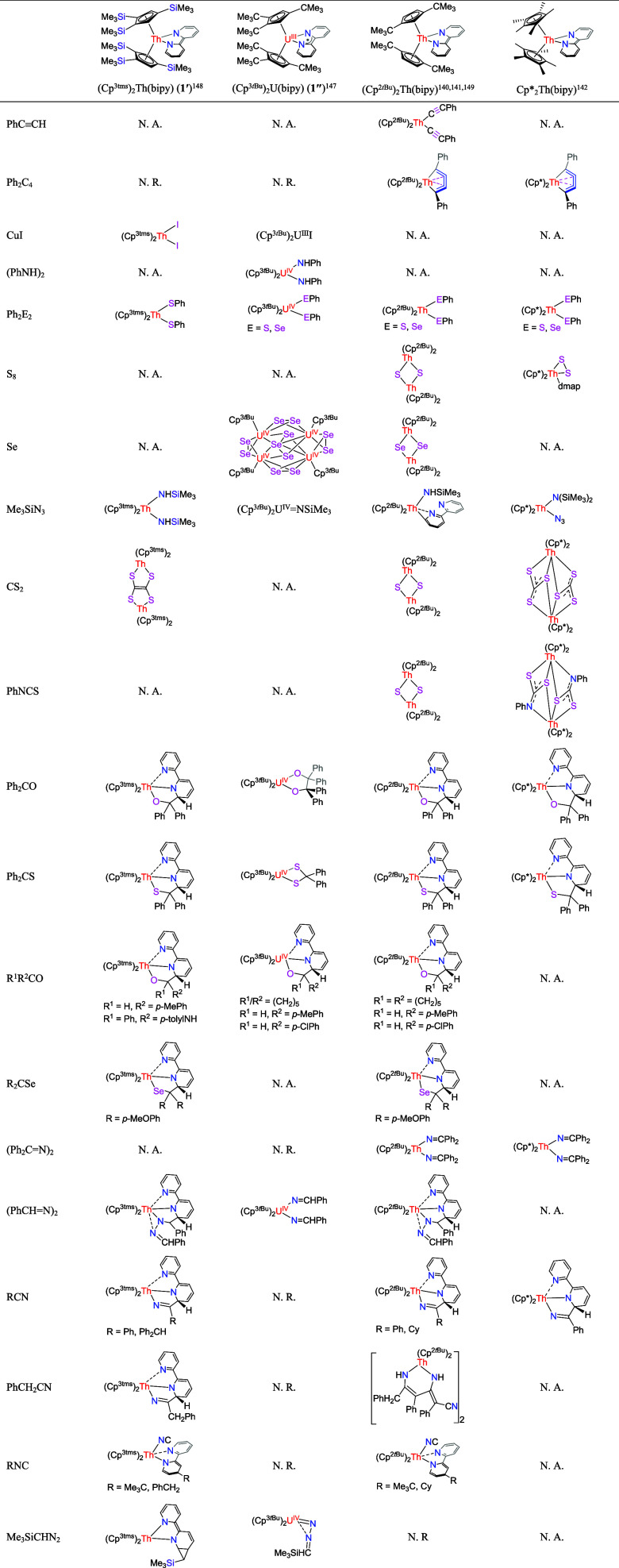
Selected Examples Concerning the Reactivity of Cp_2_An(bipy)^a^

a Cp^3tms^ = η^5^-1,2,4-(Me_3_Si)_3_C_5_H_2_. Cp* = η^5^-C_5_Me_5_. Cp^3*t*Bu^ = η^5^-1,2,4-(Me_3_C)_3_C_5_H_2_. Cp^2*t*Bu^ = η^5^-1,3-(Me_3_C)_2_C_5_H_3_. N. R.= no reaction. N. A. = not applicable (not tested or does not yield a clean product).

## Results and Discussion

### Structural Aspects

The thorium bipyridyl metallocenes [η^5^-1,2,4-(Me_3_*E*)_3_C_5_H_2_]_2_Th(bipy) (*E* = C (**1**)^139^ and Si (**1′**))^148^ are known complexes, featuring a dianionic bipy ligand coordinated to a Cp′_2_Th(IV) fragment. These bent metallocenes are structurally very similar, but the Th–C and Th–N distances in **1** are slightly longer than those in its analogue **1′**(Table 2) and the Cp(cent)-Th-Cp(cent) angle for **1** is slightly increased. Both aspects can be readily explained by the increased steric bulk exerted by the 1,2,4-(Me_3_C)_3_C_5_H_2_ ligand. Nevertheless, the longer and more flexible C–Si bonds in the 1,2,4-(Me_3_Si)_3_C_5_H_2_ might be able to accommodate changes in the metal environment more readily than its 1,2,4-(Me_3_C)_3_C_5_H_2_ counterpart. Besides these structural aspects both ligands also differ electronically in their ability to donate electron density to the Th atom. To illucidate this aspect density functional theory (DFT) investigations were performed. The natural charge analysis of the individual [(bipy)Th]^2+^ and the two [η^5^-1,2,4-(Me_3_E)_3_C_5_H_2_]^−^ fragments reveal values of 0.643, −0.345, and −0.298 for **1′**, and 0.571, −0.303, and −0.268 for **1**, respectively (Table 3), which points to an increased charge separation and also an increased Lewis acidity at the Th^4+^ ion in **1′**. This is also in line with the increased natural charge on Th of 1.575 for **1′**compared to that of 1.560 for **1**, attributed to the electron withdrawing nature of Me_3_Si groups, in which the natural charges on Me_3_Si groups (0.502–0.518) in **1′**are larger than those on Me_3_C groups (0.050–0.066) in **1** (Table 3). This aspect might also influence the reactivity of the complexes **1** and **1′**.^148^

**Table 2:** Selected Distances (Å) and Angles (deg) for Compounds **2**–**23**^a^

compound	C(Cp)–Th^b^	C(Cp)–Th^c^	Cp(cent)–Th	Th–*X*	Cp(cent)–Th–Cp(cent)	*X*–Th–*X*/*Y*
**1** (Me_3_C)^139^	2.881(56)	2.811(5) to 2.988(5)	2.606(5), 2.622(5)	N(1) 2.325(5), N(2) 2.363(4)	134.5(5)	73.4(2)
**1′**(Me_3_Si)^148^	2.833(44)	2.782(8) to 2.918(7)	2.538(8), 2.585(8)	N(1) 2.308(7), N(2) 2.339(6)	130.5(2)	73.9(2)
**1″** (U)^70^	2.843(48)	2.790(5) to 2.904(5)	2.573(5), 2.573(5)	N(1) 2.429(4), N(2) 2.429(4)	140.6(1)	66.1(2)
**2**	2.838(60)	2.780(6) to 2.928(6)	2.566(6), 2.570(6)	I(1) 3.037(1), I(2) 3.011(1)	138.1(2)	96.7(1)
**3**	2.879(61)	2.802(5) to 2.964(5)	2.601(5), 2.625(5)	N(1) 2.491(4), N(2) 2.577(5)	133.8(2)	63.3(1)^d^
				I(1) 3.148(1)		
**4**	2.885(60)	2.803(10) to 2.964(9)	2.625(9), 2.611(9)	N(1) 2.281(9), N(2) 2.283(8)	143.1(3)	94.4(4)
**5**	2.865(43)	2.825(7) to 2.912(7)	2.598(7)	S(1) 2.824(2), S(2) 2.789(2)		62.4(2)^d^
				S(3) 2.807(2)		
**6**	2.886(62)	2.767(13) to 2.952(15)	2.605(13), 2.634(13)	S(1) 2.739(4), S(2) 2.710(3) N(1) 2.580(11)	136.0(4)	44.9(1)^e^
**7**	Th(1)	Th(1)	Th(1)	Th(1)		
	2.806(51)	2.757(15) to 2.880(15)	2.537(15)	Se(1) 3.006(2), Se(2) 3.069(2)		
	Th(2)	Th(2)	Th(2)	Se(3) 2.918(1), Se(4) 2.917(1)		
	2.825(58)	2.772(14) to 2.914(15)	2.542(15)	Se(6) 3.056(2), Se(7) 3.005(2)		
				Th(2)		
				Se(1A) 3.034(2), Se(2A) 2.999(2)		
				Se(4) 2.919(1), Se(5) 2.906(1)		
				Se(6) 3.002(2), Se(7) 3.071(2)		
**8**	2.849(41)	2.805(11) to 2.901(9)	2.581(10), 2.575(10)	S(1) 2.671(3), S(2) 2.671(2)	143.9(3)	70.9(1)
**9**	Th(1)	Th(1)	Th(1)	Th(1)		82.3(1)^f^
	2.821(52)	2.744(7) to 2.889(7)	2.548(7)	S(1) 2.850(2), S(2) 2.647(2)		
				S(2A) 2.704(2)		
**10**	2.948(92)	2.808(13) to 3.104(13)	2.695(13), 2.682(13)	N(1) 2.569(14), N(2) 2.427(11)	129.4(4)	62.1(4)^d^
				O(1) 2.157(9)		
**11**	2.978(117)	2.814(5) to 3.137(5)	2.720(5), 2.730(5)	N(1) 2.572(5), N(2) 2.390(5)	139.1(2)	63.5(2)^d^
				O(1) 2.148(4)		
**12**	2.933(76)	2.821(5) to 3.058(5)	2.672(5), 2.674(5)	N(1) 2.593(4), N(2) 2.405(4)	135.0(1)	63.1(1)^d^
				O(1) 2.172(3)		
**13**	2.934(75)	2.833(5) to 3.060(5)	2.674(5), 2.674(5)	N(1) 2.596(5), N(2) 2.389(5)	134.6(2)	63.3(2)^d^
				O(1) 2.177(3)		
**14**	2.929(82)	2.810(10) to 3.058(9)	2.689(9), 2.649(9)	N(1) 2.590(9), N(2) 2.441(8)	127.5(3)	64.5(3)^d^
				Se(1) 2.880(1)		
**15**	Th(1)	Th(1)	Th(1)	Th(1)		Th(1)
	2.930(51)	2.862(5) to 2.994(5)	2.669(5)	N(1) 2.600(4), N(2) 2.495(4)		63.1(1)^d^
	Th(2)	Th(2)	Th(2)	N(3) 2.243(4), N(6) 2.321(4)		Th(2)
	2.910(34)	2.858(4) to 2.940(5)	2.648(5)	C(62) 2.697(5)		62.8(1)^g^
				Th(2)		
				N(3) 2.330(4), N(4) 2.599(4)		
				N(5) 2.504(4), N(6) 2.243(4)		
				C(28) 2.702(5)		
**16**	2.915(47)	2.843(7) to 2.973(7)	2.667(7), 2.637(7)	N(1) 2.657(7), N(2) 2.397(7)	134.6(2)	62.4(2)^d^
				N(3) 2.293(6)		
**17**	2.921(65)	2.835(6) to 3.038(6)	2.658(6), 2.660(6)	N(1) 2.602(5), N(2) 2.400(5)	133.8(2)	63.5(2)^d^
				N(3) 2.322(5)		
**18**	2.912(46)	2.842(3) to 2.987(3)	2.659(3), 2.639(3)	N(1) 2.660(3), N(2) 2.398(3)	134.4(1)	62.6(1)^d^
				N(3) 2.312(3)		
**19**	2.914(49)	2.844(4) to 2.970(4)	2.667(4), 2.634(4)	N(1) 2.607(3), N(2) 2.402(3)	132.3(1)	63.6(1)^d^
				N(3) 2.304(3)		
**20**	Th(1)	Th(1)	Th(1)	Th(1)	Th(1)	Th(1)
	2.921(67)	2.838(5) to 3.002(5)	2.681(5), 2,640(5)	N(1) 2.612(4), N(2) 2.394(4)	139.6(1)	62.9(1)^d^
	Th(2)	Th(2)	Th(2)	N(3) 2.282(4)	Th(2)	Th(2)
	2.916(58)	2.835(4) to 2.996(5)	2.668(5), 2.641(5)	Th(2)	139.1(1)	62.9(1)^g^
				N(4) 2.635(4), N(5) 2.388(4)		
				N(6) 2.296(3)		
**21**	2.932(89)	2.811(8) to 3.114(7)	2.684(7), 2.663(7)	N(1) 2.561(7), N(2) 2.420(6)	132.3(2)	63.2(2)^d^
				O(1) 2.214(4)		
**22**	2.884(46)	2.832(5) to 2.958(4)	2.615(4), 2.621(4)	N(1) 2.703(4), N(2) 2.364(4)	133.6(1)	62.0(1)^d^
				N(3) 2.566(5)		
**23**	2.886(42)	2.804(10) to 2.953(10)	2.619(9), 2.617(9)	N(1) 2.711(10), N(2) 2.368(9)	136.9(3)	61.8(3)^d^
				N(3) 2.546(12)		

a Cp = cyclopentadienyl ring.

b Average value, the value in parentheses is the standard deviation of the mean.

c Range.

d The angle of N(1)–Th–N(2).

e The angle of S(1)–Th–S(2).

f The angle of S(2)–Th–S(2A).

g The angle of N(4)–Th(2)–N(5).

**Table 3:** Natural Charges for Thorium, [η^5^-1,2,4-(Me_3_E)_3_C_5_H_2_], [Me_3_E], [bipy], and [Th(bipy)] Units in [η^5^-1,2,4-(Me_3_E)_3_C_5_H_2_]2Th(bipy) (E = C (**1**), Si (**1′**))

	**1** (E = C)	**1′** (E = Si)
Th	1.560	1.575
[η^5^-1,2,4-(Me_3_*E*)_3_C_5_H_2_]	–0.303	–0.345
[Me_3_*E*]	0.057	0.510
	0.050	0.515
	0.066	0.518
[η^5^-1,2,4-(Me_3_*E*)_3_C_5_H_2_]′	–0.268	–0.298
[Me_3_*E*]	0.060	0.502
	0.065	0.504
	0.066	0.513
[bipy]	–0.989	–0.932
[Th(bipy)]	0.571	0.643

It is also instructive to compare the bonding situation between the Th derivative **1** and its uranium counterpart (Cp^3*t*Bu^)_2_U(bipy) (**1″**).^70^ In contrast to **1** featuring a Th^4+^ ion being coordinated by a bipyridine dianion, the related U complex (Cp^3*t*Bu^)_2_U(bipy) (**1″**)^70^ features a U(III) atom bound to a bipyridyl radical anion, which leads to increased An–N distances in **1″** whereas the An–C(Cp) distances are only marginally shortened in **1″** (Table 2). Both aspects render the uranium atom less Lewis acidic and slightly more sterically accessible for an interaction with small molecules. In addition, electron transfer process in the uranium case might originate from the U^3+^ ion, the bipyridyl ligand or jointly from the U^3+^ and the bipyridyl ligand (Table 1).

### Reactivity Studies

To evaluate the differences and similarities in the reactivity of **1** and **1′**, we exposed complex **1** to several organic molecules and compared the isolated reaction products to those obtained with (Cp^3tms^)_2_Th(bipy) (**1′**),^148^ Cp*_2_Th(bipy) and (Cp^2*t*Bu^)_2_Th (bipy) (Table 1),^142,149^ and its uranium analogue (Cp^3*t*Bu^)_2_U(bipy) (**1″**; Table 1).^147^ In the interest of the reader we discuss these results grouped according to substance classes.

### Reaction with Alkynes, CuI, and Hydrazine Derivatives

Attributed to the sterically demanding nature of the 1,2,4-(Me_3_C)_3_C_5_H_2_ ligand, no reaction occurs when (Cp^3*t*Bu^)_2_Th(bipy) (**1**) is exposed to terminal and internal alkynes, such as PhC≡CH and PhC≡CC≡CPh. In this respect the most sterically demanding derivatives (Cp^3*t*Bu^)_2_Th(bipy) (**1**), (Cp^3tms^)_2_Th(bipy) (**1′**), and (Cp^3*t*Bu^)_2_U(bipy) (**1″**) behave similarly, but the less sterically encumbered derivatives such as Cp*_2_Th(bipy) and (Cp^2*t*Bu^)_2_Th (bipy) do react (Table 1).^142,147−149^

**Scheme 1:**
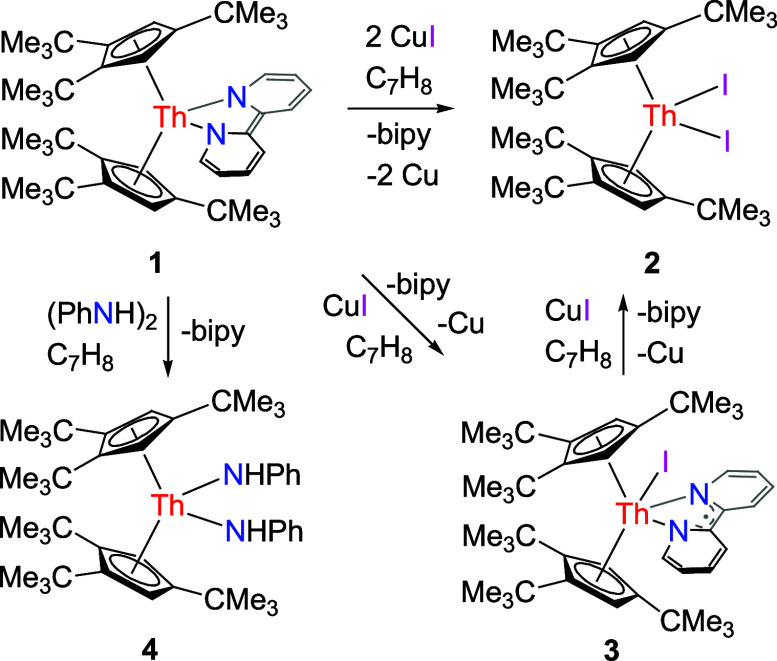
Synthesis of Compounds **2**–**4**

**Figure 1:**
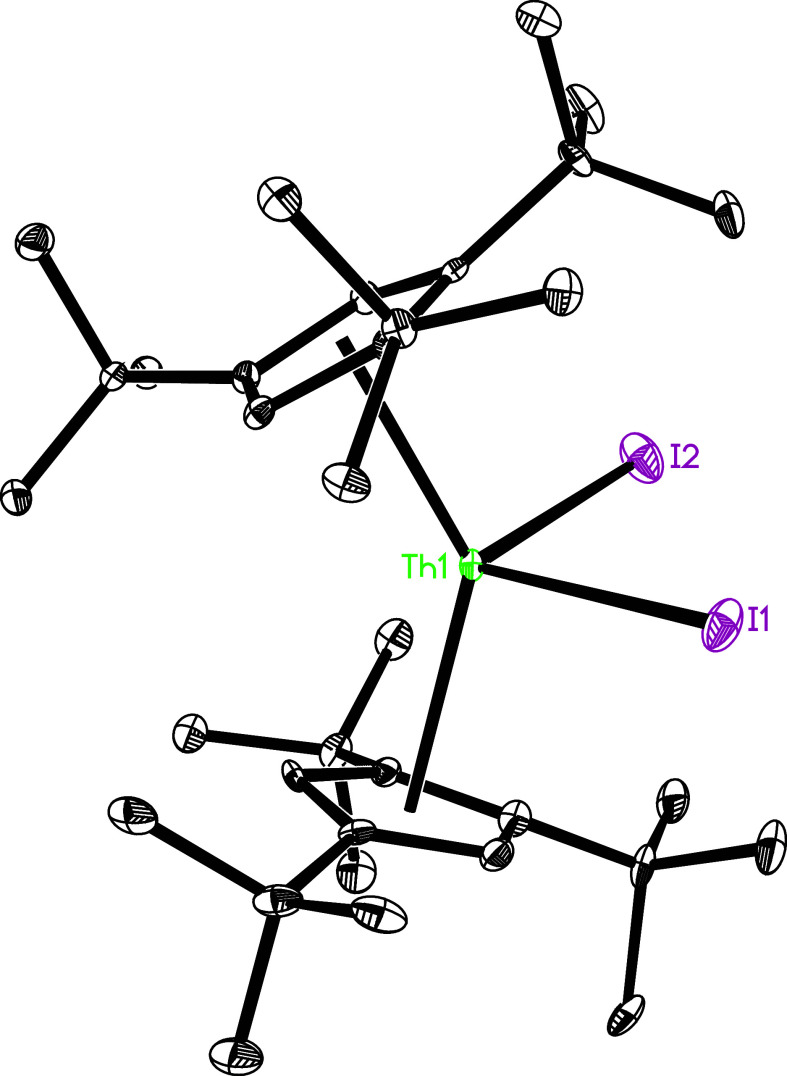
Molecular structure of **2** (thermal ellipsoids drawn at the 35% probability level).

**Figure 2:**
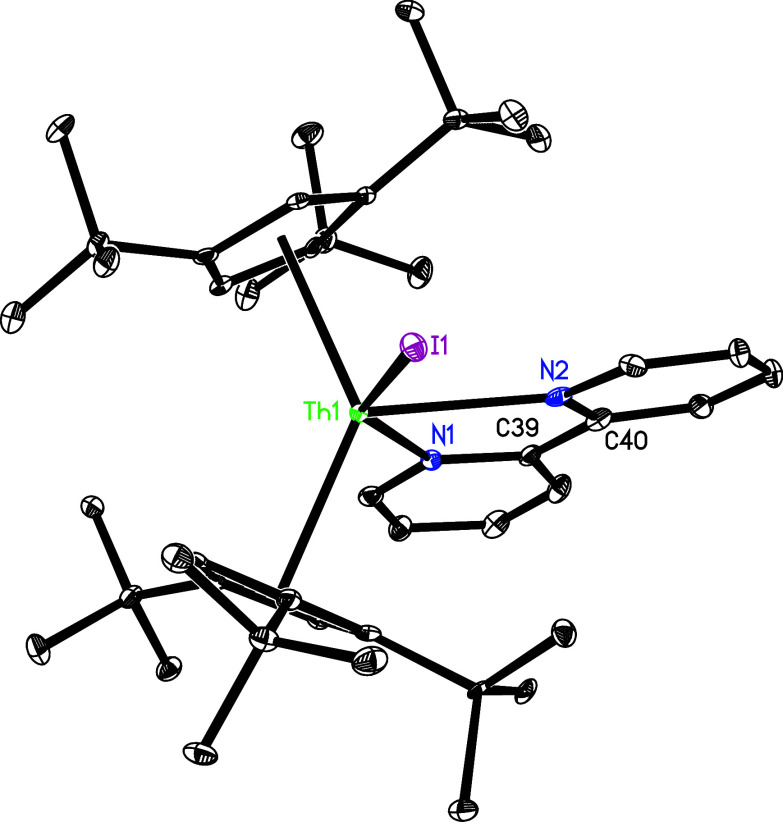
Molecular structure of **3** (thermal ellipsoids drawn at the 35% probability level).

Nevertheless, like the thorium bipyridyl metallocene (Cp^3tms^)_2_Th(bipy) (**1′**) (Table 1),^148^ the addition of 2 equiv of CuI to complex **1** exclusively yields the Th(IV) diiodide (Cp^3*t*Bu^)_2_ThI_2_ (**2**), Cu metal, and free bipy in quantitative conversion (Scheme 1). This reactivity contrasts that of (Cp^3*t*Bu^)_2_U(bipy) (**1″**), that forms with CuI exclusively the U(III) iodide (Cp^3*t*Bu^)_2_UI (Table 1).^147^ Several aspects can be invoked to account for this reactivity difference: (1) The enhanced thermodynamic stability of U(III) vs Th(III) relative to that of U(IV) vs Th(IV). (2) The different electronic structure of the actinide bipy complexes (Cp^3*t*Bu^)_2_An(bipy) (An = Th (**1**), U (**1″**)), which also reflects the former aspect. (3) The more crowded environment around a U^4+^ ion (r(Th^4+^)_C.N.=8_ = 1.05 Å vs r(U^4+^)_C.N.=8_ = 1.00 Å).^150^ Nevertheless, in contrast to the reactivity of complex **1** with Ag*X* (*X* = F, Cl, Br),^139^ the reaction of **1** with AgI is complicated and no pure compound is isolated. The molecular structure of **2** is shown in Figure 1, and selected bond distances and angles are listed in Table 2. The Th–I distances are 3.037(1) and 3.011(1) Å, and the I–Th–I angle is 96.7(1)°, which are comparable to those found in (Cp^3tms^)_2_ThI_2_ with Th–I distances of 3.022(1) and 3.018(1) Å, and a I–Th–I angle of 93.8(1)°.^148^ Nevertheless, in the related thorium complex (Cp^3*t*Bu^)_2_ThCl_2_, the Th–Cl distances are 2.619(1) and 2.624(1) Å and the Cl–Th–Cl angle is 99.4(1)°.^151^ However, treatment of complex **1** with 1 equiv of CuI gives the Th(IV) bipyridyl iodide complex (Cp^3*t*Bu^)_2_Th(I)(bipy) (**3**) featuring a coordinated bipyridyl radical anion in quantitative conversion (Scheme 1). For comparison only the Th(IV) diiodide complex (Cp^3tms^)_2_ThI_2_ is formed for the closely related derivative (Cp^3tms^)_2_Th(bipy) (**1′**; Table 1), presumable due to the less steric demanding nature of the 1,2,4-(Me_3_Si)_3_C_5_H_2_ ligand.^148^ Nevertheless, complex **3** reacts with a second molecule of CuI to yield complex **2** (Scheme 1). The molecular structure of **3** is shown in Figure 2, and selected bond distances and angles are listed in Table 2. The Th–I distance of 3.148(1) Å is significantly longer than those observed in complex **2** (3.037(1) and 3.011(1) Å), which we attribute to the steric bulk imposed by the bipy ligand. The Th–N(1) and Th–N(2) distances are 2.491(4) and 2.577(5) Å, respectively, which are longer than those found in complex **1** (2.325(5) and 2.363(4) Å), attributed to the different coordination number, the steric bulk imposed by the iodine atom and the oxidation of the [bipy]^2–^ to radical anion [bipy]·^–^. The C(39)–C(40) distance is 1.425(8) Å, which is longer than those (bipy^2–^) found in (Cp^3*t*Bu^)_2_Th(bipy) (**1**) (1.382(8) Å),^139^ Cp*_2_Th(bipy) (1.402(6) Å),^142^ (Cp^2*t*Bu^)_2_Th(bipy) (1.396(12) Å), and (Cp^3tms^)_2_Th(bipy) (**1′**; 1.396(11) Å),^140,148^ but it is close to those (bipy•^–^) found in K(bipy)(en) (1.431(3) Å),^152^ (Cp^3*t*Bu^)_2_U(bipy) (**1″**; 1.426(12) Å), and (Cp^3tms^)_2_U(bipy) (1.421(14) Å),^70,145^ supporting bipy radical anion description of coordinated bipy ligand in **3**. Moreover, the UV/vis spectrum of **3** has obvious absorptions at 382, 457, 871, 966, 1079, and 1252 nm in toluene (see Supporting Information), also supporting a reduced bipyridine radical species.^91,152−155^

**Figure 3:**
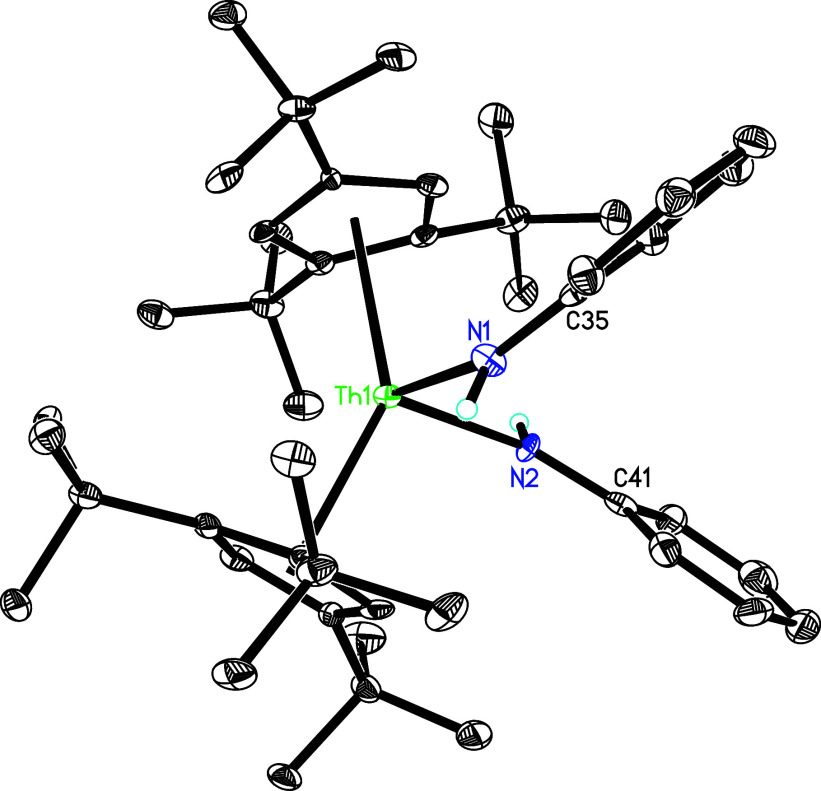
Molecular structure of **4** (thermal ellipsoids drawn at the 35% probability level).

Moreover, like the reactivity of (Cp^3*t*Bu^)_2_U(bipy) (**1″**; Table 1),^147^ treatment of **1** with the hydrazine derivative (PhNH)_2_ gives the bis-amido species (Cp^3*t*Bu^)_2_Th(NHPh)_2_ (**4**) in quantitative conversion along with bipy loss (Scheme 1). The molecular structure of **4** is provided in Figure 3, and selected bond distances and angles are listed Table 2. The Th–N distances are 2.281(9) Å for N(1) and 2.283(8) Å for N(2), and the N(1)–Th–N(2) angle is 94.4(4)°.

### Reaction with Chalcogen Derivatives

**Scheme 2:**
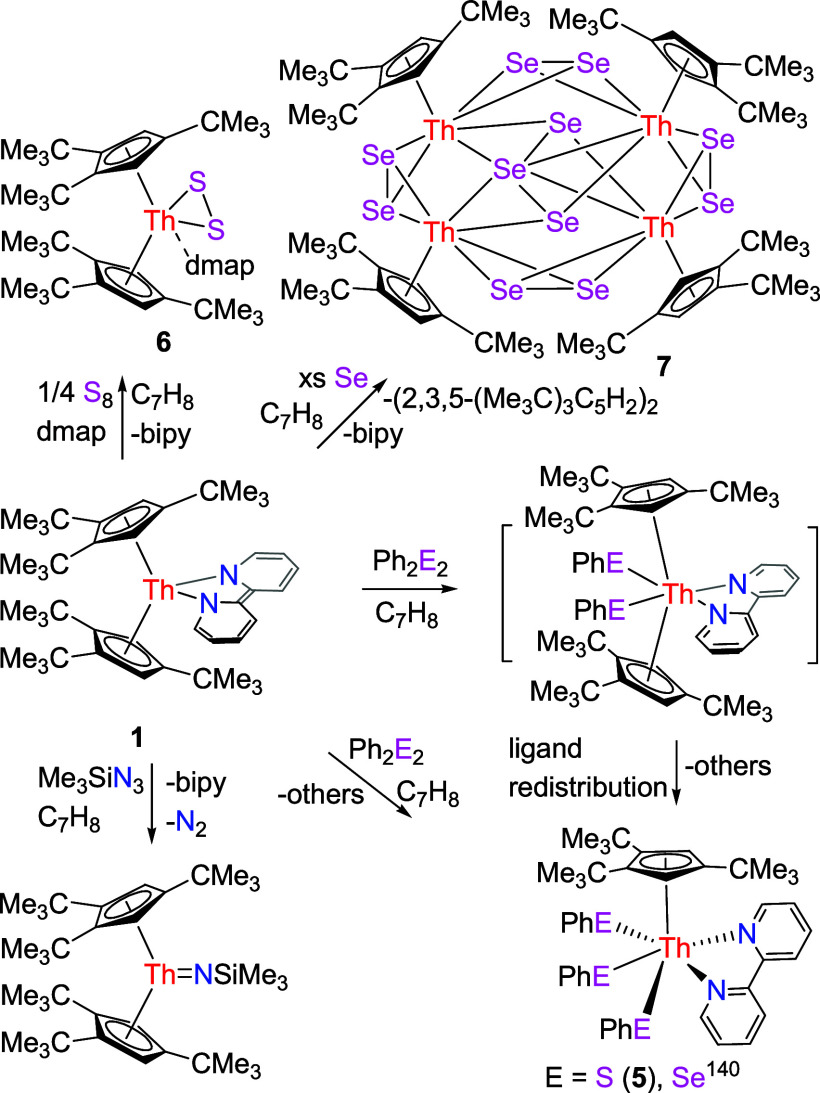
Synthesis of Compounds **5**–**7**

**Figure 4:**
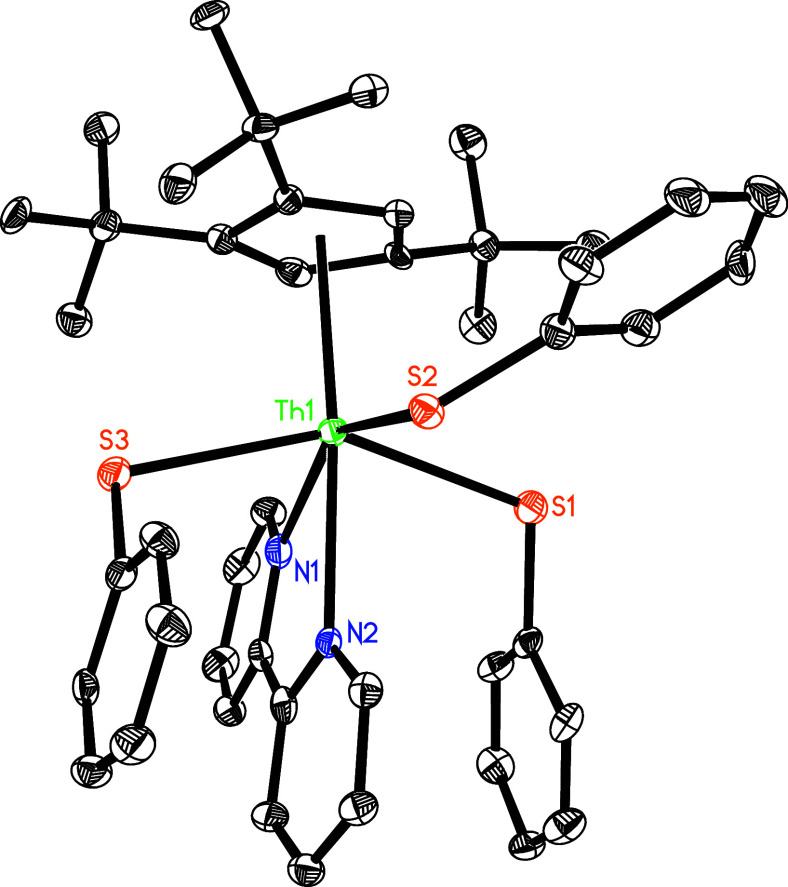
Molecular structure of **5** (thermal ellipsoids drawn at the 35% probability level).

However, contrary to the reactivity of the thorium bipyridyl metallocenes (Cp^3tms^)_2_Th(bipy) (**1′**),^148^ Cp*_2_Th(bipy) and (Cp^2*t*Bu^)_2_Th (bipy) (Table 1),^140,142,149^ treatment of complex **1** with Ph_2_S_2_ or Ph_2_Se_2_ does not yield (Cp^3*t*Bu^)_2_Th(*E*Ph)_2_ (*E* = S, Se), instead, the thorium tris(phenylsulfido) and tri(phenylselenido) complexes (Cp^3*t*Bu^)Th(SPh)_3_(bipy) (**5**) and (Cp^3*t*Bu^)Th(SePh)_3_(bipy)^139^ were isolated in 26% and 34% yield, respectively (Scheme 2). This difference presumably stems from the enhanced steric bulk of the 1,2,4-(Me_3_C)_3_C_5_H_2_ ligand, facilitating ligand redistribution processes.^139^ However, the related uranium derivative (Cp^3*t*Bu^)_2_U(bipy) (**1″**) forms exclusively (Cp^3*t*Bu^)_2_U(*E*Ph)_2_ (*E* = S, Se) (Table 1).^147^ This difference might be a manifestation of the softer nature of the U^4+^ ion and increased covalence in the U–*E* bond,^30,156,157^ these aspects combined with the smaller U^4+^ ion appear to be sufficient to effectively prevent ligand reorganization. The molecular structure of **5** is shown in Figure 4, and selected bond distances and angles are listed in Table 2. The Th^4+^ ion is η^5^-bound to one Cp-ring and *κN* coordinated to one bipy ligand and *κS* coordinated to three PhS groups in a distorted-octahedral-geometry. The Th–S(1), Th–S(2), and Th–S(3) distances are 2.824(2), 2.789(2), and 2.807(2) Å, respectively, whereas the much longer Th–N(1) and Th–N(2) distances of 2.612(7) and 2.618(6) Å, respectively, are consistent with a datively coordinated nitrogen atom. Moreover, the C–C distance between two pyridine is 1.480(11) Å, which is longer than that found in **1** (1.382(8) Å) and **3** (1.425(8) Å), supporting a neutral bipy ligand.^139^

**Figure 5:**
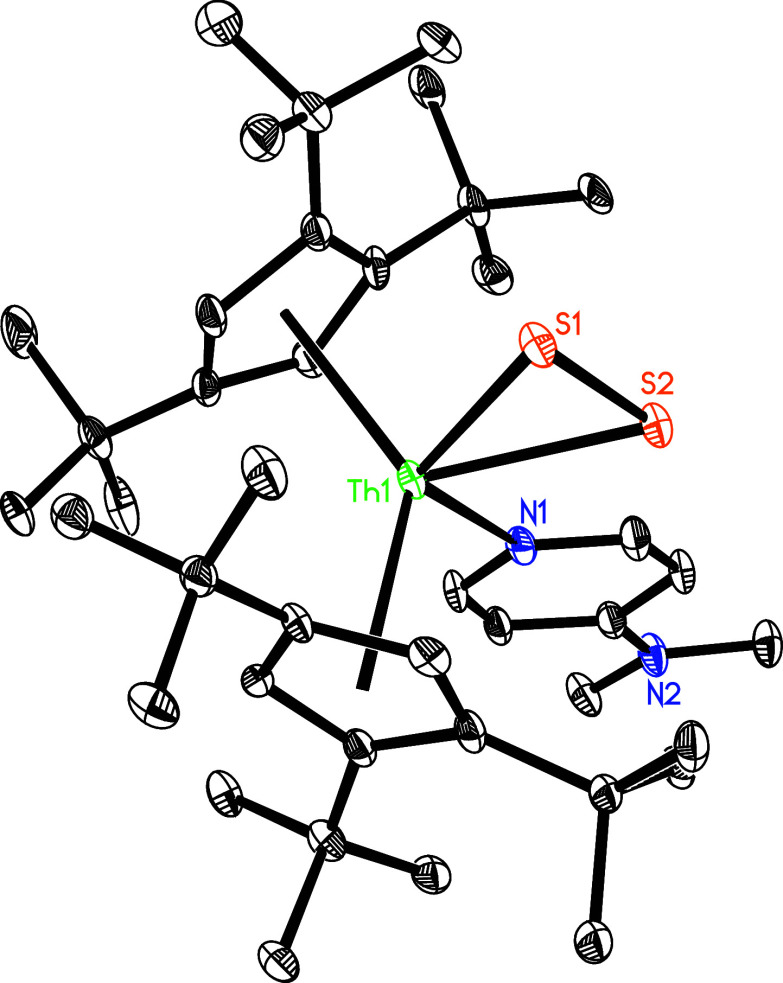
Molecular structure of **6** (thermal ellipsoids drawn at the 35% probability level).

Nevertheless, in analogy to the reactivity of the thorium bipyridyl metallocene Cp*_2_Th(bipy) but contrary to (Cp^2*t*Bu^)_2_Th (bipy) (Table 1),^142,149^ treatment of complex **1** with elemental sulfur (S_8_) in the presence of 4-(dimethylamino)pyridine (dmap) forms free bipy and the disulfide complex (Cp^3*t*Bu^)_2_ThS_2_(dmap) (**6**) (Scheme 2), presumably attributed to the increased steric bulk of the C_5_Me_5_ and 1,2,4-(Me_3_C)_3_C_5_H_2_ ligands. Nevertheless, in the presence of pyridine or THF, the reaction of **1** with S_8_ does not yield a pure compound, presumably due to the reduced Lewis basicity of pyridine and THF. The molecular structure of **6** is shown in Figure 5, and selected bond distances and angles are listed in Table 2. The Th–S distances are 2.739(4) and 2.710(3) Å, whereas the Th–N distance is 2.580(11) Å, consistent with a datively coordinated nitrogen atom. Moreover, the S–S distance is 2.083(5) Å, and the S–Th–S angle is 44.9(1)°.

**Figure 6:**
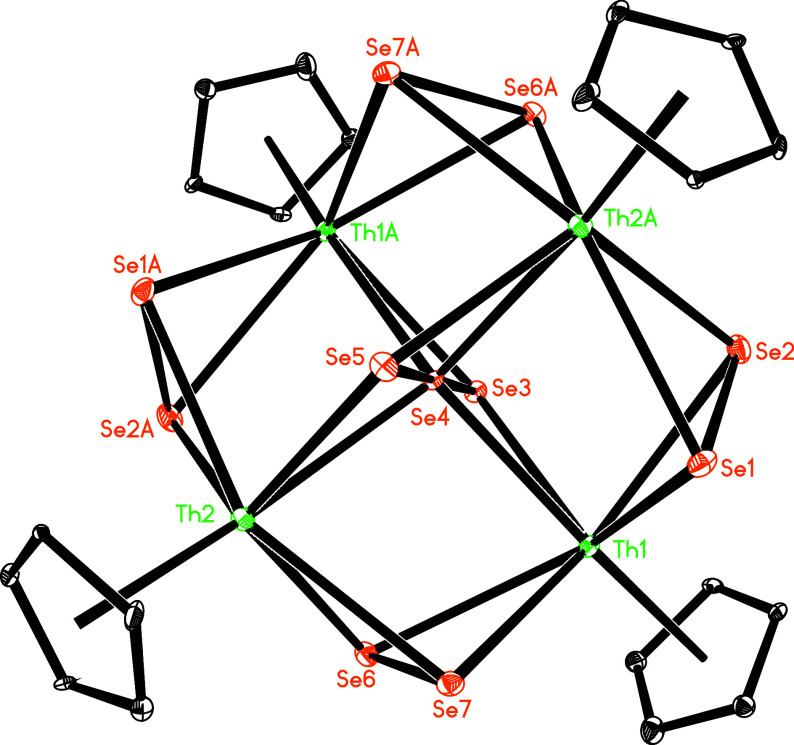
Molecular structure of **7** (*tert*-butyl group omitted for clarity, thermal ellipsoids drawn at the 35% probability level).

Nevertheless, like the reactivity of the uranium bipyridyl complex (Cp^3*t*Bu^)_2_U(bipy) (**1″**) but contrary to that of the thorium bipy complex (Cp^2*t*Bu^)_2_Th (bipy) (Table 1),^147,149^ addition of an excess of elemental selenium to complex **1** yields the thorium selenido cluster [(Cp^3*t*Bu^)Th]_4_(μ-Se_11_) (**7**), neutral bipy and (2,3,5-(Me_3_C)_3_C_5_H_2_)_2_ (Scheme 2), as a result of the steric bulk and electron richness of the 1,2,4-(Me_3_C)_3_C_5_H_2_ ligand.^138^ Complex **7** forms as a tetranuclear thorium selenido cluster, in which each Th atom is η^5^-bound to one Cp-ring and σ-bound to six selenium atoms, and the coordination environment in [(Cp^3*t*Bu^)Th(Se)_6_] fragment can be described as a significantly distorted hexagonal-pyramid (Figure 6). The bridging Se-units can be grouped into four μ_2_-η^2^:η^2^-Se_2_ units on the periphery and one central linear μ_4_-η^2^:η^2^:η^2^:η^2^-Se_3_ unit, in which two Se atoms coordinate to two Th atoms. The Th(1)–Se distances of 3.006(2), 3.069(2), 2.918(1), 2.917(1), 3.056(2), and 3.005(2) (1) Å are close to the Th(2)–Se distances of 3.034(2), 2.999(2), 2.919(1), 2.906(1), 3.002(2), and 3.071(2) Å. Moreover, the Se–Se distances within the μ_2_-Se_2_ units is within the 3σ-criterion identical with Se(1)–Se(2) (2.360(3) Å) and Se(6)–Se(7) (2.366(2) Å), whereas those of the central μ_4_-Se_3_ core are much longer with 2.641(3) Å and 2.657(3) Å for Se(3)–Se(4) and Se(4)–Se(5), respectively. Overall, these structural data are reminiscent to those found in its U(IV) analogue [(Cp^3*t*Bu^)U]_4_(μ-Se_11_).^147^

### Reaction with Organic Azides, CS_2_, and Isothiocyanates

Moreover, similar to the reactivity of the thorium bipyridyl complexes [(3-Mes-C_3_H_2_N_2_)_2_BH_2_]_2_Th(bipy),^97^ (Cp^3tms^)_2_Th(bipy) (**1′**),^148^ (Cp^2*t*Bu^)_2_Th(bipy) and Cp*_2_Th (bipy) (Table 1),^142,149^ complex **1** readily reacts with various heterounsaturated organic molecules. For example, like the uranium bipyridyl complex (Cp^3*t*Bu^)_2_U(bipy) (**1″**; Table 1),^147^ a two-electron transfer process with concomitant bipy and N_2_ release is observed upon addition of Me_3_SiN_3_ to **1**, in which the imido complex (Cp^3*t*Bu^)_2_Th=NSiMe_3_ is formed in quantitative conversion (Scheme 2),^139^ but diverging reactivity is observed for the less sterically encumbered derivatives Cp*_2_Th(bipy), (Cp^2*t*Bu^)_2_Th(bipy) and (Cp^3tms^)_2_Th(bipy) (**1′**; Table 1).^142,148,149^ The reactivity differences between (Cp^3*t*Bu^)_2_Th(bipy) (**1**) forming a terminal imido product (Cp^3*t*Bu^)_2_Th=NSiMe_3_ (Scheme 2),^139^ Cp*_2_Th(bipy) affording an azido amido complex Cp*_2_Th[N(SiMe_3_)_2_](N_3_) (Table 1),^142^ (Cp^3tms^)_2_Th(bipy) (**1′**) giving a bis-amido complex (Cp^3tms^)_2_Th(NHSiMe_3_)_2_ (Table 1),^148^ and (Cp^2*t*Bu^)_2_Th(bipy) yielding an amido pyridyl complex (Cp^2*t*Bu^)_2_Th(NHSiMe_3_)(*κC*,*κN*-C_10_H_7_N_2_) (Table 1)^149^ can be attributed to the different steric and electronic effects of the coordinated Cp ligands, in which (Cp^3*t*Bu^)_2_Th fragment provides the required steric protection for the imido complex (Cp^3*t*Bu^)_2_Th=NSiMe_3_ (Scheme 2).^139^

**Scheme 3:**
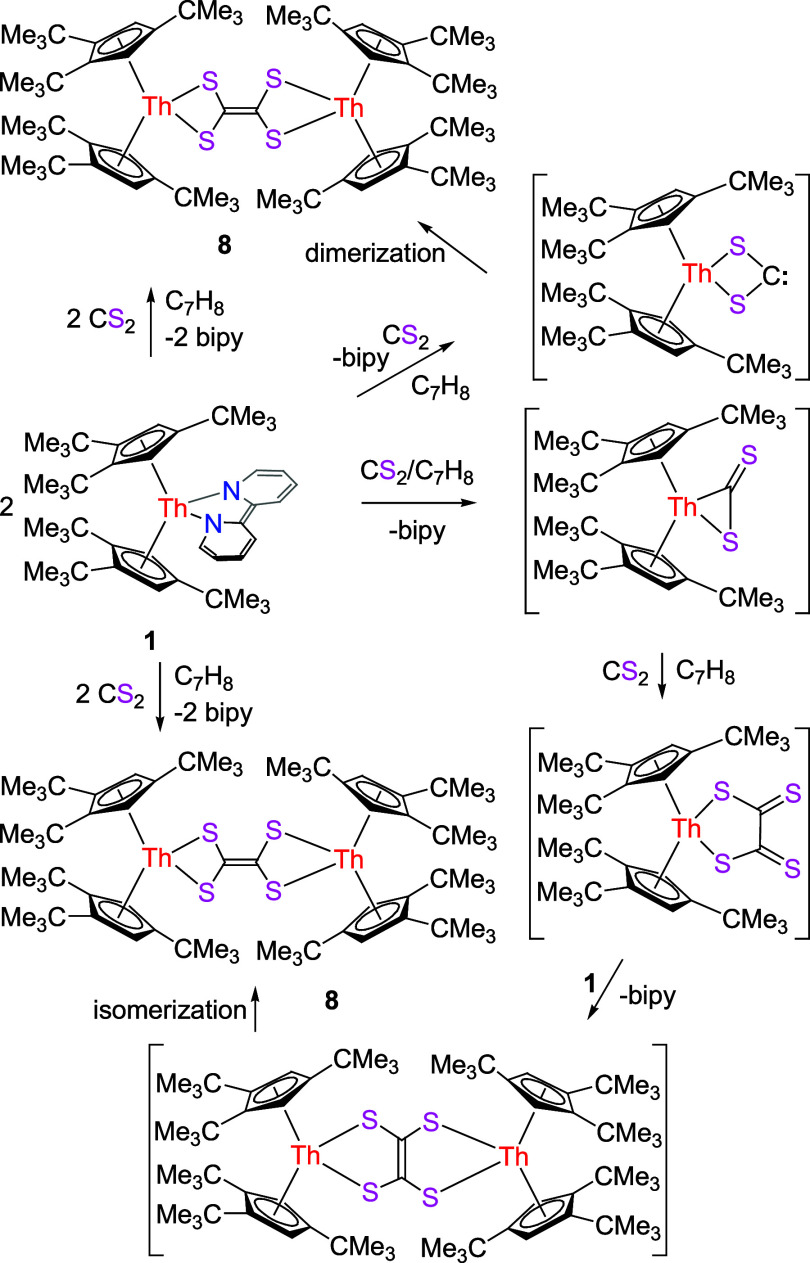
Synthesis of Compound **8**

**Figure 7:**
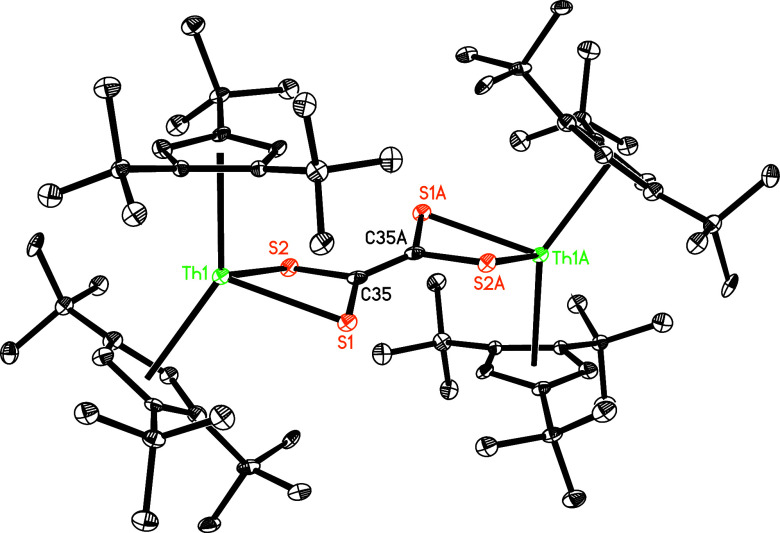
Molecular structure of **8** (thermal ellipsoids drawn at the 35% probability level).

Moreover, two-electrons are transferred when 1 equiv of CS_2_ is added to complex **1**, which irreversibly yields the terminal ethylenetetrathiolate complex [(Cp^3*t*Bu^)_2_Th]_2_(μ-S_2_C=CS_2_) (**8**) along with free bipy (Scheme 3). Although no intermediates were spectroscopically detected, it is reasonable to assume that CS_2_ initially reacts with **1** to substitute the bipy ligand and to form an intermediate with a single four-membered ring (Scheme 3; up), which dimerizes by carbene (RR′C:) coupling to yield **8** (Scheme 3). Alternately, CS_2_ initially reacts with **1** to substitute the bipy ligand and to form a metallathiirene intermediate, which couples with a second molecule of CS_2_ to give a tetrathio-oxalate, which further reacts with a second molecule of **1** to yield the side-on coordinate ethylenetetrathiolate intermediate with bipy loss (Scheme 3; down). Nevertheless, this intermediate is unstable and rearranges to the final product **8** (Scheme 3). The differences in reactivity between (Cp^2*t*Bu^)_2_Th(bipy) forming a dimeric sulfido complex [(Cp^2*t*Bu^)_2_Th]_2_(μ-S)_2_ (Table 1),^140^ Cp*_2_Th(bipy) affording a dimeric trithiocarbonate complex (Cp*_2_Th)_2_(μ-CS_3_)_2_ (Table 1),^142^ (Cp^3tms^)_2_Th(bipy) (**1′**) giving a side-on coordinate ethylenetetrathiolate complex [(Cp^3tms^)_2_Th]_2_(μ-C_2_S_4_) (Table 1),^148^ and **1** yielding a terminal ethylenetetrathiolate complex **8** may again be traced to the different steric and electronic effects exerted by the Cp ligands. Hence, the metallathiirene intermediates [η^5^-1,2,4-(Me_3_*E*)_3_C_5_H_2_]_2_Th[η^2^-C(=S)S] (*E* = Si,^148^ C) can be trapped by a second equiv of CS_2_ to finally form the side-on ethylenetetrathiolate complex [(Cp^3tms^)_2_Th]_2_(μ-C_2_S_4_) (Table 1) and the terminal ethylenetetrathiolate complex **8**, respectively. The molecular structure of **8** can be found in Figure 7, the selected bond distances and angles are available in Table 2. The C(35)–C(35A) distance of 1.34(2) Å is consistent with a C=C bond but is shorter than those found in the side-on coordinate ethylenetetrathiolate complexes [(Cp^2*t*Bu^)_2_U]_2_(μ-C_2_S_4_) (1.408(12) Å),^146^ [(Cp^3tms^)_2_Th]_2_(μ-C_2_S_4_) (1.417(13) Å), and [Na(dme)_3_]_2_[{((^Ad^ArO)_3_N)U}_2_(μ-C_2_S_4_)] (1.383(8) Å).^148,158^ Moreover, in contrast to the side-on coordinate ethylenetetrathiolate thorium complex [(Cp^3tms^)_2_Th]_2_(μ-C_2_S_4_) (Table 1),^148^ the [C_2_S_4_]^2–^ ligand in **8** binds to the Th^4+^ ion by its two terminal sulfur atoms because of the steric reasons. The experimentally observed preference for terminal [S_2_C=CS_2_] over side-on [C_2_S_4_]-coordination is also reflected in DFT computations, predicting the terminal ethylenetetrathiolate linkage isomer to be energetically more favorable than its side-on counterpart [(Cp^3*t*Bu^)_2_Th]_2_(μ-C_2_S_4_) (Δ*G* (298 K) = −3.2 kcal/mol) (see Supporting Information for details). In addition, the Th(1)–S(1) (2.671(3) Å) and Th(1)–S(2) (2.671(2) Å) distances are identical, and the S–Th(1)–S angle is 70.9(1)°.

**Figure 8:**
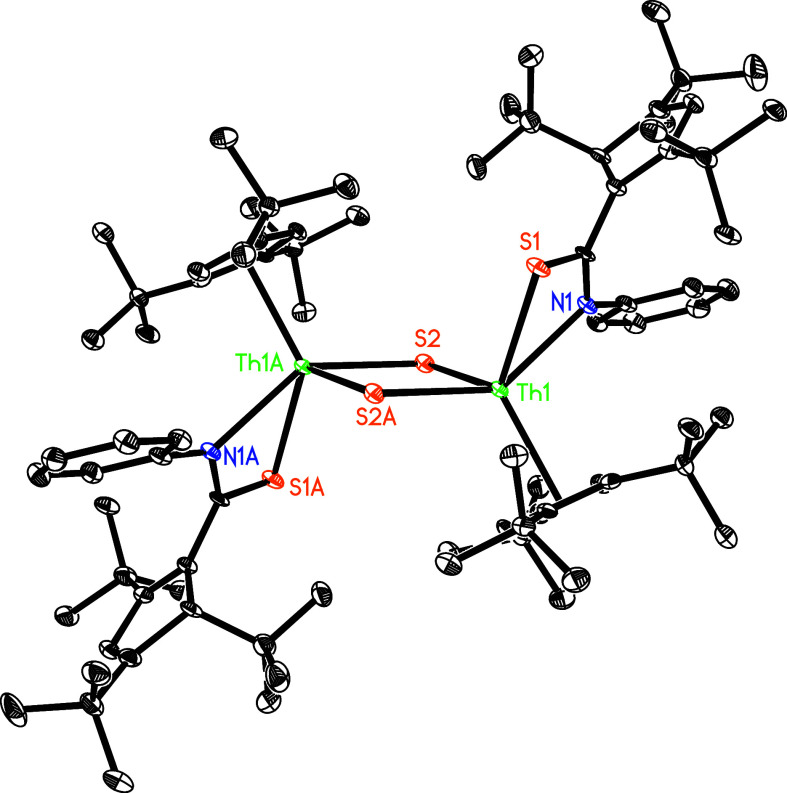
Molecular structure of **9** (thermal ellipsoids drawn at the 35% probability level).

**Scheme 4:**
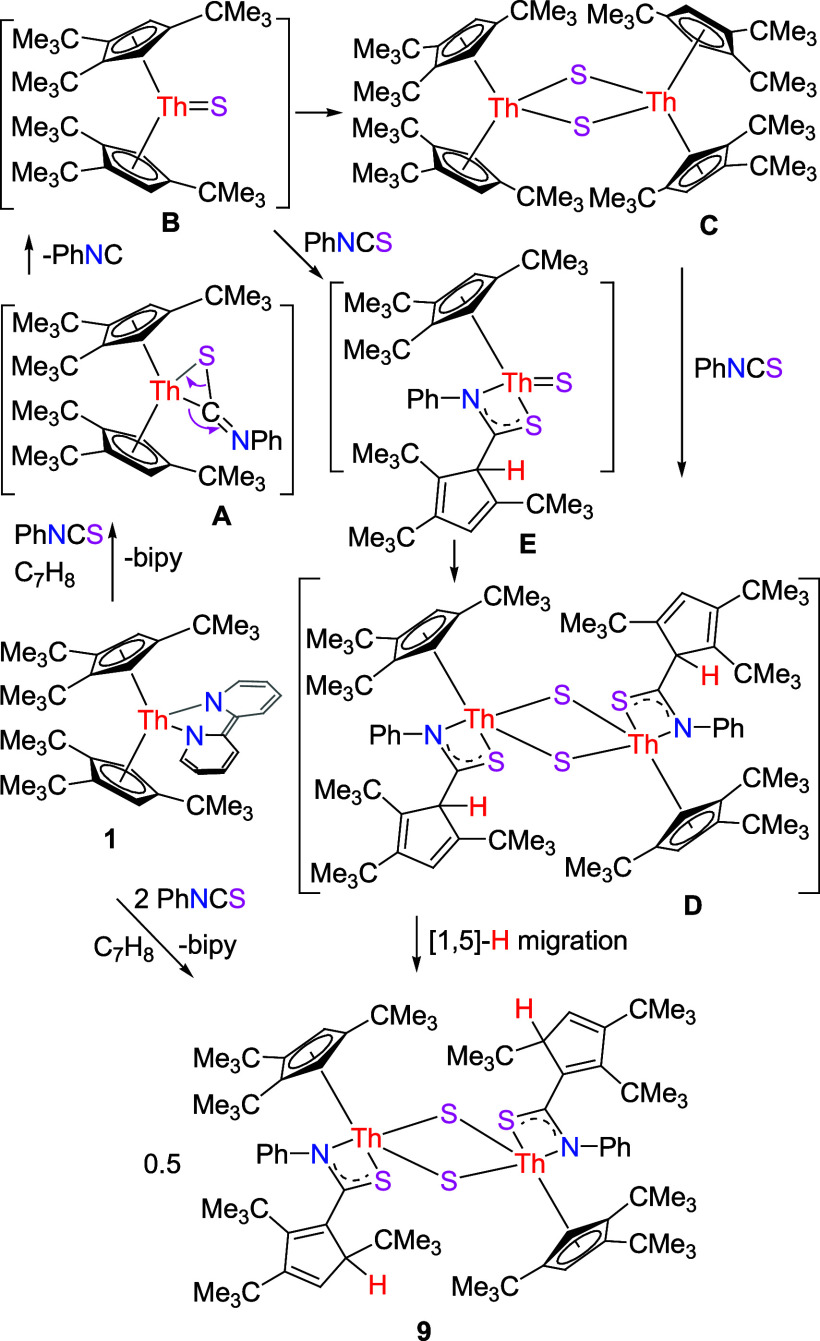
Synthesis of Compound **9**

Furthermore, two-electrons are transferred when PhNCS is added to complex **1**, which irreversibly yields the dimeric thiocarbonato sulfido complex {[(2,3,5-(Me_3_C)_3_C_5_H_2_)C(NPh)S](Cp^3*t*Bu^)Th}_2_(μ-S)_2_ (**9**) in good yield concomitant with the byproducts isonitrile PhNC and free bipy (Scheme 4), but divergent reactivity is observed for the less sterically encumbered derivatives Cp*_2_Th(bipy) and (Cp^2*t*Bu^)_2_Th (bipy) (Table 1).^142,149^ Although no intermediates were spectroscopically detected, it is reasonable to assume that PhNCS initial reacts with **1** to substitute the bipy ligand and to form a metallathiirene intermediate **A** (Scheme 4), which converts via isonitrile PhNC elimination to give a terminal sulfido complex **B**, which dimerizes to the known complex [(Cp^3*t*Bu^)_2_Th]_2_(μ-S)_2_ (**C**).^159^ In the next step, two molecules of PhNCS insert into two 1,2,4-(Me_3_C)_3_C_5_H_2_ ligands to give a dimeric thiocarbonato complex **D**. Similar to the isomerization of the (Me_3_C)_3_C_5_H_3_ ligand,^138^ intermediate **D** undergoes a [1,5]-H migration to yield the product **9**. Alternatively, when a terminal sulfido complex **B** is formed, a second molecule of PhNCS inserts into one 1,2,4-(Me_3_C)_3_C_5_H_2_ ligand to give a thiocarbonato complex **E**, which undergoes dimerization and [1,5]-H migration to yield the product **9**. It is of note that complex [(Cp^3*t*Bu^)_2_Th]_2_(μ-S)_2_ (**C**) indeed reacts with PhNCS to yield **9** (see Experimental Section for details). Moreover, PhNCS selectively inserts into the 1,2,4-(Me_3_C)_3_C_5_H_2_ ligand, forming the new C–C bond at the C–H positions rather than the C-*^t^*Bu positions for steric reasons. Furthermore, the formation of **9** is irreversible, no other species is detected even when an NMR sample of **9** is heated at 100 °C for 1 week. The differences in reactivity between (Cp^2*t*Bu^)_2_Th(bipy) forming a dimeric sulfido complex [(Cp^2*t*Bu^)_2_Th]_2_(μ-S)_2_ (Table 1),^149^ Cp*_2_Th(bipy) affording a dimeric dithiocarbonate complex (Cp*_2_Th)_2_[μ-C(=NPh)S_2_]_2_ (Table 1),^142^ and **1** yielding a dimeric thiocarbonato sulfido complex **9** may again be traced to the different steric and electronic effects exerted by the Cp ligands. Hence, the terminal sulfido intermediate Cp*_2_ThS can be trapped by a second equiv of PhNCS to form the dimeric dithiocarbonate complex (Cp*_2_Th)_2_[μ-C(=NPh)S_2_]_2_ (Table 1),^142^ whereas the intermediate (Cp^3*t*Bu^)_2_ThS forms **9** in the presence of PhNCS. Complex **9** yields as a dimeric thorium thiocarbonato sulfido complex, in which each Th atom is η^5^-bond to one Cp-ring and σ-bound to three sulfur atoms and one nitrogen atom in a distorted tetragonal-pyramide (Figure 8). The Th(1)–S(1), Th(1)–S(2), and Th(1)–S(2A) distances are 2.850(2), 2.647(2), and 2.704(2) Å, respectively, whereas the Th(1)–N(1) distance is 2.581(6) Å, and the S(2)–Th(1)–S(2A) angle is 82.3(1)°.

### Reaction with Ketones, Aldehydes, and Ketazines

**Scheme 5:**
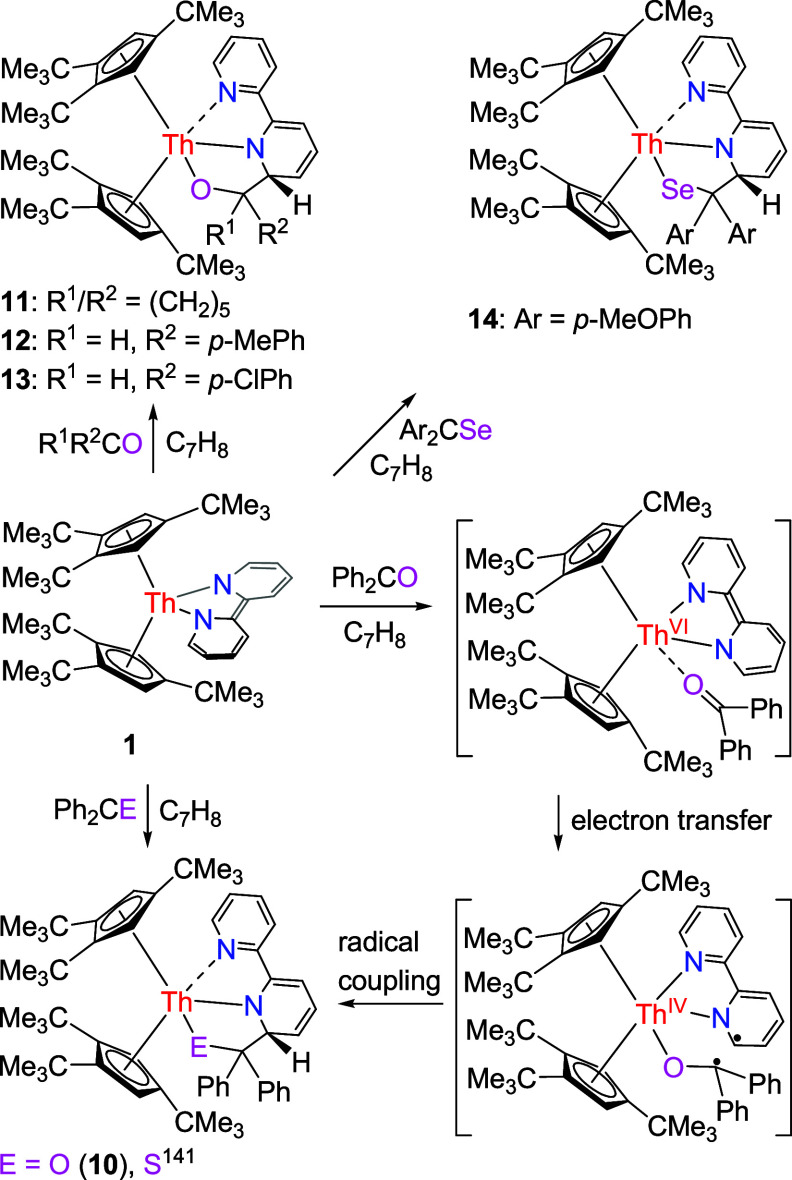
Synthesis of Compounds **10**–**14**

**Figure 9:**
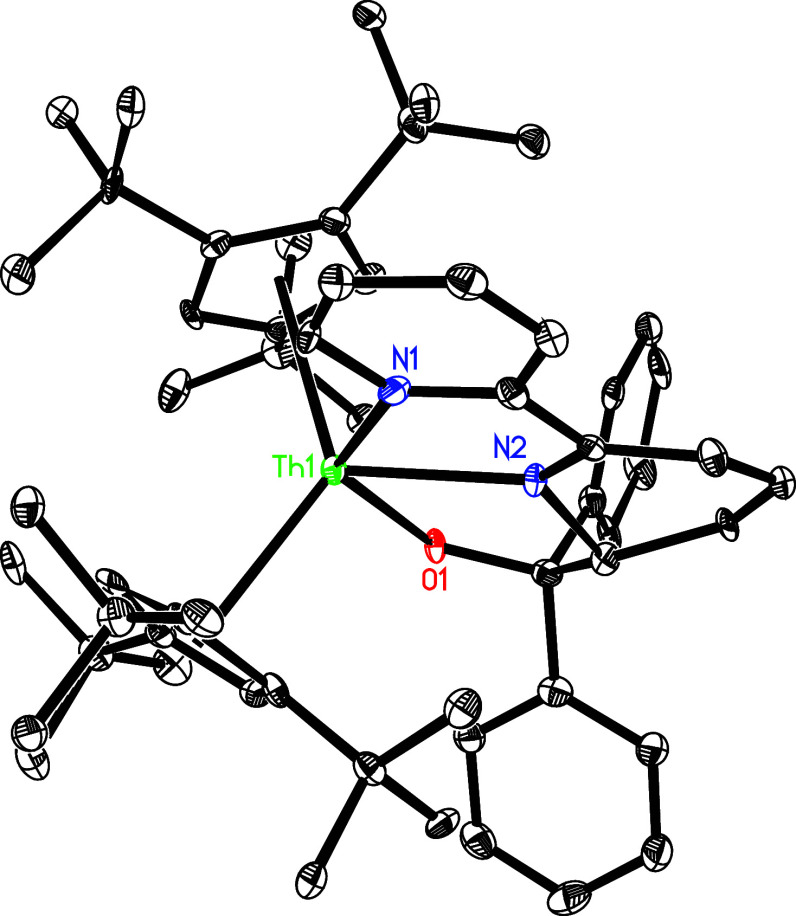
Molecular structure of **10** (thermal ellipsoids drawn at the 35% probability level).

**Figure 10:**
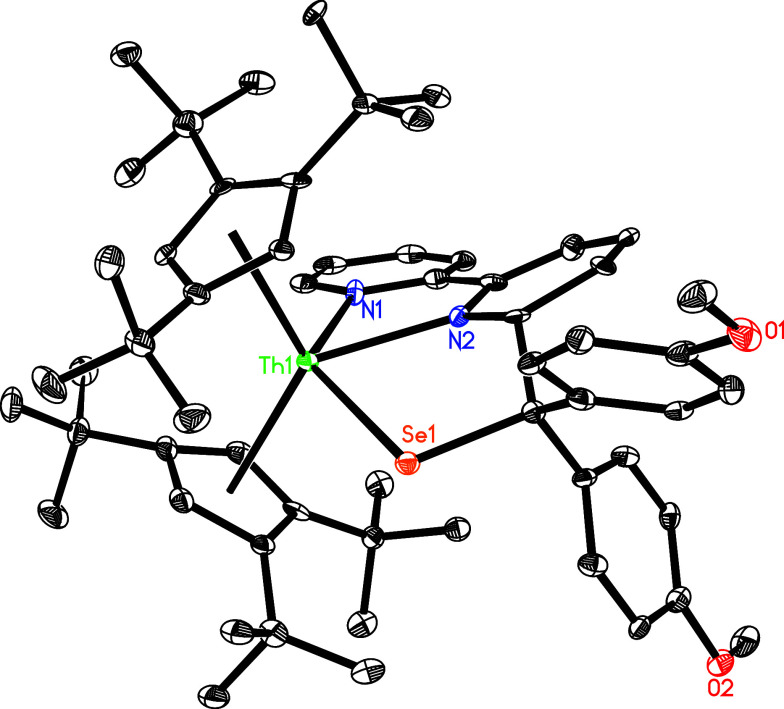
Molecular structure of **14** (thermal ellipsoids drawn at the 35% probability level).

However, contrary to the reaction with PhNCS (Scheme 4), but in accordance with [(3-Mes-C_3_H_2_N_2_)_2_BH_2_]_2_Th(bipy),^97^ (Cp^3tms^)_2_Th(bipy) (**1′**; Table 1),^148^ Cp*_2_Th(bipy), and (Cp^2*t*Bu^)_2_Th (bipy) (Table 1),^142,149^ upon exposure of complex **1** toward 1 equiv of ketone Ph_2_CO or thio-ketone Ph_2_CS, does not induce a bipy replacement, instead, the insertion products (Cp^3*t*Bu^)_2_Th[(bipy)(Ph_2_C*E*)] (*E* = O (**10**), S)^141^ are formed in quantitative conversions (Scheme 5). In agreement with the previously established reaction pattern for the reaction of (Cp^3*t*Bu^)_2_Th(bipy) (**1**) with Ph_2_CS,^141^ we propose that Ph_2_CO initially coordinates to **1** via the carbonyl oxygen-atom inducing a single-electron transfer (SET) from the bipy^2–^ ligand to Ph_2_CO forming a biradical intermediate, which then C–C couples to yield **10** (Scheme 5). Nevertheless, this is contrast to the reactivity of the uranium bipyridyl complex (Cp^3*t*Bu^)_2_U(bipy) (**1″**; Table 1),^147^ in which bipy replacement takes place to form the pinacolato (Cp^3*t*Bu^)_2_U(OCPh_2_)_2_ and disulfido (Cp^3*t*Bu^)_2_U(S_2_CPh_2_) in the presence of ketone Ph_2_CO and thio-ketone Ph_2_CS, respectively, presumably attributed to the different electron structures between the uranium bipy complex (Cp^3*t*Bu^)_2_U(bipy) (**1″**) and its thorium analogue **1** besides the more crowded environment around the U atom. In contrast, when the sterically less encumbered ketones, such as (CH_2_)_5_CO, and aldehydes, such as *p*-MePhCHO and *p*-ClPhCHO, are employed as substrates, complexes (Cp^3*t*Bu^)_2_U(bipy) (**1″**),^147^ (Cp^3tms^)_2_Th(bipy) (**1′**),^148^ Cp*_2_Th(bipy),^142^ (Cp^2*t*Bu^)_2_Th (bipy) (Table 1),^149^ and **1** exhibit identical reactivity. In the case of **1**, the insertion products (Cp^3*t*Bu^)_2_Th[(bipy)((CH_2_)_5_CO)] (**11**), (Cp^3*t*Bu^)_2_Th[(bipy)(*p*-MePhCHO)] (**12**), and (Cp^3*t*Bu^)_2_Th[(bipy)(*p*-ClPhCHO)] (**13**) are formed, respectively, in quantitative conversions (Scheme 5). The molecular structure of **10** is shown in Figure 9, for the molecular structures of **11**–**13,** see Supporting Information. The Th–N and Th–O distances are very similar in all complexes **10**–**13** with Th–N(1) ranging from 2.569(14) to 2.596(5) Å and Th–N(2) ranging from 2.389(5) to 2.427(11) Å, while the Th–O(1) distances are found in the range of 2.148(4) to 2.177(3) Å (Table 2). Also, in accordance with (Cp^3tms^)_2_Th(bipy) (**1′**; Table 1) and (Cp^2*t*Bu^)_2_Th (bipy) (Table 1),^148,149^ seleno-ketone (*p*-MeOPh)_2_CSe can insert into the bipy moiety to yield the metallocene (Cp^3*t*Bu^)_2_Th[(bipy){(*p*-MeOPh)_2_CSe}] (**14**) (Scheme 5). The molecular structure of **14** is shown in Figure 10, and selected bond distances and angles are listed in Table 2. The Th–N(1) and Th–N(2) distances of 2.590(9) and 2.441(8) Å, respectively, are in line with those found for complexes **10**–**13** (Table 2), whereas the Th–Se distance is 2.880(1) Å.

**Figure 11:**
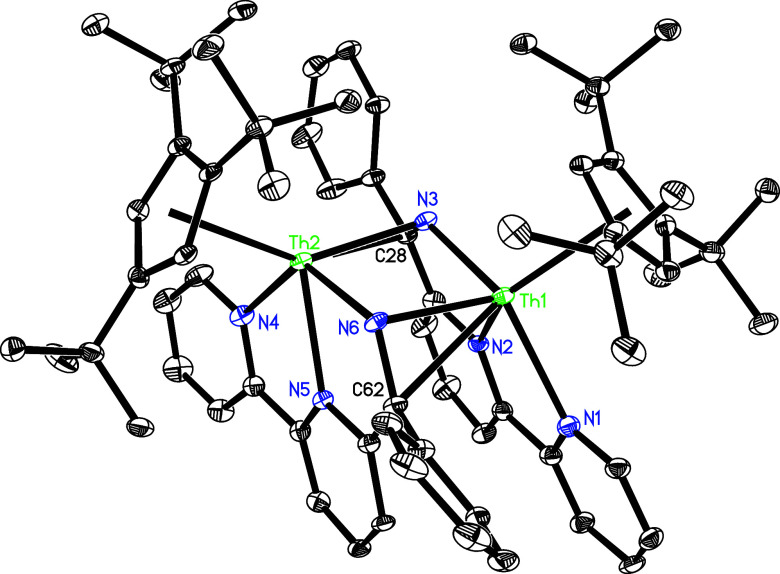
Molecular structure of **15** (thermal ellipsoids drawn at the 35% probability level).

**Scheme 6:**
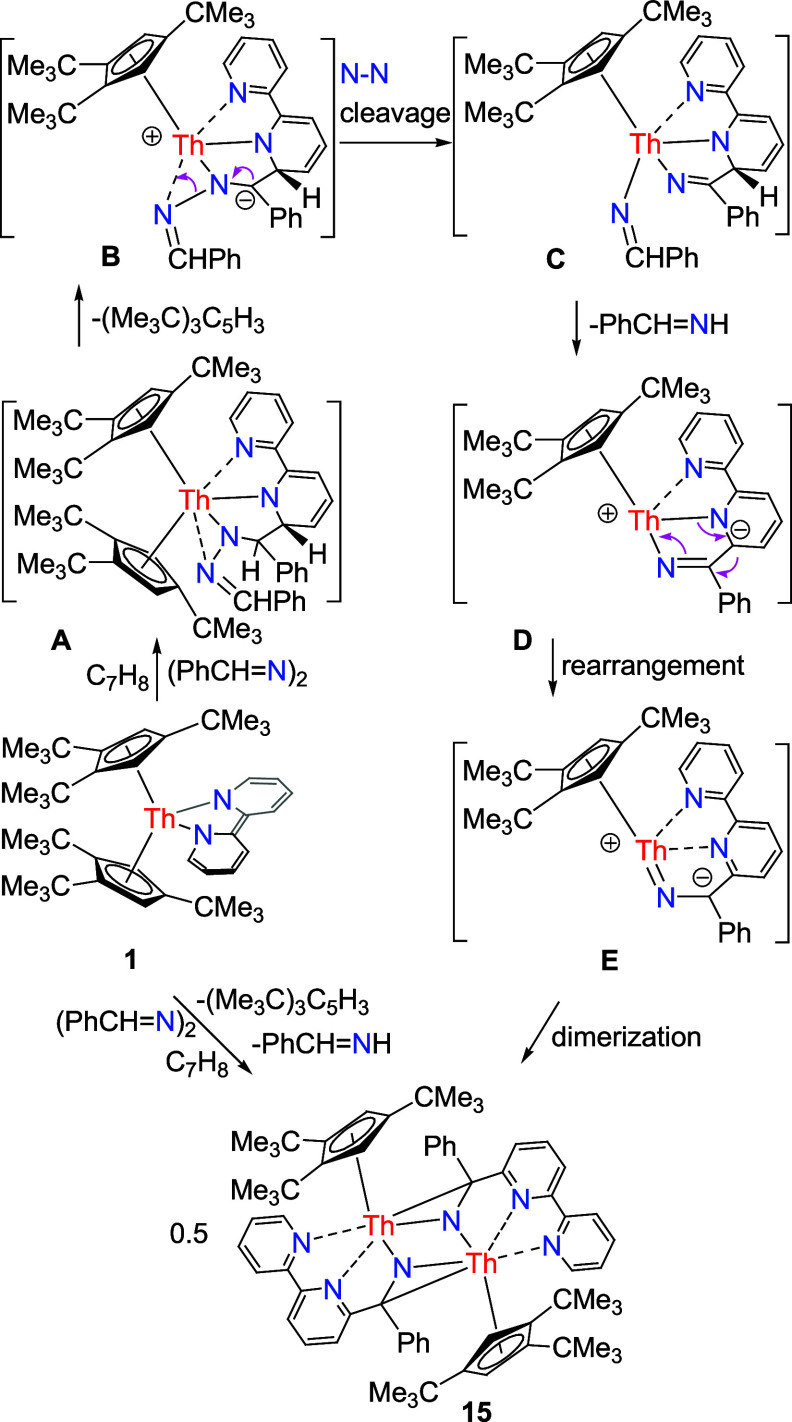
Synthesis of Compound **15**

However, contrary to the reactivity of Cp*_2_Th(bipy) and (Cp^2*t*Bu^)_2_Th(bipy) with (Ph_2_C=N)_2_ (Table 1),^142,149^ but like the reactivity of (Cp^3*t*Bu^)_2_U(bipy) (**1″**) (Table 1),^147^ complex **1** remains inert toward the ketazine (Ph_2_C=N)_2_, again as a consequence of the increased steric bulk of the 1,2,4-(Me_3_C)_3_C_5_H_2_ ligand. While the less sterically encumbered derivative (PhCH=N)_2_ inserts into the Th-bipy moiety of (Cp^2*t*Bu^)_2_Th (bipy) (Table 1) and (Cp^3tms^)_2_Th(bipy) (**1′**; Table 1),^148,149^ the dimeric imido complex [(Cp^3*t*Bu^)Th]_2_[μ-NC(Ph)(bipy)]_2_ (**15**) is isolated in good yield from **1** concomitant with the byproducts (Me_3_C)_3_C_5_H_3_ and PhCH=NH under the same conditions (Scheme 6). This change in reactivity might again be traced to the increased bulk of 1,2,4-(Me_3_C)_3_C_5_H_2_. Nevertheless, this contrasts the reactivity of the uranium bipyridyl complex (Cp^3*t*Bu^)_2_U(bipy) (**1″**) (Table 1),^147^ in which bipy replacement takes place to form the diiminato compound (Cp^3*t*Bu^)_2_U(N=CHPh)_2_ with (PhCH=N)_2_, again, presumably attributed to the different electron structures between the uranium bipy complex (Cp^3*t*Bu^)_2_U(bipy) (**1″**) and its thorium analogue **1** besides the more crowded environment around the U atom. Although no reaction intermediates could be observed spectroscopically, it is proposed that in the first step (PhCH=N)_2_ inserts into the bipy moiety of complex **1** yielding an insertion intermediate **A** (Scheme 6). To reduce the steric hindrance, intermediate **A** converts via deprotonation by one [1,2,4-(Me_3_C)_3_C_5_H_2_]^−^ ligand to give a zwitterionic intermediate **B**, followed by N–N bond cleavage to give intermediate **C**. In the next step, intermediate **C** converts via deprotonation by [PhCH=N]^−^ ligand to give a zwitterionic intermediate **D**, which rearranges to an imido zwitterionic intermediate **E**, which then dimerizes to give **15**. The molecular structure of **15** is shown in Figure 11, and selected bond distances and angles are listed in Table 2. The Th(1)–N(1), Th(1)–N(2), Th(1)–N(3) and Th(1)–N(6) distances are 2.600(4), 2.495(4), 2.243(4), and 2.321(4) Å, respectively, which are comparable to the Th(2)–N distances of 2.330(4) Å for N(3), 2.599(4) Å for N(4), 2.504(4) Å for N(5), and 2.243(4) Å for N(6). Moreover, the Th(1)–C(62) distance of 2.697(5) Å is essential identical to the Th(2)–C(28) distance of 2.702(5) Å.

### Reaction with Organic Nitriles, Acyl Nitriles, Isonitriles, and (Trimethylsilyl)diazomethane

**Scheme 7:**
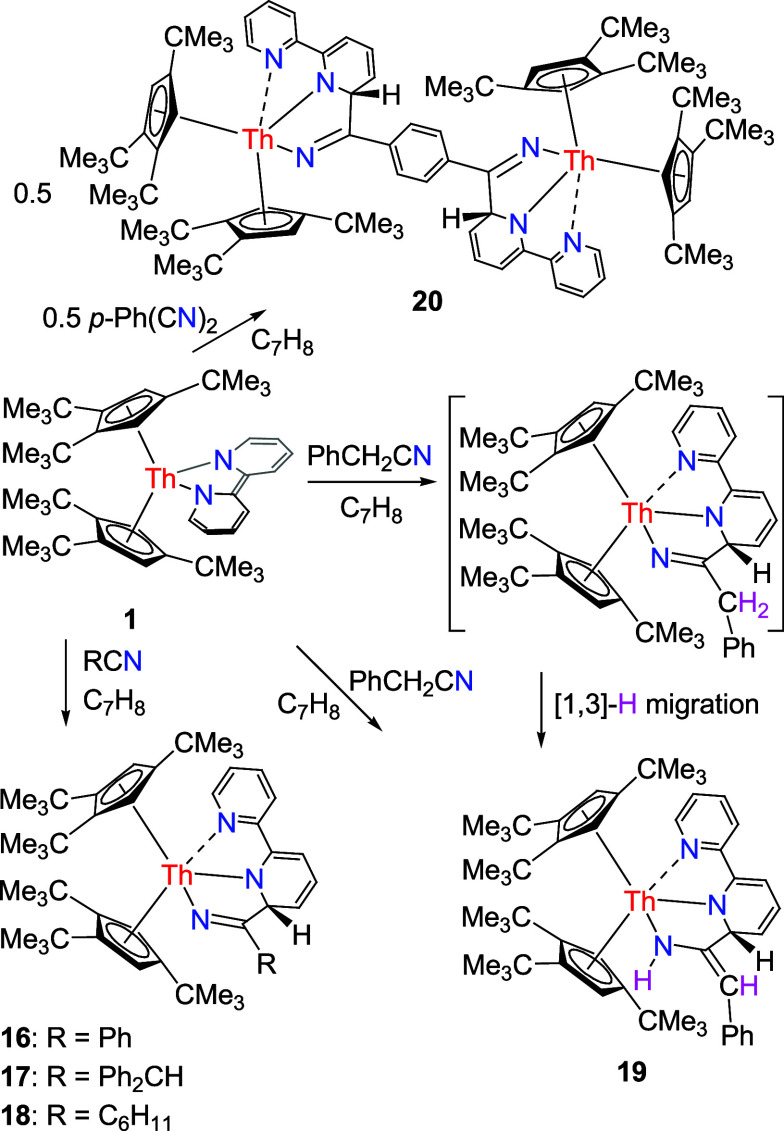
Synthesis of Compounds **16**–**20**

**Figure 12:**
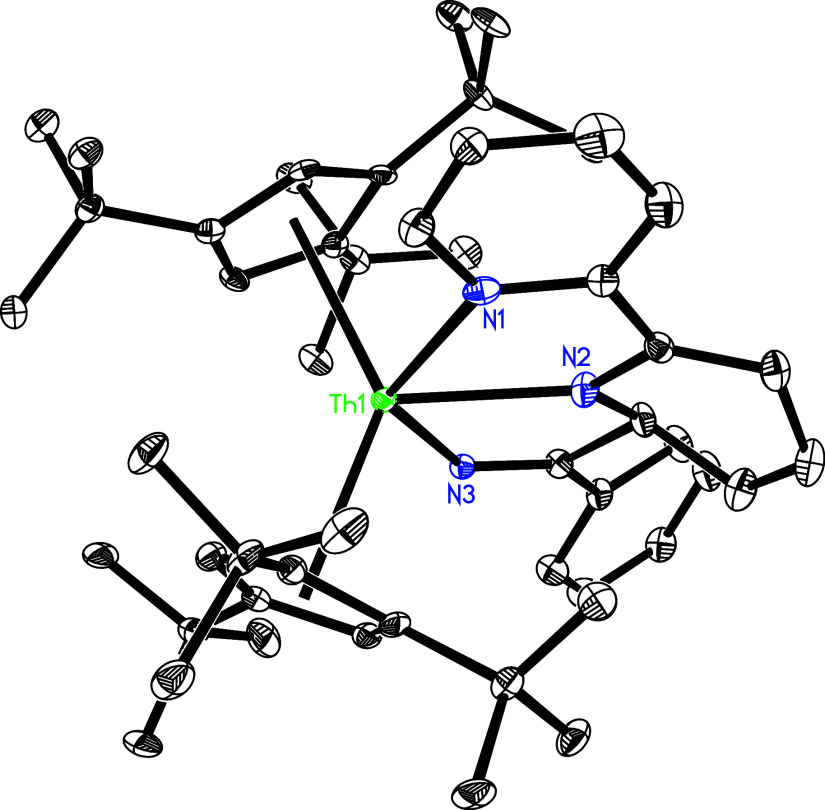
Molecular structure of **16** (thermal ellipsoids drawn at the 35% probability level).

**Figure 13:**
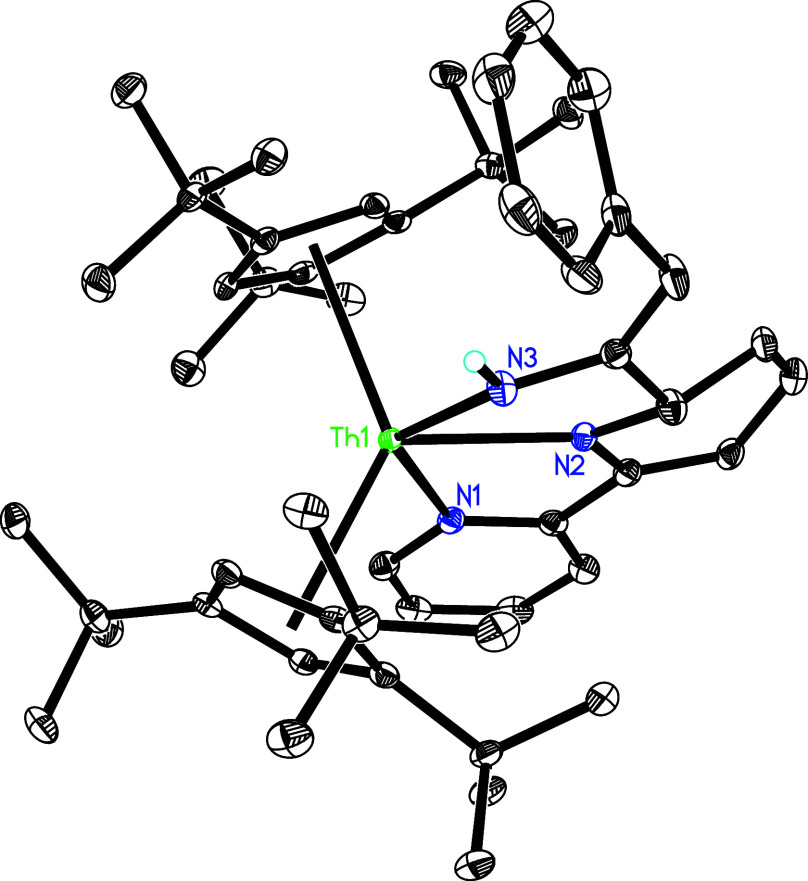
Molecular structure of **19** (thermal ellipsoids drawn at the 35% probability level). The H atom attached to N3 was located in the difference Fourier density map and refined isotropically.

**Figure 14:**
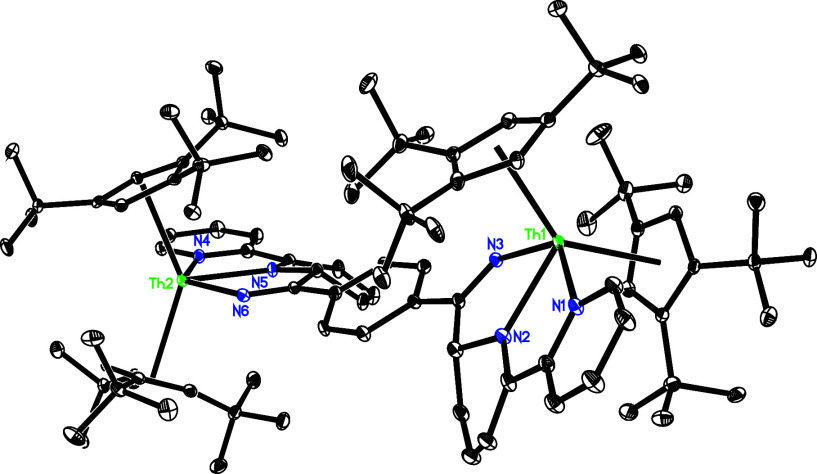
Molecular structure of **20** (thermal ellipsoids drawn at the 35% probability level).

**Figure 15:**
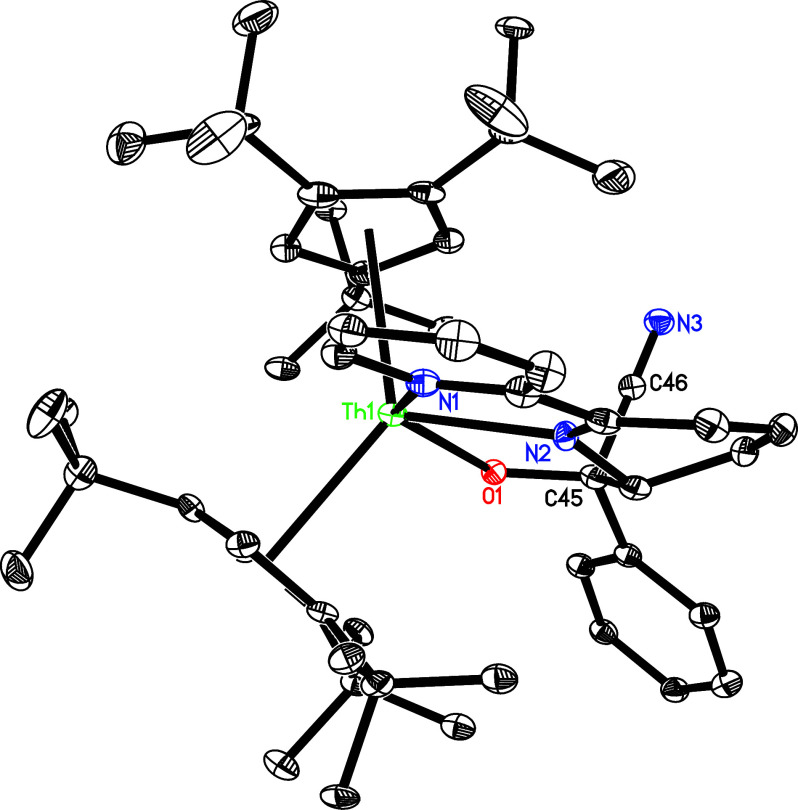
Molecular structure of **21** (thermal ellipsoids drawn at the 35% probability level).

**Scheme 8:**
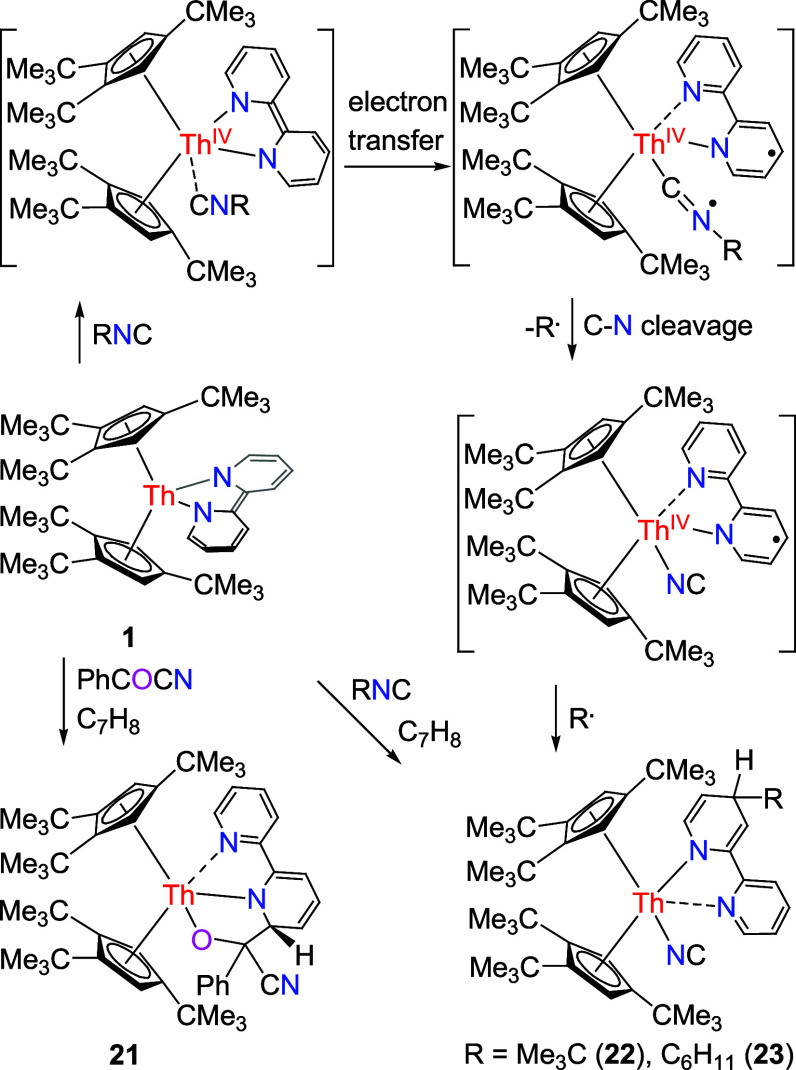
Synthesis of Compounds **21**–**23**

Furthermore, like (Cp^3tms^)_2_Th(bipy) (**1′**),^148^ Cp*_2_Th(bipy), and (Cp^2*t*Bu^)_2_Th (bipy) (Table 1),^142,149^ besides ketones, aldehydes, seleno-ketones, and ketazines, organic nitriles, such as PhCN, Ph_2_CHCN, and C_6_H_11_CN may also insert into the bipy moiety of **1** and the metallocenes (Cp^3*t*Bu^)_2_Th[(bipy)(PhCN)] (**16**), (Cp^3*t*Bu^)_2_Th[(bipy)(Ph_2_CHCN)] (**17**), and (Cp^3*t*Bu^)_2_Th[(bipy)(C_6_H_11_CN)] (**18**) are formed (Scheme 7), respectively. Nevertheless, unlike the uranium bipy complex (Cp^3tms^)_2_U(bipy),^145^ (Cp^3*t*Bu^)_2_U(bipy) (**1″**) shows no reaction with organic nitriles, such as PhCN and C_6_H_11_CN,^147^ which is attributed to the more crowded environment around the U atom.^147^ The molecular structure of **16** is shown in Figure 12, for the molecular structures of **17** and **18** see Supporting Information. The Th–N distances are very similar in all complexes **16**–**18** with Th–N(1) ranging from 2.602(5) to 2.660(3) Å, Th–N(2) ranging from 2.397(7) to 2.400(5) Å, and Th–N(3) ranging from 2.293(6) to 2.322(5) Å. These structural parameters are comparable to those found in complex **15** (Table 2). Nevertheless, benzyl nitrile PhCH_2_CN may also insert into the bipy moiety of **1** to form (Cp^3*t*Bu^)_2_Th[(bipy)(PhCH_2_CN)], which, however, converts via an [1,3]-H migration to form the bis-amido complex (Cp^3*t*Bu^)_2_Th[(bipy){C(=CHPh)NH}] (**19**) (Scheme 7). In this case divergent reactivity is observed for the less sterically encumbered derivatives (Cp^3tms^)_2_Th(bipy) (**1′**) and (Cp^2*t*Bu^)_2_Th (bipy) (Table 1).^148,149^ While insertion of PhCH_2_CN is observed for complexes **1** and (Cp^3tms^)_2_Th(bipy) (**1′**; Table 1),^148^ whereas deprotonation of PhCH_2_CN occurs for (Cp^2*t*Bu^)_2_Th (bipy) (Table 1),^149^ due to the reduced steric bulk of the 1,3-(Me_3_C)_2_C_5_H_3_ ligand. Nevertheless, the different reactivity between (Cp^3tms^)_2_Th(bipy) (**1′**) forming an insertion complex (Cp^3tms^)_2_Th[(bipy)(PhCH_2_CN)] with PhCH_2_CN (Table 1),^148^ and **1** yielding a bis-amido complex **19** may be attributed to steric effects within the insertion products [η^5^-1,2,4-(Me_3_*E*)_3_C_5_H_2_]_2_Th[(bipy)(PhCH_2_CN)] (*E* = Si,^148^ C), in which the more crowded environment at the thorium atom may facilitate the conversion of (Cp^3*t*Bu^)_2_Th[(bipy)(PhCH_2_CN)] to the corresponding bis-amido complex **19**. The molecular structure of **19** is shown in Figure 13, and selected bond distances and angles are listed in Table 2. The Th(1)–N(1) distance is 2.607(3) Å, whereas the Th(1)–N(2) and Th(1)–N(3) distances are 2.402(3) and 2.304(3) Å, respectively. Moreover, *para*-dicyanobenzene *p*-(NC)_2_Ph may also insert into the bipy moiety of **1** to form the dinuclear complex [(Cp^3*t*Bu^)_2_Th]_2_{μ-(bipy)[*p*-Ph(CN)_2_](bipy)} (**20**) in good yield (Scheme 7). The molecular structure of **20** is shown in Figure 14, and selected bond distances and angles are listed in Table 2. The Th(1)–N(1), Th(1)–N(2), and Th(1)–N(3) distances are 2.612(4), 2.394(4), and 2.282(4) Å, respectively, which are slightly shorter than the Th(2)–N distances of 2.635(4) Å for N(4), 2.388(4) Å for N(5), and 2.296(3) Å for N(6). Furthermore, benzoyl cyanide PhCOCN may also insert into the bipy moiety of **1** to form the complex (Cp^3*t*Bu^)_2_Th{(bipy)[PhC(CN)O]} (**21**) (Scheme 8), in which C=O functional group is inserted into the bipy moiety instead of the C≡N group, consistent with the more polar C=O bond. The molecular structure of **21** is shown in Figure 15, and selected bond distances and angles are listed in Table 2. The Th–N(1) and Th–N(2) distances are 2.561(7) and 2.420(6) Å, respectively, whereas the Th–O(1) distance is 2.214(4) Å. These structure parameters are close to those observed in complexes **10**–**13** (Table 2).

**Figure 16:**
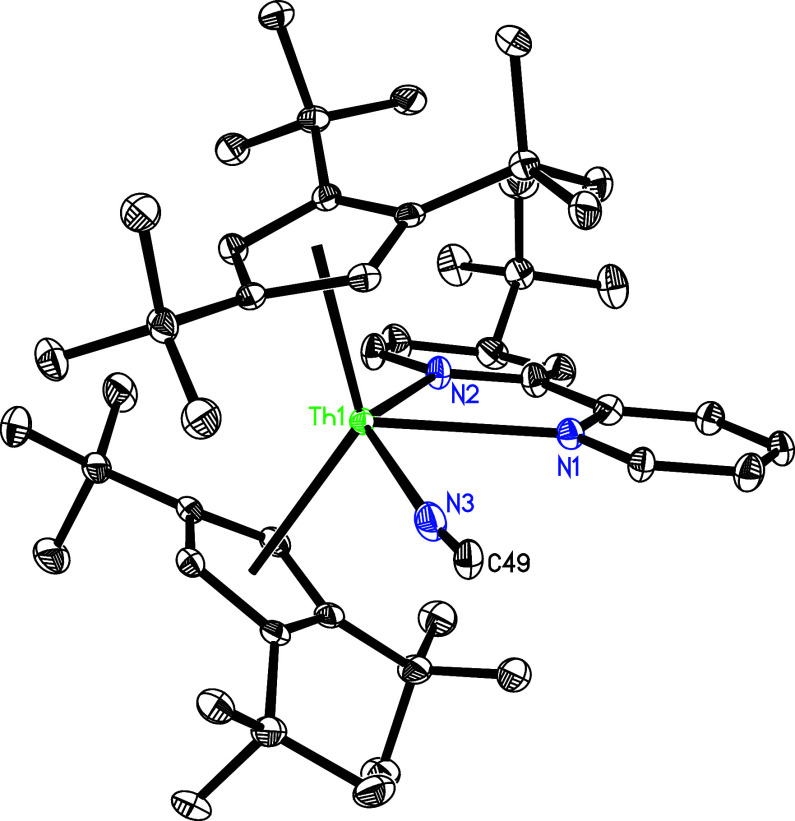
Molecular structure of **22** (thermal ellipsoids drawn at the 35% probability level).

Nevertheless, in analogy to the reactivity of [(3-Mes-C_3_H_2_N_2_)_2_BH_2_]_2_Th(bipy),^98^ (Cp^3tms^)_2_Th(bipy) (**1′**), and (Cp^2*t*Bu^)_2_Th (bipy) (Table 1),^148,149^ no insertion reaction is detected in the reaction of **1** and isonitriles Me_3_CNC and C_6_H_11_NC, instead, the thorium isocyanide amido complexes (Cp^3*t*Bu^)_2_Th[4-(Me_3_C)bipy](NC) (**22**) and (Cp^3*t*Bu^)_2_Th[4-(C_6_H_11_)bipy](NC) (**23**), respectively, are formed in quantitative conversions (Scheme 8). Nevertheless, the uranium bipy complex (Cp^3*t*Bu^)_2_U(bipy) (**1″**) shows no reaction with organic isonitriles such as C_6_H_11_NC and 2,6-Me_2_PhNC,^147^ again, attributed to the different electron structures between the uranium bipy complex (Cp^3*t*Bu^)_2_U(bipy) (**1″**) and its thorium analogue **1** besides the more crowded environment around the U atom.^149^ To account for the transformation of the complex **1** with organic isonitriles, we propose that initial coordination of RNC to **1** via the isocyanido carbon atom induces a single-electron transfer (SET) from the bipy^2–^ ligand to the π*-orbital of the N≡C group, which results in two radical anions [bipy]•^–^ and [RNC]•^–^. Nevertheless, the radical anion [RNC]•^–^ is unstable and degrades via R–NC cleavage to yield the anion CN^–^ and the radical R•. In the final step the two radicals R• and [bipy]•^–^ undergo C–C coupling to yield complexes **22**, **23** (Scheme 8), in which the C–C coupling event occurs in the *para* position for steric reasons. The molecular structure of **22** is shown in Figure 16, for the molecular structure of **23**, see Supporting Information. In complex **22**, the N(3)–C(49) distance is 1.139(7) Å (Table 2), and the angle of Th–N(3)–C(49) is nearly linear with 172.7(4)°. Moreover, analogous to the thorium isocyanide complexes [(3-Mes-C_3_H_2_N_2_)_2_BH_2_]_2_Th[4-(Me_3_C)bipy](NC),^98^ (Cp^3*t*Bu^)_2_Th(OSiMe_3_)(NC),^159^ (Cp^3tms^)_2_Th[4-(Me_3_C)bipy](NC), and (Cp^2*t*Bu^)_2_Th[4-(Me_3_C)bipy](NC),^148,149^ but in contrast to the uranium cyanide complexes (Cp^3*t*Bu^)_2_U(OSiMe_3_)(CN),^138^ (Cp^3*t*Bu^)_2_U[OC(Ph)N(*p*-tolyl)](CN),^160^ and (Cp^2*t*Bu^)_2_U[OC(Ph)N(*p*-tolyl)](CN),^161^ the CN^–^ ligand in **22** coordinates to the Th^4+^ ion by its nitrogen atom instead of the carbon atom, due to the higher Lewis acidity of the Th^4+^ ion.^148^ The experimentally observed preference for N- over C-coordination is also reflected in DFT computations, predicting the isocyanide linkage isomer to be energetically more favorable than its cyanide counterpart (Cp^3*t*Bu^)_2_Th[4-(Me_3_C)bipy](CN) (Δ*G* (298 K) = −1.7 kcal/mol) (see Supporting Information). In addition, the Th–N distances are 2.703(4) Å for N(1), 2.364(4) Å for N(2) and 2.566(5) Å for N(3), respectively, which are comparable to those observed in complex **23** with the Th–N distances of 2.711(10) Å for N(1), 2.368(9) Å for N(2), and 2.546(12) Å for N(3). Furthermore, in complex **23**, the N(3)–C(51) distance is 1.168(15) Å, and the angle of Th–N(3)-C(51) is 169.3(10)°. However, when aromatic isonitrile 2,6-Me_2_PhNC is used as substrate, no reaction is observed even when the reaction mixture is heated at 100 °C for 1 week, presumably due to the stronger C_phenyl_–NC bond. Moreover, while complexes (Cp^3tms^)_2_Th(bipy) (**1′**; Table 1), (Cp^2*t*Bu^)_2_Th (bipy) (Table 1) and **1** show a similar reactivity in the activation of isonitriles,^148,149^ the reaction occurs at ambient temperature for complex (Cp^3tms^)_2_Th(bipy) (**1′**), whereas complexes (Cp^2*t*Bu^)_2_Th(bipy) and **1** require elevated temperatures of 50 and 120 °C, respectively. This difference may again be traced to the different steric and electronic effects exerted by the Cp ligands, in which the more steric and more electron rich 1,2,4-(Me_3_C)_3_C_5_H_2_ ligand may not facilitate the formation and conversion of the isonitrile adducts (Cp^3*t*Bu^)_2_Th(bipy)(CNR) to the corresponding isocyanide amido complexes.

Furthermore, the reaction of the uranium bipy complex (Cp^3*t*Bu^)_2_U(bipy) (**1″**) with (trimethylsilyl)diazomethane Me_3_SiCHN_2_ results in the formation of the imido complex (Cp^3*t*Bu^)_2_U(=NNCHSiMe_3_) (Table 1),^147^ but (Cp^3tms^)_2_Th(bipy) (**1′**) forms a bis-amido complex (Cp^3tms^)_2_Th[5,6-(Me_3_SiCH)bipy] (Table 1),^148^ presumably attributed to the different electron structures between the uranium bipy complex (Cp^3*t*Bu^)_2_U(bipy) (**1″**) and its thorium analogue (Cp^3tms^)_2_Th(bipy) (**1′**) besides the larger Lewis acidity of the Th^4+^ ion. Nevertheless, like (Cp^2*t*Bu^)_2_Th (bipy) (Table 1),^149^ no reaction occurs for (Cp^3*t*Bu^)_2_Th(bipy) (**1**) in the presence of Me_3_SiCHN_2_ even when the reaction mixture is heated at 100 °C for 1 week. This different reactivity between (Cp^3tms^)_2_Th(bipy) (**1′**), (Cp^2*t*Bu^)_2_Th(bipy), and (Cp^3*t*Bu^)_2_Th(bipy) (**1**) is presumably attributed to the more electron deficient 1,2,4-(Me_3_Si)_3_C_5_H_2_ ligand, which may increase the Lewis acidity of the Th^4+^ ion, and therefore facilitates the formation and conversion of the Me_3_SiCHN_2_ adduct (Cp^3tms^)_2_Th(bipy)(N_2_CHSiMe_3_) to the bis-amido complex (Cp^3tms^)_2_Th[5,6-(Me_3_SiCH)bipy].^148^

## Conclusions

The thorium bipyridyl metallocene (Cp^3*t*Bu^)_2_Th(bipy) (**1**) reacts with a broad range of small molecules and the resulting products were compared to those obtained with less sterically encumbered thorium derivatives and its uranium counterpart (Cp^3*t*Bu^)_2_U(bipy) (**1″**). Like the other thorium and uranium bipy metallocenes (Table 1),^138−149^ complex **1** may act as a masked synthon for the divalent thorium fragment (Cp^3*t*Bu^)_2_Th(II) in the presence of CuI, hydrazine derivatives, Ph_2_*E*_2_ (*E* = S, Se), elemental sulfur (S_8_) and selenium (Se), organic azides, CS_2_ and isothiocyanates. However, in contrast to its uranium analogue the electron required for these two-electron processes exclusively originates from the dianionic [bipy]^2–^ ligand, whereas for the related uranium derivatives the electrons are provided jointly from the U(III) atom and the bipyridyl radical anion [bipy]•^–^. Moreover, complex **1** may induce C–C σ-bond formation when mixed with substrates featuring polar C=O, C=S, C=Se, C=N, and C≡N functionalities, such as aldehydes, ketones, acyl nitriles, thio-ketone, seleno-ketones, ketazines, and organic nitriles. Furthermore, organic isonitriles undergo C–N bond cleavage and C–C coupling processes when exposed to complex **1**. Also, a single electron-transfer (SET) process may occur when 1 equiv of CuI is added to complex **1**.

Besides several similarities, differences in the reactivity of the related uranium and thorium compounds are also noted. For example, while the reaction of the uranium bipy complex (Cp^3*t*Bu^)_2_U(bipy) (**1″**) with CuI exclusively forms the U(III) iodide (Cp^3*t*Bu^)_2_UI (Table 1),^147^ complex **1** gives the Th(IV) diiodide complex (Cp^3*t*Bu^)_2_ThI_2_ (**2**) and Th(IV) bipyridyl iodide complex (Cp^3*t*Bu^)_2_Th(I)(bipy) (**3**), in the presence of 2 or 1 equiv of CuI, respectively. Treatment of the uranium bipy complex (Cp^3*t*Bu^)_2_U(bipy) (**1″**) with Ph_2_S_2_ or Ph_2_Se_2_ affords the disulfido or diselenido complex (Cp^3*t*Bu^)_2_U(*E*Ph)_2_ (*E* = S, Se) (Table 1),^147^ whereas complex **1** degrades to the trisulfido or triselenido complexes (Cp^3*t*Bu^)Th(*E*Ph)_3_(bipy) (*E* = S (**5**), Se).^139^ Moreover, while the bipy ligand is replaced on addition of Ph_2_CO or Ph_2_CS to the uranium bipy complex (Cp^3*t*Bu^)_2_U(bipy) (**1″**) to give the U(IV) pinacolato complex (Cp^3*t*Bu^)_2_U(OCPh_2_)_2_ and disulfido complex (Cp^3*t*Bu^)_2_U(S_2_CPh_2_) (Table 1),^147^ respectively, insertion of Ph_2_C*E* is observed for its thorium counterpart **1** to yield (Cp^3*t*Bu^)_2_Th[(bipy)(Ph_2_C*E*)] (*E* = O (**10**), S).^140^ Bipy ligand replacement also takes place for the uranium bipy complex (Cp^3*t*Bu^)_2_U(bipy) (**1″**) with ketazine (PhCH=N)_2_ to form the U(IV) diiminato complex (Cp^3*t*Bu^)_2_U(N=CHPh)_2_ (Table 1),^147^ whereas complex **1** degrades to the dimeric imido complex [(Cp^3*t*Bu^)Th]_2_[μ-NC(Ph)(bipy)]_2_ (**15**). In addition, while the uranium bipy complex (Cp^3*t*Bu^)_2_U(bipy) (**1″**) shows no reactivity toward organic nitriles such as PhCN and C_6_H_11_CN,^147^ insertion of PhCN and C_6_H_11_CN into the bipy moiety of complex **1** yields the products (Cp^3*t*Bu^)_2_Th[(bipy)(PhCN)] (**16**) and (Cp^3*t*Bu^)_2_Th[(bipy)(C_6_H_11_CN)] (**18**), respectively. Reaction of complex **1** with isonitriles Me_3_CNC and C_6_H_11_NC gives the thorium isocyanide amido complexes (Cp^3*t*Bu^)_2_Th[4-(Me_3_C)bipy](NC) (**22**) and (Cp^3*t*Bu^)_2_Th[4-(C_6_H_11_)bipy](NC) (**23**), respectively, whereas the uranium bipy complex (Cp^3*t*Bu^)_2_U(bipy) (**1″**) remains unaffected.^147^ Furthermore, the reaction of the uranium bipy complex (Cp^3*t*Bu^)_2_U(bipy) (**1″**) with (trimethylsilyl)diazomethane Me_3_SiCHN_2_ gives an imido complex (Cp^3*t*Bu^)_2_U(=NNCHSiMe_3_) (Table 1),^147^ whereas complex **1** remains unperturbed. In most cases these differences in the reactivity between the thorium complex **1** and its uranium analogue (Cp^3*t*Bu^)_2_U(bipy) (**1″**) can be attributed to the different electronic structures between the (Cp^3*t*Bu^)_2_An and [bipy] fragments and the steric effects around the metal atom.

This study also illustrates that relatively minor variations in the auxiliary cyclopentadienyl ligand impact the reactivity of these thorium bipyridyl metallocenes. For example, reaction of complex **1** with 1 equiv of CuI gives the Th(VI) bipyridyl iodide complex (Cp^3*t*Bu^)_2_Th(I)(bipy) (**3**), whereas (Cp^3tms^)_2_Th(bipy) (**1′**) exclusively affords a Th(IV) diiodide compound (Cp^3tms^)_2_ThI_2_ (Table 1) regardless of the amount of the added CuI.^148^ While the reaction of (Cp^3tms^)_2_Th(bipy) (**1′**) with Ph_2_S_2_ yields the disulfido complex (Cp^3tms^)_2_Th(SPh)_2_ (Table 1),^148^ complex **1** degrades to a thorium trisulfido complex (Cp^3*t*Bu^)Th(SPh)_3_(bipy) (**5**). Moreover, exposure of complex **1** to Me_3_SiN_3_ gives an imido complex (Cp^3*t*Bu^)_2_Th=NSiMe_3_,^139^ whereas the closely related derivative (Cp^3tms^)_2_Th(bipy) (**1′**) forms a bis-amido complex (Cp^3tms^)_2_Th(NHSiMe_3_)_2_ (Table 1).^148^ While the reaction of (Cp^3tms^)_2_Th(bipy) (**1′**) with CS_2_ gives a side-on coordinate ethylenetetrathiolate complex [(Cp^3tms^)_2_Th]_2_(μ-C_2_S_4_) (Table 1),^148^ complex **1** yields a terminally coordinate ethylenetetrathiolate compound [(Cp^3*t*Bu^)_2_Th]_2_(μ-S_2_C=CS_2_) (**8**). In addition, treatment of (Cp^3tms^)_2_Th(bipy) (**1′**) with ketazine (PhCH=N)_2_ gives an insertion product (Cp^3tms^)_2_Th[(bipy)(PhCHNN=CHPh)] (Table 1),^148^ whereas complex **1** degrades to a dimeric imido complex [(Cp^3*t*Bu^)_2_Th]_2_[μ-NC(Ph)(bipy)]_2_ (**15**). While reaction of (Cp^3tms^)_2_Th(bipy) (**1′**) with PhCH_2_CN gives an insertion product (Cp^3tms^)_2_Th[(bipy)(PhCH_2_CN)] (Table 1),^148^ complex **1** yields a bis-amido complex (Cp^3*t*Bu^)_2_Th[(bipy){C(=CHPh)NH}] (**19**). Whereas, reaction of (Cp^3tms^)_2_Th(bipy) (**1′**) with isonitriles occurs at ambient temperature,^148^ while the same reaction for complex **1** requires heating to 120 °C to proceed. Furthermore, while (Cp^3tms^)_2_Th(bipy) (**1′**) readily reacts with (trimethylsilyl)diazomethane Me_3_SiCHN_2_ to yield a bis-amido complex (Cp^3tms^)_2_Th[5,6-(Me_3_SiCH)bipy] (Table 1),^148^ complex **1** remains unaffected. In summary, although the dominant reaction patterns of thorium bipy metallocenes are very similar, minor changes in the steric and electronic properties of the supporting Cp ligand effect the individual reactivity. These changes are predominantly for steric reasons, but electronic properties such as the electronic richness of the Cp ligand may also play a role. To further understand these effects, we continue with our investigations on actinide complexes bearing redox noninnocent ligands.

## Experimental Section

### General Procedures

All reactions and product manipulations were carried out under an atmosphere of dry dinitrogen with rigid exclusion of air and moisture using standard Schlenk or cannula techniques, or in a glovebox. All organic solvents were freshly distilled from sodium benzophenone ketyl immediately prior to use. (Cp^3*t*Bu^)_2_Th(bipy) (**1**),^139^ [(Cp^3*t*Bu^)_2_Th]_2_(μ-S)_2_,^159^ and (*p*-MeOPh)_2_CSe^145^ were prepared according to literature methods. All other chemicals were purchased from Aldrich Chemical Co. and Beijing Chemical Co. and used as received unless otherwise noted. Infrared spectra were recorded in KBr pellets on an Avatar 360 Fourier transform spectrometer. ^1^H and ^13^C{^1^H} NMR spectra were recorded on a JEOL AV 400 at 400 and 100 MHz, respectively, or recorded on a JEOL 600 spectrometer at 600 and 150 MHz, respectively. ^77^Se{^1^H} NMR spectra were recorded on a JEOL 600 spectrometer at 114 MHz. All chemical shifts are reported in δ units with reference to the residual protons of the deuterated solvents, which served as internal standards, for proton and carbon chemical shifts, and to external Me_2_Se (0.00 ppm) for selenium chemical shifts. Electronic absorption spectroscopic measurements were recorded at 293 K in toluene in 1 cm quartz cuvettes on a UV-3600i Plus spectrometer. Melting points were obtained on an X-6 melting point apparatus and were uncorrected. Elemental analyses were performed on a Vario EL elemental analyzer.

***Caution!*** Natural thorium (primary isotope ^232^Th) is a weak α-emitter (4.012 MeV) with a half-life of 1.41 × 10^1^^0^ years. Therefore, manipulations and reactions should be carried out in monitored fume hoods or in an inert-atmosphere drybox in a laboratory equipped with α- and β-counting equipment.

### Preparation of (Cp^3*t*Bu^)_2_ThI_2_ (**2**)

#### Method A

Solid CuI (96 mg, 0.50 mmol) was added to a toluene (15 mL) solution of (Cp^3*t*Bu^)_2_Th(bipy) (**1**; 214 mg, 0.25 mmol) with stirring at room temperature. After the solution was stirred at room temperature overnight, the solvent was removed. The residue was extracted with *n*-hexane (10 mL × 3) and filtered. The volume of the filtrate was reduced to 3 mL, colorless crystals of **2** were formed when this solution was kept at −20 °C overnight. Crystals of **2** were isolated by filtration, quickly washed with cold *n*-hexane (2 mL), and dried at room temperature under vacuum overnight. Yield: 219 mg (92%). M.p.: 145–147 °C (dec.). ^1^H NMR (C_6_D_6_): δ 6.78 (s, 4H, ring *H*), 1.64 (s, 36H, C(C*H*_3_)_3_), 1.27 (s, 18H, C(C*H*_3_)_3_) ppm. ^13^C{^1^H} NMR (C_6_D_6_): δ 150.3 (ring *C*), 148.3 (ring *C*), 119.6 (ring *C*), 35.8 ((CH_3_)_3_*C*), 34.7 ((CH_3_)_3_*C*), 32.7 ((CH_3_)_3_*C*), 32.3 ((CH_3_)_3_*C*) ppm. IR (KBr, cm^–1^): *v* 2959 (s), 1457 (s), 1389 (s), 1361 (s), 1237 (s), 1163 (s), 1104 (s), 1020 (s), 839 (s). Anal. calcd. for C_34_H_58_I_2_Th: C, 42.87; H, 6.14. Found: C, 42.88; H, 6.15.

#### Method B

##### NMR Scale

CuI (7.6 mg, 0.04 mmol) was slowly added to a J. Young NMR tube charged with (Cp^3*t*Bu^)_2_Th(bipy) (**1**; 17.1 mg, 0.02 mmol), and C_6_D_6_ (0.5 mL). Resonances of **2** and those of 2,2′-bipyridine were observed by ^1^H NMR spectroscopy (100% conversion) when this solution was kept at room temperature overnight.

### Preparation of (Cp^3*t*Bu^)_2_Th(I)(bipy) (**3**)

#### Method A

Solid CuI (48 mg, 0.25 mmol) was added to a toluene (15 mL) solution of (Cp^3*t*Bu^)_2_Th(bipy) (**1**; 214 mg, 0.25 mmol) with stirring at room temperature. After the solution was stirred at room temperature overnight, the solvent was removed. The residue was extracted with benzene (10 mL × 3) and filtered. The volume of the filtrate was reduced to 3 mL, brown crystals of **3** formed when this solution was kept at 10 °C for 2 days. Crystals of **3** were isolated by decantation of the supernatant, rapidly washed with cold *n*-hexane (2 mL), and dried at room temperature under vacuum overnight. Yield: 233 mg (95%). M.p.: 180–182 °C (dec.). ^1^H NMR (C_6_D_6_): δ 1.69 (s, 36H, C(C*H*_3_)_3_), 1.33 (s, 18H, C(C*H*_3_)_3_) ppm; other protons were not observed. ^13^C{^1^H} NMR (C_6_D_6_): δ 34.3 ((CH_3_)_3_*C*) ppm; other carbons were not observed. UV/vis (toluene): λ_max_/nm 382 (ε/M^–1^ cm^–1^ 4.00 × 10^3^), 457 (1.36 × 10^3^), 871 (7.41 × 10^2^), 966 (6.18 × 10^2^), 1089 (3.01 × 10^2^), 1252 (1.23 × 10^2^). IR (KBr, cm^–1^): *v* 2947 (s), 1589 (m), 1473 (s), 1373 (s), 1234 (s), 1018 (s), 833 (s). Anal. calcd. for C_44_H_66_IN_2_Th: C, 53.82; H, 6.77; N, 2.85. Found: C, 53.85; H, 6.75; N, 2.83.

#### Method B

##### NMR Scale

CuI (3.8 mg, 0.02 mmol) was slowly added to a J. Young NMR tube charged with (Cp^3*t*Bu^)_2_Th(bipy) (**1**; 17.1 mg, 0.02 mmol) and C_6_D_6_ (0.5 mL). Resonances of **3** were observed by ^1^H NMR spectroscopy (100% conversion) when this solution was kept at room temperature overnight.

#### Reaction of (Cp^3*t*Bu^)_2_Th(bipy) (**1**) with CuI

##### NMR Scale

CuI (3.8 mg, 0.02 mmol) was slowly added to a J. Young NMR tube charged with (Cp^3*t*Bu^)_2_Th(bipy) (**1**; 17.1 mg, 0.02 mmol) and C_6_D_6_ (0.5 mL). Resonances of **3** were observed by ^1^H NMR spectroscopy (100% conversion) when this solution was kept at room temperature overnight. CuI (3.8 mg, 0.02 mmol) was slowly added to this mixture, resonances of **2** along with those of 2,2′-bipyridine were observed by ^1^H NMR spectroscopy when this solution was kept at room temperature overnight.

### Preparation of (Cp^3*t*Bu^)_2_Th(NHPh)_2_ (**4**)

#### Method A

A toluene solution (5 mL) of (PhNH)_2_ (46 mg, 0.25 mmol) was added to a toluene (10 mL) solution of (Cp^3*t*Bu^)_2_Th(bipy) (**1**; 214 mg, 0.25 mmol) with stirring at room temperature. After the solution was stirred at 120 °C for 3 days, the solvent was removed. The residue was extracted with benzene (10 mL × 3) and filtered. The volume of the filtrate was reduced to 3 mL, colorless crystals of **4** formed when this solution was kept at 10 °C for 2 days. Crystals of **4** were isolated by decantation of the supernatant, rapidly washed with cold *n*-hexane (2 mL), and dried at room temperature under vacuum overnight. Yield: 190 mg (86%). M.p.: 177–179 °C (dec.). ^1^H NMR (C_6_D_6_): δ 7.25 (t, *J* = 7.6 Hz, 4H, phenyl), 6.89 (d, *J* = 8.2 Hz, 4H, phenyl), 6.74 (t, *J* = 7.3 Hz, 2H, phenyl), 6.56 (s, 4H, ring C*H*), 5.08 (s, 2H, N*H*), 1.393 (s, 18H, C(C*H*_3_)_3_), 1.385 (s, 36H, C(C*H*_3_)_3_) ppm. ^13^C{^1^H} NMR (C_6_D_6_): δ 156.9 (phenyl *C*), 145.3 (phenyl *C*), 144.4 (phenyl *C*), 129.2 (phenyl *C*), 119.3 (ring *C*), 118.3 (ring *C*), 115.9 (ring *C*), 35.1 ((CH_3_)_3_*C*), 34.2 ((*C*H_3_)_3_C), 34.1 ((CH_3_)_3_*C*), 32.9 ((*C*H_3_)_3_C) ppm. IR (KBr, cm^–1^): *v* 2954 (s), 1589 (s), 1481 (s), 1365 (s), 1257 (s), 1010 (m), 825 (s). Anal. calcd. for C_46_H_70_N_2_Th: C, 62.56; H, 7.99; N, 3.17. Found: C, 62.59; H, 7.96; N, 3.15.

#### Method B

##### NMR Scale

A C_6_D_6_ (0.3 mL) solution of (PhNH)_2_ (3.7 mg, 0.02 mmol) was slowly added to a J. Young NMR tube charged with (Cp^3*t*Bu^)_2_Th(bipy) (**1**; 17.1 mg, 0.02 mmol) and C_6_D_6_ (0.2 mL). Resonances of **4** and those of 2,2′-bipyridine were observed by ^1^H NMR spectroscopy (100% conversion) when this solution was kept at 120 °C for 3 days.

### Preparation of (Cp^3*t*Bu^)Th(SPh)_3_(bipy)·0.5C_6_H_6_ (**5**·0.5C_6_H_6_)

This compound was prepared as yellow crystals from the reaction of (Cp^3*t*Bu^)_2_Th(bipy) (**1**; 214 mg, 0.25 mmol) and Ph_2_S_2_ (54 mg, 0.25 mmol) in toluene (15 mL) at 50 °C and recrystallization from a benzene solution by a similar procedure as that in the synthesis of **4**. The product was isolated by filtration, quickly washed with cold *n*-hexane (2 mL), and dried at room temperature under vacuum overnight. Yield: 67 mg (26%; based on Th). M.p.: 122–124 °C (dec.). ^1^H NMR (C_6_D_6_): δ 9.43 (d, *J* = 3.4 Hz, 2H, bipy), 7.15 (s, 6H, phenyl), 7.05 (d, *J* = 7.2 Hz, 6H, phenyl), 6.82 (s, 2H, ring C*H*), 6.67 (t, *J* = 7.8 Hz, 2H, bipy), 6.61 (t, *J* = 7.4 Hz, 6H, phenyl), 6.56 (t, *J* = 7.0 Hz, 3H, phenyl), 6.46 (d, *J* = 8.1 Hz, 2H, bipy), 6.37 (t, *J* = 6.3 Hz, 2H, bipy), 1.93 (s, 18H, C(C*H*_3_)_3_), 1.79 (s, 9H, C(C*H*_3_)_3_) ppm. ^13^C{^1^H} NMR (C_6_D_6_): δ 153.3 (bipy *C*), 150.6 (bipy *C*), 148.4 (bipy *C*), 147.0 (bipy *C*), 146.9 (bipy *C*), 137.9 (phenyl *C*), 133.2 (phenyl *C*), 128.5 (phenyl *C*), 127.5 (phenyl *C*), 124.4 (phenyl *C*), 122.9 (ring *C*), 121.8 (ring *C*), 119.8 (ring *C*), 36.0 ((CH_3_)_3_*C*), 35.3 ((CH_3_)_3_*C*), 34.6 ((*C*H_3_)_3_C), 32.3 ((*C*H_3_)_3_C) ppm. IR (KBr, cm^–1^): *v* 2959 (s), 1384 (s), 1260 (s), 1088 (s), 1014 (s), 804 (s). Anal. calcd. for C_51_H_58_N_2_S_3_Th: C, 59.63; H, 5.69; N, 2.73. Found: C, 59.65; H, 5.66; N, 2.75. After isolation of the yellow crystals of **5**, the solvent of the mother liquid was removed. ^1^H NMR spectroscopy showed the presence of the resonances of **5** along with those of 2,2′-bipyridine and other unidentified compounds in the residue.

### Preparation of (Cp^3*t*Bu^)_2_ThS_2_(dmap) (**6**)

#### Method A

This compound was prepared as yellow crystals from the reaction of (Cp^3*t*Bu^)_2_Th(bipy) (**1**; 214 mg, 0.25 mmol), S_8_ (16 mg, 0.0625 mmol), and dmap (31 mg, 0.25 mmol) in toluene (15 mL) at room temperature and recrystallized from a benzene solution by a similar procedure as that in the synthesis of **4**. The product was isolated by filtration, quickly washed with cold *n*-hexane (2 mL), and dried at room temperature under vacuum overnight. Yield: 188 mg (85%). M.p.: 145–147 °C (dec.). ^1^H NMR (C_6_D_6_): δ 8.47 (s, 2H, dmap), 6.53 (s, 4H, ring C*H*), 6.10 (d, *J* = 4.1 Hz, 2H, dmap), 2.21 (s, 6H, N(C*H*_3_)_2_), 1.49 (s, 36H, C(C*H*_3_)_3_), 1.31 (s, 18H, C(C*H*_3_)_3_) ppm. ^13^C{^1^H} NMR (C_6_D_6_): δ 156.7 (py *C*), 150.6 (py *C*), 144.1 (ring *C*), 143.4 (ring *C*), 118.0 (ring *C*), 106.8 (py *C*), 38.3 (N*C*H_3_), 35.1 ((CH_3_)_3_*C*), 34.6 ((*C*H_3_)_3_C), 33.9 ((CH_3_)_3_*C*), 32.7 ((*C*H_3_)_3_C) ppm. IR (KBr, cm^–1^): *v* 2963 (s), 1616 (s), 1455 (s), 1360 (s), 1232 (s), 952 (s), 804 (s), 758 (s). Anal. Calcd for C_41_H_68_N_2_S_2_Th: C, 55.63; H, 7.74; N, 3.16. Found: C, 55.66; H, 7.72; N, 3.18.

#### Method B

##### NMR Scale

Solid S_8_ (1.3 mg, 0.0051 mmol) was slowly added to a J. Young NMR tube charged with (Cp^3*t*Bu^)_2_Th(bipy) (**1**; 17.1 mg, 0.02 mmol), dmap (2.5 mg, 0.02 mmol) and C_6_D_6_ (0.2 mL). Resonances of **6** and those of 2,2′-bipyridine were observed by ^1^H NMR spectroscopy (100% conversion) when this solution was kept at room temperature for 1 week.

### Preparation of [(Cp^3*t*Bu^)Th]_4_(μ-Se_11_) (**7**)

This compound was prepared as orange crystals from the reaction of (Cp^3*t*Bu^)_2_Th(bipy) (**1**; 214 mg, 0.25 mmol) and Se (79 mg, 1.0 mmol) in toluene (15 mL) at 80 °C and recrystallization from a benzene solution by a similar procedure as that in the synthesis of **4**. The product was isolated by decantation of the supernatant, rapidly washed with cold *n*-hexane (2 mL), and dried at room temperature under vacuum overnight. Yield: 106 mg (62%; base on Th). M.p. > 300 °C (dec.). ^1^H NMR (C_6_D_6_): δ 6.62 (s, 2H, ring C*H*), 1.68 (s, 18H, C(C*H*_3_)_3_), 1.43 (s, 9H, C(C*H*_3_)_3_) ppm. ^13^C{^1^H} NMR (C_6_D_6_): δ 148.5 (ring *C*), 147.2 (ring *C*), 119.2 (ring *C*), 35.2 ((CH_3_)_3_*C*), 35.0 ((*C*H_3_)_3_C), 33.8 ((CH_3_)_3_*C*), 33.1 ((*C*H_3_)_3_C) ppm. ^77^Se{^1^H} NMR (C_6_D_6_): δ 1042.9 (s, 1Se, (Se)_2_*Se*), 242.4 (s, 8Se, *Se*_2_), 237.3 (s, 2Se, (*Se*)_2_Se) ppm. IR (KBr, cm^–1^): *v* 2957 (s), 1384 (s), 1261 (m), 1163 (m), 1102 (m), 1019 (m), 826 (s). Anal. calcd. for C_68_H_116_Se_11_Th_4_: C, 29.91; H, 4.28. Found: C, 29.88; H, 4.30. After isolation of the orange crystals of **7**, the solvent of the mother liquid was removed. ^1^H NMR spectroscopy showed the presence of the resonances of **7** along with those of 2,2′-bipyridine and (2,3,5-(Me_3_C)_3_C_5_H_2_)_2_^138^ (^1^H NMR (C_6_D_6_): δ 6.48 (4H, C*H*), 1.38 (s, 36H, C(C*H*_3_)_3_), 1.01 (s, 18H, C(C*H*_3_)_3_) ppm) in the residue.

### Preparation of [(Cp^3*t*Bu^)_2_Th]_2_(μ-S_2_C=CS_2_) (**8**)

#### Method A

This compound was obtained as colorless crystals from the reaction of (Cp^3*t*Bu^)_2_Th(bipy) (**1**; 214 mg, 0.25 mmol) and CS_2_ (19.0 mg, 0.25 mmol) in toluene (15 mL) at room temperature, followed by recrystallization from an *n*-hexane solution using a similar procedure as outlined in the synthesis of **4**. The product was isolated by filtration, quickly washed with cold *n*-hexane (2 mL), and dried at room temperature under vacuum overnight. Yield: 167 mg (86%). M.p.: 166–168 °C (dec.). ^1^H NMR (C_6_D_6_): δ 6.55 (s, 4H, ring C*H*), 1.62 (s, 36H, C(C*H*_3_)_3_), 1.43 (s, 18H, C(C*H*_3_)_3_) ppm. ^13^C{^1^H} NMR (C_6_D_6_): δ 149.3 (ring *C*), 136.6 (ring *C*), 123.6 (ring *C*), 121.1 (C=*C*), 34.3 (C(*C*H_3_)_3_), 34.0 (*C*(CH_3_)_3_), 33.7 (*C*(CH_3_)_3_), 32.7 (C(*C*H_3_)_3_) ppm. IR (KBr, cm^–1^): *v* 2958 (s), 2867 (s), 1599 (s), 1477 (s), 1458 (s), 1360 (s), 1237 (s), 1104 (s), 1012 (s), 800 (s). Anal. calcd. for C_70_H_116_S_4_Th_2_: C, 54.24; H, 7.54. Found: C, 54.26; H, 7.53.

#### Method B

##### NMR Scale

A C_6_D_6_ (0.3 mL) solution of CS_2_ (1.5 mg, 0.02 mmol) was slowly added to a J. Young NMR tube charged with (Cp^3*t*Bu^)_2_Th(bipy) (**1**; 17.1 mg, 0.02 mmol) and C_6_D_6_ (0.2 mL). Resonances of **8** and those of 2,2′-bipyridine were observed by ^1^H NMR spectroscopy (100% conversion) when this solution was kept at room temperature overnight.

### Preparation of {[(2,3,5-(Me_3_C)_3_C_5_H_2_)C(NPh)S](Cp^3*t*Bu^)Th}_2_(μ-S)_2_ (**9**)

#### Method A

This compound was prepared as colorless crystals from the reaction of (Cp^3*t*Bu^)_2_Th(bipy) (**1**; 214 mg, 0.25 mmol) and PhNCS (68 mg, 0.50 mmol) in toluene (15 mL) at 80 °C and recrystallization from a benzene solution by a similar procedure as that in the synthesis of **4**. The product was isolated by filtration, quickly washed with cold *n*-hexane (2 mL), and dried at room temperature under vacuum overnight. Yield: 264 mg (82%). M.p.: 206–208 °C (dec.). ^1^H NMR (C_6_D_6_): δ 7.20 (m, 2H, phenyl), 6.68 (m, 2H, phenyl), 6.56 (s, 2H, ring C*H*), 6.45 (m, 1H, phenyl), 5.97 (t, *J* = 1.8 Hz, 1H, ring C*H*), 2.97 (s, 1H, ring C*H*), 1.59 (s, 9H, C(C*H*_3_)_3_), 1.31 (s, 9H, C(C*H*_3_)_3_), 1.26 (s, 18H, C(C*H*_3_)_3_), 1.18 (s, 9H, C(C*H*_3_)_3_), 1.09 (s, 9H, C(C*H*_3_)_3_) ppm. ^13^C{^1^H} NMR (C_6_D_6_): δ 160.1 (phenyl *C*), 156.6 (phenyl *C*), 153.8 (phenyl *C*), 149.3 (phenyl *C*), 136.6 (ring *C*), 129.6 (ring *C*), 126.9 (ring *C*), 126.7 (ring *C*), 125.7 (ring *C*), 123.6 (ring *C*), 121.1 (ring *C*), 119.3 (N=*C*), 63.3 (ring *C*), 35.5 ((CH_3_)_3_*C*), 34.2 ((*C*H_3_)_3_C), 34.1 ((*C*H_3_)_3_C), 34.02 ((CH_3_)_3_*C*), 33.98 ((*C*H_3_)_3_C), 33.9 ((*C*H_3_)_3_C), 32.7 ((CH_3_)_3_*C*), 32.3 ((*C*H_3_)_3_C), 32.2 ((CH_3_)_3_*C*), 32.1 ((*C*H_3_)_3_C), 30.4 ((CH_3_)_3_*C*), 29.7 ((CH_3_)_3_*C*) ppm. IR (KBr, cm^–1^): *v* 2958 (s), 1593 (s), 1491 (s), 1458 (s), 1389 (s), 1362 (s), 1239 (s), 1166 (m), 824 (s). Anal. calcd. for C_82_H_126_N_2_S_4_Th_2_: C, 56.86; H, 7.33; N, 1.62. Found: C, 56.88; H, 7.30; N, 1.64.

#### Method B

##### NMR Scale

A C_6_D_6_ (0.3 mL) solution of PhNCS (5.4 mg, 0.04 mmol) was slowly added to a J. Young NMR tube charged with (Cp^3*t*Bu^)_2_Th(bipy) (**1**; 17.1 mg, 0.02 mmol) and C_6_D_6_ (0.2 mL). Resonances of **9** and those of 2,2′-bipyridine and PhNC (^1^H NMR (C_6_D_6_): δ 7.82 (d, *J* = 7.8 Hz, 2H), 7.10 (m, 3H) ppm)^142^ were observed by ^1^H NMR spectroscopy (100% conversion) when this solution was kept at 80 °C overnight.

#### Method C

##### NMR Scale

A C_6_D_6_ (0.3 mL) solution of PhNCS (2.7 mg, 0.02 mmol) was slowly added to a J. Young NMR tube charged with [(Cp^3*t*Bu^)_2_Th]_2_(μ-S)_2_ (14.6 mg, 0.01 mmol) and C_6_D_6_ (0.2 mL). Resonances of **9** were observed by ^1^H NMR spectroscopy (100% conversion) when this solution was kept at 80 °C overnight.

### Preparation of (Cp^3*t*Bu^)_2_Th[(bipy)(Ph_2_CO)] (**10**)

#### Method A

This compound was prepared as purple crystals from the reaction of (Cp^3*t*Bu^)_2_Th(bipy) (**1**; 214 mg, 0.25 mmol) and Ph_2_CO (46 mg, 0.25 mmol) in toluene (15 mL) at room temperature and recrystallization from a benzene solution by a similar procedure as that in the synthesis of **4**. The product was isolated by filtration, quickly washed with cold *n*-hexane (2 mL), and dried at room temperature under vacuum overnight. Yield: 218 mg (84%). M.p.: 211–213 °C (dec.). ^1^H NMR (C_6_D_6_): δ 8.63 (s, 1H, bipy), 8.02 (s, 2H, phenyl), 7.87 (s, 2H, phenyl), 7.35 (s, 4H, phenyl), 7.24 (s, 3H, bipy and phenyl), 7.14 (s, 1H, bipy), 7.11 (s, 1H, bipy), 6.81 (s, 1H, ring C*H*), 6.61 (s, 2H, ring C*H*), 6.37 (m, 3H, bipy and ring C*H*), 5.94 (s, 1H, bipy), 5.04 (s, 1H, bipy), 1.47 (s, 9H, C(C*H*_3_)_3_), 1.42 (s, 9H, C(C*H*_3_)_3_), 1.28 (s, 9H, C(C*H*_3_)_3_), 1.22 (s, 18H, C(C*H*_3_)_3_), 1.08 (s, 9H, C(C*H*_3_)_3_) ppm. ^13^C{^1^H} NMR (C_6_D_6_): δ 163.0 (bipy *C*), 150.7 (bipy *C*), 149.3 (bipy *C*), 148.8 (bipy *C*), 147.3 (bipy *C*), 144.1 (phenyl *C*), 142.5 (phenyl *C*), 140.5 (phenyl *C*), 139.6 (phenyl *C*), 137.0 (phenyl *C*), 132.2 (phenyl *C*), 126.9 (phenyl *C*), 126.8 (phenyl *C*), 126.7 (ring *C*), 122.9 (ring *C*), 122.0 (ring *C*), 121.1 (ring *C*), 119.9 (ring *C*), 119.0 (ring *C*), 118.2 (ring *C*), 117.3 (ring *C*), 116.8 (ring *C*), 115.5 (ring *C*), 115.0 (bipy *C*), 114.5 (bipy *C*), 99.5 (bipy *C*), 98.0 (bipy *C*), 96.3 (O*C*), 77.6 (bipy *C*), 35.3 ((CH_3_)_3_*C*), 35.0 ((CH_3_)_3_*C*), 34.8 ((CH_3_)_3_*C*), 34.4 ((CH_3_)_3_*C*), 34.2 ((CH_3_)_3_*C*), 33.8 ((CH_3_)_3_*C*), 33.1 ((*C*H_3_)_3_C), 32.9 ((*C*H_3_)_3_C), 32.2 ((*C*H_3_)_3_C), 31.6 ((*C*H_3_)_3_C), 30.5 ((*C*H_3_)_3_C), 29.7 ((*C*H_3_)_3_C) ppm. IR (KBr, cm^–1^): *v* 2957 (s), 1603 (m), 1477 (s), 1360 (s), 1236 (m), 1164 (s), 1016 (s), 823 (s). Anal. Calcd for C_57_H_76_N_2_OTh: C, 66.00; H, 7.39; N, 2.70. Found: C, 65.98; H, 7.36; N, 2.73.

#### Method B

##### NMR Scale

A C_6_D_6_ (0.3 mL) solution of Ph_2_CO (3.6 mg, 0.02 mmol) was slowly added to a J. Young NMR tube charged with (Cp^3*t*Bu^)_2_Th(bipy) (**1**; 17.1 mg, 0.02 mmol) and C_6_D_6_ (0.2 mL). Resonances of **10** were observed by ^1^H NMR spectroscopy (100% conversion) when this solution was kept at room temperature overnight.

### Preparation of (Cp^3*t*Bu^)_2_Th[(bipy)((CH_2_)_5_CO)] (**11**)

#### Method A

This compound was prepared as purple crystals from the reaction of (Cp^3*t*Bu^)_2_Th(bipy) (**1**; 214 mg, 0.25 mmol) and (CH_2_)_5_CO (25 mg, 0.25 mmol) in toluene (15 mL) at room temperature and recrystallization from a benzene solution by a similar procedure as that in the synthesis of **4**. The product was isolated by filtration, quickly washed with cold *n*-hexane (2 mL) and dried at room temperature under vacuum overnight. Yield: 196 mg (82%). M.p.: 222–224 °C (dec.). ^1^H NMR (C_6_D_6_): δ 8.55 (s, 1H, bipy), 7.32 (d, *J* = 8.4 Hz, 1H, bipy), 7.15 (s, 2H, ring C*H*), 6.76 (t, *J* = 7.7 Hz, 1H, bipy), 6.61 (s, 1H, ring C*H*), 6.50 (t, *J* = 5.7 Hz, 1H, bipy), 6.33 (s, 1H, ring C*H*), 6.08 (m, 1H, bipy), 5.76 (s, 1H, bipy), 5.30 (s, 1H, bipy), 5.08 (d, *J* = 9.0 Hz, 1H, bipy), 1.96–1.37 (m, 64H, Cy and C(C*H*_3_)_3_) ppm. ^13^C{^1^H} NMR (C_6_D_6_): δ 163.1 (bipy *C*), 150.6 (bipy *C*), 147.8 (bipy *C*), 144.1 (bipy *C*), 142.7 (bipy *C*), 140.3 (ring *C*), 139.3 (ring *C*), 136.6 (ring *C*), 129.6 (ring *C*), 128.3 (ring *C*), 127.9 (ring *C*), 126.7 (ring *C*), 123.9 (ring *C*), 122.1 (ring *C*), 119.7 (ring *C*), 119.2 (bipy *C*), 115.7 (bipy *C*), 114.6 (bipy *C*), 112.0 (bipy *C*), 96.2 (O*C*), 77.7 (bipy *C*), 36.8 ((CH_3_)_3_*C*), 36.2 ((CH_3_)_3_*C*), 35.8 ((CH_3_)_3_*C*), 35.6 ((CH_3_)_3_*C*), 35.0 ((CH_3_)_3_*C*), 34.6 ((CH_3_)_3_*C*), 34.5 ((*C*H_3_)_3_C), 34.0 ((*C*H_3_)_3_C), 32.8 ((*C*H_3_)_3_C), 32.2 ((*C*H_3_)_3_C), 30.5 ((*C*H_3_)_3_C), 29.8 ((*C*H_3_)_3_C), 27.0 (Cy *C*), 23.1 (Cy *C*), 22.8 (Cy *C*) ppm. IR (KBr, cm^–1^): *v* 2955 (s), 1604 (m), 1465 (s), 1373 (s), 1249 (s), 1026 (s), 802 (s). Anal. calcd. for C_50_H_76_N_2_OTh: C, 63.00; H, 8.04; N, 2.94. Found: C, 62.97; H, 8.06; N, 2.96.

#### Method B

##### NMR Scale

A C_6_D_6_ (0.3 mL) solution of (CH_2_)_5_CO (2.0 mg, 0.02 mmol) was slowly added to a J. Young NMR tube charged with (Cp^3*t*Bu^)_2_Th(bipy) (**1**; 17.1 mg, 0.02 mmol) and C_6_D_6_ (0.2 mL). Resonances of **11** were observed by ^1^H NMR spectroscopy (100% conversion) when this solution was kept at room temperature overnight.

### Preparation of (Cp^3*t*Bu^)_2_Th[(bipy)(*p*-MePhCHO)] (**12**).

#### Method A

This compound was prepared as purple microcrystals from the reaction of (Cp^3*t*Bu^)_2_Th(bipy) (**1**; 214 mg, 0.25 mmol) and *p*-tolylCHO (30 mg, 0.25 mmol) in toluene (15 mL) at room temperature and recrystallization from a benzene solution by a similar procedure as that in the synthesis of **4**. The product was isolated by filtration, quickly washed with cold *n*-hexane (2 mL), and dried at room temperature under vacuum overnight. Yield: 205 mg (84%). M.p.: 262–264 °C (dec.). ^1^H NMR (C_6_D_6_): δ 8.62 (d, *J* = 5.3 Hz, 1H, bipy), 7.62 (d, *J* = 8.0 Hz, 2H, phenyl), 7.36 (d, *J* = 8.4 Hz, 1H, bipy), 7.14 (d, *J* = 8.0 Hz, 2H, phenyl), 6.82 (m, 1H, bipy), 6.66 (d, *J* = 3.3 Hz, 1H, ring C*H*), 6.55 (d, *J* = 3.3 Hz, 1H, ring C*H*), 6.52 (m, 1H, bipy), 6.30 (s, 1H, ring C*H*), 6.13 (m, 2H, bipy), 6.06 (s, 1H, ring C*H*), 5.68 (d, *J* = 8.9 Hz, 1H, bipy), 5.59 (d, *J* = 5.8 Hz, 1H, bipy), 5.09 (m, 1H, OC*H*), 2.19 (s, 3H, C*H*_3_), 1.64 (s, 9H, C(C*H*_3_)_3_), 1.51 (s, 9H, C(C*H*_3_)_3_), 1.443 (s, 9H, C(C*H*_3_)_3_), 1.437 (s, 9H, C(C*H*_3_)_3_), 1.33 (s, 9H, C(C*H*_3_)_3_), 1.15 (s, 9H, C(C*H*_3_)_3_) ppm. ^13^C{^1^H} NMR (C_6_D_6_): δ 164.0 (bipy *C*), 152.4 (bipy *C*), 147.6 (bipy *C*), 145.1 (bipy *C*), 143.5 (bipy *C*), 141.8 (phenyl *C*), 141.4 (phenyl *C*), 140.5 (phenyl *C*), 139.8 (phenyl *C*), 139.1 (ring *C*), 137.1 (ring *C*), 136.4 (ring *C*), 129.0 (ring *C*), 128.5 (ring *C*), 128.3 (ring *C*), 125.8 (ring *C*), 122.0 (ring *C*), 119.0 (ring *C*), 117.3 (ring *C*), 116.0 (bipy *C*), 115.8 (bipy *C*), 115.1 (bipy *C*), 100.8 (bipy *C*), 89.2 (*C*O), 70.8 (bipy *C*), 35.23 ((CH_3_)_3_*C*), 35.19 ((*C*H_3_)_3_C), 34.7 ((CH_3_)_3_*C*), 34.6 ((CH_3_)_3_*C*), 34.4 ((*C*H_3_)_3_C), 34.36 ((CH_3_)_3_*C*), 34.3 ((CH_3_)_3_*C*), 34.2 ((*C*H_3_)_3_C), 33.04 ((*C*H_3_)_3_C), 33.02 ((*C*H_3_)_3_C), 21.2 (*C*H_3_) ppm. IR (KBr, cm^–1^): *v* 2954 (s), 1604 (m), 1465 (s), 1373 (s), 1242 (s), 1026 (s), 810 (s). Anal. calcd. for C_52_H_74_N_2_OTh: C, 64.04; H, 7.65; N, 2.87. Found: C, 64.06; H, 7.66; N, 2.85. Purple crystals of **12**·0.5C_6_H_14_ suitable for X-ray structural analysis were isolated from a mixture of benzene and *n*-hexane (4:1) solution.

#### Method B

##### NMR Scale

A C_6_D_6_ (0.3 mL) solution of *p*-tolylCHO (2.4 mg, 0.02 mmol) was slowly added to a J. Young NMR tube charged with (Cp^3*t*Bu^)_2_Th(bipy) (**1**; 17.1 mg, 0.02 mmol) and C_6_D_6_ (0.2 mL). Resonances of **12** were observed by ^1^H NMR spectroscopy (100% conversion) when this solution was kept at room temperature overnight.

### Preparation of (Cp^3*t*Bu^)_2_Th[(bipy)(*p*-ClPhCHO)] (**13**)

#### Method A

This compound was prepared as orange microcrystals from the reaction of (Cp^3*t*Bu^)_2_Th(bipy) (**1**; 214 mg, 0.25 mmol) and *p*-ClPhCHO (36 mg, 0.25 mmol) in toluene (15 mL) at room temperature and recrystallization from a benzene solution by a similar procedure as that in the synthesis of **4**. The product was isolated by filtration, quickly washed with cold *n*-hexane (2 mL) and dried at room temperature under vacuum overnight. Yield: 214 mg (86%). M.p.: 177–179 °C (dec.). ^1^H NMR (C_6_D_6_): δ 8.60 (d, *J* = 5.2 Hz, 1H, bipy), 7.46 (d, *J* = 8.4 Hz, 2H, phenyl), 7.35 (d, *J* = 8.4 Hz, 1H, bipy), 7.26 (d, *J* = 8.4 Hz, 2H, phenyl), 6.81 (t, *J* = 7.8 Hz, 1H, bipy), 6.60 (d, *J* = 3.2 Hz, 1H, ring C*H*), 6.51 (t, *J* = 6.3 Hz, 1H, bipy), 6.47 (d, *J* = 3.2 Hz, 1H, ring C*H*), 6.31 (s, 1H, ring C*H*), 6.14 (m, 1H, bipy), 6.01 (s, 1H, ring C*H*), 5.98 (d, *J* = 10.0 Hz, 1H, bipy), 5.60 (d, *J* = 5.7 Hz, 1H, bipy), 5.48 (d, *J* = 8.7 Hz, 1H, bipy), 4.94 (m, 1H, OC*H*), 1.60 (s, 9H, C(C*H*_3_)_3_), 1.48 (s, 9H, C(C*H*_3_)_3_), 1.40 (s, 9H, C(C*H*_3_)_3_), 1.36 (s, 9H, C(C*H*_3_)_3_), 1.31 (s, 9H, C(C*H*_3_)_3_), 1.10 (s, 9H, C(C*H*_3_)_3_) ppm. ^13^C{^1^H} NMR (C_6_D_6_): δ 163.9 (bipy *C*), 152.5 (bipy *C*), 147.6 (bipy *C*), 145.4 (bipy *C*), 143.6 (bipy *C*), 142.2 (phenyl *C*), 142.0 (phenyl *C*), 141.7 (phenyl *C*), 140.0 (phenyl *C*), 139.2 (ring *C*), 137.3 (ring *C*), 132.9 (ring *C*), 129.8 (ring *C*), 128.4 (ring *C*), 126.1 (ring *C*), 122.0 (ring *C*), 119.2 (ring *C*), 117.6 (ring *C*), 115.9 (ring *C*), 115.8 (bipy *C*), 115.2 (bipy *C*), 114.3 (bipy *C*), 100.9 (bipy *C*), 88.2 (*C*O), 70.7 (bipy *C*), 35.2 ((CH_3_)_3_*C*), 34.7 ((CH_3_)_3_*C*), 34.5 ((CH_3_)_3_*C*), 34.4 ((*C*H_3_)_3_C), 34.3 ((*C*H_3_)_3_C), 34.29 ((CH_3_)_3_*C*), 34.2 ((CH_3_)_3_*C*), 34.1 ((*C*H_3_)_3_C), 33.0 ((*C*H_3_)_3_C), 32.96 ((*C*H_3_)_3_C) ppm. IR (KBr, cm^–1^): *v* 2954 (s), 1604 (m), 1473 (s), 1373 (s), 1242 (s), 1033 (s), 817(s). Anal. calcd. for C_51_H_71_N_2_ClOTh: C, 61.52; H, 7.19; N, 2.81. Found: C, 61.55; H, 7.16; N, 2.83. Orange crystals of **13**·0.5C_6_H_14_ suitable for X-ray structural analysis were isolated from a mixture of benzene and *n*-hexane (4:1) solution.

#### Method B

##### NMR Scale

A C_6_D_6_ (0.3 mL) solution of *p*-ClPhCHO (2.8 mg, 0.02 mmol) was slowly added to a J. Young NMR tube charged with (Cp^3*t*Bu^)_2_Th(bipy) (**1**; 17.1 mg, 0.02 mmol) and C_6_D_6_ (0.2 mL). Resonances of **13** were observed by ^1^H NMR spectroscopy (100% conversion) when this solution was kept at room temperature overnight.

### Preparation of (Cp^3*t*Bu^)_2_Th[(bipy){(*p*-MeOPh)_2_CSe}] (**14**)

#### Method A

This compound was prepared as purple crystals from the reaction of (Cp^3*t*Bu^)_2_Th(bipy) (**1**; 214 mg, 0.25 mmol) and (*p*-MeOPh)_2_CSe (77 mg, 0.25 mmol) in toluene (15 mL) at room temperature and recrystallization from a benzene solution by a similar procedure as that in the synthesis of **4**. The product was isolated by filtration, quickly washed with cold *n*-hexane (2 mL), and dried at room temperature under vacuum overnight. Yield: 244 mg (84%). M.p.: 252–254 °C (dec.). ^1^H NMR (C_6_D_6_): δ 8.81 (d, *J* = 5.4 Hz, 1H, bipy), 8.18 (d, *J* = 8.9 Hz, 2H, phenyl), 8.14 (d, *J* = 8.4 Hz, 2H, phenyl), 7.37 (d, *J* = 8.5 Hz, 1H, bipy), 7.21 (s, 1H, bipy), 6.93 (d, *J* = 9.0 Hz, 2H, phenyl), 6.89 (d, *J* = 3.2 Hz, 1H, ring C*H*), 6.87 (d, *J* = 9.0 Hz, 2H, phenyl), 6.74 (m, 1H, bipy), 6.58 (d, *J* = 3.2 Hz, 1H, ring C*H*), 6.47 (t, *J* = 6.1 Hz, 1H, bipy), 6.41 (s, 1H, ring C*H*), 6.02 (s, 1H, ring C*H*), 5.97 (m, 1H, bipy), 5.22 (m, 2H, bipy), 3.36 (s, 3H, OC*H*_3_), 3.33 (s, 3H, OC*H*_3_), 1.78 (s, 9H, C(C*H*_3_)_3_), 1.66 (s, 9H, C(C*H*_3_)_3_), 1.44 (s, 9H, C(C*H*_3_)_3_), 1.40 (s, 9H, C(C*H*_3_)_3_), 1.37 (s, 9H, C(C*H*_3_)_3_), 0.72 (s, 9H, C(C*H*_3_)_3_) ppm. ^13^C{^1^H} NMR (C_6_D_6_): δ 163.1 (bipy *C*), 158.6 (bipy *C*), 158.2 (bipy *C*), 157.6 (bipy *C*), 154.2 (bipy *C*), 148.6 (phenyl *C*), 146.3 (phenyl *C*), 143.5 (phenyl *C*), 143.2 (phenyl *C*), 140.6 (phenyl *C*), 140.5 (phenyl *C*), 139.0 (phenyl *C*), 137.5 (phenyl *C*), 137.4 (ring *C*), 133.3 (ring *C*), 133.1 (ring *C*), 132.5 (ring *C*), 124.8 (ring *C*), 124.2 (ring *C*), 123.1 (ring *C*), 119.1 (ring *C*), 117.6 (ring *C*), 113.7 (ring *C*), 113.6 (bipy *C*), 112.8 (bipy *C*), 112.3 (bipy *C*), 99.6 (bipy *C*), 80.3 (*C*Se), 79.1 (bipy *C*), 54.7 (O*C*H_3_), 54.5 (O*C*H_3_), 36.2 ((*C*H_3_)_3_C), 35.9 ((CH_3_)_3_*C*), 35.2 ((CH_3_)_3_*C*), 34.7 ((CH_3_)_3_*C*), 34.67 ((CH_3_)_3_*C*), 34.4 ((CH_3_)_3_*C*), 33.8 ((*C*H_3_)_3_C), 33.7 ((*C*H_3_)_3_C), 33.4 ((*C*H_3_)_3_C), 32.4 ((*C*H_3_)_3_C) ppm. ^77^Se{^1^H} NMR (C_6_D_6_): δ 652.3 ppm. IR (KBr, cm^–1^): *v* 2947 (s), 1597 (m), 1489 (m), 1381 (m), 1257 (s), 1033 (s), 802 (s). Anal. calcd. for C_59_H_80_N_2_O_2_SeTh: C, 61.07; H, 6.95; N, 2.41. Found: C, 61.05; H, 6.96; N, 2.44.

#### Method B

##### NMR Scale

A C_6_D_6_ (0.3 mL) solution of (*p*-MeOPh)_2_CSe (6.1 mg, 0.02 mmol) was slowly added to a J. Young NMR tube charged with (Cp^3*t*Bu^)_2_Th(bipy) (**1**; 17.1 mg, 0.02 mmol) and C_6_D_6_ (0.2 mL). Resonances of **14** were observed by ^1^H NMR spectroscopy (100% conversion) when this solution was kept at room temperature overnight.

### Preparation of [(Cp^3*t*Bu^)Th]_2_[μ-NC(Ph)(bipy)]_2_ (**15**)

#### Method A

This compound was prepared as purple crystals from the reaction of (Cp^3*t*Bu^)_2_Th(bipy) (**1**; 214 mg, 0.25 mmol) and (PhCH=N)_2_ (52 mg, 0.25 mmol) in toluene (15 mL) at 120 °C and recrystallization from a benzene solution by a similar procedure as that in the synthesis of **4**. The product was isolated by filtration, quickly washed with cold *n*-hexane (2 mL) and dried at room temperature under vacuum overnight. Yield: 145 mg (80%). M.p.: 202–204 °C (dec.). ^1^H NMR (C_6_D_6_): δ 7.75 (d, *J* = 7.4 Hz, 4H, phenyl), 7.66 (d, *J* = 7.6 Hz, 2H, phenyl), 7.48 (s, 2H, bipy), 7.01 (s, 2H, bipy), 6.87 (s, 2H, bipy), 6.81 (s, 2H, bipy), 6.71 (d, *J* = 8.3 Hz, 2H, bipy), 6.67 (m, 4H, phenyl), 6.45 (s, 2H, bipy), 6.35 (s, 2H, bipy), 6.12 (s, 2H, ring C*H*), 5.97 (s, 2H, ring C*H*), 1.40 (s, 36H, C(C*H*_3_)_3_), 1.31 (s, 18H, C(C*H*_3_)_3_) ppm. ^13^C{^1^H} NMR (C_6_D_6_): δ 170.7 (Th*C*), 160.1 (bipy *C*), 156.6 (bipy *C*), 153.8 (bipy *C*), 149.3 (bipy *C*), 147.5 (bipy *C*), 141.0 (bipy *C*), 140.5 (bipy *C*), 136.6 (bipy *C*), 129.6 (bipy *C*), 128.9 (bipy *C*), 128.8 (phenyl *C*), 128.6 (phenyl *C*), 126.7 (phenyl *C*), 125.5 (phenyl *C*), 121.1 (ring *C*), 119.3 (ring *C*), 114.0 (ring *C*), 34.5 ((CH_3_)_3_*C*), 34.0 ((CH_3_)_3_*C*), 30.4 ((*C*H_3_)_3_C), 29.7 ((*C*H_3_)_3_C) ppm. IR (KBr, cm^–1^): *v* 2958 (s), 1658 (s), 1581 (s), 1457 (s), 1388 (s), 1360 (s), 1238 (s), 1164 (s), 823 (s). Anal. calcd. for C_68_H_82_N_6_Th_2_: C, 56.42; H, 5.71; N, 5.81. Found: C, 56.45; H, 5.69; N, 5.83.

#### Method B

##### NMR Scale

A C_6_D_6_ (0.3 mL) solution of (PhCH=N)_2_ (4.2 mg, 0.02 mmol) was slowly added to a J. Young NMR tube charged with (Cp^3*t*Bu^)_2_Th(bipy) (**1**; 17.1 mg, 0.02 mmol) and C_6_D_6_ (0.2 mL). Resonances of **15** and those of (Me_3_C)_3_C_5_H_3_^138^ and PhCH=NH^162^ were observed by ^1^H NMR spectroscopy (100% conversion) when this solution was kept at 120 °C overnight.

### Preparation of (Cp^3*t*Bu^)_2_Th[(bipy)(PhCN)] (**16**)

#### Method A

This compound was prepared as orange crystals from the reaction of (Cp^3*t*Bu^)_2_Th(bipy) (**1**; 214 mg, 0.25 mmol) and PhCN (26 mg, 0.25 mmol) in toluene (15 mL) at room temperature and recrystallization from a benzene solution by a similar procedure as that in the synthesis of **4**. The product was isolated by filtration, quickly washed with cold *n*-hexane (2 mL), and dried at room temperature under vacuum overnight. Yield: 201 mg (84%). M.p.: 198–200 °C (dec.). ^1^H NMR (C_6_D_6_): δ 8.60 (d, *J* = 5.3 Hz, 1H, bipy), 8.05 (d, *J* = 7.9 Hz, 2H, phenyl), 7.32 (m, 3H, phenyl and bipy), 7.18 (t, *J* = 7.3 Hz, 1H, phenyl), 6.81 (t, *J* = 7.7 Hz, 1H, bipy), 6.64 (s, 1H, bipy), 6.55 (d, *J* = 3.3 Hz, 1H, ring C*H*), 6.51 (m, 1H, bipy), 6.37 (d, *J* = 3.0 Hz, 1H, ring C*H*), 6.24 (s, 1H, ring C*H*), 6.11 (m, 1H, bipy), 6.06 (s, 1H, ring C*H*), 5.67 (d, *J* = 5.8 Hz, 1H, bipy), 5.48 (m 1H, bipy), 1.64 (s, 9H, C(C*H*_3_)_3_), 1.52 (s, 9H, C(C*H*_3_)_3_), 1.45 (s, 9H, C(C*H*_3_)_3_), 1.35 (s, 9H, C(C*H*_3_)_3_), 1.25 (s, 9H, C(C*H*_3_)_3_), 1.14 (s, 9H, C(C*H*_3_)_3_) ppm. ^13^C{^1^H} NMR (C_6_D_6_): δ 176.6 (bipy *C*), 164.6 (bipy *C*), 153.2 (bipy *C*), 147.2 (bipy *C*), 143.3 (bipy *C*), 142.9 (phenyl *C*), 142.8 (phenyl *C*), 142.3 (phenyl *C*), 140.5 (phenyl *C*), 140.2 (ring *C*), 138.0 (ring *C*), 137.5 (ring *C*), 129.3 (ring *C*), 128.54 (ring *C*), 128.51 (ring *C*), 124.9 (ring *C*), 121.9 (ring *C*), 119.4 (ring *C*), 117.4 (ring *C*), 116.0 (bipy *C*), 115.8 (bipy *C*), 114.5 (bipy *C*), 113.7 (bipy *C*), 102.4 (N=*C*), 79.5 (bipy *C*), 35.4 ((CH_3_)_3_*C*), 35.0 ((CH_3_)_3_*C*), 34.7 ((CH_3_)_3_*C*), 34.5 ((CH_3_)_3_*C*), 34.4 ((*C*H_3_)_3_C), 34.3 ((*C*H_3_)_3_C), 34.0 ((*C*H_3_)_3_C), 33.2((CH_3_)_3_*C*), 33.1 ((*C*H_3_)_3_C) ppm. IR (KBr, cm^–1^): *v* 2955 (s), 1604 (s), 1465 (s), 1373 (s), 1257 (s), 1157 (s), 1010 (s), 933 (s), 817 (s). Anal. calcd. for C_51_H_71_N_3_Th: C, 63.93; H, 7.47; N, 4.39. Found: C, 63.95; H, 7.46; N, 4.36.

#### Method B

##### NMR Scale

A C_6_D_6_ (0.3 mL) solution of PhCN (2.1 mg, 0.02 mmol) was slowly added to a J. Young NMR tube charged with (Cp^3*t*Bu^)_2_Th(bipy) (**1**; 17.1 mg, 0.02 mmol) and C_6_D_6_ (0.2 mL). Resonances of **16** were observed by ^1^H NMR spectroscopy (100% conversion) when this solution was kept at room temperature overnight.

### Preparation of (Cp^3*t*Bu^)_2_Th[(bipy)(Ph_2_CHCN)] (**17**)

#### Method A

This compound was prepared as orange crystals from the reaction of (Cp^3*t*Bu^)_2_Th(bipy) (**1**; 214 mg, 0.25 mmol) and Ph_2_CHCN (43 mg, 0.25 mmol) in toluene (15 mL) at room temperature and recrystallization from a benzene solution by a similar procedure as that in the synthesis of **4**. The product was isolated by filtration, quickly washed with cold *n*-hexane (2 mL), and dried at room temperature under vacuum overnight. Yield: 215 mg (82%). M.p.: 173–175 °C (dec.). ^1^H NMR (C_6_D_6_): δ 8.65 (d, *J* = 5.3 Hz, 1H, bipy), 7.84 (d, *J* = 7.8 Hz, 2H, phenyl), 7.63 (d, *J* = 7.4 Hz, 2H, phenyl), 7.21 (m, 5H, phenyl and bipy), 7.09 (m, 2H, phenyl), 6.75 (m, 2H, phenyl), 6.48 (m, 1H, bipy), 6.41 (d, *J* = 3.2 Hz, 1H, ring C*H*), 6.32 (s, 1H, ring C*H*), 6.11 (s, 1H, ring C*H*), 5.97 (m, 2H, bipy and ring C*H*), 5.54 (d, *J* = 5.7 Hz, 1H, bipy), 5.50 (m, 1H, bipy), 5.29 (s, 1H, C*H*), 1.66 (s, 9H, C(C*H*_3_)_3_), 1.52 (s, 9H, C(C*H*_3_)_3_), 1.25 (m, 27H, C(C*H*_3_)_3_), 1.06 (s, 9H, C(C*H*_3_)_3_) ppm. ^13^C{^1^H} NMR (C_6_D_6_): δ 178.3 (bipy *C*), 164.2 (bipy *C*), 153.1 (bipy *C*), 147.8 (bipy *C*), 143.1 (bipy *C*), 142.9 (phenyl *C*), 142.5 (phenyl *C*), 140.9 (phenyl *C*), 140.5 (phenyl *C*), 137.3 (ring *C*), 130.4 (ring *C*), 130.2 (ring *C*), 128.4 (ring *C*), 126.3 (ring *C*), 126.2 (ring *C*), 125.2 (ring *C*), 121.8 (ring *C*), 118.9 (ring *C*), 118.0 (ring *C*), 117.2 (bipy *C*), 116.4 (bipy *C*), 114.9 (bipy *C*), 114.1 (bipy *C*), 102.7 (*C*=N), 82.9 (bipy *C*), 60.7 (*C*H), 35.1 ((CH_3_)_3_*C*), 34.9 ((CH_3_)_3_*C*), 34.7 ((CH_3_)_3_*C*), 34.6 ((CH_3_)_3_*C*), 34.4 ((*C*H_3_)_3_C), 34.36 ((*C*H_3_)_3_C), 34.2 ((*C*H_3_)_3_C), 34.1 ((CH_3_)_3_*C*), 33.3 ((*C*H_3_)_3_C), 33.2 ((*C*H_3_)_3_C) ppm. IR (KBr, cm^–1^): *v* 2955 (s), 1612 (s), 1473 (s), 1365 (s), 1249 (s), 1157 (m), 1003 (m), 817 (s). Anal. calcd. for C_58_H_77_N_3_Th: C, 66.45; H, 7.40; N, 4.01. Found: C, 66.48; H, 7.38; N, 4.02.

#### Method B

##### NMR Scale

A C_6_D_6_ (0.3 mL) solution of Ph_2_CHCN (3.4 mg, 0.02 mmol) was slowly added to a J. Young NMR tube charged with (Cp^3*t*Bu^)_2_Th(bipy) (**1**; 17.1 mg, 0.02 mmol) and C_6_D_6_ (0.2 mL). Resonances of **17** were observed by ^1^H NMR spectroscopy (100% conversion) when this solution was kept at room temperature overnight.

### Preparation of (Cp^3^*t*Bu)_2_Th[(bipy)(C_6_H_11_CN)]·0.5C_6_H_6_ (**18**·0.5C_6_H_6_)

#### Method A

This compound was prepared as orange crystals from the reaction of (Cp^3*t*Bu^)_2_Th(bipy) (**1**; 214 mg, 0.25 mmol) and C_6_H_11_CN (28 mg, 0.25 mmol) in toluene (15 mL) at room temperature and recrystallization from a benzene solution by a similar procedure as that in the synthesis of **4**. The product was isolated by filtration, quickly washed with cold *n*-hexane (2 mL), and dried at room temperature under vacuum overnight. Yield: 201 mg (80%). M.p.: 221–223 °C (dec.). ^1^H NMR (C_6_D_6_): δ 8.61 (t, *J* = 4.2 Hz, 1H, bipy), 7.30 (m, 1H, bipy), 7.15 (s, 3H, C_6_*H*_6_), 6.78 (m, 1H, bipy), 6.49 (m, 1H, bipy), 6.45 (s, 1H, ring C*H*), 6.33 (s, 1H, ring C*H*), 6.15 (m, 4H, ring C*H* and bipy), 5.60 (t, *J* = 4.9 Hz, 1H, bipy), 5.42 (s, 1H, bipy), 2.62 (m, 1H, Cy), 2.32 (d, *J* = 12.6 Hz, 1H, Cy), 2.17 (d, *J* = 11.6 Hz, 1H, Cy), 1.97 (d, *J* = 10.4 Hz, 1H, Cy), 1.87 (m, 2H, Cy), 1.76 (m, 2H, Cy), 1.60 (s, 18H, C(C*H*_3_)_3_), 1.51 (s, 9H, C(C*H*_3_)_3_), 1.44 (s, 9H, C(C*H*_3_)_3_), 1.37 (m, 3H, Cy), 1.22 (s, 9H, C(C*H*_3_)_3_), 1.16 (s, 9H, C(C*H*_3_)_3_) ppm. ^13^C{^1^H} NMR (C_6_D_6_): δ 184.5 (bipy *C*), 164.5 (bipy *C*), 153.3 (bipy *C*), 147.2 (bipy *C*), 142.7 (bipy *C*), 142.5 (ring *C*), 142.4 (ring *C*), 141.8 (ring *C*), 140.1 (ring *C*), 139.7 (ring *C*), 137.2 (ring *C*), 128.5 (*C*_6_H_6_), 128.3 (ring *C*), 125.3 (ring *C*), 121.9 (ring *C*), 119.2 (ring *C*), 117.7 (bipy *C*), 115.4 (bipy *C*), 114.9 (bipy *C*), 113.6 (bipy *C*), 101.8 (N=*C*), 80.8 (bipy *C*), 45.3 (Cy *C*), 34.8 ((CH_3_)_3_*C*), 34.6 ((CH_3_)_3_*C*), 34.4 ((CH_3_)_3_*C*), 34.36 ((CH_3_)_3_*C*), 34.3 ((*C*H_3_)_3_C), 33.4 ((*C*H_3_)_3_C), 33.3 ((*C*H_3_)_3_C), 31.3 ((CH_3_)_3_*C*), 30.4 ((*C*H_3_)_3_C), 27.5 (Cy *C*), 27.2 (Cy *C*), 27.1 (Cy *C*) ppm. IR (KBr, cm^–1^): *v* 2939 (s), 1604 (s), 1465 (s), 1373 (s), 1249 (s), 1018 (s), 802 (s). Anal. Calcd for C_54_H_80_N_3_Th: C, 64.65; H, 8.04; N, 4.19. Found: C, 64.68; H, 8.06; N, 4.16.

#### Method B

##### NMR Scale

A C_6_D_6_ (0.3 mL) solution of C_6_H_11_CN (2.2 mg, 0.02 mmol) was slowly added to a J. Young NMR tube charged with (Cp^3*t*Bu^)_2_Th(bipy) (**1**; 17.1 mg, 0.02 mmol) and C_6_D_6_ (0.2 mL). Resonances of **18** were observed by ^1^H NMR spectroscopy (100% conversion) when this solution was kept at room temperature overnight.

### Preparation of (Cp^3*t*Bu^)_2_Th[(bipy){C(=CHPh)NH}] (**19**)

#### Method A

This compound was prepared as purple crystals from the reaction of (Cp^3*t*Bu^)_2_Th(bipy) (**1**; 214 mg, 0.25 mmol) and PhCH_2_CN (30 mg, 0.25 mmol) in toluene (15 mL) at room temperature and recrystallization from a benzene solution by a similar procedure as that in the synthesis of **4**. The product was isolated by filtration, quickly washed with cold *n*-hexane (2 mL), and dried at room temperature under vacuum overnight. Yield: 199 mg (82%). M.p.: 182–184 °C (dec.). ^1^H NMR (C_6_D_6_): δ 8.69 (d, *J* = 5.4 Hz, 1H, bipy), 7.67 (d, *J* = 7.8 Hz, 2H, phenyl), 7.41 (t, *J* = 7.1 Hz, 2H, phenyl), 7.32 (d, *J* = 8.4 Hz, 1H, phenyl), 7.09 (t, *J* = 7.6 Hz, 1H, bipy), 6.80 (t, *J* = 7.8 Hz, 1H, bipy), 6.70 (s, 1H, C*H*), 6.49 (t, *J* = 6.3 Hz, 1H, bipy), 6.43 (s, 2H, ring C*H*), 6.25 (s, 1H, ring C*H*), 6.19 (t, *J* = 7.2 Hz, 1H, bipy), 5.97 (s, 1H, ring C*H*), 5.74 (d, *J* = 5.8 Hz, 1H, bipy), 5.71 (d, *J* = 8.5 Hz, 1H, bipy), 5.57 (s, 1H, bipy), 5.50 (s, 1H, N*H*), 1.44 (s, 9H, C(C*H*_3_)_3_), 1.43 (s, 9H, C(C*H*_3_)_3_), 1.41 (s, 9H, C(C*H*_3_)_3_), 1.39 (s, 9H, C(C*H*_3_)_3_), 1.23 (s, 9H, C(C*H*_3_)_3_), 1.04 (s, 9H, C(C*H*_3_)_3_) ppm. ^13^C{^1^H} NMR (C_6_D_6_): δ 164.3 (bipy *C*), 161.5 (bipy *C*), 153.3 (bipy *C*), 147.6 (bipy *C*), 145.8 (bipy *C*), 143.4 (phenyl *C*), 142.0 (phenyl *C*), 141.9 (phenyl *C*), 141.2 (phenyl *C*), 141.0 (ring *C*), 140.5 (ring *C*), 137.5 (ring *C*), 129.8 (ring *C*), 128.7 (ring *C*), 128.3 (ring *C*), 124.4 (ring *C*), 123.0 (ring *C*), 122.0 (ring *C*), 119.6 (ring *C*), 118.8 (bipy *C*), 115.6 (bipy *C*), 113.0 (bipy *C*), 104.3 (N*C*=C), 92.1 (C=*C*H), 68.4 (bipy *C*), 35.5 ((CH_3_)_3_*C*), 35.1 ((*C*H_3_)_3_C), 34.9 ((CH_3_)_3_*C*), 34.8 ((CH_3_)_3_*C*), 34.6 ((CH_3_)_3_*C*), 34.56 ((CH_3_)_3_*C*), 34.5 ((CH_3_)_3_*C*), 34.4 ((*C*H_3_)_3_C), 34.3 ((*C*H_3_)_3_C), 33.2 ((*C*H_3_)_3_C), 33.1 ((*C*H_3_)_3_C), 33.0 ((*C*H_3_)_3_C) ppm. IR (KBr, cm^–1^): *v* 2958 (s), 1584 (s), 1560 (s), 1473 (s), 1389 (s), 1356 (s), 1237 (s), 1162 (s), 1058 (s), 1004 (s), 831 (s). Anal. calcd. for C_52_H_73_N_3_Th: C, 64.24; H, 7.57; N, 4.32. Found: C, 64.25; H, 7.56; N, 4.34.

#### Method B

##### NMR Scale

A C_6_D_6_ (0.3 mL) solution of PhCH_2_CN (2.4 mg, 0.02 mmol) was slowly added to a J. Young NMR tube charged with (Cp^3*t*Bu^)_2_Th(bipy) (**1**; 17.1 mg, 0.02 mmol) and C_6_D_6_ (0.2 mL). Resonances of **19** were observed by ^1^H NMR spectroscopy (100% conversion) when this solution was kept at room temperature overnight.

### Preparation of [(Cp^3*t*Bu^)_2_Th]_2_{μ-(bipy)[*p*-Ph(CN)_2_](bipy)} (**20**)

#### Method A

This compound was prepared as purple crystals from the reaction of (Cp^3*t*Bu^)_2_Th(bipy) (**1**; 214 mg, 0.25 mmol) and *p*-(NC)_2_Ph (16 mg, 0.125 mmol) in toluene (15 mL) at room temperature and recrystallization from a benzene solution by a similar procedure as that in the synthesis of **4**. The product was isolated by filtration, quickly washed with cold *n*-hexane (2 mL), and dried at room temperature under vacuum overnight. Yield: 198 mg (86%). M.p.: 262–264 °C (dec.). ^1^H NMR (C_6_D_6_): δ 8.19 (d, *J* = 4.1 Hz, 4H, phenyl), 7.72 (m, 2H, bipy), 7.32 (m, 2H, bipy), 6.67 (s, 2H, ring C*H*), 6.59 (t, *J* = 3.5 Hz, 2H, bipy), 6.51 (m, 2H, bipy), 6.42 (m, 4H, bipy and ring C*H*), 6.30 (s, 2H, ring C*H*), 6.11 (m, 2H, bipy), 6.03 (s, 2H, ring C*H*), 5.69 (m, 2H, bipy), 5.57 (m, 2H, bipy), 1.67 (s, 18H, C(C*H*_3_)_3_), 1.53 (s, 18H, C(C*H*_3_)_3_), 1.44 (s, 18H, C(C*H*_3_)_3_), 1.42 (s, 18H, C(C*H*_3_)_3_), 1.30 (s, 18H, C(C*H*_3_)_3_), 1.13 (s, 18H, C(C*H*_3_)_3_) ppm. ^13^C{^1^H} NMR (C_6_D_6_): δ 177.0 (bipy *C*), 164.7 (bipy *C*), 153.2 (bipy *C*), 147.2 (bipy *C*), 143.6 (bipy *C*), 143.1 (phenyl *C*), 142.8 (phenyl *C*), 142.7 (phenyl *C*), 142.3 (phenyl *C*), 140.4 (ring *C*), 138.3 (ring *C*), 137.8 (ring *C*), 137.4 (ring *C*), 132.3 (ring *C*), 132.0 (ring *C*), 125.3 (ring *C*), 124.9 (ring *C*), 121.9 (ring *C*), 119.6 (ring *C*), 119.4 (bipy *C*), 116.1 (bipy *C*), 114.1 (bipy *C*), 112.8 (bipy *C*), 102.5 (N=*C*), 79.6 (bipy *C*), 35.1 ((*C*H_3_)_3_C), 35.0 ((CH_3_)_3_*C*), 34.6 ((*C*H_3_)_3_C), 34.5 ((CH_3_)_3_*C*), 34.4 ((CH_3_)_3_*C*), 34.3 ((*C*H_3_)_3_C), 34.2 ((CH_3_)_3_*C*), 34.1 ((*C*H_3_)_3_C), 33.9 ((CH_3_)_3_*C*), 33.3 ((*C*H_3_)_3_C), 33.1 ((*C*H_3_)_3_C), 33.0 ((CH_3_)_3_*C*) ppm. IR (KBr, cm^–1^): *v* 2955 (s), 1581 (m), 1458 (m), 1427 (m), 1357 (m), 1257 (m), 1095 (m), 1018 (m), 802 (s). Anal. calcd. for C_96_H_136_N_6_Th_2_: C, 62.73; H, 7.46; N, 4.57. Found: C, 62.75; H, 7.44; N, 4.56.

#### Method B

##### NMR Scale

A C_6_D_6_ (0.3 mL) solution of *p*-(NC)_2_Ph (1.3 mg, 0.01 mmol) was slowly added to a J. Young NMR tube charged with (Cp^3*t*Bu^)_2_Th(bipy) (**1**; 17.1 mg, 0.02 mmol) and C_6_D_6_ (0.2 mL). Resonances of **20** were observed by ^1^H NMR spectroscopy (100% conversion) when this solution was kept at room temperature overnight.

### Preparation of (Cp^3*t*Bu^)_2_Th{(bipy)[PhC(CN)O]} (**21**)

#### Method A

This compound was prepared as orange crystals from the reaction of (Cp^3*t*Bu^)_2_Th(bipy) (**1**; 214 mg, 0.25 mmol) and PhCOCN (33 mg, 0.25 mmol) in toluene (15 mL) at room temperature and recrystallization from a benzene solution by a similar procedure as that in the synthesis of **4**. The product was isolated by filtration, quickly washed with cold *n*-hexane (2 mL), and dried at room temperature under vacuum overnight. Yield: 207 mg (84%). M.p.: 210–212 °C (dec.). ^1^H NMR (C_6_D_6_): δ 8.59 (d, *J* = 5.2 Hz, 1H, bipy), 7.75 (d, *J* = 2.8 Hz, 1H, bipy), 7.71 (d, *J* = 7.7 Hz, 2H, phenyl), 7.33 (d, *J* = 8.3 Hz, 1H, phenyl), 7.18 (t, *J* = 7.7 Hz, 2H, phenyl), 7.08 (t, *J* = 7.3 Hz, 1H, bipy), 6.82 (t, *J* = 7.7 Hz, 1H, bipy), 6.64 (d, *J* = 2.9 Hz, 1H, ring C*H*), 6.51 (t, *J* = 6.3 Hz, 1H, bipy), 6.38 (d, *J* = 2.6 Hz, 1H, ring C*H*), 6.22 (t, *J* = 6.7 Hz, 1H, bipy), 5.78 (s, 2H, ring C*H*), 5.71 (d, *J* = 5.7 Hz, 1H, bipy), 5.53 (d, *J* = 8.8 Hz, 1H, bipy), 1.84 (s, 9H, C(C*H*_3_)_3_), 1.43 (s, 9H, C(C*H*_3_)_3_), 1.40 (s, 9H, C(C*H*_3_)_3_), 1.31 (s, 9H, C(C*H*_3_)_3_), 1.20 (s, 9H, C(C*H*_3_)_3_), 0.92 (s, 9H, C(C*H*_3_)_3_) ppm. ^13^C{^1^H} NMR (C_6_D_6_): δ 163.6 (bipy *C*), 151.6 (bipy *C*), 149.3 (bipy *C*), 147.9 (bipy *C*), 147.3 (bipy *C*), 144.2 (phenyl *C*), 144.0 (phenyl *C*), 143.4 (phenyl *C*), 143.2 (phenyl *C*), 142.9 (ring *C*), 139.8 (ring *C*), 137.9 (ring *C*), 136.6 (ring *C*), 126.2 (ring *C*), 123.6 (ring *C*), 122.2 (ring *C*), 121.1 (ring *C*), 119.4 (ring *C*), 118.7 (ring *C*), 117.5 (bipy *C*), 116.1 (bipy *C*), 115.3 (bipy *C*), 114.0 (bipy *C*), 102.3 (*C*≡N), 88.9 (*C*O), 72.4 (bipy *C*), 35.7 ((CH_3_)_3_*C*), 35.5 ((*C*H_3_)_3_C), 35.2 ((*C*H_3_)_3_C), 34.74 ((*C*H_3_)_3_C), 34.72 ((*C*H_3_)_3_C), 34.6 ((CH_3_)_3_*C*), 34.2 ((*C*H_3_)_3_C), 34.1 ((*C*H_3_)_3_C), 34.0 ((CH_3_)_3_*C*), 33.8 ((CH_3_)_3_*C*), 33.5 ((CH_3_)_3_*C*), 32.7 ((CH_3_)_3_*C*) ppm. IR (KBr, cm^–1^): *v* 2957 (s), 1958 (w), 1604 (s), 1475 (s), 1359 (s), 1237 (s), 1165 (s), 1046 (s), 835 (s). Anal. calcd. for C_52_H_71_N_3_OTh: C, 63.33; H, 7.26; N, 4.26. Found: C, 63.35; H, 7.24; N, 4.28.

#### Method B

##### NMR Scale

A C_6_D_6_ (0.3 mL) solution of PhCOCN (2.6 mg, 0.02 mmol) was slowly added to a J. Young NMR tube charged with (Cp^3*t*Bu^)_2_Th(bipy) (**1**; 17.1 mg, 0.02 mmol) and C_6_D_6_ (0.2 mL). Resonances of **21** were observed by ^1^H NMR spectroscopy (100% conversion) when this solution was kept at room temperature overnight.

### Preparation of (Cp^3*t*Bu^)_2_Th[4-(Me_3_C)bipy](NC) (**22**)

#### Method A

This compound was prepared as yellow microcrystals from the reaction of (Cp^3*t*Bu^)_2_Th(bipy) (**1**; 214 mg, 0.25 mmol) and Me_3_CNC (21 mg, 0.25 mmol) in toluene (15 mL) at 120 °C and recrystallization from a benzene solution by a similar procedure as that in the synthesis of **4**. The product was isolated by filtration, quickly washed with cold *n*-hexane (2 mL), and dried at room temperature under vacuum overnight. Yield: 192 mg (82%). M.p.: 168–170 °C (dec.). ^1^H NMR (C_6_D_6_): δ 7.20 (d, *J* = 8.1 Hz, 1H, bipy), 7.15 (s, 1H, bipy), 6.94 (s, 1H, ring C*H*), 6.88 (s, 1H, ring C*H*), 6.83 (t, *J* = 7.7 Hz, 1H, bipy), 6.69 (s, 1H, bipy), 6.56 (s, 1H, ring C*H*), 6.52 (t, *J* = 6.5 Hz, 1H, bipy), 6.48 (s, 1H, ring C*H*), 5.32 (s, 1H, bipy), 4.80 (m, 1H, bipy), 3.58 (s, 1H, bipy), 1.52 (d, *J* = 3.1 Hz, 18H, C(C*H*_3_)_3_), 1.44 (s, 9H, C(C*H*_3_)_3_), 1.39 (d, *J* = 4.4 Hz, 18H, C(C*H*_3_)_3_), 1.24 (s, 9H, C(C*H*_3_)_3_), 1.00 (s, 9H, C(C*H*_3_)_3_) ppm. ^13^C{^1^H} NMR (C_6_D_6_): δ 161.7 (*C*N), 155.4 (bipy *C*), 147.5 (bipy *C*), 147.2 (bipy *C*), 146.8 (bipy *C*), 145.5 (bipy *C*), 144.3 (ring *C*), 143.7 (ring *C*), 143.4 (ring *C*), 138.1 (ring *C*), 132.6 (ring *C*), 128.3 (ring *C*), 127.9 (ring *C*), 126.7 (ring *C*), 122.1 (ring *C*), 119.3 (ring *C*), 117.9 (bipy *C*), 116.6 (bipy *C*), 104.8 (bipy *C*), 95.2 (bipy *C*), 45.6 (bipy *C*), 36.2 ((CH_3_)_3_*C*), 35.2 ((CH_3_)_3_*C*), 35.1 ((CH_3_)_3_*C*), 34.9 ((CH_3_)_3_*C*), 34.8 ((*C*H_3_)_3_C), 34.5 ((*C*H_3_)_3_C), 34.4 ((*C*H_3_)_3_C), 34.3 ((*C*H_3_)_3_C), 34.27 ((CH_3_)_3_*C*), 34.2 ((*C*H_3_)_3_C), 33.2 ((*C*H_3_)_3_C), 33.0 ((*C*H_3_)_3_C), 27.4 ((*C*H_3_)_3_C) ppm. IR (KBr, cm^–1^): *v* 2955 (s), 2052 (m, C≡N), 1589 (s), 1465 (s), 1373 (s), 1242 (s), 1010 (s), 841 (s), 794 (s). Anal. calcd. for C_49_H_75_N_3_Th: C, 62.73; H, 8.06; N, 4.48. Found: C, 62.75; H, 8.04; N, 4.46. Yellow crystals of **22**·0.5C_6_H_14_ suitable for X-ray structural analysis were isolated from a mixed benzene and *n*-hexane (4:1) solution.

#### Method B

##### NMR Scale

A C_6_D_6_ (0.3 mL) solution of Me_3_CNC (1.7 mg, 0.02 mmol) was slowly added to a J. Young NMR tube charged with (Cp^3*t*Bu^)_2_Th(bipy) (**1**; 17.1 mg, 0.02 mmol) and C_6_D_6_ (0.2 mL). Resonances of **22** were observed by ^1^H NMR spectroscopy (100% conversion) when this solution was kept at 120 °C overnight.

### Preparation of (Cp^3*t*Bu^)_2_Th[4-(C_6_H_11_)bipy](NC) (**23**)

#### Method A

This compound was prepared as yellow crystals from the reaction of (Cp^3*t*Bu^)_2_Th(bipy) (**1**; 214 mg, 0.25 mmol) and C_6_H_11_NC (28 mg, 0.25 mmol) in toluene (15 mL) at 120 °C and recrystallization from a benzene solution by a similar procedure as that in the synthesis of **4**. The product was isolated by filtration, quickly washed with cold *n*-hexane (2 mL), and dried at room temperature under vacuum overnight. Yield: 202 mg (84%). M.p.: 210–212 °C (dec.). ^1^H NMR (C_6_D_6_): δ 7.17 (d, *J* = 8.3 Hz, 1H, bipy), 7.15 (s, 1H, bipy), 6.92 (s, 2H, ring C*H*), 6.83 (t, *J* = 7.7 Hz, 1H, bipy), 6.69 (s, 1H, ring C*H*), 6.55 (s, 1H, ring C*H*), 6.50 (m, 2H, bipy), 5.16 (s, 1H, bipy), 4.66 (m, 1H, bipy), 3.66 (s, 1H, bipy), 1.86 (m, 4H, Cy), 1.72 (d, *J* = 12.3 Hz, 1H, Cy), 1.54 (s, 9H, C(C*H*_3_)_3_), 1.52 (s, 9H, C(C*H*_3_)_3_), 1.46 (s, 9H, C(C*H*_3_)_3_), 1.39 (s, 18H, C(C*H*_3_)_3_), 1.31 (m, 4H, Cy), 1.25 (s, 9H, C(C*H*_3_)_3_), 1.20 (m, 2H, Cy) ppm. ^13^C{^1^H} NMR (C_6_D_6_): δ 161.6 (*C*N), 155.2 (bipy *C*), 147.4 (bipy *C*), 146.8 (bipy *C*), 146.6 (bipy *C*), 145.3 (bipy *C*), 144.3 (ring *C*), 143.6 (ring *C*), 143.2 (ring *C*), 138.0 (ring *C*), 132.0 (ring *C*), 128.3 (ring *C*), 128.1 (ring *C*), 127.9 (ring *C*), 122.0 (ring *C*), 119.2 (ring *C*), 118.0 (bipy *C*), 116.6 (bipy *C*), 106.7 (bipy *C*), 96.5 (bipy *C*), 47.4 (bipy *C*), 40.4 (Cy *C*), 35.2 ((CH_3_)_3_*C*), 35.14 ((CH_3_)_3_*C*), 35.11 ((CH_3_)_3_*C*), 34.9 ((CH_3_)_3_*C*), 34.8 ((CH_3_)_3_*C*), 34.4 ((*C*H_3_)_3_C), 34.3 ((*C*H_3_)_3_C), 34.29 ((*C*H_3_)_3_C), 33.2 ((*C*H_3_)_3_C), 33.1 ((*C*H_3_)_3_C), 30.54 (Cy *C*), 30.46 (Cy *C*), 27.3 ((*C*H_3_)_3_C), 27.2 ((CH_3_)_3_*C*) ppm. IR (KBr, cm^–1^): *v* 2939 (s), 2052 (m; C≡N), 1643 (m), 1581 (s), 1465 (s), 1373 (s), 1242 (s), 1010 (s), 794 (s). Anal. calcd. for C_51_H_77_N_3_Th: C, 63.53; H, 8.05; N, 4.36. Found: C, 63.55; H, 8.06; N, 4.34.

#### Method B

##### NMR Scale

A C_6_D_6_ (0.3 mL) solution of C_6_H_11_NC (2.2 mg, 0.02 mmol) was slowly added to a J. Young NMR tube charged with (Cp^3*t*Bu^)_2_Th(bipy) (**1**; 17.1 mg, 0.02 mmol) and C_6_D_6_ (0.2 mL). Resonances of **23** were observed by ^1^H NMR spectroscopy (100% conversion) when this solution was kept at 120 °C overnight.

### X-ray Crystallography

Single-crystal X-ray diffraction measurements were carried out on a Rigaku Saturn CCD diffractometer at 100(2) K using Mο Kα radiation (λ = 0.71073 Å) or Cu Kα radiation (λ = 1.54184 Å). An empirical absorption correction was applied using the SADABS program.^163^ All structures were solved by direct methods and refined by full-matrix least-squares on *F*^2^ using the SHELXL program package.^164^ The hydrogen atom (N–H) in **19** was located in the difference Fourier map and refined isotropically. Other hydrogen atoms were geometrically fixed using the riding model. The crystal data and experimental data for **2**–**23** are summarized in the Supporting Information. Selected bond lengths and angles are listed in Table 2.

## Supporting Information Available

Crystal parameters for compounds **2**–**23** and NMR spectra (PDF)
Computational details and Cartesian coordinates of all stationary points optimized at the B3PW91-PCM level (XYZ)

## Supplementary Material

ic4c02782_si_001.pdf

ic4c02782_si_002.xyz
