# Supplementary material for: Influence of 1,2,4-Tri-tert-butylcyclopentadienyl Ligand on the Reactivity of the Thorium Bipyridyl Metallocene [η5-1,2,4-(Me3C)3C5H2]2Th(bipy)]
Source: Inorg Chem. 2024 Oct 3;63(41):19188–212. doi: 10.1021/acs.inorgchem.4c02782 (PMC11483809; doi:10.1021/acs.inorgchem.4c02782)
Supplement: Supplementary file 1 — ic4c02782_si_001.pdf [file ic4c02782_si_001.pdf]

Supporting Information for

Influence of the 1,2,4-Tri-*tert*-butylcyclopentadienyl Ligand on the Reactivity of the Thorium Bipyridyl Metallocene [η^5-1,2,4-$(Me_3C)_3C_5H_2]_2$Th(bipy)

Dongwei Wang,[†] Yi Heng,[†] Tongyu Li,[†] Wanjian Ding,[†] Guohua Hou,[†] Guofu Zi,*[,†] and Marc D. Walter*[,‡]

[†]Department of Chemistry, Beijing Normal University, Beijing 100875, China

[‡]Institut für Anorganische und Analytische Chemie, Technische Universität Braunschweig, Hagenring 30, 38106 Braunschweig, Germany

*Corresponding authors. E-mail: gzi@bnu.edu.cn (G.Z.), mwalter@tu-bs.de (M.D.W.)

Table of contents

1. **Figures** **S2**
2. **Crystallographic details** **S5**
3. **Computational details** **S10**
4. **NMR spectra** **S30**
5. **References** **S53**

1. Figures

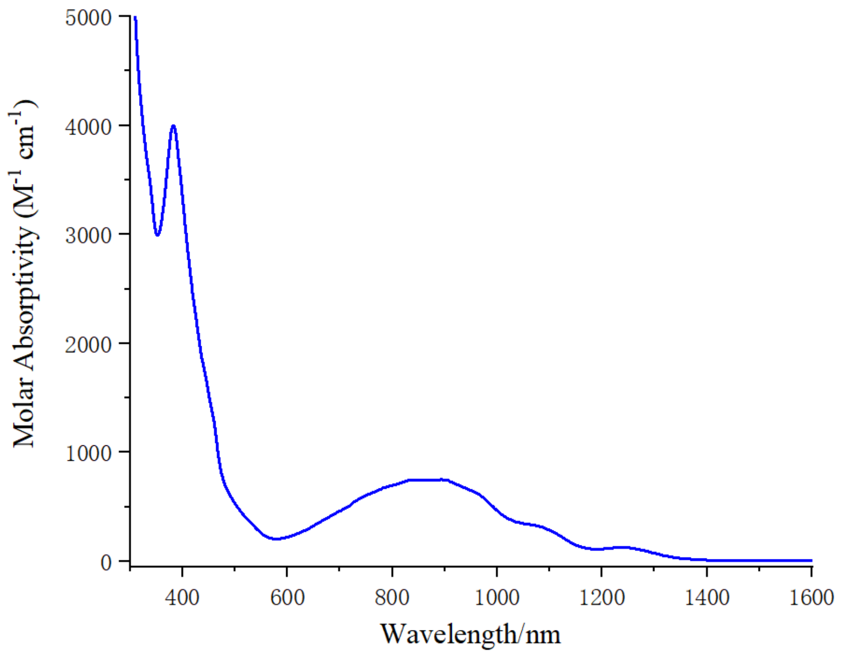

Figure S1. UV-Vis spectrum of **3** in toluene.

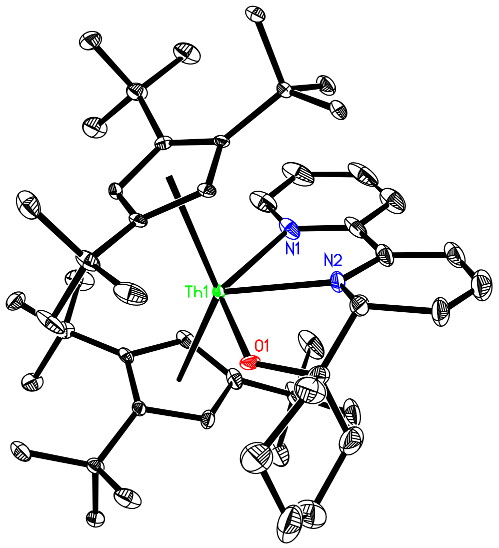

Figure S2. Molecular structure of 11 (thermal ellipsoids drawn at the 35% probability level).

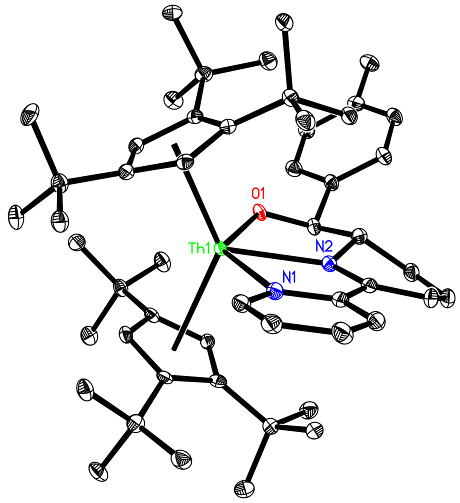

Figure S3. Molecular structure of 12 (thermal ellipsoids drawn at the 35% probability level).

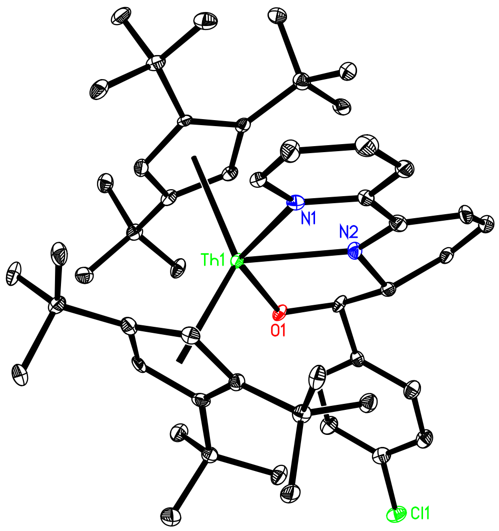

Figure S4. Molecular structure of 13 (thermal ellipsoids drawn at the 35% probability level).

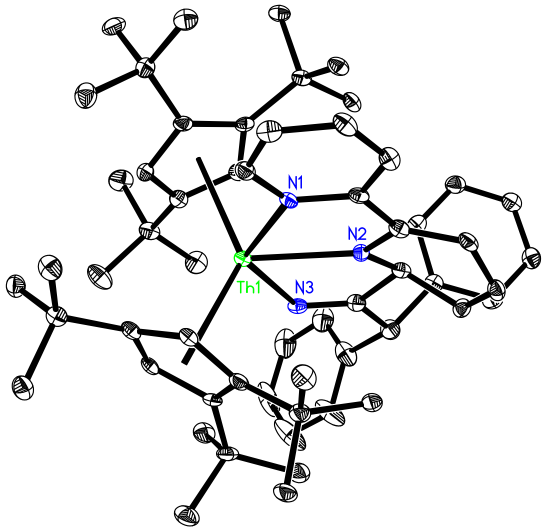

Figure S5. Molecular structure of 17 (thermal ellipsoids drawn at the 35% probability level).

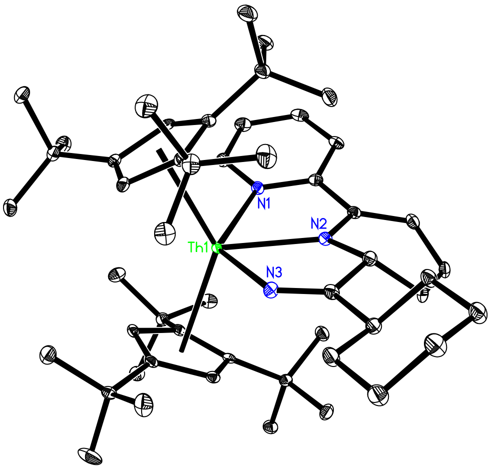

Figure S6. Molecular structure of 18 (thermal ellipsoids drawn at the 35% probability level).

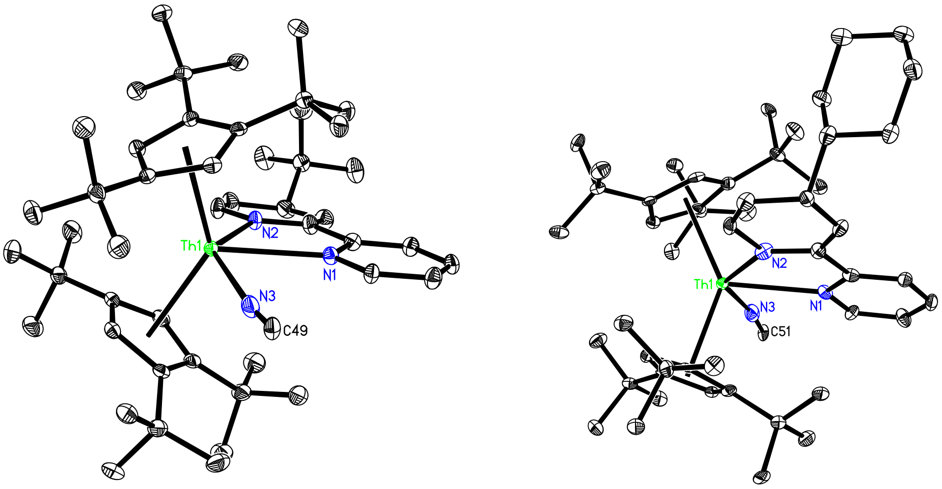

Figure S7. Molecular structures of 22 (left) and 23 (right) (thermal ellipsoids drawn at the 35% probability level).

2. Crystallographic details

Table S1. Crystal Data and Experimental Parameters for Compounds 2-6

Compound	**2**	**3**	**4**	**5** C_6H_6	**6**
Formula	$C_{34}H_{58}I_2Th$	$C_{44}H_{66}IN_2Th$	$C_{46}H_{70}N_2Th$	$C_{51}H_{58}N_2S_3Th$	$C_{41}H_{68}N_2S_2Th$
Fw	952.64	981.92	883.08	1027.21	885.13
crystal system	triclinic	triclinic	monoclinic	monoclinic	triclinic
space group	$P\bar{1}$	$P\bar{1}$	$P2_1$	$P2_1/c$	$P\bar{1}$
a (Å)	11.045(1)	10.335(1)	10.443(1)	13.170(1)	10.445(1)
b (Å)	17.744(1)	10.957(1)	10.000(1)	22.905(1)	12.418(1)
c (Å)	19.984(1)	20.880(1)	20.049(1)	31.454(1)	19.289(1)
α (deg)	69.76(1)	80.54(1)	90	90	71.76(1)
β (deg)	88.83(1)	84.49(1)	98.53(1)	101.08(1)	76.90(1)
γ (deg)	80.85(1)	62.17(1)	90	90	81.87(1)
V (Å^3)	3625.5(3)	2061.99(11)	2070.58(10)	9311.2(5)	2307.4(3)
Z	4	2	2	8	2
D_{calc} (g/cm^3)	1.745	1.582	1.416	1.466	1.274
μ(Mo/Kα)$_{calc}$ (cm^{-1})	5.835	17.754	11.835	11.846	11.443
size (mm)	0.20 × 0.15 × 0.10	0.15 × 0.10 × 0.10	0.10 × 0.10 × 0.10	0.15 × 0.15 × 0.15	0.15 × 0.10 × 0.10
F(000)	1832	974	900	4128	900
2θ range (deg)	6.65 to 59.60	8.59 to 153.27	8.56 to 152.84	6.84 to 154.52	7.52 to 159.53
no. of reflns, collected	36617	27566	15082	76946	26571
no of obsd reflns	17344	8214	7207	18668	9107
no of variables	704	451	460	1045	435
abscorr (T_{max}, T_{min})	1.00, 0.82	1.00, 0.74	1.00, 0.70	1.00, 0.76	1.00, 0.41
R	0.049	0.035	0.039	0.049	0.081
R_w	0.099	0.084	0.107	0.125	0.213
R_{all}	0.068	0.039	0.042	0.070	0.102
Gof	1.04	1.06	1.10	1.05	1.14
CCDC	2363739	2363745	2363733	2363736	2363732

Table S2. Crystal Data and Experimental Parameters for Compounds 7-11

Compound	**7**	**8**	**9**	**10**	**11**
Formula	$C_{68}H_{116}Se_{11}Th_4$	$C_{70}H_{116}S_4Th_2$	$C_{82}H_{126}N_2S_4Th_2$	$C_{57}H_{76}N_2OTh$	$C_{50}H_{76}N_2OTh$
Fw	2730.32	1549.94	1732.16	1037.23	953.16
crystal system	monoclinic	triclinic	triclinic	orthorhombic	monoclinic
space group	$P2/n$	$P\bar{1}$	$P\bar{1}$	$P2_12_12_1$	$P2_1/c$
a (Å)	13.594(1)	10.288(1)	10.052(1)	12.446(1)	18.896(1)
b (Å)	14.519(1)	11.740(1)	13.565(1)	16.763(1)	12.966(1)
c (Å)	22.683(1)	16.480(1)	16.721(1)	23.878(1)	19.097(1)
α (deg)	90	69.46(1)	99.51(1)	90	90
β (deg)	93.62(1)	78.55(1)	101.07(1)	90	105.32(1)
γ (deg)	90	68.22(1)	107.24(1)	90	90
V (Å^3)	4467.9(5)	1725.50(18)	2076.16(12)	4981.84(15)	4512.65(10)
Z	2	1	1	4	4
D_{calc} (g/cm^3)	2.029	1.492	1.385	1.383	1.403
μ(Mo/Kα)$_{calc}$ (cm^{-1})	11.149	15.198	12.700	9.942	10.920
size (mm)	0.20 × 0.20 × 0.20	0.10 × 0.10 × 0.10	0.20 × 0.20 × 0.10	0.10 × 0.10 × 0.10	0.10 × 0.10 × 0.10
F(000)	2516	780	876	2120	1952
2θ range (deg)	6.67 to 59.70	8.52 to 154.79	7.02 to 153.22	6.44 to 152.36	8.34 to 153.08
no. of reflns, collected	10890	19389	26172	19662	34475
no of obsd reflns	10890	6834	8330	9038	9114
no of variables	395	361	425	568	505
abscorr (T_{max}, T_{min})	1.00, 0.47	1.00, 0.59	1.00, 0.42	1.00, 0.90	1.00, 0.66
R	0.069	0.057	0.055	0.060	0.042
R_w	0.134	0.130	0.139	0.142	0.101
R_{all}	0.120	0.070	0.063	0.070	0.048
Gof	1.02	1.13	1.05	1.02	1.07
CCDC	2363735	2363730	2363731	2363738	2363741

Table S3. Crystal Data and Experimental Parameters for Compounds 12-15

Compound	**12** $0.5C_6H_{14}$	**13** $0.5C_6H_{14}$	**14**	**15**
Formula	$C_{55}H_{81}N_2OTh$	$C_{54}H_{78}ClN_2OTh$	$C_{59}H_{80}N_2O_2SeTh$	$C_{68}H_{82}N_6Th_2$
Fw	1018.25	1038.67	1160.25	1447.47
crystal system	monoclinic	monoclinic	triclinic	triclinic
space group	*I*2/*a*	*I*2/*a*	$P\bar{1}$	$P\bar{1}$
a (Å)	17.411(1)	17.394(1)	16.261(1)	10.745(1)
b (Å)	13.418(1)	13.397(1)	18.700(1)	14.692(1)
c (Å)	42.839(1)	42.811(1)	21.194(1)	21.899(1)
α (deg)	90	90	112.69(1)	73.19(1)
β (deg)	96.70(1)	96.62(1)	101.67(1)	76.96(1)
γ (deg)	90	90	90.08(1)	86.60(1)
V (Å^3)	9939.6(5)	9909.4(5)	5800.8(3)	3224.02(9)
Z	8	8	4	2
D_{calc} (g/cm^3)	1.361	1.392	1.329	1.491
μ(Mo/Kα)$_{calc}$ (cm^{-1})	9.952	10.479	9.296	15.087
size (mm)	0.10 × 0.10 × 0.10	0.10 × 0.10 × 0.10	0.10 × 0.10 × 0.10	0.20 × 0.20 × 0.10
F(000)	4184	4248	2352	1424
2θ range (deg)	6.91 to 152.99	8.32 to 153.39	7.24 to 153.11	8.61 to 153.11
no. of reflns, collected	35347	35093	76811	43599
no of obsd reflns	9924	9889	23335	13080
no of variables	552	551	1210	703
abscorr (T_{max}, T_{min})	1.00, 0.84	1.00, 0.90	1.00, 0.61	1.00, 0.35
R	0.039	0.043	0.088	0.037
R_w	0.088	0.094	0.235	0.094
R_{all}	0.051	0.058	0.103	0.042
Gof	1.06	1.04	1.04	1.13
CCDC	2363734	2363750	2363751	2363737

Table S4. Crystal Data and Experimental Parameters for Compounds 16-19

Compound	**16**	**17**	**18** 0.5C_6H_6	**19**
Formula	$C_{51}H_{71}N_3Th$	$C_{58}H_{77}N_3Th$	$C_{54}H_{80}N_3Th$	$C_{52}H_{73}N_3Th$
Fw	958.14	1048.26	1003.25	972.17
crystal system	monoclinic	triclinic	triclinic	monoclinic
space group	$P2_1/n$	$P\bar{1}$	$P\bar{1}$	$P2_1/c$
a (Å)	11.110(1)	11.772(1)	11.175(1)	11.241(1)
b (Å)	37.018(1)	13.394(1)	12.260(1)	20.778(1)
c (Å)	11.841(1)	18.291(1)	19.802(1)	20.177(1)
α (deg)	90	77.25(1)	102.36(1)	90
β (deg)	108.76(1)	83.53(1)	101.31(1)	98.75(1)
γ (deg)	90	64.71(1)	105.29(1)	90
V (Å^3)	4611.32(9)	2542.90(14)	2462.57(6)	4657.78(5)
Z	4	2	2	4
D_{calc} (g/cm^3)	1.380	1.369	1.353	1.386
μ(Mo/Kα)$_{calc}$ (cm^{-1})	10.682	9.736	10.024	10.584
size (mm)	0.15 × 0.15 × 0.15	0.10 × 0.10 × 0.10	0.20 × 0.20 × 0.20	0.10 × 0.10 × 0.10
F(000)	1952	1072	1030	1984
2θ range (deg)	8.24 to 153.20	7.44 to 152.16	7.79 to 152.59	7.96 to 153.02
no. of reflns, collected	32388	33227	32582	36383
no of obsd reflns	9289	10223	9907	9391
no of variables	514	577	541	528
abscorr (T_{max}, T_{min})	1.00, 0.84	1.00, 0.79	1.00, 0.16	1.00, 0.72
R	0.058	0.047	0.031	0.033
R_w	0.126	0.098	0.076	0.083
R_{all}	0.062	0.057	0.032	0.037
Gof	1.28	1.06	1.06	1.07
CCDC	2363744	2363742	2363743	2363740

Table S5. Crystal Data and Experimental Parameters for Compounds 20-23

Compound	**20**	**21**	**22** 0.5C_6H_{14}	**23**
Formula	$C_{96}H_{136}N_6Th_2$	$C_{52}H_{71}N_3OTh$	$C_{52}H_{82}N_3Th$	$C_{51}H_{77}N_3Th$
Fw	1838.18	986.15	981.24	964.19
crystal system	monoclinic	monoclinic	monoclinic	orthorhombic
space group	$P2_1/n$	$P2_1/c$	$P2_1/c$	$Pna2_1$
a (Å)	17.830(1)	10.858(1)	17.933(1)	25.908(1)
b (Å)	20.920(1)	22.625(1)	16.597(1)	13.343(1)
c (Å)	28.572(1)	20.601(1)	17.008(1)	13.479(1)
α (deg)	90	90	90	90
β (deg)	106.84(1)	101.38(1)	101.54(1)	90
γ (deg)	90	90	90	90
V (Å^3)	10200.07(19)	4961.2(3)	4959.65(10)	4659.6(2)
Z	4	4	4	4
D_{calc} (g/cm^3)	1.197	1.320	1.314	1.374
μ(Mo/Kα)$_{calc}$ (cm^{-1})	9.637	9.961	9.940	10.572
size (mm)	0.15 × 0.15 × 0.15	0.10 × 0.10 × 0.10	0.10 × 0.10 × 0.10	0.15 × 0.15 × 0.15
F(000)	3736	2008	2020	1976
2θ range (deg)	6.46 to 153.01	7.82 to 152.99	7.33 to 152.92	6.82 to 153.30
no. of reflns, collected	88283	36780	38425	34589
no of obsd reflns	20646	9900	9998	7020
no of variables	973	532	527	514
abscorr (T_{max}, T_{min})	1.00, 0.77	1.00, 0.69	1.00, 0.86	1.00, 0.70
R	0.038	0.057	0.040	0.046
R_w	0.091	0.150	0.102	0.113
R_{all}	0.044	0.071	0.045	0.054
Gof	1.04	1.09	1.09	1.02
CCDC	2363747	2363749	2363746	2363748

3. Computational details

Calculations were performed with the Gaussian 09 program (G09),[1] employing the B3PW91 functional, plus a polarizable continuum model (PCM) (denoted as B3PW91-PCM), with a standard 6-31G(d) basis set for the elements C, H, N, S and Si and a quasi-relativistic 5f-in-valence effective-core potential (ECP60MWB) treatment with 60 electrons in the core region for Th and the corresponding optimized segmented ((14s13p10d8f6g)/[10s9p5d4f3g]) basis set for the valence shells of Th,[2-4] to fully optimize the structures, and to also account for the experimental reaction conditions using toluene as a solvent (dielectric constant ε = 2.379). All stationary points were subsequently characterized by vibrational analyses, from which their respective zero-point (vibrational) energy (ZPE) were extracted and used in the relative energy determinations. In addition, frequency calculations were also performed to ensure that the determined structures resided at minima and 1st order saddle points, respectively, on their potential energy hypersurfaces.

Table S6. The optimized Cartesian Coordinates (in Å) and structures (the hydrogen atoms omitted for clarity) of stationary points for some stationary points, obtained with B3PW91-PCM/6-31G(d)/ECP60MWB method.

Species	Cartesian coordinates				
**1**	C	5.079516	13.784901	8.367300	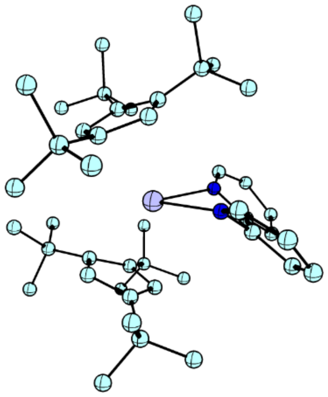 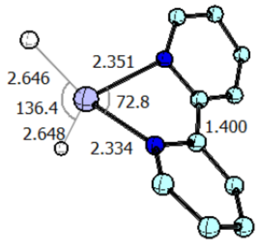
	H	6.027343	13.565199	7.886301	
	C	5.026206	14.581316	9.469713	
	H	5.939679	14.973118	9.904981	
	C	3.735806	14.939935	9.987400	
	H	3.652378	15.662439	10.795098	
	C	2.621970	14.363599	9.460675	
	H	1.644949	14.614404	9.861528	
	C	2.733864	13.363822	8.424370	
	C	1.711007	12.459175	8.115065	
	C	0.429777	12.455499	8.783345	
	H	0.145431	13.304097	9.397062	
	C	-0.390703	11.373640	8.706723	
	H	-1.335204	11.361411	9.244559	
	C	-0.009014	10.252966	7.895441	
	H	-0.626805	9.363724	7.828320	
	C	1.112901	10.368945	7.131842	
	H	1.373514	9.597361	6.414279	
	C	1.045469	13.675568	4.207847	
	C	2.072249	13.751925	3.187865	
	C	3.061478	14.655409	3.683160	
	H	3.917643	14.992875	3.112343	
	C	2.672944	15.207767	4.926241	
	C	1.464933	14.549399	5.262656	
	H	0.892210	14.742142	6.159355	
	C	-0.382803	13.086709	4.203252	
	C	-1.108424	13.414499	5.521726	
	H	-2.100666	12.947605	5.508583	
	H	-1.255065	14.493252	5.649340	
	H	-0.575009	13.027925	6.394548	
	C	-1.227137	13.752096	3.095084	
	H	-2.275580	13.442744	3.193796	
	H	-0.903708	13.485306	2.087644	
	H	-1.189010	14.844095	3.183186	
	C	-0.434438	11.554141	4.058111	
	H	-0.075868	11.069987	4.972429	
	H	0.151820	11.181242	3.218138	

	H -1.471771 11.226555 3.908394<br>C 2.114248 13.395209 1.682646<br>C 1.517128 14.592095 0.904411<br>H 1.553595 14.398961 -0.175870<br>H 2.087718 15.505310 1.106022<br>H 0.476753 14.783586 1.179041<br>C 1.379076 12.118045 1.251771<br>H 1.537434 11.956705 0.178346<br>H 0.301444 12.167232 1.410532<br>H 1.758574 11.232182 1.771906<br>C 3.565840 13.233842 1.188898<br>H 4.085803 12.436395 1.726516<br>H 4.151813 14.151157 1.292857<br>H 3.562348 12.974824 0.123457<br>C 3.216903 16.506442 5.522054<br>C 2.942466 17.627198 4.494366<br>H 1.873733 17.689343 4.259526<br>H 3.483772 17.453785 3.557400<br>H 3.261697 18.598072 4.895329<br>C 4.728002 16.447581 5.782127<br>H 5.099821 17.429235 6.103039<br>H 5.281138 16.167771 4.877837<br>H 4.961645 15.729365 6.575606<br>C 2.496467 16.872477 6.824340<br>H 1.420487 17.004907 6.660077<br>H 2.890285 17.819417 7.214041<br>H 2.637856 16.110042 7.595818<br>C 4.726548 10.023195 4.686329<br>C 5.114063 10.161827 6.074326<br>C 5.930668 11.334423 6.139107<br>H 6.437130 11.659579 7.037252<br>C 6.115207 11.905595 4.861722<br>C 5.310978 11.132678 3.991821<br>H 5.274944 11.264437 2.919802<br>C 4.120078 8.851998 3.880796<br>C 5.036768 7.616023 4.003693<br>H 4.666479 6.809227 3.358252<br>H 5.092797 7.225486 5.020571<br>H 6.055508 7.863899 3.684280<br>C 2.679231 8.484881 4.272000<br>H 1.994179 9.316823 4.066227<br>H 2.582460 8.214761 5.323070<br>H 2.334408 7.629143 3.677613<br>C 4.082135 9.180528 2.375241<br>H 5.085245 9.327021 1.960709<br>H 3.487594 10.073674 2.158751<br>H 3.622991 8.344037 1.835778<br>C 5.118036 9.234294 7.313887<br>C 6.484953 8.509820 7.334538<br>H 6.553831 7.856794 8.214263<br>H 7.309639 9.229552 7.381918<br>H 6.633654 7.892696 6.442595<br>C 4.013662 8.171799 7.402859<br>H 4.151498 7.600070 8.328678<br>H 4.033352 7.451733 6.583177<br>H 3.021340 8.626681 7.452659	

	C 5.004751 10.054730 8.616028<br>H 4.070317 10.621872 8.647412<br>H 5.830785 10.759214 8.749594<br>H 5.025273 9.371947 9.474398<br>C 7.232220 12.859319 4.441950<br>C 7.668425 13.787161 5.583048<br>H 8.505558 14.415599 5.255465<br>H 8.010865 13.220714 6.456618<br>H 6.859503 14.453645 5.898671<br>C 6.848442 13.711360 3.225142<br>H 6.012309 14.380359 3.452925<br>H 6.563173 13.092154 2.368135<br>H 7.698949 14.330988 2.915584<br>C 8.441026 11.978083 4.052027<br>H 9.288523 12.603740 3.743057<br>H 8.188499 11.310069 3.220998<br>H 8.762600 11.359017 4.897042<br>N 3.976565 13.259284 7.739968<br>N 1.949319 11.455770 7.136311<br>Th 3.386331 12.516874 5.607496	
**1′**	Th -2.984674 17.440348 5.754957<br>Si -5.816799 18.950549 2.744928<br>Si -5.441187 14.973083 3.164055<br>Si -6.575868 17.392097 8.197228<br>Si -2.779105 21.890046 6.222740<br>Si 0.352022 20.014436 4.700041<br>Si -1.224707 17.662180 9.709357<br>N -2.585056 15.259908 6.397893<br>N -1.907155 16.420466 3.936612<br>C -5.433112 17.866248 4.248713<br>C -5.334644 16.428506 4.375966<br>C -5.512680 16.135877 5.756815<br>H -5.537290 15.130347 6.163795<br>C -5.726125 17.320112 6.515940<br>C -5.646876 18.378611 5.565621<br>H -5.809019 19.426038 5.798275<br>C -6.301760 20.675123 3.353963<br>H -7.182595 20.641214 4.005120<br>H -6.553408 21.304838 2.491400<br>H -5.497614 21.179556 3.898457<br>C -7.337703 18.265471 1.846959<br>H -7.167321 17.288305 1.385319<br>H -7.640486 18.959287 1.052376<br>H -8.180300 18.164979 2.541035<br>C -4.409812 19.149263 1.495794<br>H -3.504444 19.569069 1.949739<br>H -4.722351 19.832210 0.695841<br>H -4.142091 18.196917 1.027134<br>C -7.274046 14.486891 3.081673<br>H -7.654631 14.236211 4.078977<br>H -7.413516 13.608024 2.439491<br>H -7.897193 15.295905 2.685032<br>C -4.837791 15.284055 1.398832<br>H -5.271412 16.175896 0.937280<br>H -5.130227 14.424130 0.782368<br>H -3.747464 15.365923 1.350141	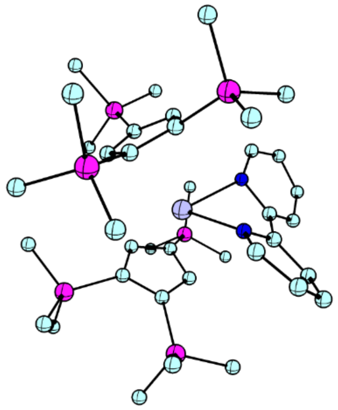<br>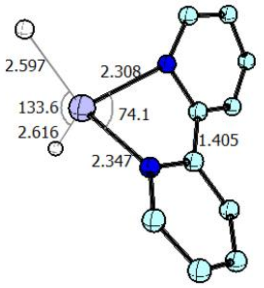

	C	-4.481391	13.495591	3.845351		
	H	-3.414171	13.719862	3.940513		
	H	-4.593682	12.639652	3.167830		
	H	-4.843784	13.183748	4.831293		
	C	-8.418245	17.670027	7.856780		
	H	-8.584479	18.619559	7.334637		
	H	-8.991800	17.696073	8.791697		
	H	-8.829627	16.870431	7.230160		
	C	-6.369849	15.747886	9.105480		
	H	-6.780973	14.916583	8.520601		
	H	-6.910938	15.774916	10.059315		
	H	-5.321728	15.521107	9.327219		
	C	-5.970571	18.819991	9.276169		
	H	-4.932413	18.690635	9.598329		
	H	-6.591333	18.896158	10.177599		
	H	-6.043091	19.776710	8.746279		
	C	-2.097464	20.152118	6.519855		
	C	-0.921010	19.482535	5.998764		
	C	-0.616202	18.434514	6.919091		
	H	0.238908	17.772396	6.831672		
	C	-1.537659	18.420894	8.009709		
	C	-2.450129	19.478272	7.722404		
	H	-3.268349	19.775879	8.371387		
	C	-1.756227	23.095035	7.268802		
	H	-1.855765	22.851319	8.333292		
	H	-2.108165	24.124762	7.126818		
	H	-0.691036	23.064868	7.021398		
	C	-2.757846	22.461264	4.419054		
	H	-1.744789	22.587195	4.025275		
	H	-3.260444	23.434076	4.343816		
	H	-3.288614	21.765805	3.760110		
	C	-4.557263	22.036692	6.860448		
	H	-5.287272	21.536156	6.216666		
	H	-4.832987	23.098408	6.898566		
	H	-4.666447	21.636654	7.874460		
	C	1.045859	21.731093	5.115597		
	H	0.325531	22.543667	4.983126		
	H	1.900010	21.938879	4.458090		
	H	1.407712	21.766095	6.150019		
	C	-0.286107	20.034498	2.920633		
	H	-0.513223	19.020927	2.571651		
	H	0.487779	20.443525	2.257607		
	H	-1.184084	20.647870	2.795978		
	C	1.829256	18.838005	4.795768		
	H	2.305315	18.872253	5.782900		
	H	2.581261	19.141908	4.056580		
	H	1.554238	17.800845	4.578775		
	C	-2.547463	16.446159	10.290835		
	H	-3.545905	16.897227	10.283788		
	H	-2.337973	16.132335	11.321562		
	H	-2.572320	15.548024	9.664375		
	C	0.459436	16.808794	9.700809		
	H	0.474998	15.969224	8.997360		
	H	0.689358	16.414736	10.698334		
	H	1.259297	17.505477	9.423990		
	C	-1.179906	19.104712	10.938523		

	H -0.403833 19.830432 10.668992<br>H -0.969337 18.746017 11.953811<br>H -2.137714 19.637803 10.966585<br>C -3.008432 14.296953 7.276598<br>H -4.071300 14.317739 7.503764<br>C -2.173535 13.415131 7.893338<br>H -2.567643 12.678473 8.585579<br>C -0.760985 13.558275 7.676308<br>H -0.059342 12.974459 8.266692<br>C -0.307404 14.430303 6.735946<br>H 0.759744 14.538893 6.566114<br>C -1.250102 15.161308 5.915776<br>C -0.926451 15.695230 4.656647<br>C 0.360655 15.502677 4.030369<br>H 1.177285 15.078268 4.604781<br>C 0.522178 15.745357 2.700720<br>H 1.474928 15.537752 2.220288<br>C -0.567394 16.273115 1.935324<br>H -0.474182 16.460201 0.870868<br>C -1.709448 16.615514 2.597619<br>H -2.509902 17.135636 2.080693	
**8**	C 8.198722 11.431857 10.733669<br>C 7.235744 11.421488 11.819097<br>C 6.007287 10.939258 11.262871<br>H 5.071308 10.898315 11.806210<br>C 6.136787 10.714484 9.871394<br>C 7.498324 10.959606 9.579707<br>H 7.938462 10.854351 8.597515<br>C 9.617985 12.030421 10.583297<br>C 9.584870 13.550696 10.849039<br>H 8.821807 14.035727 10.229081<br>H 10.556758 13.988989 10.589254<br>H 9.383821 13.804919 11.891399<br>C 10.117563 11.878408 9.131956<br>H 10.167252 10.831138 8.819985<br>H 11.130854 12.291745 9.062032<br>H 9.488708 12.430413 8.424496<br>C 10.684660 11.363439 11.470622<br>H 10.436287 11.383240 12.532514<br>H 11.645157 11.880637 11.348390<br>H 10.834672 10.322623 11.163806<br>C 7.184806 12.107915 13.203069<br>C 6.679431 13.554737 12.990217<br>H 7.351098 14.135597 12.353318<br>H 6.594090 14.071792 13.955029<br>H 5.691202 13.552019 12.517250<br>C 6.159797 11.420435 14.128057<br>H 5.136186 11.490094 13.748540<br>H 6.169666 11.907552 15.110192<br>H 6.393047 10.362232 14.277606<br>C 8.504882 12.160456 13.985239<br>H 9.267854 12.763460 13.492541<br>H 8.921073 11.162010 14.153876<br>H 8.325575 12.610187 14.969354<br>C 4.987334 10.613346 8.869706<br>C 4.024581 9.461328 9.187840	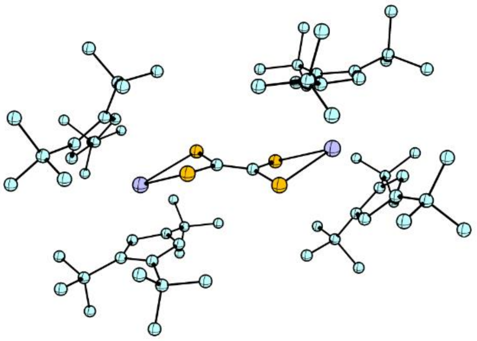 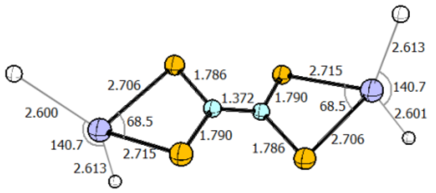

	H 4.523777 8.494599 9.066734<br>H 3.166949 9.481006 8.503518<br>H 3.630327 9.533076 10.208261<br>C 4.203001 11.942127 8.955023<br>H 3.759383 12.084720 9.947170<br>H 3.391505 11.951411 8.215914<br>H 4.858086 12.797148 8.751766<br>C 5.506361 10.456557 7.435391<br>H 6.113689 11.318619 7.136306<br>H 4.659754 10.391276 6.741041<br>H 6.109532 9.551406 7.318569<br>C 8.221327 7.042370 13.489375<br>C 8.194314 6.058513 12.428309<br>C 6.838763 6.009645 11.972274<br>H 6.481946 5.338695 11.203886<br>C 6.015892 6.874850 12.725491<br>C 6.888982 7.544309 13.617643<br>H 6.568176 8.240595 14.380630<br>C 9.324665 7.482196 14.474007<br>C 10.464811 8.232786 13.761901<br>H 10.910599 7.652030 12.952509<br>H 11.259857 8.492697 14.472600<br>H 10.103973 9.176893 13.329685<br>C 8.753901 8.436663 15.540996<br>H 8.322227 9.344021 15.108664<br>H 9.560772 8.749941 16.213694<br>H 7.984264 7.949083 16.149848<br>C 9.882426 6.287489 15.273012<br>H 9.072019 5.754613 15.783603<br>H 10.579972 6.651509 16.037876<br>H 10.421664 5.568547 14.657172<br>C 9.159694 4.927974 11.997943<br>C 10.656171 5.274794 11.953925<br>H 11.067336 5.569912 12.920123<br>H 10.852012 6.071870 11.230138<br>H 11.214658 4.387746 11.630278<br>C 8.937341 3.743295 12.968742<br>H 9.187942 3.994659 14.002573<br>H 9.560427 2.891153 12.667163<br>H 7.890190 3.419729 12.948646<br>C 8.810027 4.411531 10.587133<br>H 9.532222 3.636470 10.303743<br>H 8.852766 5.211146 9.843343<br>H 7.817365 3.952437 10.540867<br>C 4.493492 6.830439 12.813640<br>C 3.891391 8.207494 13.126518<br>H 4.283935 8.619025 14.063086<br>H 2.802394 8.129934 13.232346<br>H 4.098204 8.929560 12.329424<br>C 3.864579 6.275236 11.528376<br>H 4.108682 6.885464 10.653212<br>H 2.772451 6.252547 11.624604<br>H 4.198103 5.251296 11.327796<br>C 4.136522 5.875541 13.975051<br>H 4.542177 4.873505 13.796442<br>H 3.047037 5.790433 14.079610	

H	4.543170	6.240529	14.924947
C	8.763476	7.822919	8.344294
S	9.891564	8.117393	9.703065
S	7.112382	7.286342	8.762781
Th	7.602489	8.584053	11.086364
C	9.152053	7.999737	7.040142
S	8.024204	7.704890	5.681284
S	10.803102	8.536296	6.621689
C	9.717335	4.390787	4.651363
C	10.680023	4.400985	3.565685
C	11.908648	4.883343	4.121493
H	12.844518	4.923941	3.577918
C	11.779499	5.108319	5.512976
C	10.418036	4.863276	5.805047
H	9.978133	4.968699	6.787324
C	8.298150	3.792155	4.802259
C	8.331405	2.271778	4.537090
H	9.094614	1.787126	5.157167
H	7.359611	1.833470	4.797203
H	8.532373	2.017111	3.494831
C	7.798791	3.944565	6.253636
H	7.748988	4.991927	6.565260
H	6.785564	3.531114	6.323857
H	8.427825	3.392868	6.961179
C	7.231268	4.458794	3.914930
H	7.479656	4.439126	2.853042
H	6.270931	3.941294	4.037146
H	7.080945	5.499543	4.221827
C	10.730627	3.713853	2.182022
C	11.236677	2.267383	2.395676
H	10.565771	1.686909	3.033731
H	11.321310	1.749463	1.431265
H	12.225332	2.270836	2.867748
C	11.754778	4.401131	1.255978
H	12.778411	4.334249	1.635883
H	11.746061	3.911803	0.274925
H	11.519467	5.458501	1.103850
C	9.410278	3.660301	1.400369
H	8.647493	3.057729	1.893874
H	8.993942	4.658527	1.230743
H	9.589368	3.209582	0.416665
C	12.929126	5.209691	6.514466
C	13.891601	6.361908	6.196231
H	13.392129	7.328528	6.317016
H	14.749103	6.342656	6.880741
H	14.286119	6.289974	5.175935
C	13.713708	3.881065	6.429065
H	14.157177	3.738516	5.436844
H	14.525341	3.871976	7.168025
H	13.058827	3.025925	6.632479
C	12.410251	5.366397	7.948848
H	11.803079	4.504252	8.248005
H	13.256941	5.431795	8.643084
H	11.806978	6.271474	8.065742
C	9.694803	8.780052	1.895044
C	9.721644	9.764019	2.956001

	C 11.077162 9.813070 3.412145<br>H 11.433849 10.484149 4.180483<br>C 11.900169 8.947915 2.659053<br>C 11.027225 8.278227 1.766925<br>H 11.348320 7.582035 1.003994<br>C 8.591547 8.340003 0.910435<br>C 7.451240 7.589690 1.622573<br>H 7.005678 8.170455 2.432082<br>H 6.656049 7.330052 0.911931<br>H 7.811818 6.645399 2.054651<br>C 9.162366 7.385087 -0.156085<br>H 9.593573 6.477776 0.276819<br>H 8.355610 7.071759 -0.828900<br>H 9.932325 7.872199 -0.764892<br>C 8.034080 9.534530 0.110949<br>H 8.844663 10.067147 -0.399641<br>H 7.336633 9.170371 -0.653937<br>H 7.494828 10.253736 0.726471<br>C 8.756205 10.894651 3.386060<br>C 7.259740 10.547752 3.429954<br>H 6.848799 10.252040 2.463864<br>H 7.064067 9.751061 4.154197<br>H 6.701088 11.434879 3.753084<br>C 8.978731 12.079157 2.415082<br>H 8.728332 11.827601 1.381244<br>H 8.355577 12.931343 2.716387<br>H 10.025875 12.402719 2.435323<br>C 9.105612 11.411335 4.796845<br>H 8.383220 12.186282 5.080062<br>H 9.062949 10.611796 5.540734<br>H 10.098170 11.870645 4.843188<br>C 13.422589 8.992439 2.571086<br>C 14.024881 7.615258 2.259193<br>H 13.631658 7.202587 1.323416<br>H 15.113771 7.693001 2.152340<br>H 13.819030 6.894038 3.057291<br>C 14.051213 9.548583 3.856081<br>H 13.806552 8.939257 4.731726<br>H 15.143385 9.570824 3.760237<br>H 13.717947 10.572817 4.055590<br>C 13.779589 9.946597 1.409078<br>H 13.373493 10.948614 1.586795<br>H 14.869076 10.032037 1.304824<br>H 13.373410 9.580745 0.459315<br>Th 10.313495 7.238612 4.297981	
**8′**	Th 9.239711 4.449577 18.443027<br>Th 8.115718 2.512745 12.042222<br>S 9.219197 2.203382 16.902503<br>S 7.321030 1.960287 14.598513<br>S 9.972830 5.270538 15.929136<br>S 8.349277 4.863866 13.451341<br>C 10.968446 2.944691 21.882117<br>C 12.567777 2.033186 18.805724<br>C 12.048515 7.113126 19.154799<br>C 7.016042 7.156879 20.723056<br>C 6.280629 6.972008 17.240262	

C	6.071882	2.323808	19.439720
C	12.093329	3.329532	12.076015
C	10.936531	1.827599	9.056312
C	9.516994	-1.026369	13.023718
C	4.032315	2.460951	12.587620
C	5.251403	0.484163	9.861439
C	6.970696	5.269622	9.466995
C	11.184200	3.663106	20.527964
C	11.833473	3.312089	19.279404
C	12.055725	4.545579	18.585195
H	12.549801	4.623770	17.626974
C	11.657352	5.651458	19.373009
C	11.063407	5.090351	20.524941
H	10.705565	5.663657	21.368963
C	9.733953	3.504448	22.615965
H	9.807242	4.575779	22.819525
H	9.625417	3.000831	23.584043
H	8.817163	3.328464	22.045174
C	12.198568	3.255426	22.768128
H	13.127202	2.897530	22.314753
H	12.090771	2.772244	23.747956
H	12.301201	4.333922	22.931104
C	10.778683	1.423011	21.843371
H	9.926825	1.131331	21.220306
H	10.577236	1.064973	22.860446
H	11.665536	0.894163	21.491056
C	11.728112	0.745727	18.715381
H	11.129175	0.548444	19.601571
H	12.398450	-0.111662	18.572049
H	11.054573	0.781241	17.856267
C	13.769232	1.796829	19.748425
H	14.377883	2.703827	19.842359
H	14.406916	1.001507	19.341769
H	13.463492	1.488788	20.751422
C	13.161586	2.242480	17.398014
H	12.389899	2.518681	16.672453
H	13.618179	1.303711	17.062440
H	13.946414	3.006986	17.388517
C	13.581976	7.173648	18.994869
H	14.085769	6.751762	19.872087
H	13.909454	8.214815	18.880236
H	13.915519	6.617757	18.112142
C	11.667530	7.968121	20.371303
H	10.586843	7.972716	20.549685
H	11.977408	9.006839	20.207963
H	12.160178	7.611401	21.283116
C	11.404096	7.713538	17.897826
H	11.700599	7.171052	16.994695
H	11.705486	8.761393	17.773559
H	10.308363	7.690649	17.955542
C	7.006869	5.995544	19.701375
C	6.680293	5.907028	18.290094
C	6.467238	4.515580	18.018526
H	6.185426	4.114633	17.053986
C	6.583054	3.743351	19.198522
C	6.974723	4.657313	20.204764

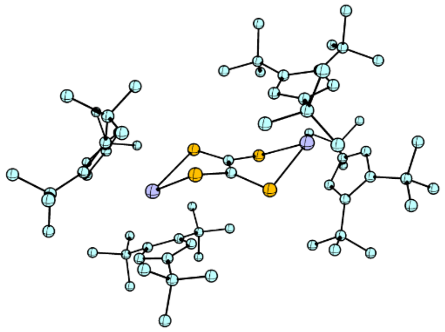

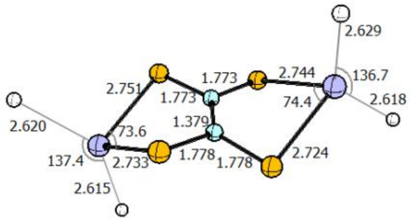

	H	7.095806	4.401561	21.249763	
	C	7.897237	6.823219	21.942299	
	H	7.558310	5.934320	22.480958	
	H	7.867696	7.658616	22.651832	
	H	8.940072	6.669925	21.651637	
	C	7.510076	8.515884	20.207535	
	H	8.526197	8.453556	19.803012	
	H	7.532570	9.229970	21.039817	
	H	6.866129	8.942189	19.438406	
	C	5.573218	7.319830	21.257795	
	H	4.864459	7.567557	20.464000	
	H	5.536610	8.119945	22.008682	
	H	5.231725	6.392875	21.731779	
	C	7.399732	7.957206	16.854894	
	H	7.845090	8.461132	17.713285	
	H	6.994506	8.732596	16.191705	
	H	8.196665	7.439561	16.311863	
	C	5.039336	7.748770	17.730962	
	H	4.238024	7.058472	18.019640	
	H	4.660821	8.381878	16.918480	
	H	5.244467	8.402381	18.580590	
	C	5.840807	6.290242	15.931294	
	H	6.628764	5.667397	15.498856	
	H	5.595230	7.062662	15.192892	
	H	4.945336	5.675142	16.074371	
	C	7.114462	1.442755	20.142827	
	H	7.999245	1.303138	19.511063	
	H	6.697922	0.449042	20.350889	
	H	7.426389	1.872974	21.101501	
	C	4.832022	2.436055	20.354228	
	H	5.087190	2.870983	21.327409	
	H	4.399903	1.442324	20.529731	
	H	4.061998	3.065969	19.894085	
	C	5.642798	1.648347	18.132435	
	H	4.844168	2.212005	17.636515	
	H	5.257317	0.643547	18.345162	
	H	6.478318	1.550295	17.433435	
	C	11.032607	2.242483	11.779385	
	C	10.545171	1.661449	10.542806	
	C	9.802113	0.497433	10.915901	
	H	9.376881	-0.200971	10.206527	
	C	9.850797	0.285433	12.313363	
	C	10.556180	1.396461	12.833289	
	H	10.763190	1.548914	13.884227	
	C	13.443811	2.932990	11.441018	
	H	13.726288	1.913245	11.727936	
	H	14.227896	3.612421	11.798350	
	H	13.441502	2.989362	10.351047	
	C	12.362866	3.408825	13.590259	
	H	11.462194	3.647983	14.162210	
	H	13.090203	4.206611	13.780877	
	H	12.787505	2.475570	13.976852	
	C	11.704558	4.751739	11.633108	
	H	11.432920	4.814024	10.578637	
	H	12.550295	5.432290	11.796192	
	H	10.861463	5.121834	12.225082	

	C 11.274798 3.252369 8.596645<br>H 10.446514 3.945317 8.779791<br>H 11.465466 3.247570 7.516526<br>H 12.166067 3.656856 9.076215<br>C 9.808577 1.339543 8.125915<br>H 9.580212 0.278023 8.256505<br>H 10.114195 1.474937 7.081799<br>H 8.887672 1.908494 8.281136<br>C 12.152414 0.910373 8.784532<br>H 13.018281 1.178839 9.394094<br>H 12.446095 0.976665 7.728704<br>H 11.902532 -0.133903 9.002514<br>C 8.062913 -1.466287 12.794365<br>H 7.357614 -0.783421 13.281239<br>H 7.896351 -2.463772 13.220693<br>H 7.822637 -1.524643 11.726438<br>C 10.449975 -2.107800 12.434432<br>H 10.263668 -2.264199 11.365749<br>H 10.293039 -3.064621 12.948835<br>H 11.501555 -1.823898 12.556185<br>C 9.785336 -0.935466 14.530323<br>H 10.841149 -0.722136 14.733265<br>H 9.542167 -1.892592 15.007842<br>H 9.180410 -0.156832 15.003832<br>C 5.201922 2.643396 11.589667<br>C 5.690145 1.827043 10.494772<br>C 6.510166 2.686456 9.694362<br>H 6.937613 2.393994 8.745199<br>C 6.518589 4.005250 10.200783<br>C 5.771286 3.944640 11.401973<br>H 5.578298 4.789819 12.047188<br>C 4.129337 1.261848 13.549337<br>H 4.386564 0.324891 13.059563<br>H 3.161436 1.119518 14.047371<br>H 4.872722 1.450779 14.327430<br>C 3.891440 3.704879 13.487784<br>H 4.815041 3.910688 14.038078<br>H 3.097218 3.522781 14.221714<br>H 3.610933 4.599187 12.920079<br>C 2.720000 2.366256 11.777528<br>H 2.634580 3.199967 11.070839<br>H 1.861514 2.411230 12.459689<br>H 2.639540 1.434392 11.212787<br>C 4.193936 0.806775 8.778694<br>H 3.311887 1.295307 9.202101<br>H 3.865838 -0.116865 8.284232<br>H 4.608436 1.472252 8.013469<br>C 4.665213 -0.573765 10.804633<br>H 5.365097 -0.844078 11.602018<br>H 4.456565 -1.484334 10.229864<br>H 3.723889 -0.261984 11.259351<br>C 6.433486 -0.197633 9.143918<br>H 6.847604 0.408640 8.334265<br>H 6.096305 -1.140958 8.698103<br>H 7.243324 -0.429150 9.842745<br>C 7.455561 4.930298 8.050196	

	H	8.325064	4.264039	8.059418	
	H	7.750292	5.848295	7.528379	
	H	6.666850	4.448771	7.460706	
	C	5.748645	6.202903	9.338677	
	H	4.924512	5.703128	8.816938	
	H	6.017636	7.102840	8.771050	
	H	5.384245	6.522294	10.320837	
	C	8.081422	6.028991	10.207371	
	H	7.757252	6.350733	11.201967	
	H	8.371839	6.924787	9.643775	
	H	8.982587	5.415104	10.332961	
	C	8.530116	2.952541	15.444441	
	C	8.904621	4.202511	14.999900	
**22** (Th-NC)	C	5.258520	9.233933	12.105271	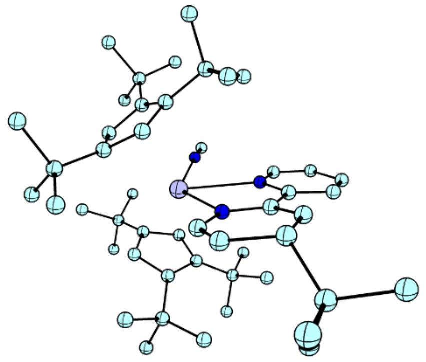 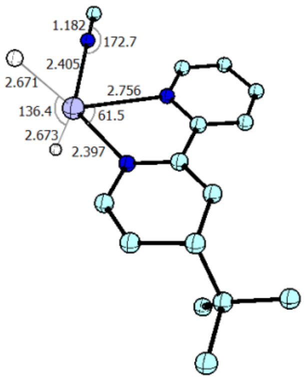
	C	4.331029	10.320446	11.862798	
	C	3.428857	10.326661	12.973670	
	H	2.680287	11.090402	13.141958	
	C	3.800213	9.354344	13.936106	
	C	4.875078	8.650802	13.353067	
	H	5.395035	7.832806	13.829736	
	C	6.627893	8.867179	11.485040	
	C	7.610633	10.040660	11.698055	
	H	7.376335	10.908045	11.078445	
	H	8.627725	9.719970	11.438318	
	H	7.615058	10.361525	12.746185	
	C	6.601578	8.480522	9.993178	
	H	6.307174	7.435937	9.872309	
	H	7.606905	8.582701	9.564191	
	H	5.917287	9.086704	9.401846	
	C	7.258830	7.669809	12.224864	
	H	7.506186	7.908400	13.265116	
	H	8.192015	7.392920	11.719926	
	H	6.606284	6.791011	12.218006	
	C	4.390302	11.587774	10.978613	
	C	2.993737	12.190468	10.730412	
	H	2.382957	11.537971	10.103119	
	H	3.105828	13.140508	10.194112	
	H	2.446991	12.409778	11.652009	
	C	5.182504	12.656434	11.771189	
	H	4.672126	12.895229	12.711248	
	H	5.261252	13.580651	11.183695	
	H	6.193606	12.325481	12.018705	
	C	5.018697	11.419775	9.589108	
	H	6.076020	11.153598	9.624277	
	H	4.945836	12.370084	9.045481	
	H	4.487733	10.661987	9.005144	
	C	3.461899	9.388328	15.426466	
	C	2.046829	9.911103	15.697965	
	H	1.900096	10.921453	15.300265	
	H	1.864480	9.955782	16.778636	
	H	1.288278	9.259569	15.255654	
	C	4.464730	10.372274	16.073457	
	H	5.495927	10.029136	15.934084	
	H	4.275009	10.456782	17.151594	
	H	4.378007	11.371712	15.631866	
	C	3.628757	8.023300	16.102690	

	H 2.922553 7.290348 15.707828<br>H 3.453362 8.119782 17.181387<br>H 4.638007 7.620466 15.967771<br>C 0.141627 6.364540 12.732045<br>C 0.097879 6.354421 11.281253<br>C -0.104902 7.708345 10.874709<br>H -0.243980 8.016071 9.847538<br>C -0.213379 8.566935 11.994387<br>C 0.014527 7.730305 13.119107<br>H -0.073525 8.059577 14.145717<br>C -0.112177 5.281813 13.809923<br>C 0.441273 3.874637 13.539710<br>H 0.000016 3.401572 12.661360<br>H 0.205870 3.232586 14.397633<br>H 1.528108 3.886652 13.427039<br>C 0.446066 5.697548 15.185046<br>H 1.532957 5.791678 15.159547<br>H 0.195609 4.922934 15.919856<br>H 0.016944 6.633386 15.556268<br>C -1.649698 5.182117 13.970447<br>H -2.070908 6.145680 14.279012<br>H -1.899215 4.439866 14.740099<br>H -2.144680 4.885152 13.041810<br>C -0.007762 5.213019 10.244971<br>C -0.084692 5.779816 8.812620<br>H -1.000940 6.358602 8.649887<br>H -0.096017 4.945282 8.100978<br>H 0.773658 6.413058 8.567685<br>C 1.186309 4.241807 10.259262<br>H 2.080259 4.729724 9.861514<br>H 0.972380 3.376519 9.618121<br>H 1.429664 3.872527 11.254676<br>C -1.325343 4.434072 10.447192<br>H -1.347203 3.862614 11.376338<br>H -1.463952 3.723377 9.622240<br>H -2.181955 5.118239 10.450265<br>C -0.963945 9.901768 12.038610<br>C -0.766368 10.760692 10.783179<br>H 0.246196 11.173030 10.726842<br>H -1.458652 11.611375 10.803602<br>H -0.965124 10.199208 9.863884<br>C -0.600255 10.741534 13.268403<br>H -0.805968 10.208743 14.202623<br>H -1.192120 11.664978 13.280489<br>H 0.458138 11.019326 13.263623<br>C -2.466063 9.541552 12.128745<br>H -2.786116 8.974114 11.247701<br>H -3.075936 10.452516 12.190063<br>H -2.671843 8.931867 13.015361<br>C 4.896360 5.153163 10.747984<br>H 4.840621 5.011175 11.818055<br>C 5.707972 4.340270 9.973163<br>H 6.301179 3.564294 10.445478<br>C 5.739480 4.558849 8.597306<br>H 6.369957 3.956853 7.948727<br>C 4.955947 5.570232 8.067706	

	H	4.986450	5.774532	7.004652	
	C	4.144940	6.346965	8.908780	
	C	3.283034	7.428621	8.405577	
	C	3.045679	7.583371	7.076788	
	H	3.477237	6.864979	6.389197	
	C	2.164149	8.643732	6.475511	
	H	1.334015	8.140672	5.939562	
	C	1.597620	9.459619	7.607813	
	H	0.895484	10.258546	7.385869	
	C	1.886204	9.214185	8.900127	
	H	1.408289	9.820667	9.664036	
	C	2.870877	9.489943	5.348779	
	C	4.047352	10.284365	5.919988	
	H	3.709136	10.982992	6.693338	
	H	4.546060	10.863562	5.132625	
	H	4.791240	9.618830	6.374861	
	C	1.848053	10.456061	4.733828	
	H	0.974477	9.916190	4.345321	
	H	2.296738	11.008497	3.898976	
	H	1.494613	11.194842	5.461366	
	C	3.371706	8.553535	4.238999	
	H	4.180559	7.899621	4.584834	
	H	3.764576	9.135612	3.396252	
	H	2.561456	7.918934	3.856154	
	C	3.725392	5.191859	13.871603	
	N	4.129255	6.142295	10.248323	
	N	2.712428	8.216619	9.398684	
	N	3.274095	6.014039	13.152564	
	Th	2.598004	7.859468	11.766181	
**22′** (Th-CN)	C	5.242828	9.238349	12.110585	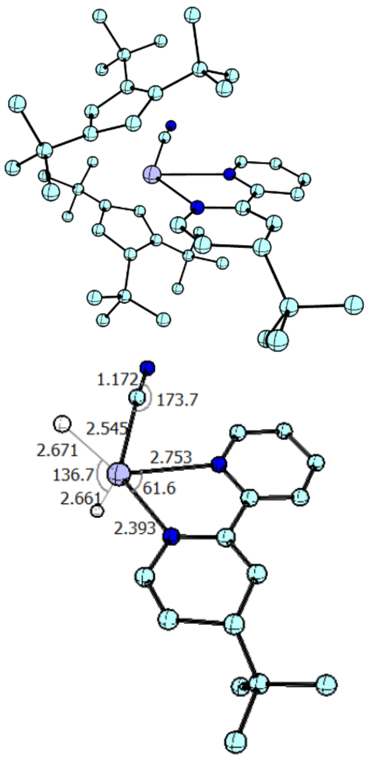
	C	4.321591	10.329254	11.865055	
	C	3.421528	10.345473	12.979570	
	H	2.678149	11.115027	13.145236	
	C	3.789221	9.376875	13.945751	
	C	4.858724	8.663152	13.362842	
	H	5.379815	7.849827	13.845881	
	C	6.606768	8.857219	11.487221	
	C	7.601843	10.021035	11.695899	
	H	7.374237	10.890711	11.076911	
	H	8.614566	9.689780	11.432575	
	H	7.613538	10.342127	12.743909	
	C	6.570649	8.468486	9.995803	
	H	6.279371	7.422456	9.879114	
	H	7.572046	8.573425	9.558574	
	H	5.879064	9.070607	9.408776	
	C	7.227182	7.653824	12.226440	
	H	7.485021	7.891809	13.264267	
	H	8.153323	7.363923	11.715895	
	H	6.563335	6.783450	12.227075	
	C	4.388940	11.594056	10.977975	
	C	2.995992	12.203712	10.726433	
	H	2.383388	11.553219	10.098506	
	H	3.113840	13.152362	10.188974	
	H	2.448304	12.426804	11.646533	
	C	5.187447	12.658141	11.770315	
	H	4.678213	12.901712	12.709720	

H	5.273317	13.580914	11.181692
H	6.195931	12.319763	12.018760
C	5.018556	11.419891	9.589907
H	6.073106	11.143353	9.626841
H	4.955515	12.370991	9.046551
H	4.481506	10.667375	9.004741
C	3.454919	9.420428	15.437003
C	2.034166	9.926754	15.709359
H	1.866886	10.925526	15.291389
H	1.860371	9.991335	16.790416
H	1.283872	9.251664	15.288825
C	4.448231	10.424851	16.067680
H	5.483208	10.094628	15.924930
H	4.264330	10.516209	17.146193
H	4.344936	11.419002	15.617836
C	3.642888	8.066442	16.130146
H	2.942312	7.318365	15.754165
H	3.476213	8.178513	17.208743
H	4.654738	7.671929	15.992195
C	0.166383	6.362102	12.726325
C	0.102682	6.351308	11.274658
C	-0.108829	7.702990	10.869567
H	-0.257097	8.010462	9.843459
C	-0.214575	8.562210	11.990988
C	0.027791	7.727679	13.112178
H	-0.050637	8.057471	14.139286
C	-0.080459	5.279066	13.806338
C	0.484195	3.876918	13.533787
H	0.038942	3.397903	12.660438
H	0.262382	3.234036	14.394749
H	1.570248	3.901712	13.413268
C	0.477175	5.697079	15.181207
H	1.564854	5.782413	15.157767
H	0.219401	4.925818	15.917113
H	0.051696	6.636039	15.548747
C	-1.617062	5.171028	13.970137
H	-2.041945	6.131094	14.284726
H	-1.860291	4.424345	14.737389
H	-2.114132	4.875827	13.042272
C	-0.011906	5.209534	10.239467
C	-0.103465	5.776249	8.807890
H	-1.022280	6.353313	8.653564
H	-0.120200	4.941650	8.096498
H	0.751413	6.410990	8.554432
C	1.179369	4.235129	10.241294
H	2.069966	4.718220	9.831617
H	0.953994	3.369080	9.605162
H	1.432598	3.866642	11.234490
C	-1.327425	4.430871	10.456573
H	-1.334675	3.854342	11.382877
H	-1.478051	3.724488	9.630085
H	-2.183890	5.114920	10.475763
C	-0.967912	9.894989	12.038230
C	-0.763789	10.760428	10.788359
H	0.250307	11.170221	10.738931
H	-1.453835	11.612863	10.810714

	H	-0.960053	10.204518	9.865154	
	C	-0.609981	10.728337	13.273897	
	H	-0.820913	10.191452	14.204534	
	H	-1.200814	11.652380	13.287233	
	H	0.448808	11.004684	13.275608	
	C	-2.469929	9.532888	12.119568	
	H	-2.786028	8.970559	11.233859	
	H	-3.080720	10.442977	12.183868	
	H	-2.678715	8.917773	13.001713	
	C	4.837953	5.137507	10.756456	
	H	4.774097	4.998455	11.827608	
	C	5.637236	4.306898	9.987198	
	H	6.211846	3.520146	10.464601	
	C	5.681374	4.521503	8.611203	
	H	6.304279	3.905985	7.968023	
	C	4.919949	5.545770	8.073675	
	H	4.960202	5.746320	7.010198	
	C	4.117889	6.339288	8.907174	
	C	3.274364	7.430658	8.393033	
	C	3.038418	7.577879	7.063197	
	H	3.457786	6.847045	6.381075	
	C	2.173463	8.647744	6.453916	
	H	1.344776	8.152888	5.908530	
	C	1.600718	9.467378	7.580412	
	H	0.896701	10.262848	7.352197	
	C	1.886689	9.229157	8.874528	
	H	1.405639	9.838941	9.634887	
	C	2.899922	9.488771	5.335919	
	C	4.079796	10.269063	5.919395	
	H	3.742391	10.971082	6.690019	
	H	4.592578	10.843225	5.137448	
	H	4.811708	9.594686	6.380535	
	C	1.893333	10.467649	4.714436	
	H	1.018179	9.938388	4.314993	
	H	2.356026	11.019005	3.886548	
	H	1.540209	11.206883	5.441698	
	C	3.400501	8.549002	4.229017	
	H	4.198232	7.885137	4.581636	
	H	3.808033	9.128206	3.391287	
	H	2.586681	7.924596	3.837092	
	N	3.810802	5.175523	13.954461	
	N	4.093078	6.140560	10.248145	
	N	2.712901	8.234085	9.378985	
	C	3.344465	6.013828	13.281226	
	Th	2.595112	7.899865	11.745508	

Table S7. Frequencies of the stationary points optimized for some stationary points, obtained with B3PW91-PCM/6-31G(d)/ECP60MWB method.

Species	Frequencies (cm^{-1})
**1**	23 27 41 46 51 56 66 68 71 72 79 81 84 89 96 100 102 110
	118 128 132 136 143 158 167 175 180 185 187 190 199 204 204 211 220 229 233
	234 238 238 246 248 250 255 264 265 270 274 277 290 291 295 305 310 315 316
	322 327 342 350 352 353 359 365 369 372 373 376 381 384 390 394 395 397 401
	405 423 427 430 434 435 437 465 466 475 476 523 541 542 554 555 562 563 595
	597 606 647 649 654 657 660 671 674 689 690 707 730 738 746 791 799 801 810
	830 830 832 833 845 852 854 857 930 933 937 937 940 940 942 944 946 947 949
	951 953 954 956 957 959 962 963 969 969 970 972 973 975 996 1020 1022
	1023 1036 1038 1049 1051 1051 1051 1052 1054 1055
	1057 1058 1060 1062 1063 1097 1139 1145 1155 1178
	1180 1194 1197 1202 1205 1224 1224 1230 1232 1236
	1238 1239 1239 1241 1242 1244 1260 1261 1270 1272
	1276 1277 1285 1312 1313 1317 1327 1381 1387 1401
	1403 1405 1412 1412 1412 1414 1415 1415 1416 1420
	1422 1425 1425 1429 1435 1442 1442 1447 1449 1453
	1454 1455 1458 1486 1492 1497 1498 1499 1501 1501
	1502 1503 1506 1507 1507 1508 1510 1510 1512 1515
	1517 1518 1519 1521 1522 1523 1524 1525 1526 1528
	1529 1530 1532 1534 1536 1539 1540 1545 1545 1547
	1550 1550 1553 1555 1574 1582 1661 1669 3045 3047
	3049 3050 3052 3053 3055 3055 3056 3056 3058 3061
	3061 3062 3063 3065 3072 3074 3116 3116 3119 3120
	3122 3123 3124 3124 3124 3125 3125 3128 3130 3130
	3133 3134 3134 3137 3140 3143 3144 3145 3145 3149
	3149 3153 3159 3163 3167 3167 3173 3180 3185 3190
	3197 3198 3199 3202 3217 3220 3223 3229 3238 3240
	3256 3276 3278 3283
**1′**	23 25 29 35 40 43 46 55 56 57 64 65 65 72 72 76 81 84
	89 91 99 102 104 110 113 119 125 129 133 133 137 138 141 144 148 149 151
	152 155 161 164 167 169 171 172 174 178 180 181 187 188 191 193 195 196 199
	202 207 208 210 211 214 216 218 219 223 225 229 229 231 237 243 250 265 266
	272 274 284 294 299 302 357 358 362 366 369 373 375 393 406 427 428 432 436
	507 510 519 607 621 622 623 624 635 637 643 644 651 652 653 659 664 684 685
	686 688 688 690 690 691 693 693 693 694 696 697 698 700 700 702 709 732 737
	749 775 776 778 780 780 781 781 782 782 785 786 788 794 804 849 857 860 863
	867 868 868 870 871 871 873 875 876 876 877 878 879 883 883 889 890 894 935
	942 942 946 950 957 964 995 1003 1004 1024 1037 1039 1104
	1123 1126 1158 1178 1181 1187 1189 1238 1241 1242
	1284 1290 1293 1298 1304 1305 1306 1306 1307 1308
	1308 1309 1310 1312 1313 1314 1315 1318 1318 1319
	1319 1321 1323 1326 1329 1396 1398 1403 1435 1472
	1473 1474 1475 1476 1476 1477 1478 1479 1479 1479
	1479 1480 1480 1482 1482 1483 1484 1486 1487 1487
	1487 1488 1490 1490 1490 1491 1492 1493 1493 1494
	1495 1497 1498 1498 1499 1501 1502 1502 1506 1537
	1570 1577 1664 1669 3050 3050 3051 3051 3051 3052
	3052 3053 3054 3054 3055 3056 3056 3057 3058 3059
	3059 3061 3127 3129 3129 3129 3130 3130 3131 3131
	3132 3132 3132 3132 3133 3133 3135 3135 3136 3138
	3138 3139 3142 3145 3147 3149 3149 3149 3152 3152
	3153 3153 3155 3156 3156 3157 3159 3167 3197 3203
	3205 3217 3222 3229 3231 3235 3238 3238 3240 3241
**8**	7 23 24 26 26 39 43 43 49 50 54 62 65 66 68 69 72 72

	77 77 79 80 84 88 94 95 97 101 101 102 103 104 109 121 122 130 130<br>133 134 136 138 146 150 154 157 169 170 171 173 176 179 183 189 189 191 192<br>195 195 204 204 207 207 211 211 221 221 225 225 240 241 244 244 246 248 249<br>254 255 262 262 265 265 269 269 271 271 277 278 281 281 281 282 287 288 288<br>289 289 295 295 304 306 307 318 319 324 324 326 326 331 331 333 334 342 348<br>348 348 352 353 353 355 356 357 368 368 372 373 374 377 377 380 380 383 383<br>387 387 394 394 395 395 399 399 405 405 425 425 429 430 433 434 434 436 436<br>456 456 465 465 475 475 476 476 489 489 534 547 547 553 553 558 558 559 559<br>562 563 567 567 597 597 598 598 649 649 651 651 667 667 679 679 695 695 706<br>706 724 802 802 804 804 815 829 829 830 830 832 832 834 834 846 847 851 851<br>854 854 859 859 902 934 934 937 937 938 938 939 939 940 940 942 943 947 947<br>951 951 952 952 953 953 954 954 959 959 959 959 962 962 963 963 967 967 970<br>970 971 971 972 972 975 975 1022 1022 1024 1024 1048 1048<br>1049 1049 1050 1050 1051 1051 1053 1053 1054 1054<br>1055 1055 1056 1056 1059 1059 1062 1062 1064 1064<br>1071 1071 1139 1139 1141 1141 1194 1194 1198 1198<br>1205 1205 1208 1208 1223 1223 1224 1224 1231 1231<br>1233 1233 1236 1237 1239 1239 1240 1240 1241 1241<br>1243 1243 1245 1245 1260 1260 1261 1261 1271 1271<br>1274 1274 1276 1276 1278 1278 1315 1315 1315 1315<br>1383 1383 1391 1391 1404 1404 1405 1405 1408 1408<br>1410 1410 1411 1411 1412 1412 1413 1414 1414 1414<br>1415 1415 1420 1420 1422 1422 1423 1424 1424 1425<br>1426 1426 1438 1438 1443 1443 1445 1445 1448 1448<br>1450 1450 1454 1455 1456 1456 1460 1460 1462 1493<br>1493 1495 1495 1499 1499 1500 1500 1502 1502 1502<br>1502 1504 1504 1504 1504 1506 1506 1507 1508 1509<br>1509 1511 1511 1514 1515 1515 1515 1515 1515 1516<br>1517 1517 1517 1520 1520 1522 1522 1522 1522 1523<br>1523 1525 1525 1526 1527 1528 1528 1530 1531 1532<br>1532 1534 1534 1535 1535 1537 1537 1540 1540 1541<br>1542 1545 1546 1547 1547 1550 1550 1551 1551 1555<br>1555 1556 1556 1632 1632 3045 3045 3046 3046 3046<br>3046 3050 3050 3051 3051 3052 3052 3053 3053 3055<br>3055 3055 3055 3056 3056 3057 3057 3061 3061 3061<br>3062 3063 3063 3065 3065 3065 3065 3068 3068 3071<br>3071 3104 3105 3118 3118 3120 3120 3120 3120 3120<br>3120 3121 3121 3123 3123 3124 3124 3124 3125 3126<br>3126 3126 3126 3127 3127 3128 3128 3132 3132 3132<br>3132 3133 3133 3133 3133 3135 3135 3137 3137 3137<br>3137 3138 3138 3144 3144 3150 3150 3155 3155 3155<br>3155 3156 3156 3157 3157 3160 3160 3163 3163 3171<br>3171 3172 3172 3177 3177 3183 3183 3183 3184 3191<br>3191 3199 3199 3253 3253 3265 3265 3282 3283 3289<br>3289
**8′**	7 14 21 29 30 33 37 43 46 49 53 56 60 60 62 65 66 69<br>74 75 77 78 81 84 86 90 94 95 97 98 101 102 105 108 113 116 119<br>132 133 134 138 139 145 166 171 173 176 178 180 181 183 187 189 190 193 196<br>201 203 204 207 210 211 215 218 221 223 228 235 237 237 243 244 244 248 249<br>253 255 255 258 259 260 261 264 267 268 273 276 277 279 280 284 285 288 289<br>292 292 295 299 302 313 316 316 321 323 323 327 334 335 339 344 348 352 352<br>353 355 357 357 358 360 362 363 364 366 367 370 373 375 378 379 381 381 382<br>382 384 389 391 393 399 404 406 410 410 416 423 426 427 431 431 436 438 440<br>444 446 466 467 467 468 476 477 479 479 544 545 546 547 551 554 556 557 559<br>563 564 566 567 594 595 596 597 648 648 650 650 670 677 680 681 685 687 689<br>690 736 797 809 815 815 829 831 831 833 834 834 836 838 840 846 850 853 855<br>856 858 862 865 936 936 937 937 937 939 941 941 942 942 944 944 947 947 950

	950 951 952 952 952 954 954 955 956 957 963 965 966 966 968 968 969 970 970<br>972 973 974 975 975 976 976 1018 1019 1019 1022 1050 1050 1051 1051 1052 1052 1052 1053<br>1054 1055 1055 1055 1055 1056 1057 1057 1059 1059 1060 1063 1063 1063 1064 1072 1140 1140 1144<br>1146 1194 1195 1197 1198 1202 1203 1206 1207 1223 1224 1224 1225 1232 1232 1233 1234 1235 1236<br>1236 1238 1238 1238 1240 1241 1244 1245 1246 1248 1259 1259 1261 1261 1269 1270 1272 1273 1275<br>1276 1277 1278 1310 1311 1313 1313 1379 1380 1381 1383 1400 1400 1401 1403 1409 1410 1410 1411<br>1412 1414 1414 1415 1415 1416 1417 1418 1419 1419 1420 1421 1422 1423 1423 1424 1424 1425 1425<br>1426 1440 1441 1442 1442 1444 1446 1448 1449 1451 1452 1454 1454 1455 1455 1456 1456 1457 1494<br>1497 1499 1499 1501 1501 1502 1502 1502 1503 1504 1504 1505 1505 1505 1506 1506 1507 1508 1508<br>1508 1509 1510 1511 1512 1512 1513 1514 1516 1516 1517 1517 1519 1519 1520 1520 1522 1522 1523<br>1524 1524 1524 1525 1526 1527 1527 1527 1528 1528 1530 1530 1532 1533 1533 1534 1535 1535 1535<br>1538 1538 1540 1540 1544 1544 1545 1546 1548 1549 1550 1551 1557 1558 1559 1560 1564 1566 3045<br>3045 3048 3049 3050 3050 3051 3051 3053 3053 3053 3054 3054 3054 3054 3055 3055 3056 3056 3056<br>3058 3058 3059 3059 3063 3063 3063 3063 3063 3064 3065 3066 3070 3070 3071 3072 3117 3118 3118<br>3120 3121 3121 3121 3121 3121 3123 3124 3125 3125 3125 3125 3125 3126 3126 3126 3127 3127 3127<br>3128 3128 3129 3131 3131 3132 3132 3132 3133 3134 3134 3136 3137 3138 3138 3139 3140 3140 3140<br>3141 3142 3145 3146 3147 3151 3152 3152 3152 3155 3155 3158 3161 3161 3163 3163 3163 3163 3164<br>3167 3167 3171 3174 3177 3179 3185 3188 3193 3198 3221 3226 3260 3260 3272 3275 3275 3278 3281<br>3289
**22** (Th-NC)	16 25 31 34 41 48 51 56 64 64 67 68 72 79 81 86 89 94<br>100 102 106 121 123 131 135 142 143 146 148 154 162 169 177 179 180 188 196<br>199 200 208 212 219 223 226 229 233 239 245 247 249 257 257 265 268 272 273<br>274 276 278 281 287 289 297 301 306 308 313 315 319 323 336 340 345 350 351<br>353 360 368 371 373 374 376 380 381 386 394 395 406 408 422 426 427 432 433<br>438 464 465 467 474 475 485 517 536 538 541 554 555 561 563 594 596 635 646<br>647 650 658 670 673 675 689 706 732 759 784 789 791 803 813 830 830 831 833<br>836 853 855 856 865 881 900 933 934 935 936 940 941 947 950 951 952 954 957<br>958 960 963 965 966 967 968 969 970 970 972 979 984 1003 1016 1019<br>1021 1022 1028 1046 1049 1049 1050 1052 1052 1053<br>1053 1054 1055 1055 1057 1057 1060 1063 1089 1105<br>1141 1142 1148 1169 1196 1197 1203 1204 1207 1223<br>1223 1225 1229 1230 1233 1234 1236 1239 1242 1243<br>1255 1257 1261 1262 1272 1274 1275 1277 1315 1318<br>1321 1322 1331 1346 1353 1361 1378 1384 1401 1404<br>1410 1410 1411 1412 1412 1414 1415 1417 1417 1418<br>1419 1422 1423 1424 1426 1441 1442 1444 1445 1447<br>1453 1453 1456 1464 1485 1495 1499 1499 1500 1500<br>1501 1502 1503 1503 1506 1506 1507 1508 1508 1509<br>1511 1511 1514 1515 1517 1518 1520 1521 1521 1522<br>1522 1523 1524 1524 1527 1529 1530 1531 1532 1533<br>1535 1536 1538 1541 1542 1543 1545 1548 1549 1554<br>1555 1629 1652 1670 1721 2134 2913 3041 3044 3044<br>3045 3050 3051 3052 3052 3053 3056 3056 3058 3060<br>3062 3063 3064 3065 3066 3066 3070 3071 3113 3116<br>3119 3119 3120 3121 3121 3122 3123 3123 3124 3125<br>3127 3128 3129 3131 3131 3131 3132 3134 3136 3137<br>3138 3143 3145 3146 3147 3150 3151 3157 3158 3159<br>3168 3175 3177 3178 3178 3184 3187 3190 3201 3208<br>3216 3217 3225 3230 3241 3258 3262 3269 3276 3286<br>3289
**22′** (Th-CN)	23 28 32 36 43 50 54 55 63 66 68 68 76 80 86 87 92 98<br>102 103 110 122 129 133 135 141 143 151 152 163 168 172 181 182 185 197 200<br>204 210 220 224 227 232 235 241 246 247 251 255 258 258 265 268 273 274 275<br>278 279 282 287 289 293 299 303 307 310 313 317 321 322 341 342 348 350 352<br>353 360 368 372 373 375 376 380 381 386 395 396 407 408 422 426 427 432 434<br>439 464 466 468 474 475 486 516 537 538 542 554 556 561 563 594 597 635 646<br>647 651 658 668 674 675 690 706 732 760 785 787 792 803 813 830 831 831 833<br>837 854 855 865 870 880 902 934 935 936 937 940 941 948 949 951 953 954 957

	958 960 963 964 966 967 968 968 969 970 973 980 986 1007 1019 1021 1021 1022 1029 1046 1049 1050 1050 1052 1052 1053 1054 1054 1055 1056 1057 1058 1060 1064 1088 1105 1139 1142 1148 1169 1195 1197 1202 1204 1206 1223 1223 1226 1230 1231 1233 1234 1235 1239 1242 1243 1255 1257 1261 1262 1272 1274 1275 1277 1313 1318 1321 1323 1334 1346 1353 1362 1374 1384 1401 1404 1410 1411 1412 1412 1412 1414 1415 1417 1417 1419 1419 1422 1423 1424 1426 1441 1443 1443 1445 1448 1453 1453 1454 1465 1486 1495 1499 1500 1500 1501 1501 1502 1503 1504 1506 1507 1508 1509 1509 1510 1511 1511 1514 1515 1517 1519 1520 1521 1522 1522 1523 1523 1524 1525 1528 1529 1531 1532 1533 1533 1535 1536 1538 1541 1542 1543 1547 1547 1549 1554 1556 1629 1653 1670 1722 2225 2915 3042 3044 3045 3046 3050 3051 3052 3053 3053 3056 3056 3059 3060 3062 3063 3064 3065 3066 3067 3070 3070 3113 3116 3119 3119 3120 3121 3121 3122 3123 3124 3125 3125 3127 3128 3129 3131 3132 3132 3134 3134 3136 3137 3139 3143 3144 3147 3147 3150 3151 3156 3159 3160 3167 3175 3177 3177 3178 3185 3187 3188 3200 3208 3216 3217 3218 3229 3241 3258 3259 3272 3272 3275 3292

Table S8. The electronic energies, enthalpies and free energies (in au at 298 K) and corresponding relative values with ZPE correction (in Kcal·mol^{-1}) and entropies (in cal·mol^{-1}·K^{-1}) for **8** and **8′**, obtained with B3PW91-PCM/6-31G(d)/ECP60MWB method.

	E	ZPE	G	H	S
**8′**	-5143.02939 (0.0)	1.71560	-5143.14513 (0.0)	-5142.93361 (0.0)	445.2
**8**	-5143.03611 (-4.2)	1.71547	-5143.15025 (-3.2)	-5142.94047 (-4.3)	441.5

Table S9. The electronic energies, enthalpies and free energies (in au at 298 K) and corresponding relative values with ZPE correction (in Kcal·mol^{-1}) and entropies (in cal·mol^{-1}·K^{-1}) for **22** and **22′**, obtained with B3PW91-PCM/6-31G(d)/ECP60MWB method.

species	E	ZPE	G	H	S
**22′** (Th-CN)	-2482.60307 (0.0)	1.14044	-2482.68341 (0.0)	-2482.54044 (0.0)	300.90
**22** (Th-NC)	-2482.60435 (-0.8)	1.13944	-2482.68616 (-1.7)	-2482.54125 (-0.5)	305.00

4. NMR spectra

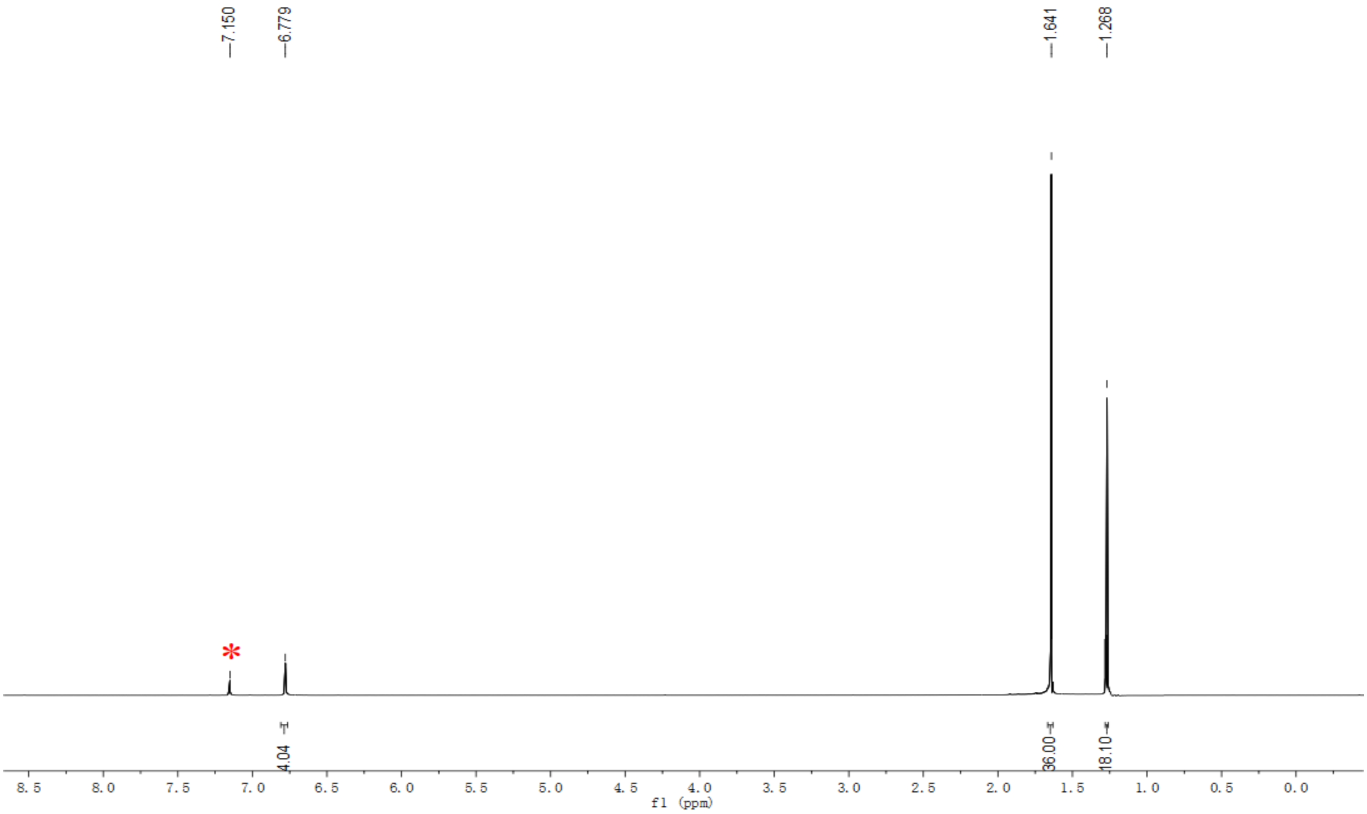

Figure S8. ^{1}H NMR spectrum for compound **2** (* solvent).

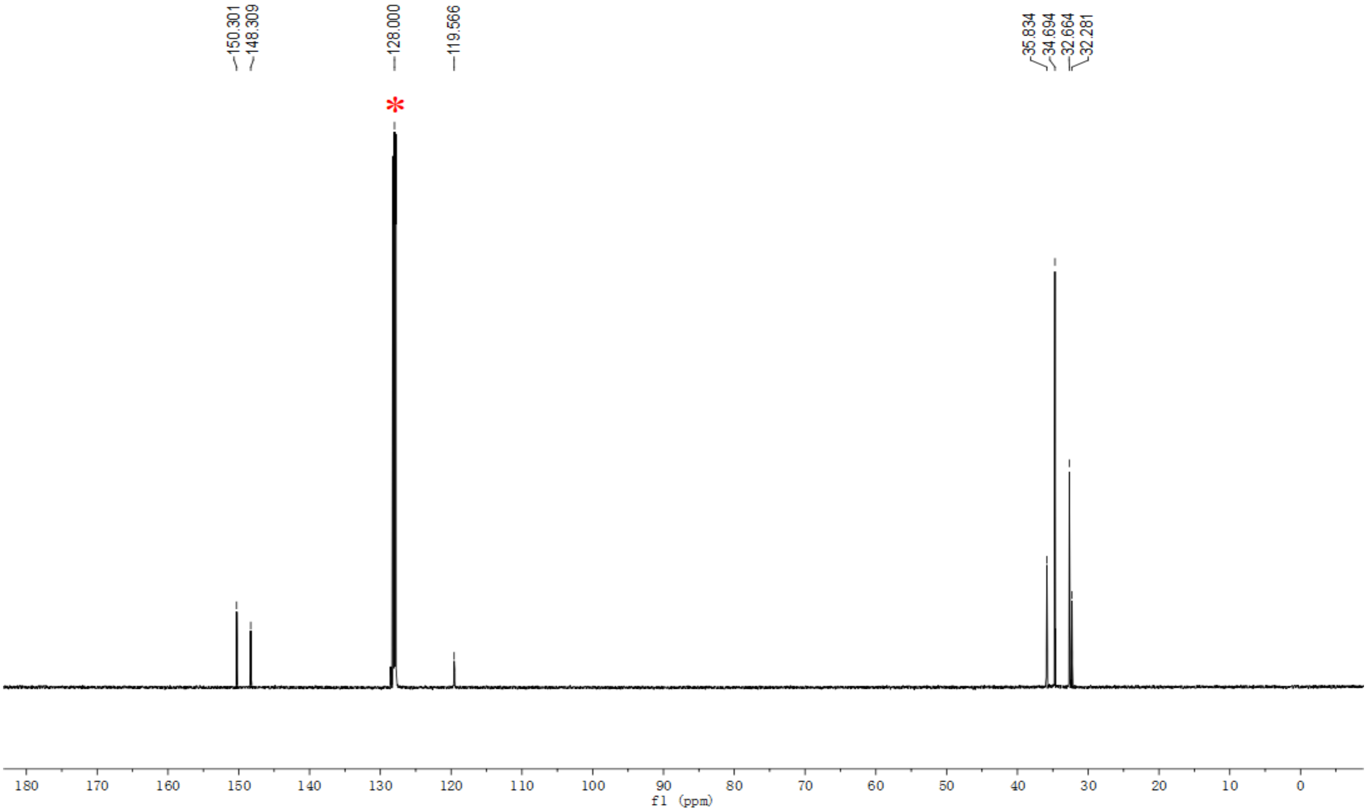

Figure S9. $^{13}C\{^{1}H\}$ NMR spectrum for compound **2** (* solvent).

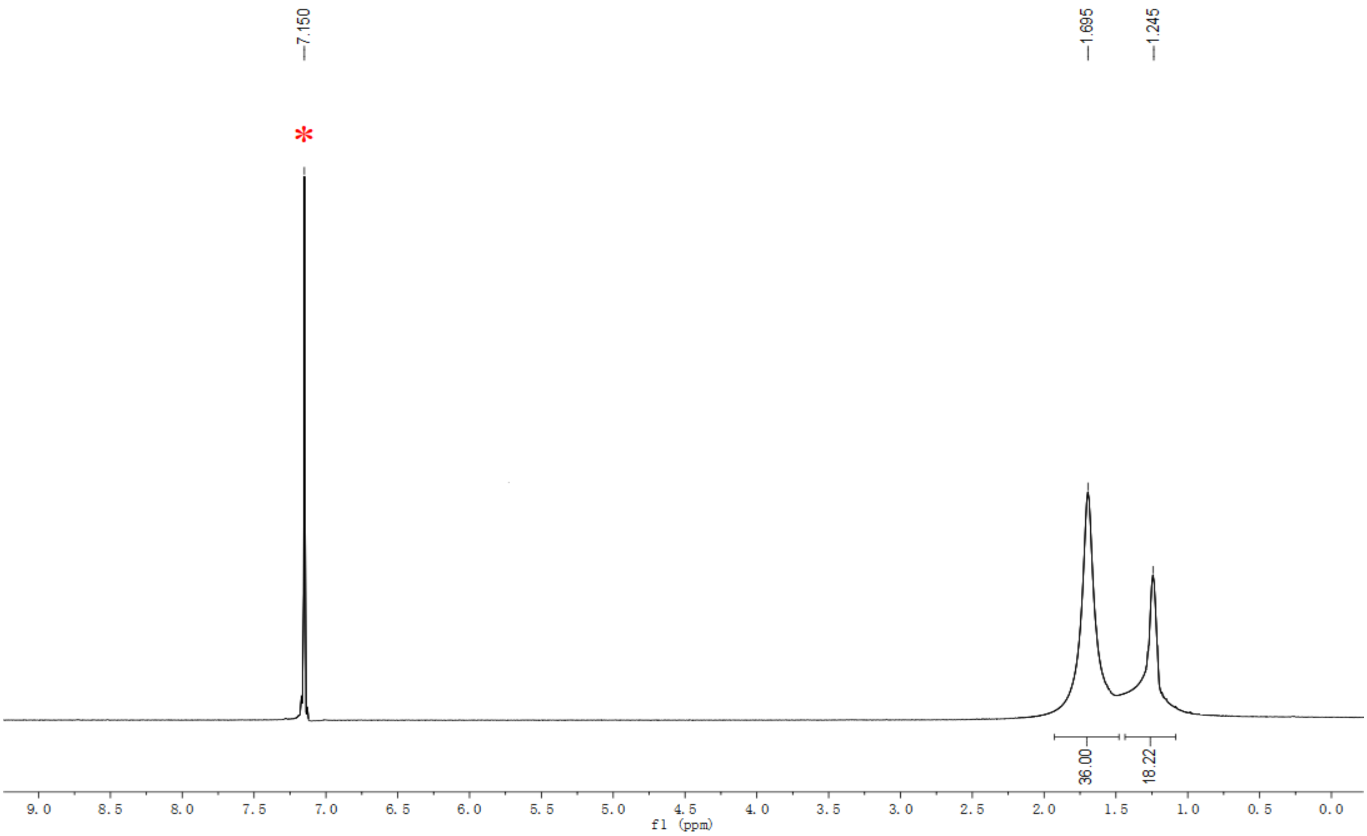

Figure S10. ^{1}H NMR spectrum for compound **3** (* solvent).

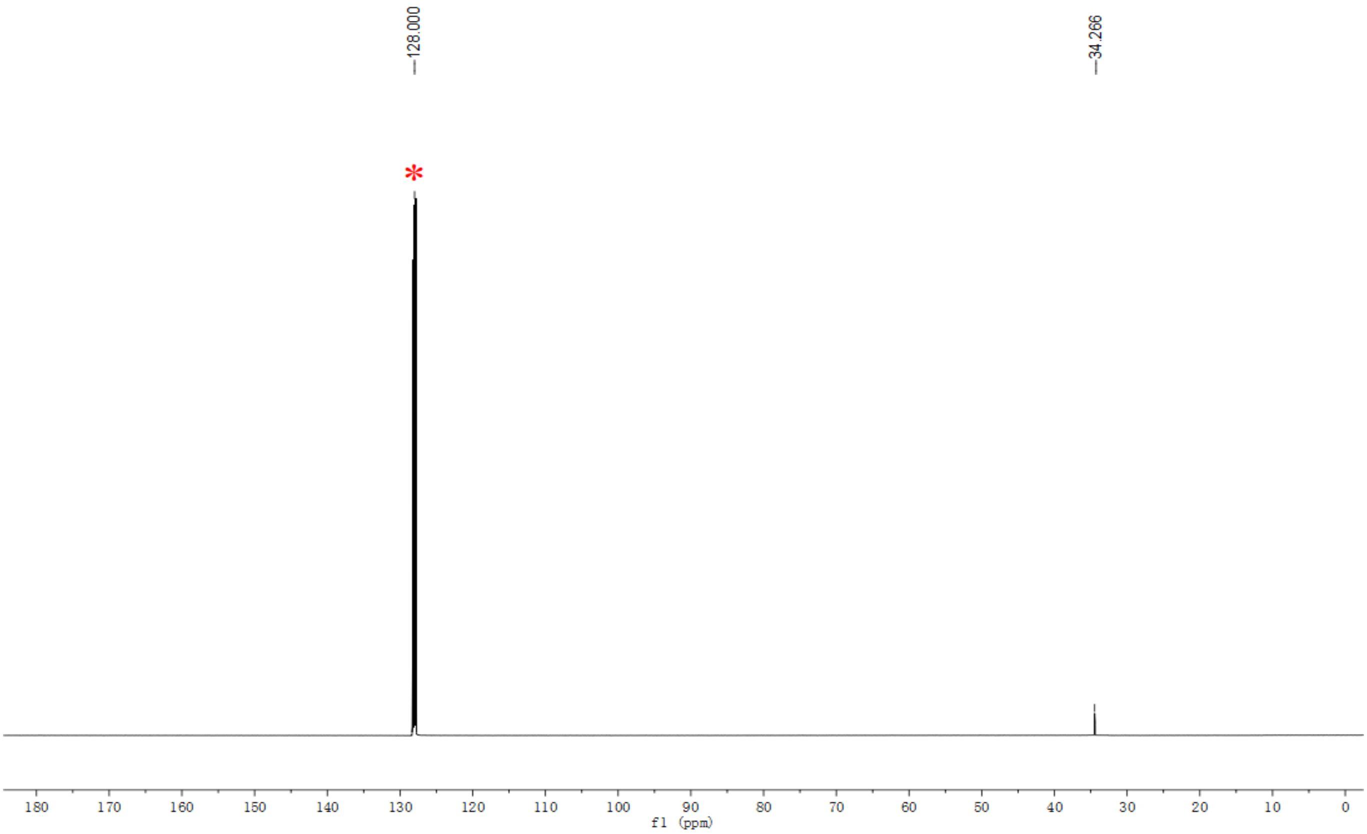

Figure S11. $^{13}C\{^{1}H\}$ NMR spectrum for compound **3** (* solvent).

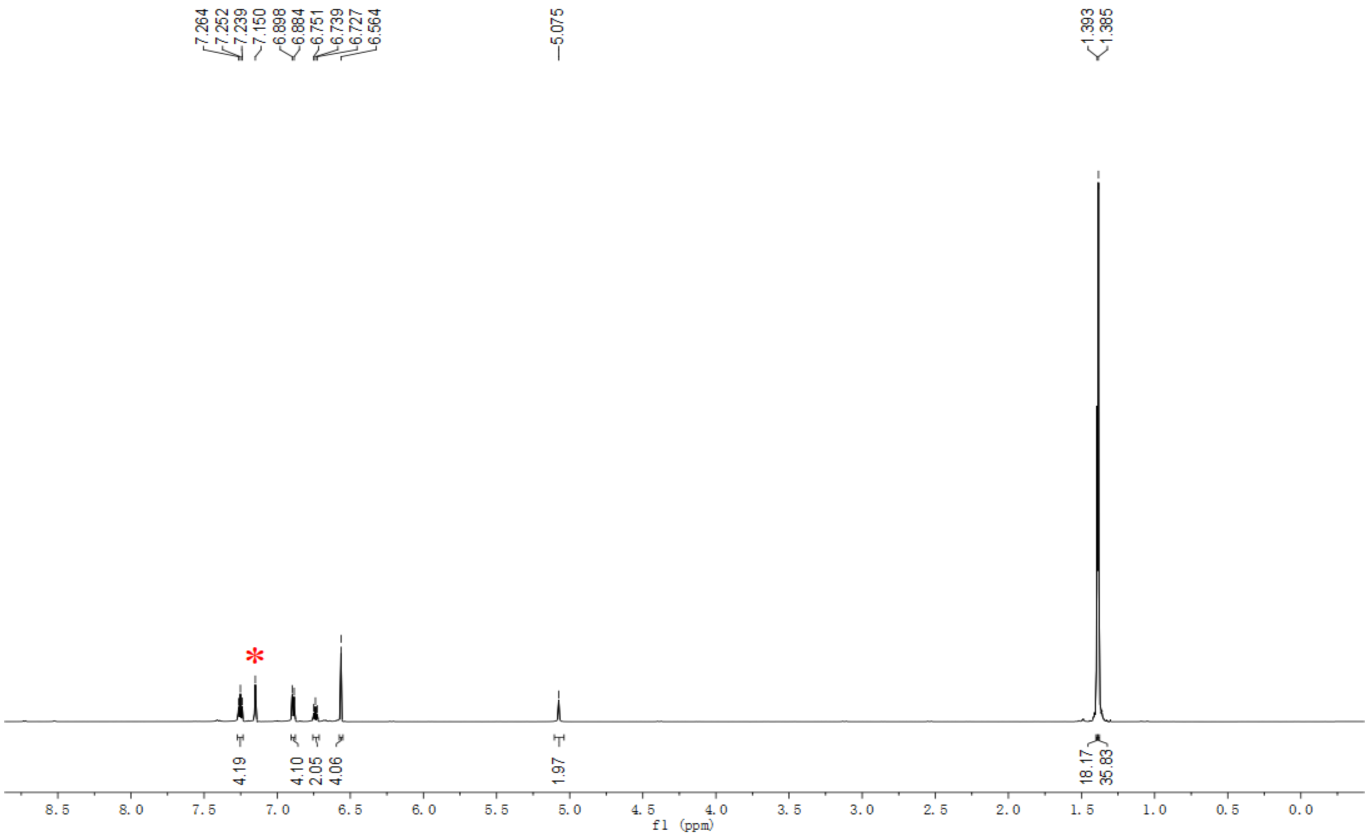

Figure S12. ^{1}H NMR spectrum for compound **4** (* solvent).

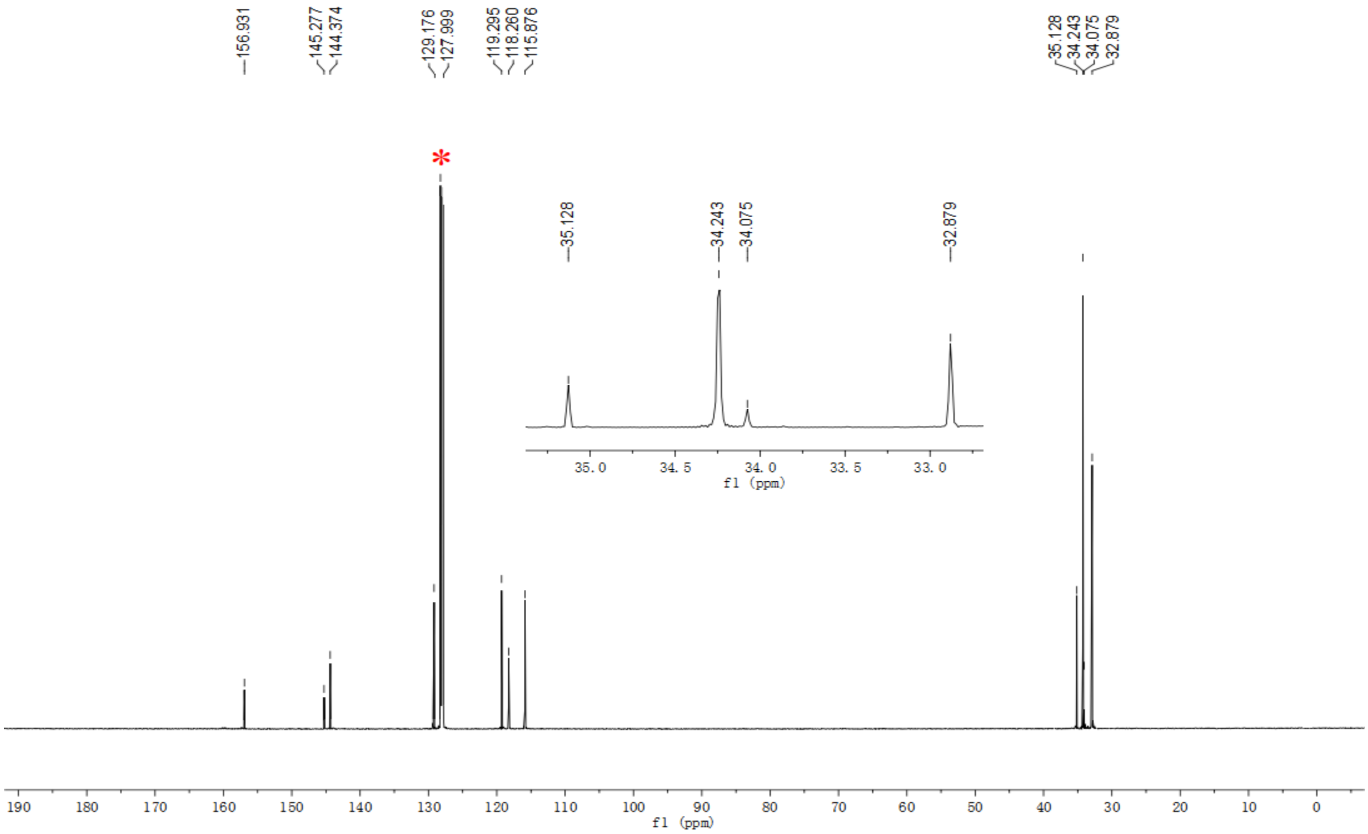

Figure S13. $^{13}C\{^{1}H\}$ NMR spectrum for compound **4** (* solvent).

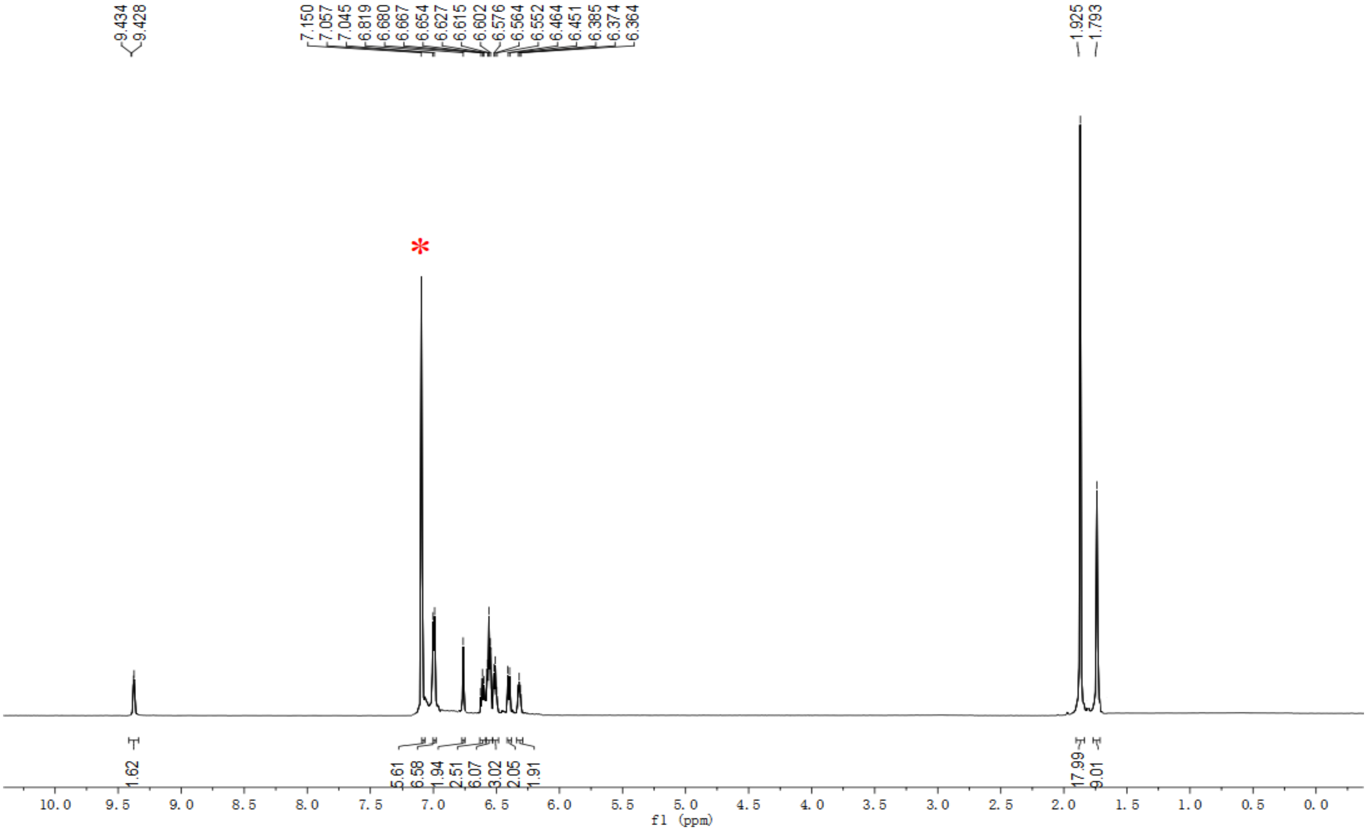

Figure S14. ^{1}H NMR spectrum for compound **5** (* solvent).

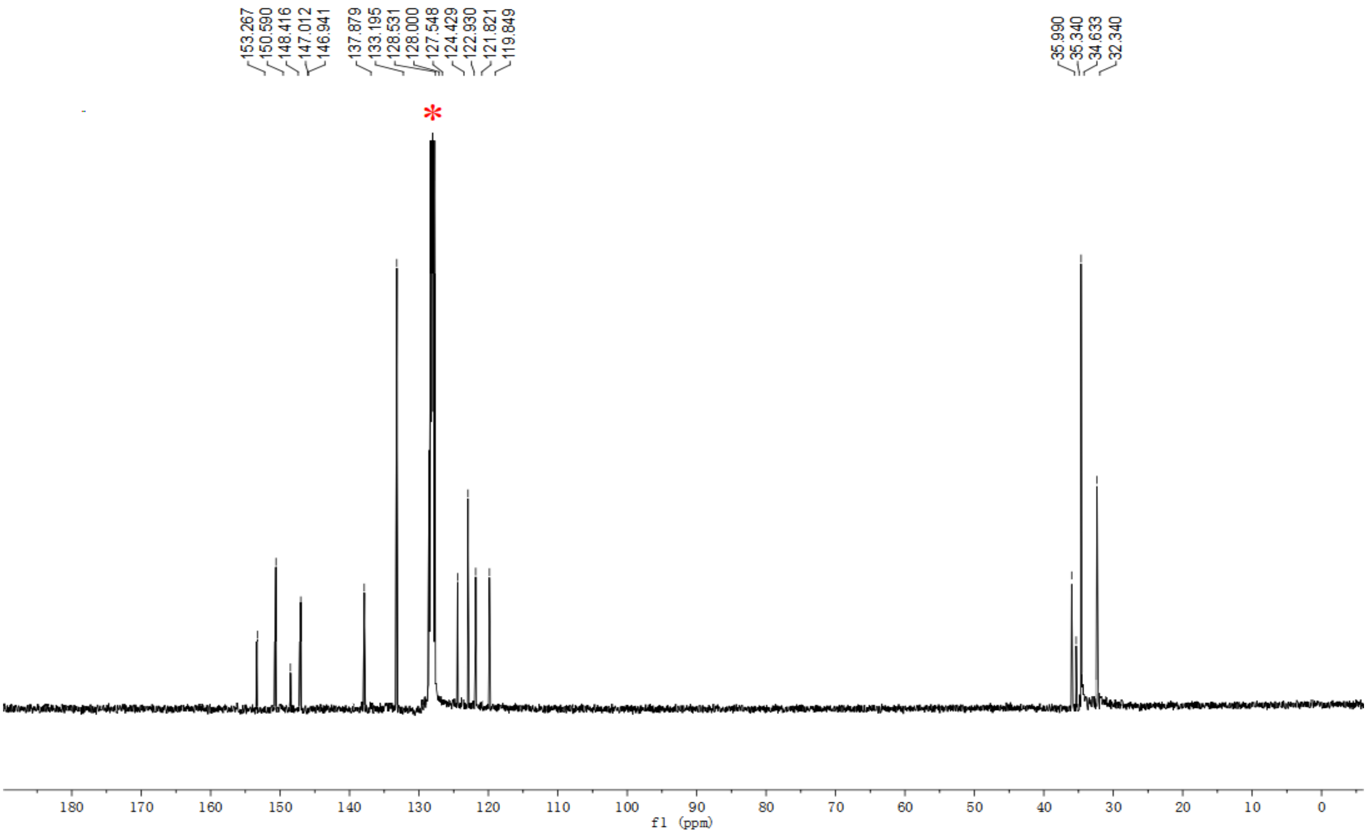

Figure S15. $^{13}C\{^{1}H\}$ NMR spectrum for compound **5** (* solvent).

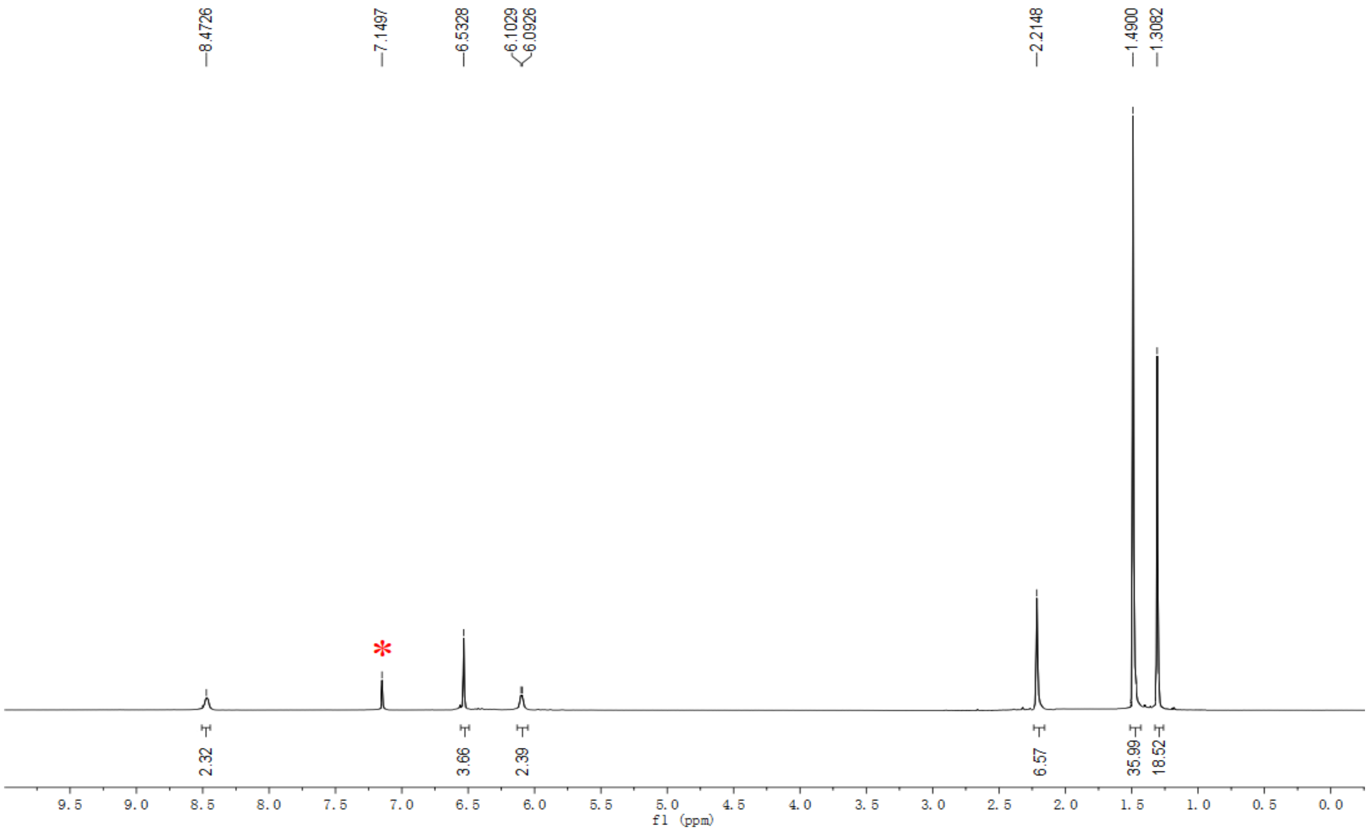

Figure S16. ^{1}H NMR spectrum for compound **6** (* solvent).

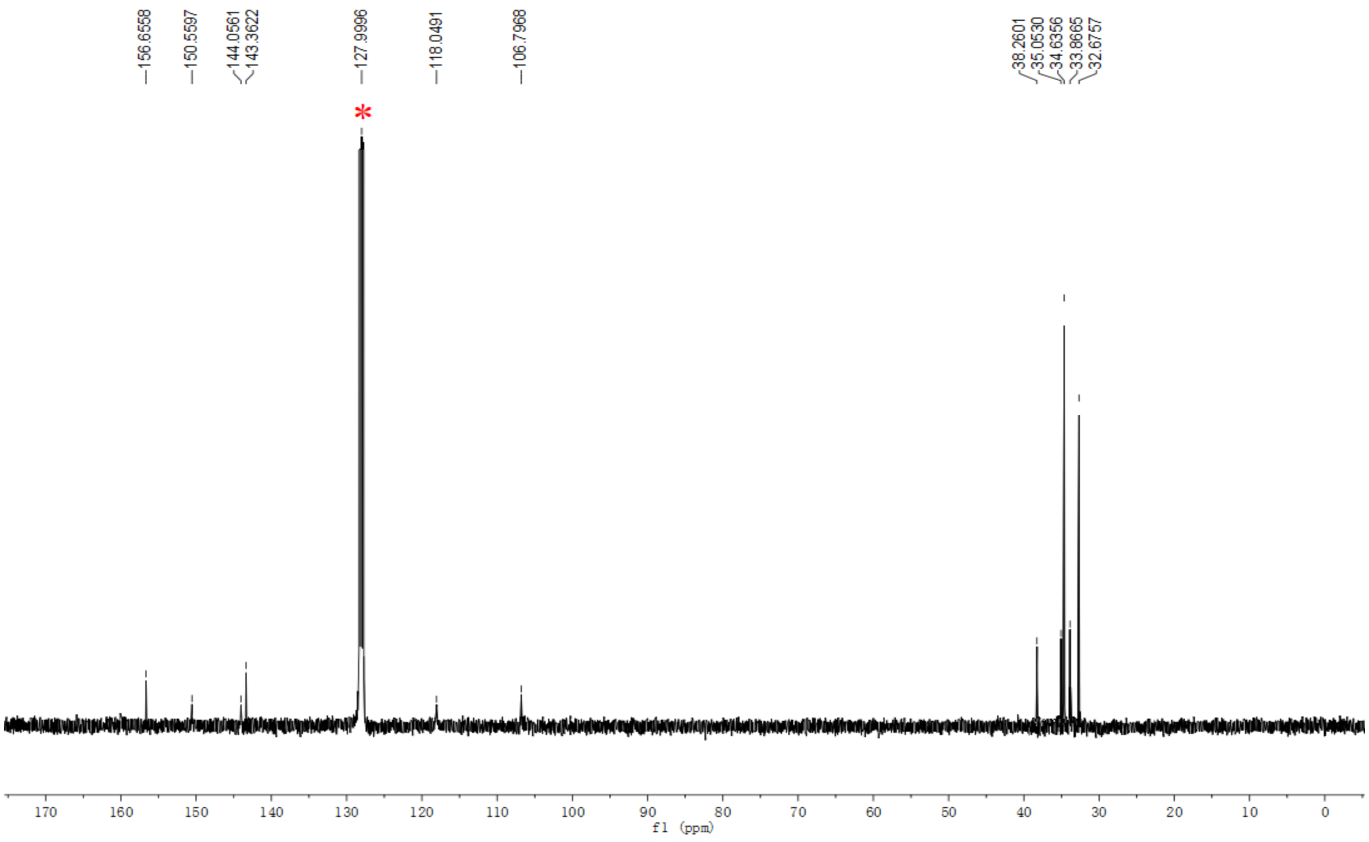

Figure S17. $^{13}C\{^{1}H\}$ NMR spectrum for compound **6** (* solvent).

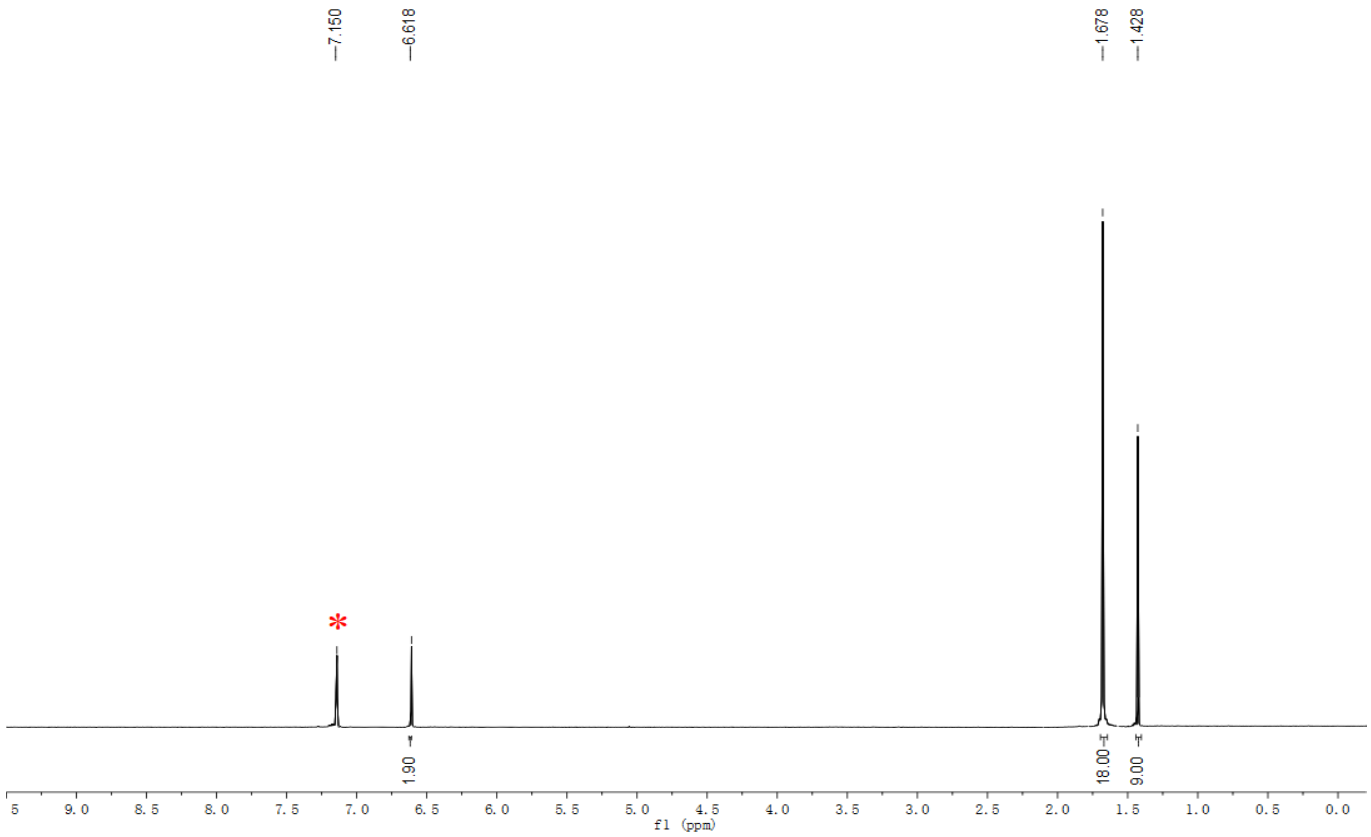

Figure S18. ^{1}H NMR spectrum for compound **7** (* solvent).

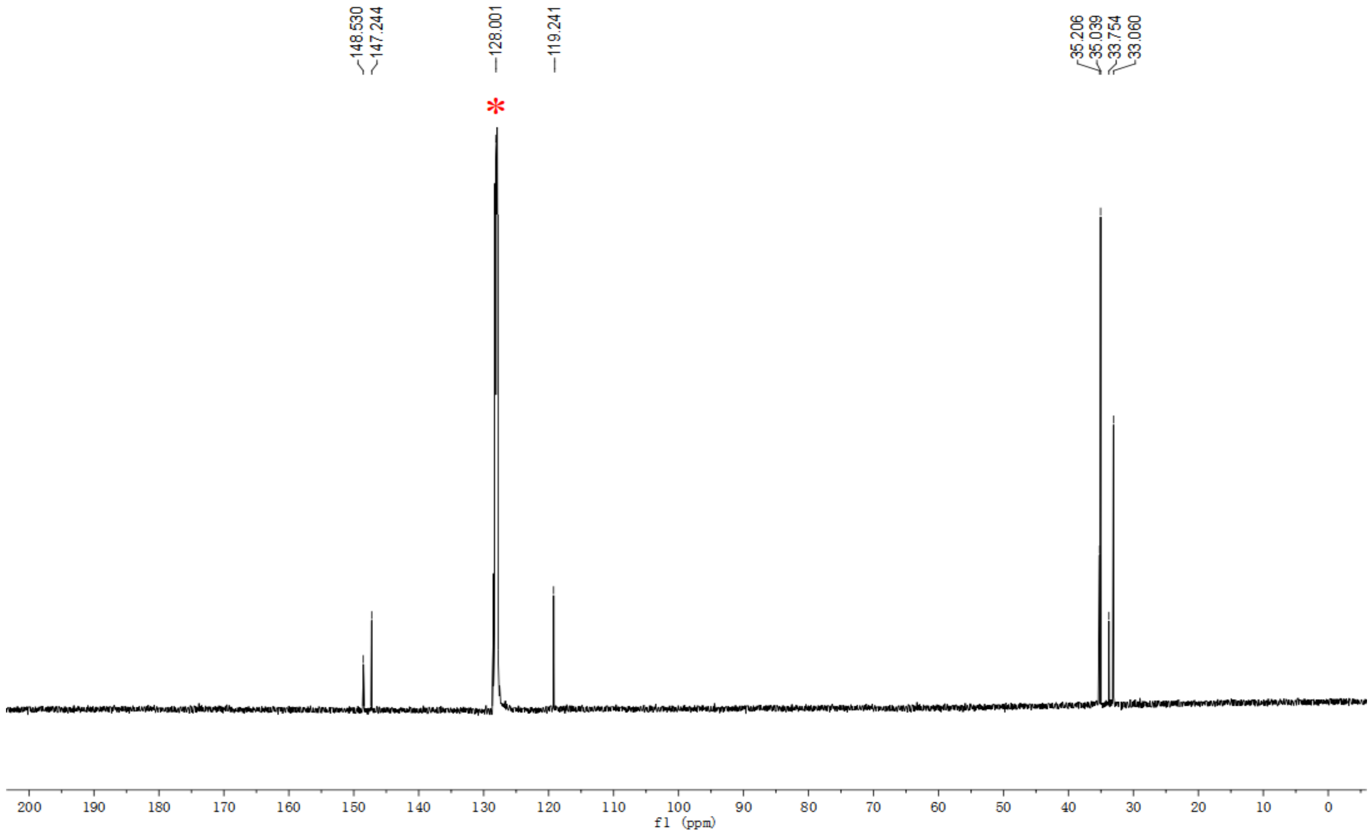

Figure S19. $^{13}C\{^{1}H\}$ NMR spectrum for compound **7** (* solvent).

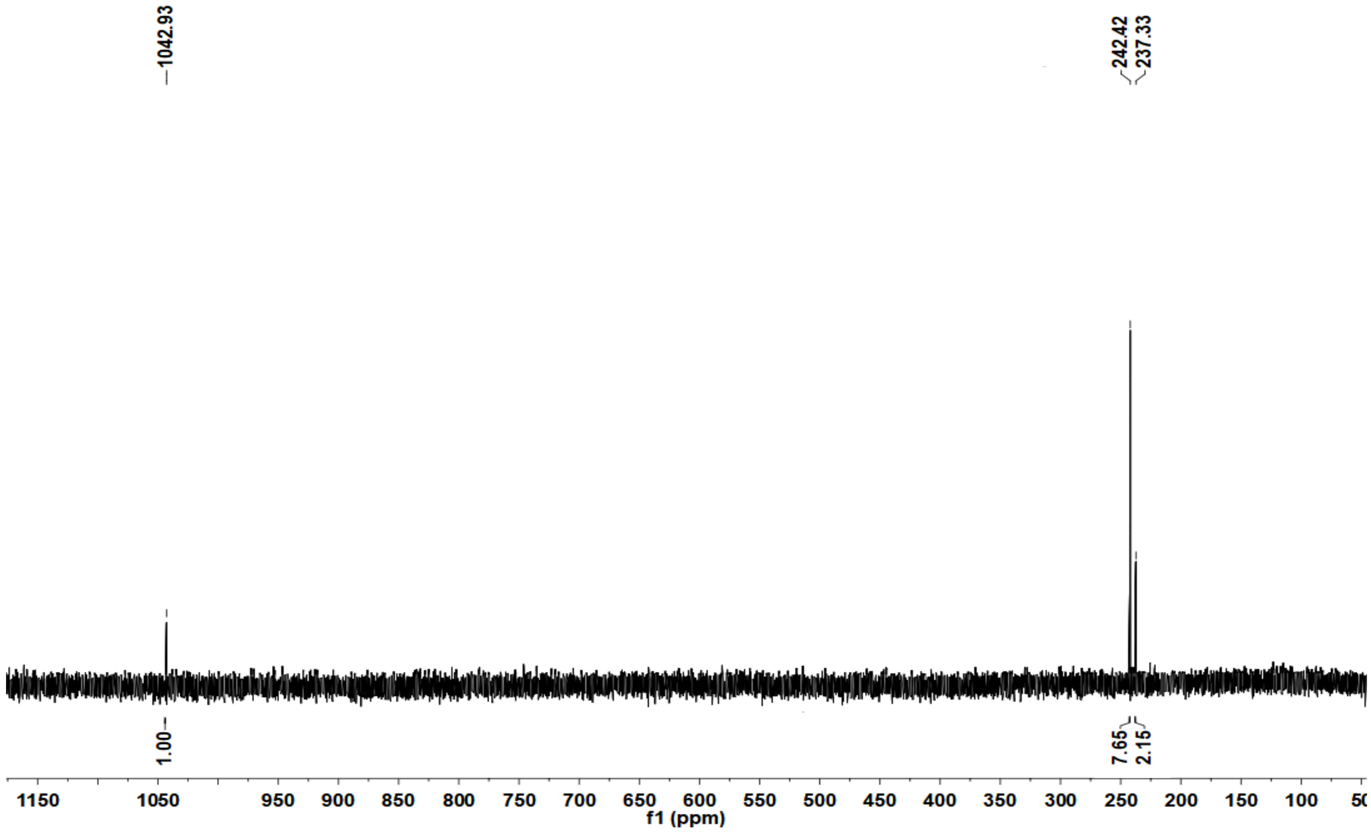

Figure S20. $^{77}Se\{^{1}H\}$ NMR spectrum for compound **7**.

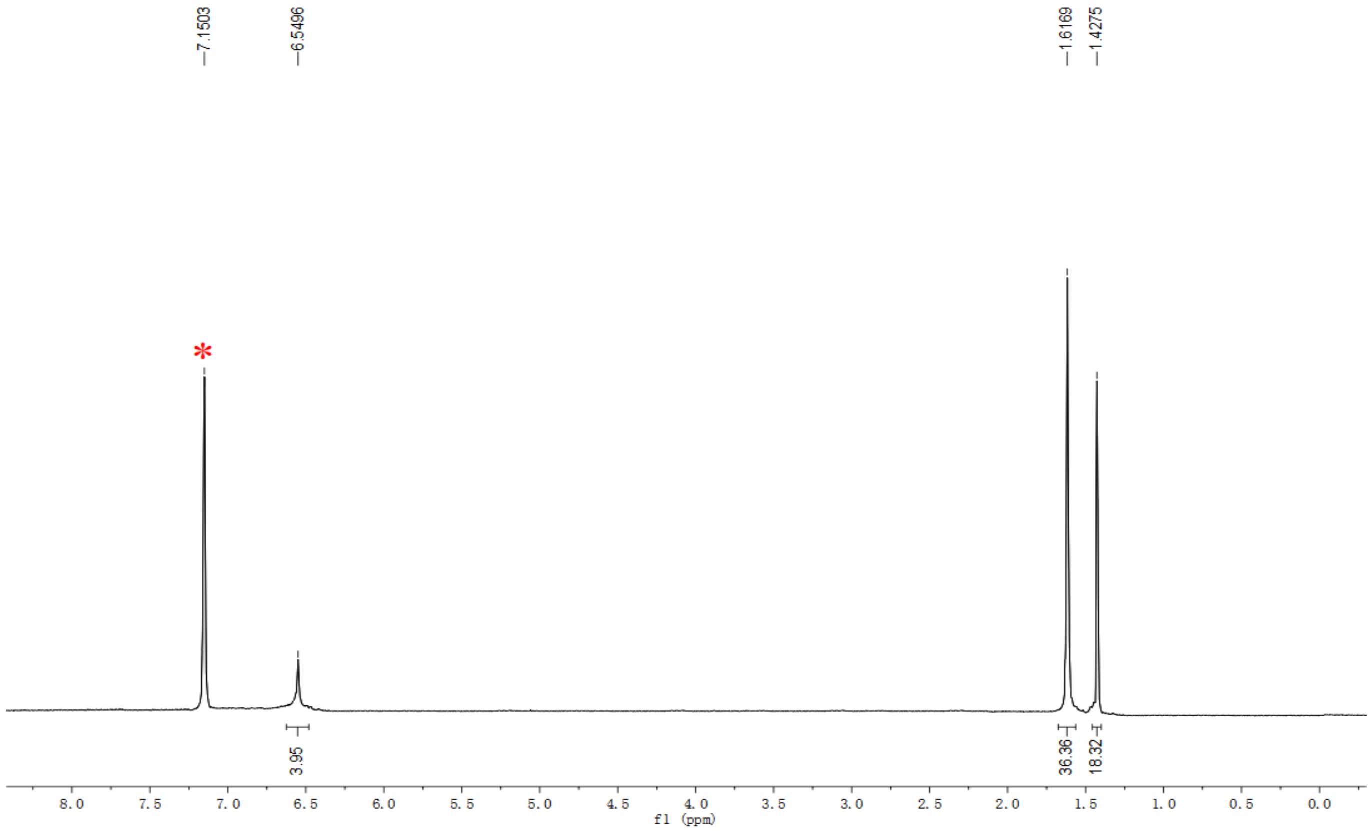

Figure S21. ^{1}H NMR spectrum for compound **8** (* solvent).

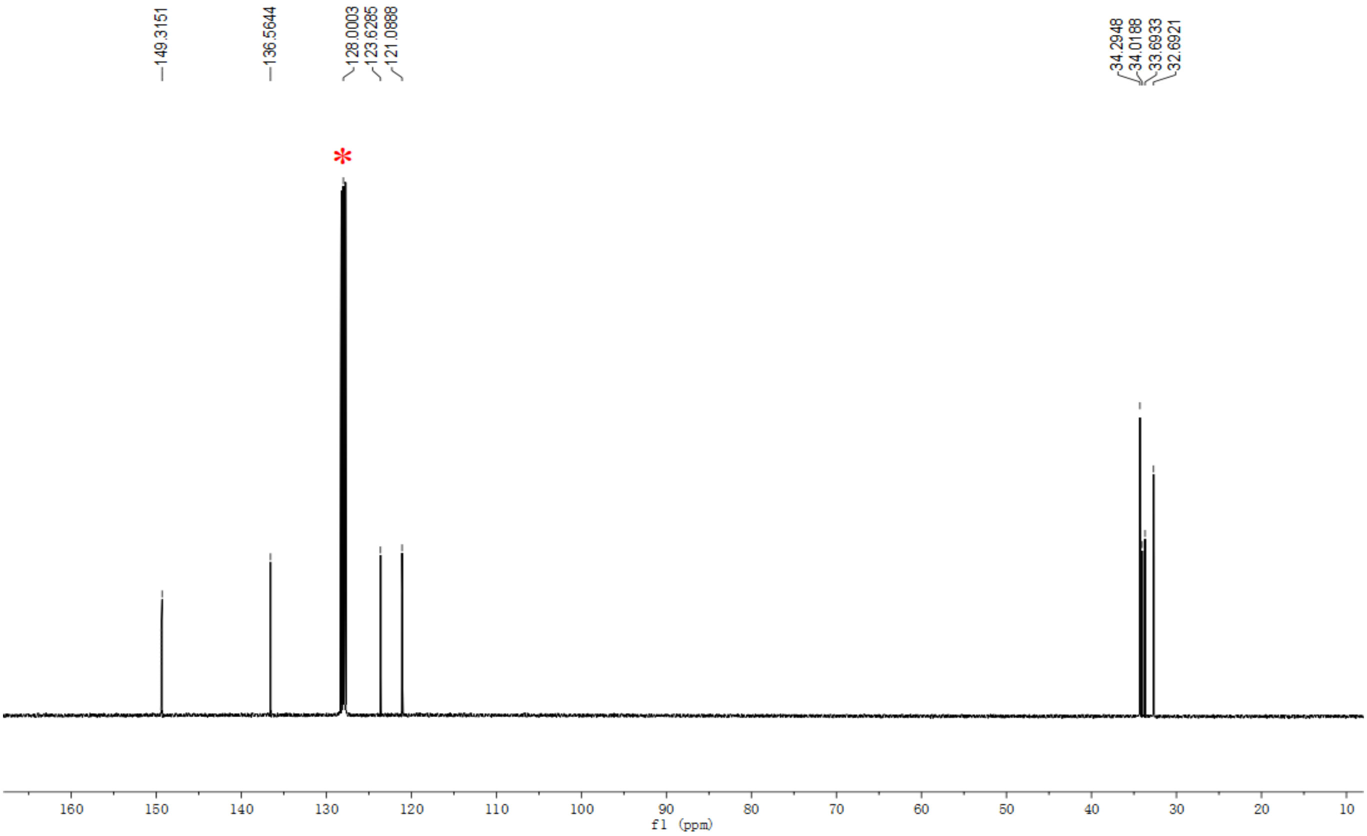

Figure S22. $^{13}C\{^{1}H\}$ NMR spectrum for compound **8** (* solvent).

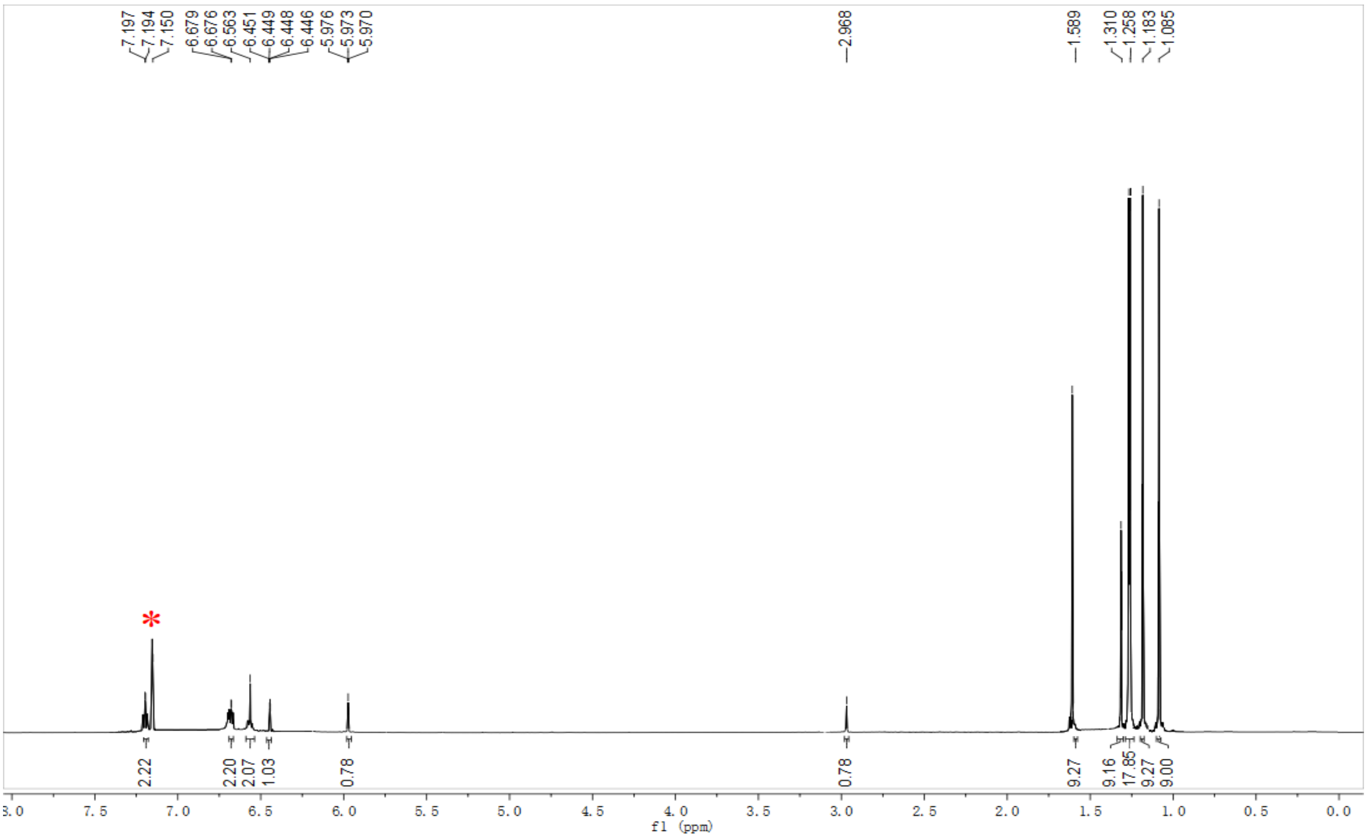

Figure S23. ^{1}H NMR spectrum for compound **9** (* solvent).

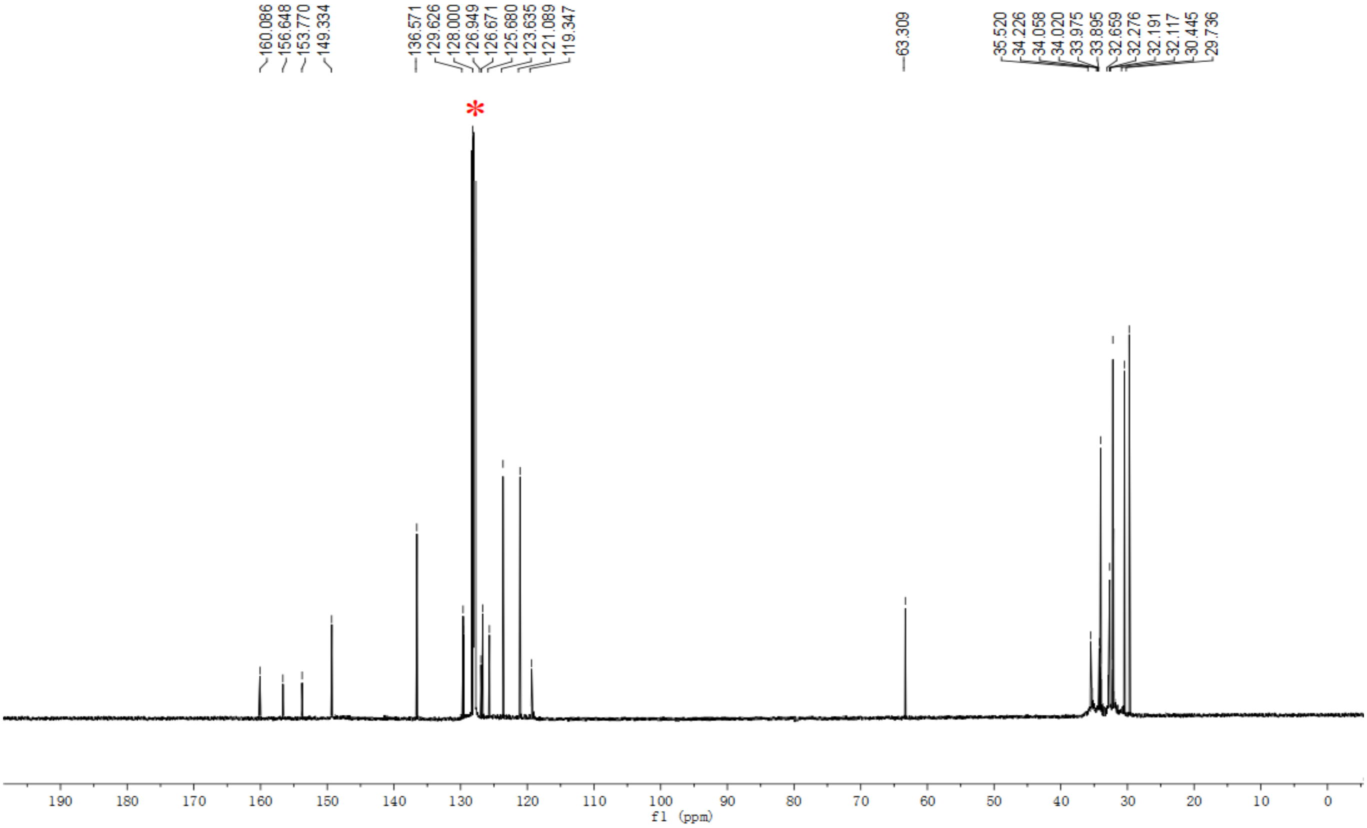

Figure S24. $^{13}C\{^{1}H\}$ NMR spectrum for compound **9** (* solvent).

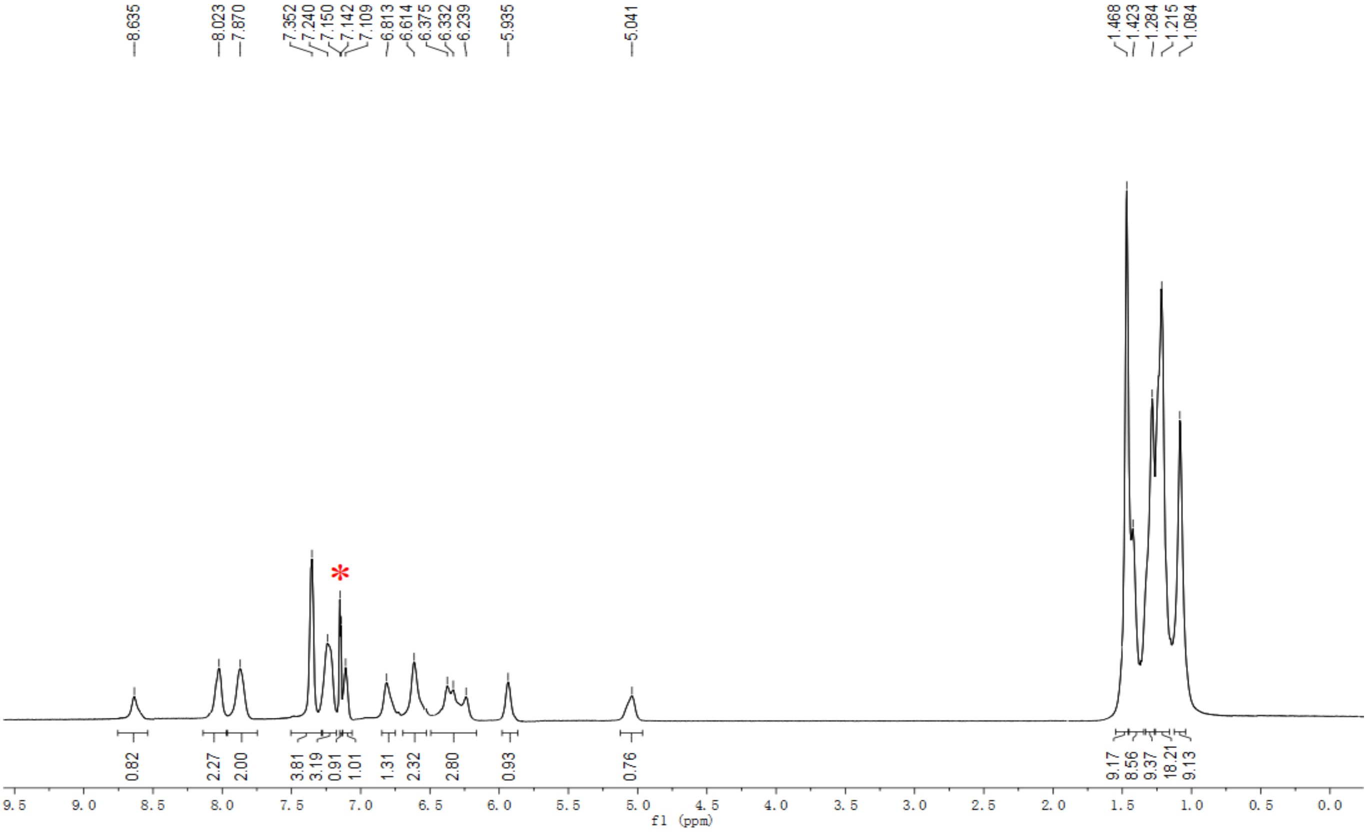

Figure S25. ^{1}H NMR spectrum for compound **10** (* solvent).

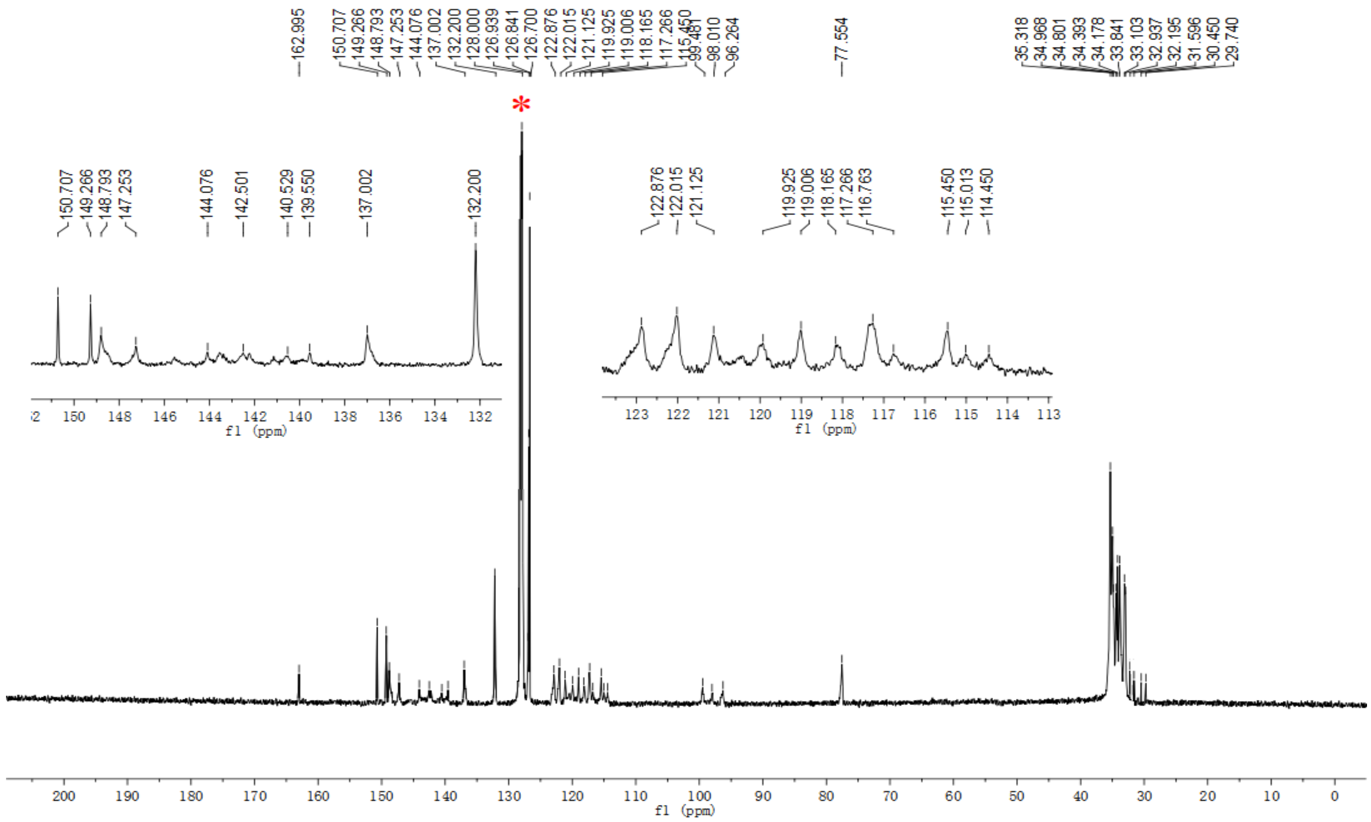

Figure S26. $^{13}C\{^1H\}$ NMR spectrum for compound **10** (* solvent).

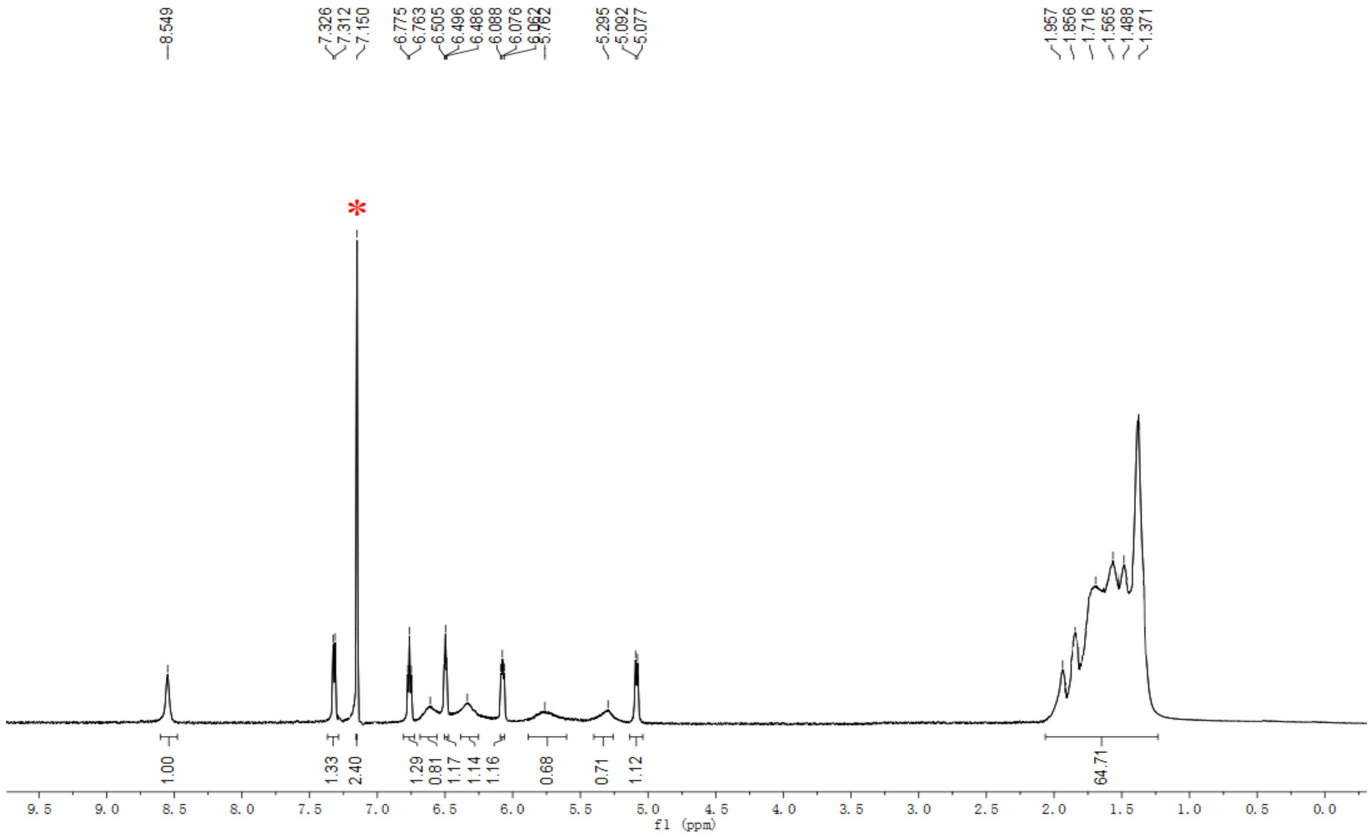

Figure S27. ^1H NMR spectrum for compound **11** (* solvent).

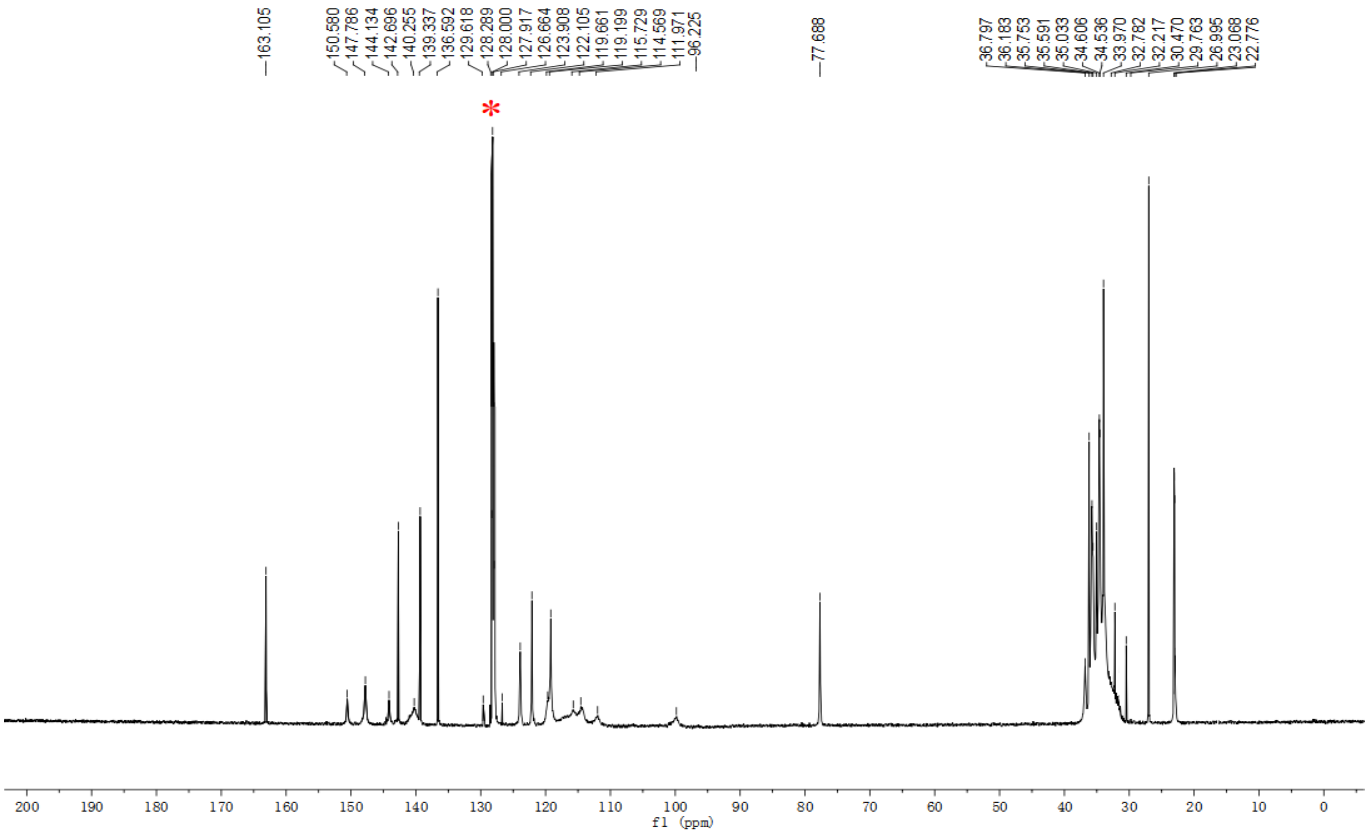

Figure S28. $^{13}C\{^{1}H\}$ NMR spectrum for compound **11** (* solvent).

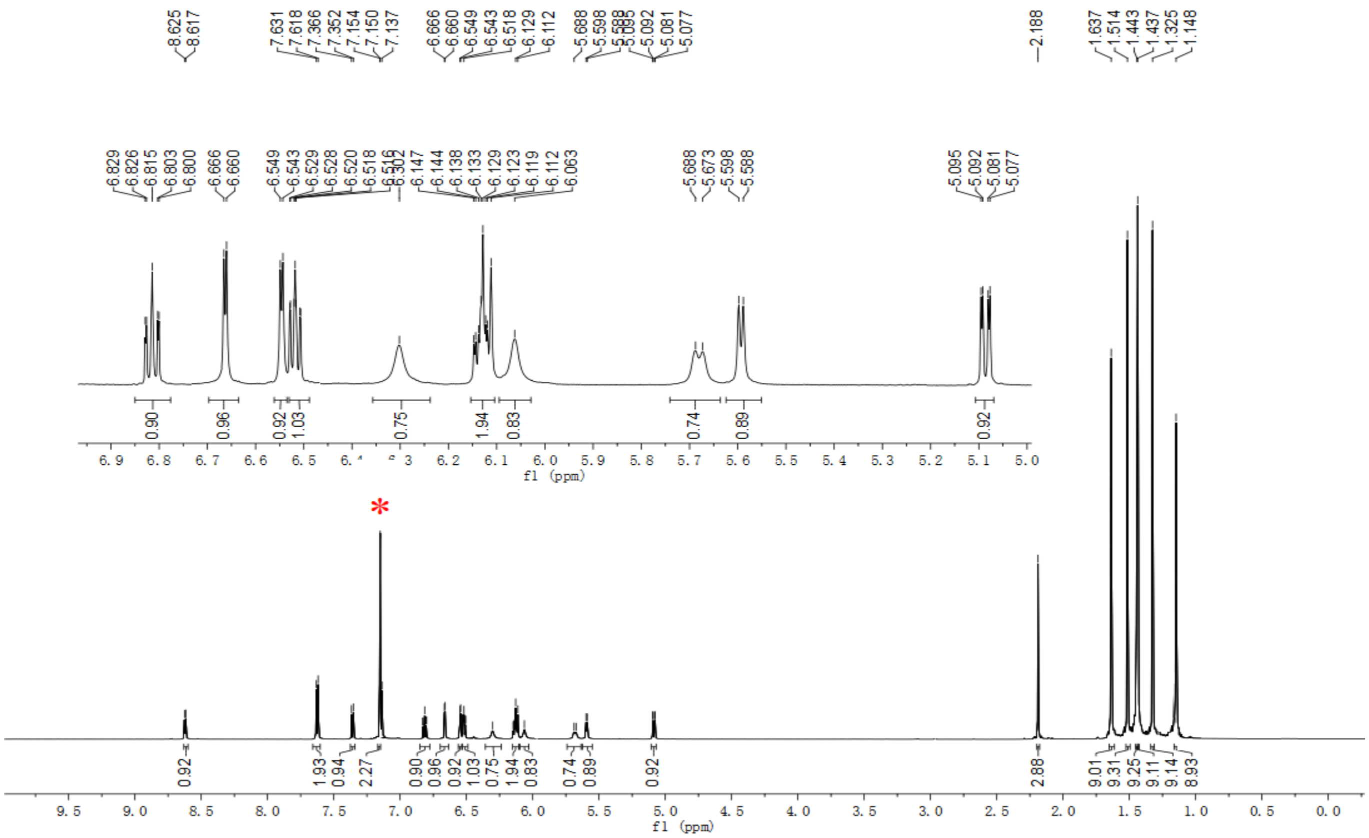

Figure S29. ^{1}H NMR spectrum for compound **12** (* solvent).

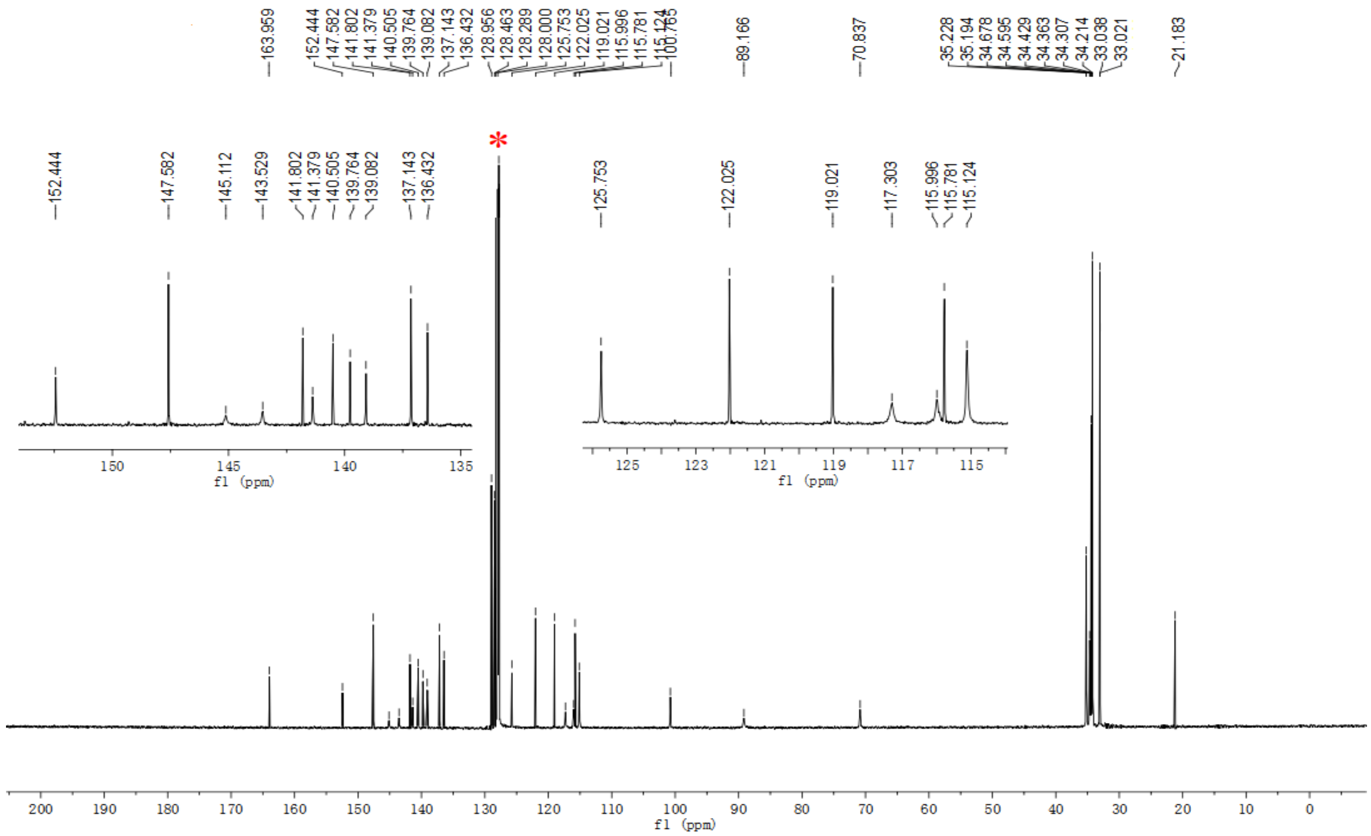

Figure S30. $^{13}C\{^{1}H\}$ NMR spectrum for compound **12** (* solvent).

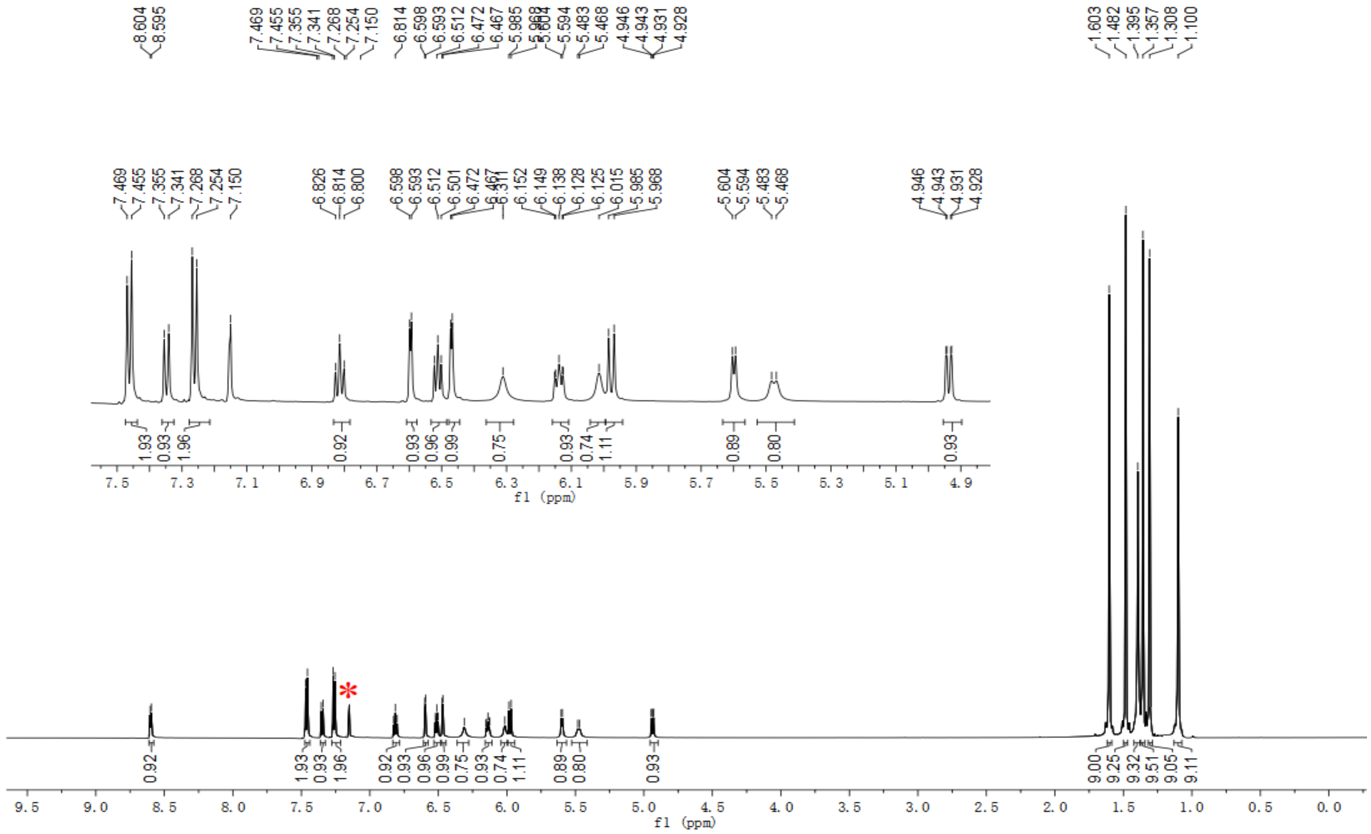

Figure S31. ^{1}H NMR spectrum for compound **13** (* solvent).

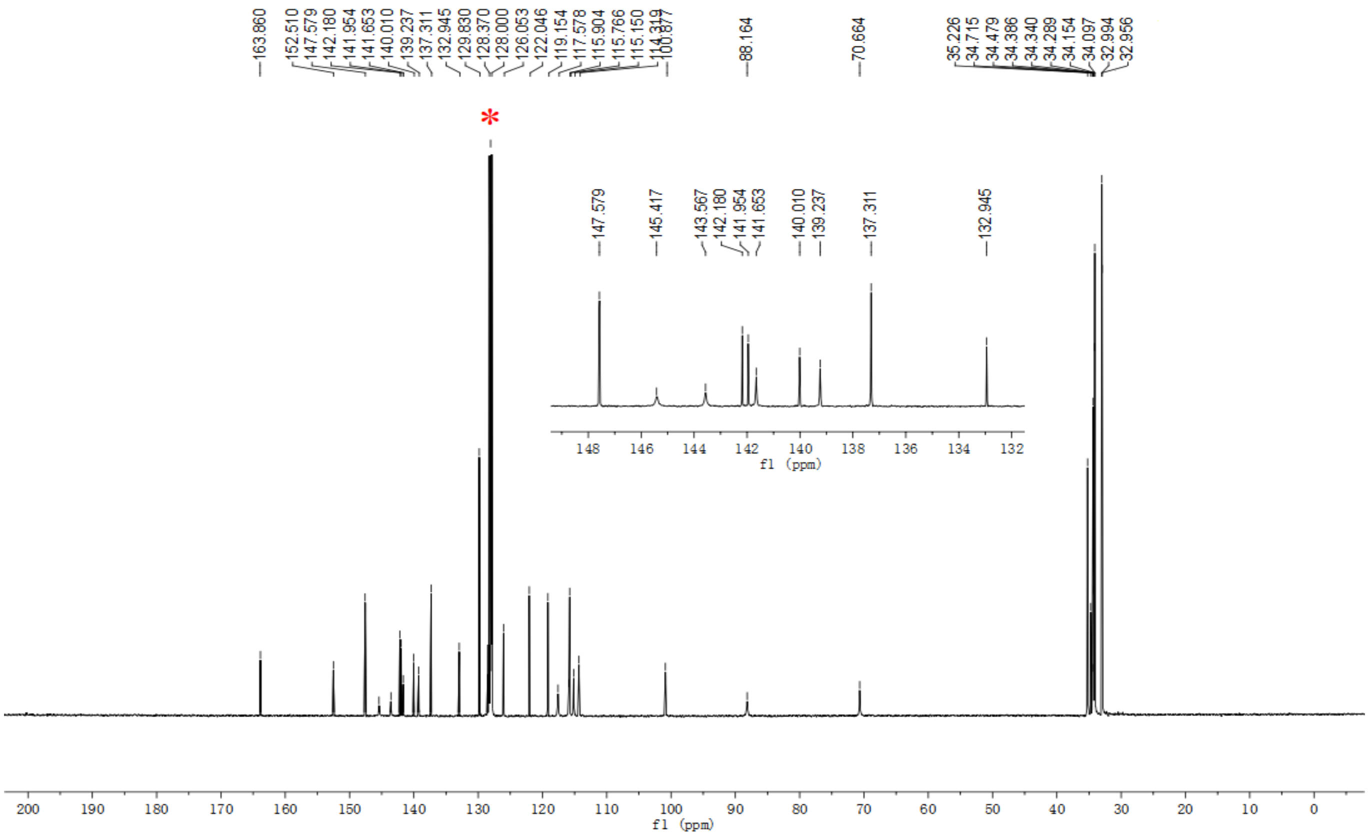

Figure S32. $^{13}C\{^{1}H\}$ NMR spectrum for compound **13** (* solvent).

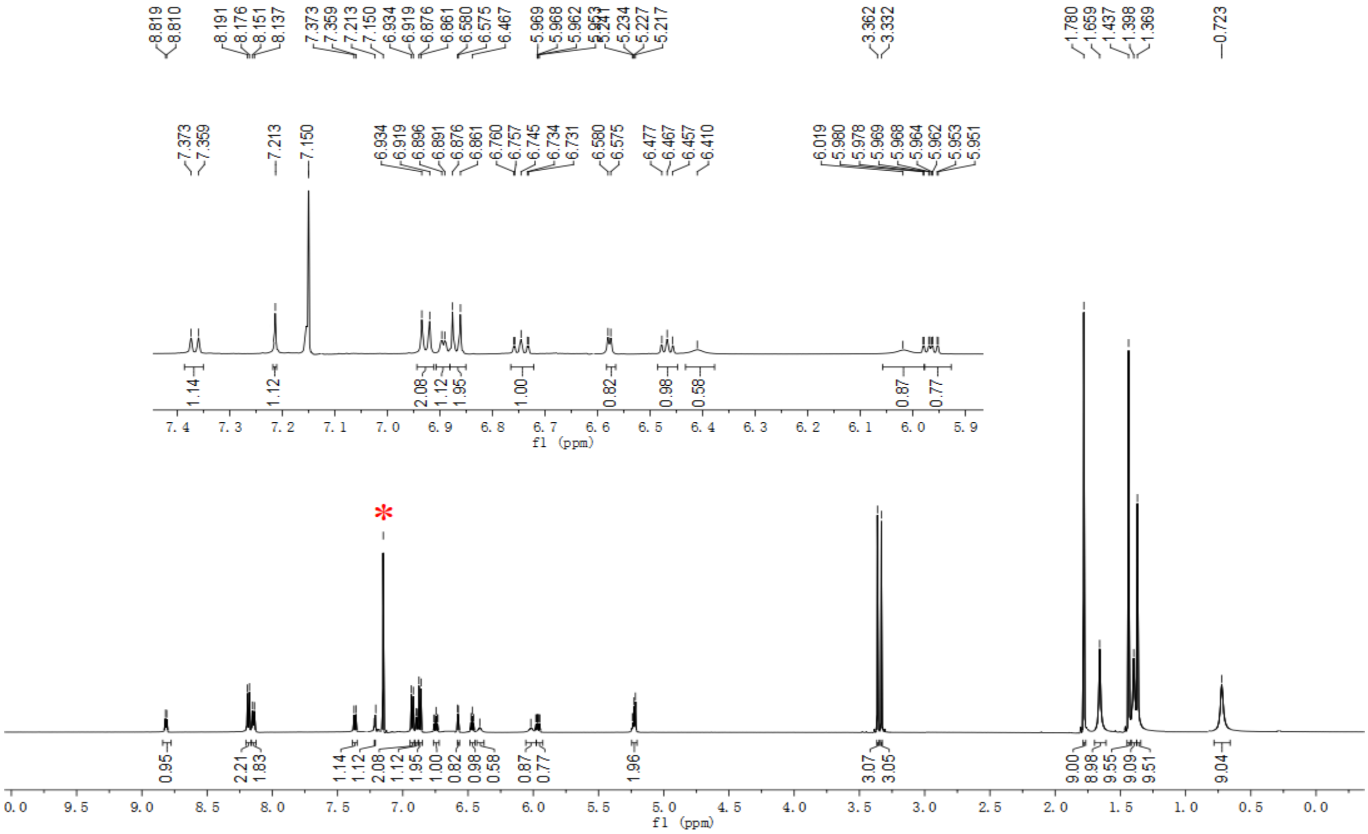

Figure S33. ^{1}H NMR spectrum for compound **14** (* solvent).

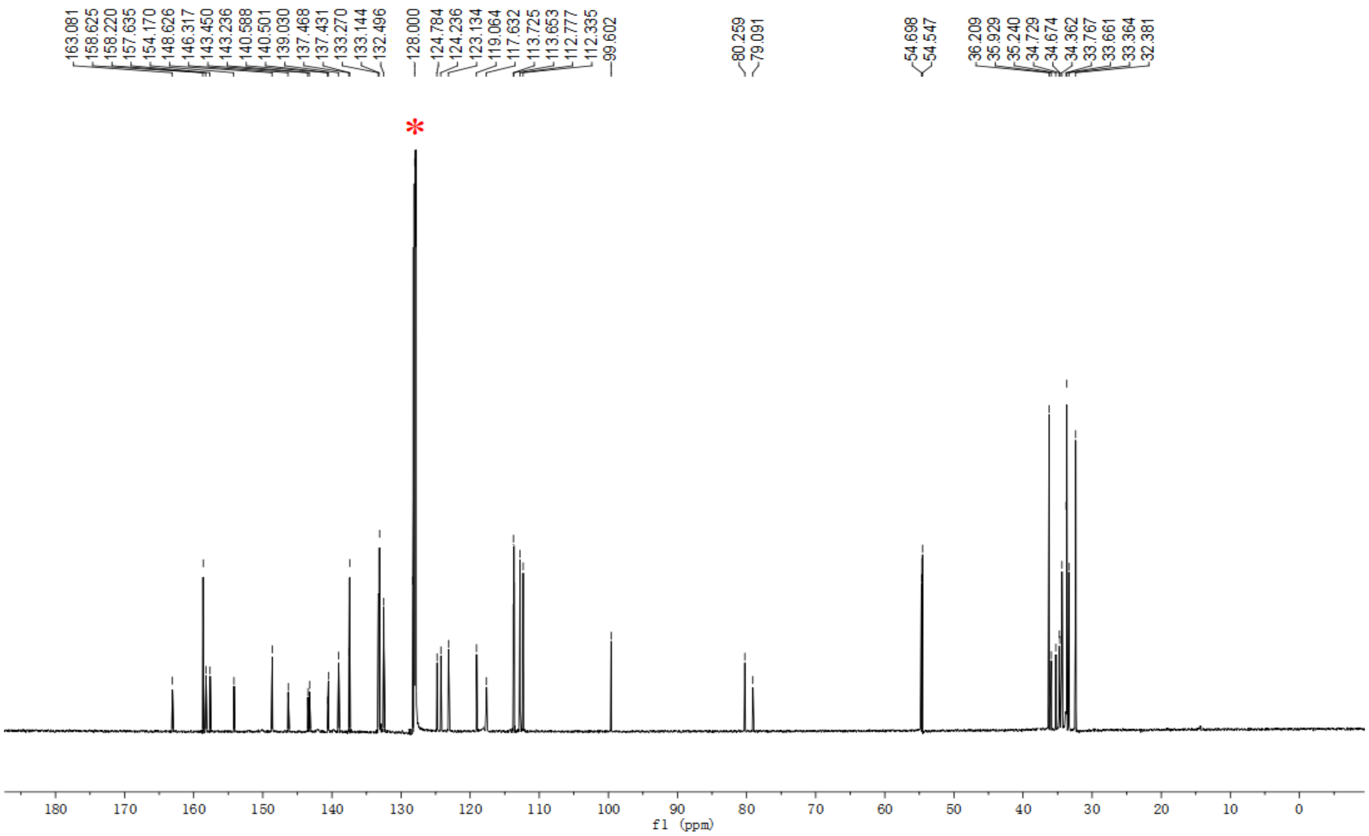

Figure S34. $^{13}C\{^{1}H\}$ NMR spectrum for compound **14** (* solvent).

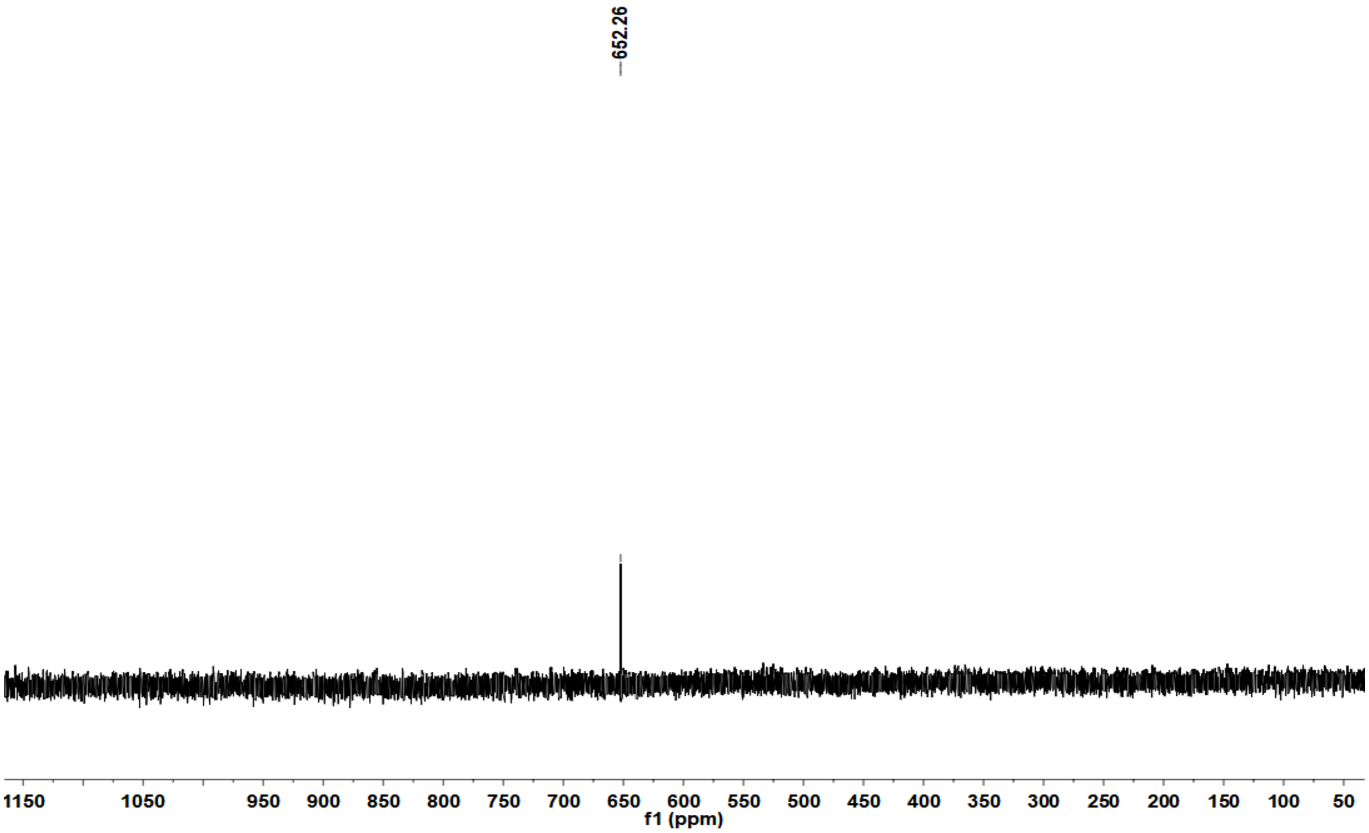

Figure S35. $^{77}Se\{^{1}H\}$ NMR spectrum for compound **14**.

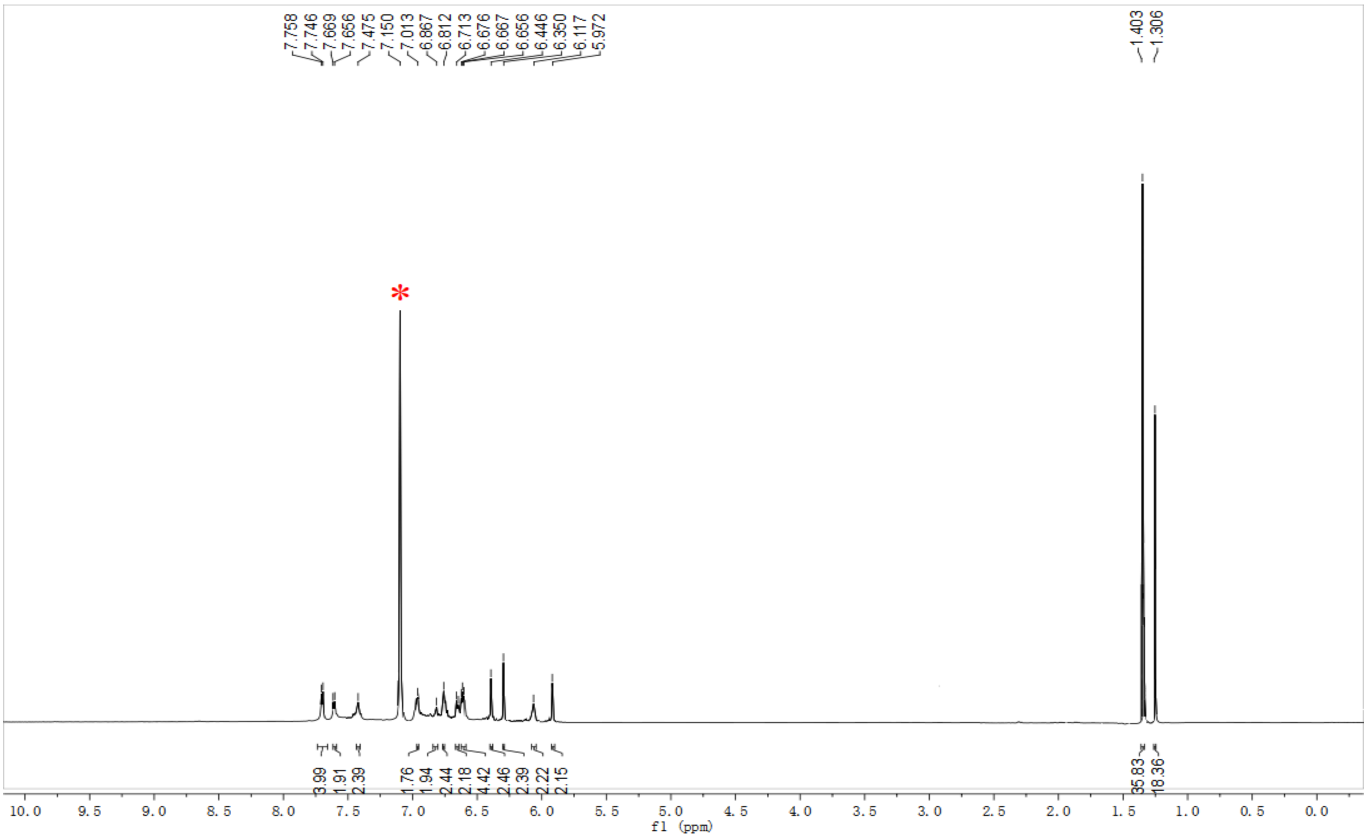

Figure S36. ^{1}H NMR spectrum for compound **15** (* solvent).

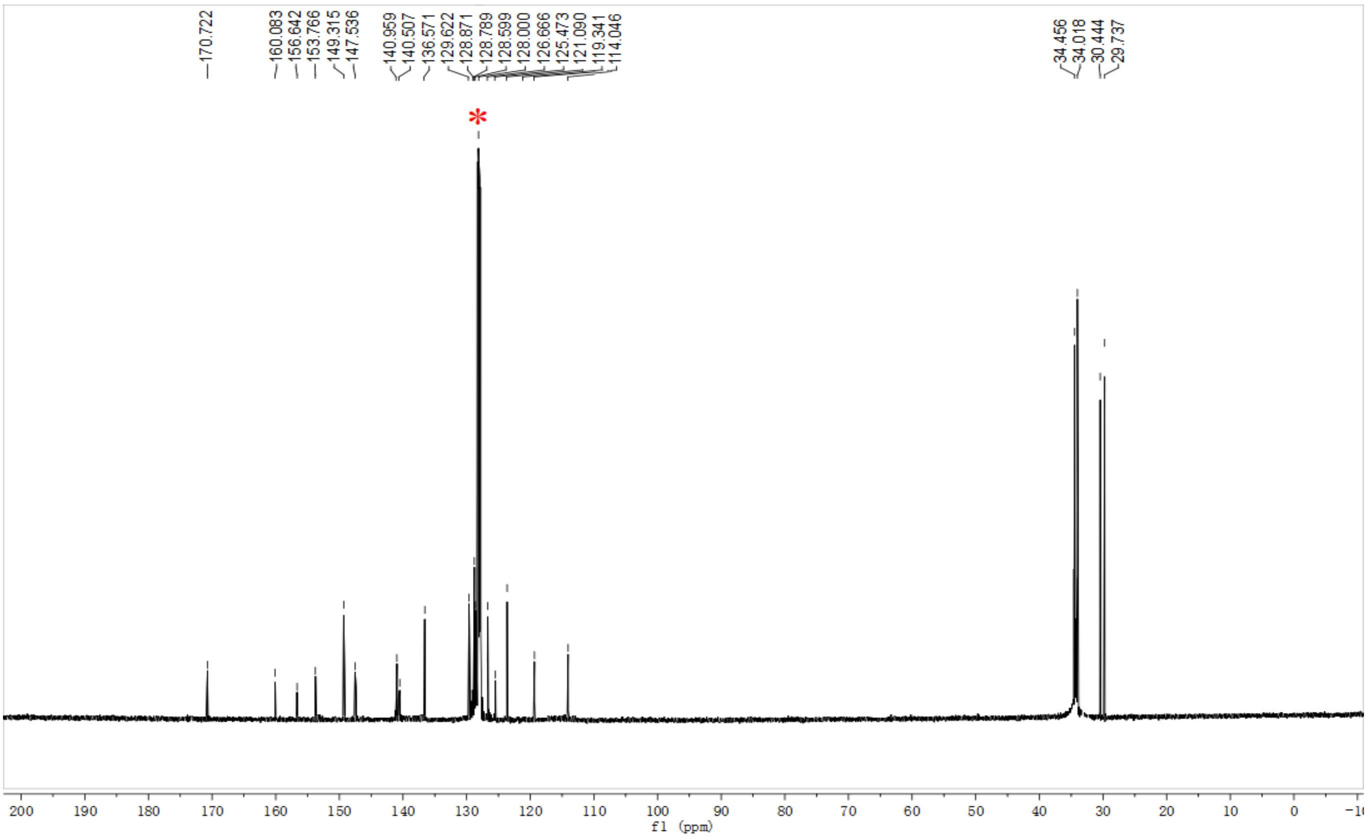

Figure S37. $^{13}C\{^{1}H\}$ NMR spectrum for compound **15** (* solvent).

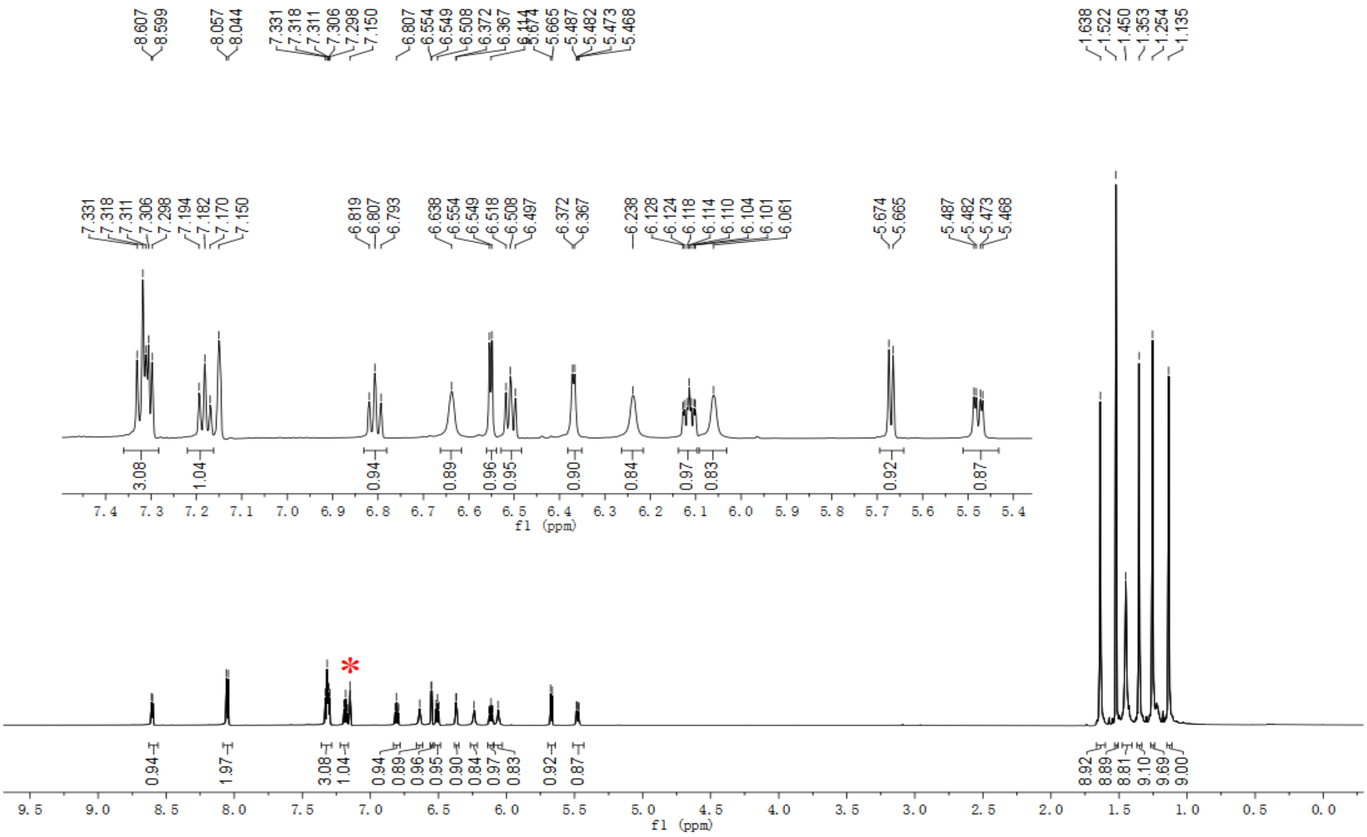

Figure S38. ^{1}H NMR spectrum for compound **16** (* solvent).

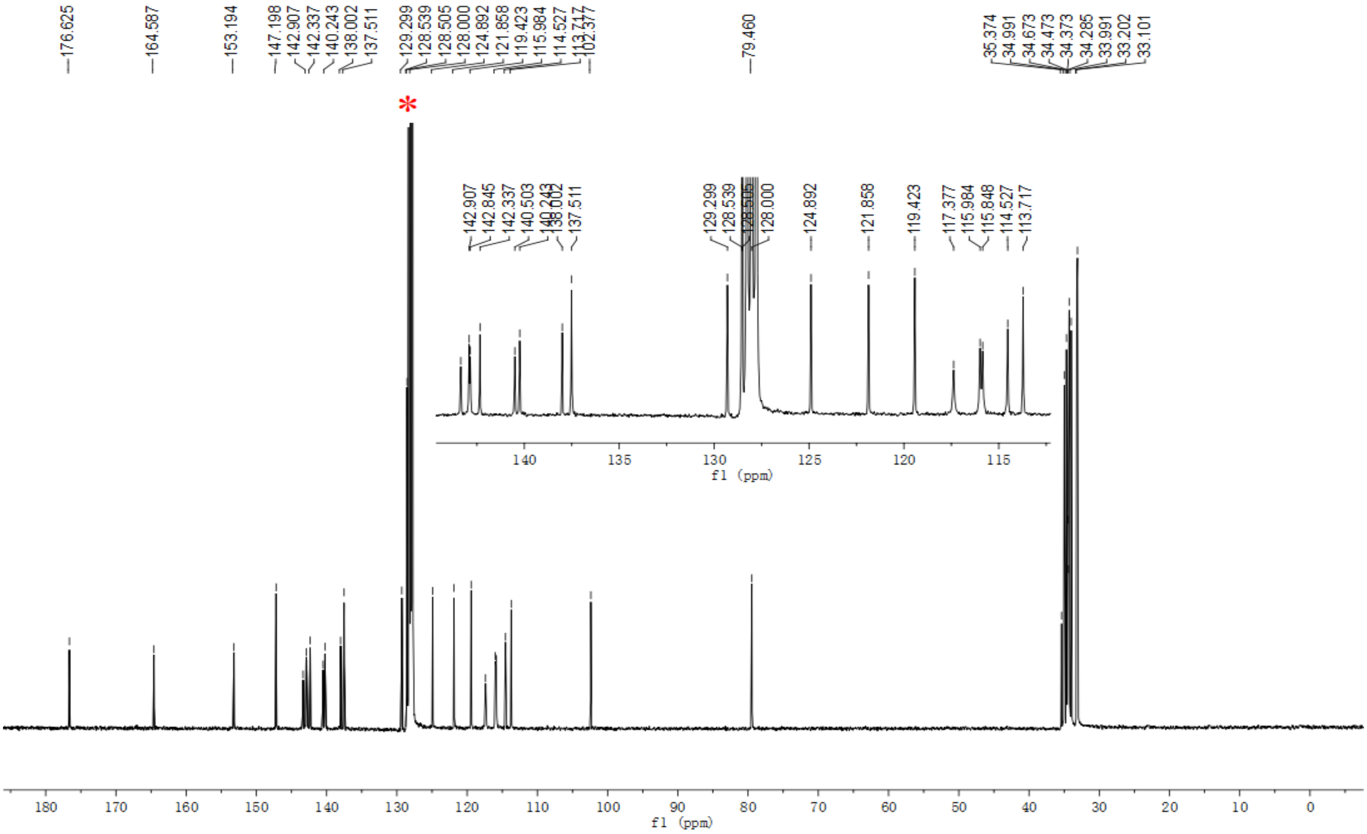

Figure S39. $^{13}C\{^{1}H\}$ NMR spectrum for compound **16** (* solvent).

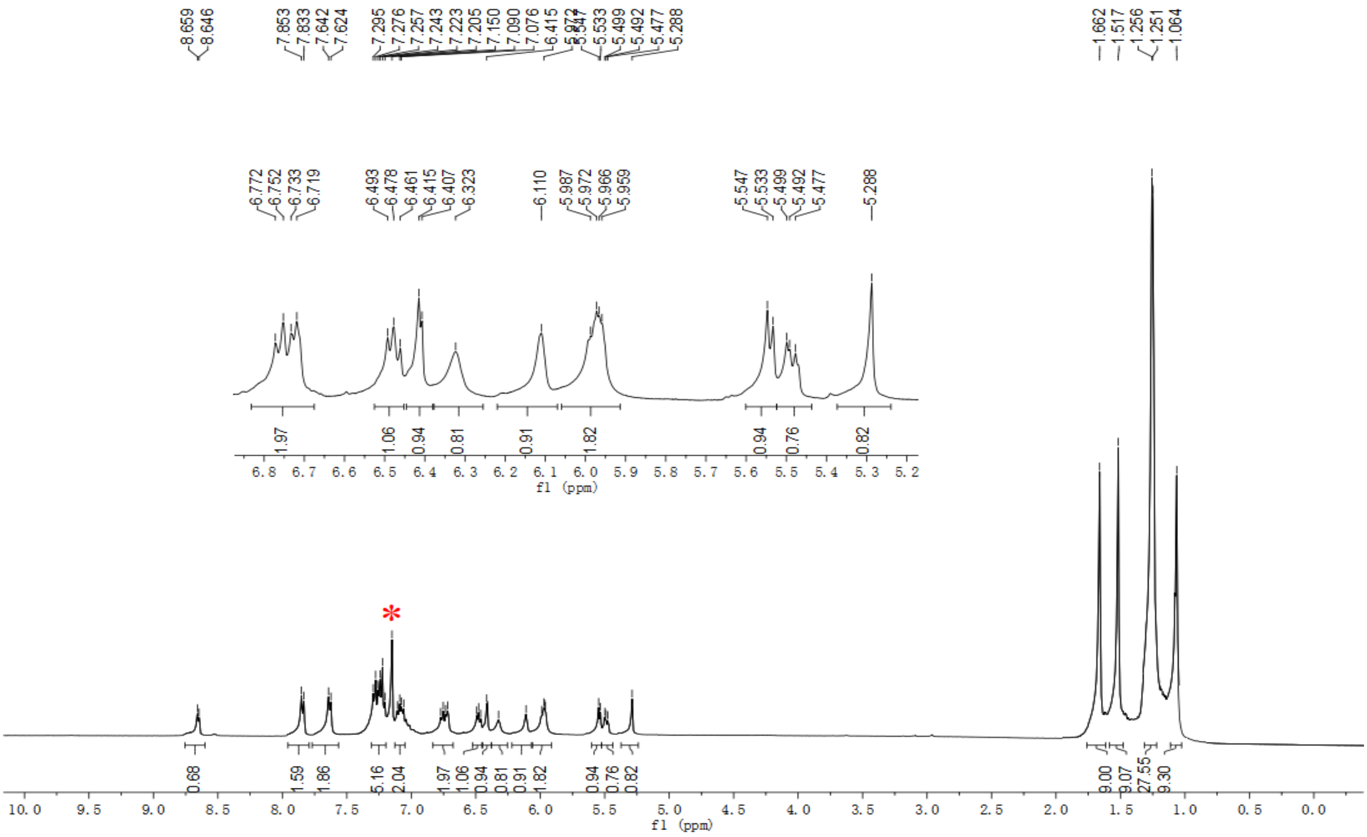

Figure S40. ^{1}H NMR spectrum for compound **17** (* solvent).

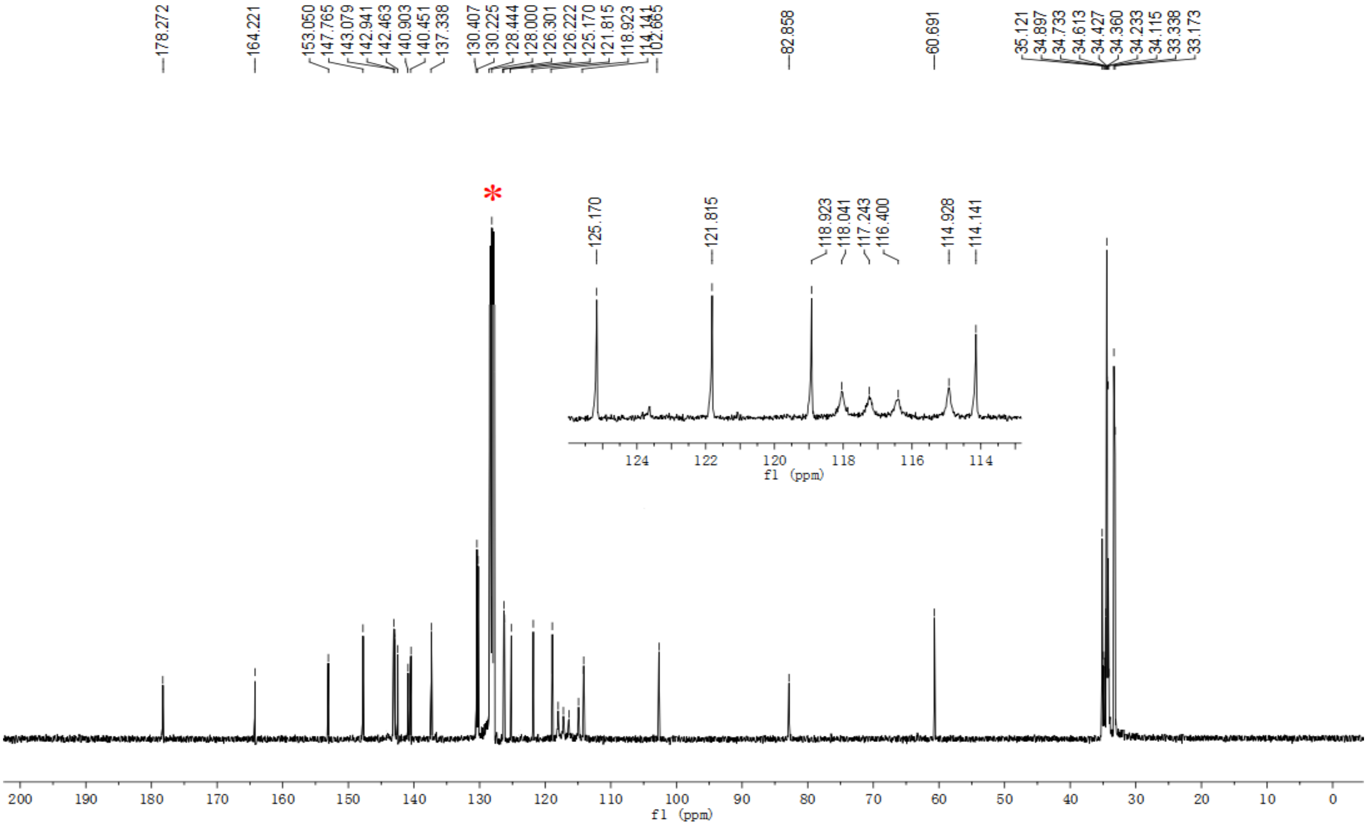

Figure S41. ^{13}C{^{1}H} NMR spectrum for compound **17** (* solvent).

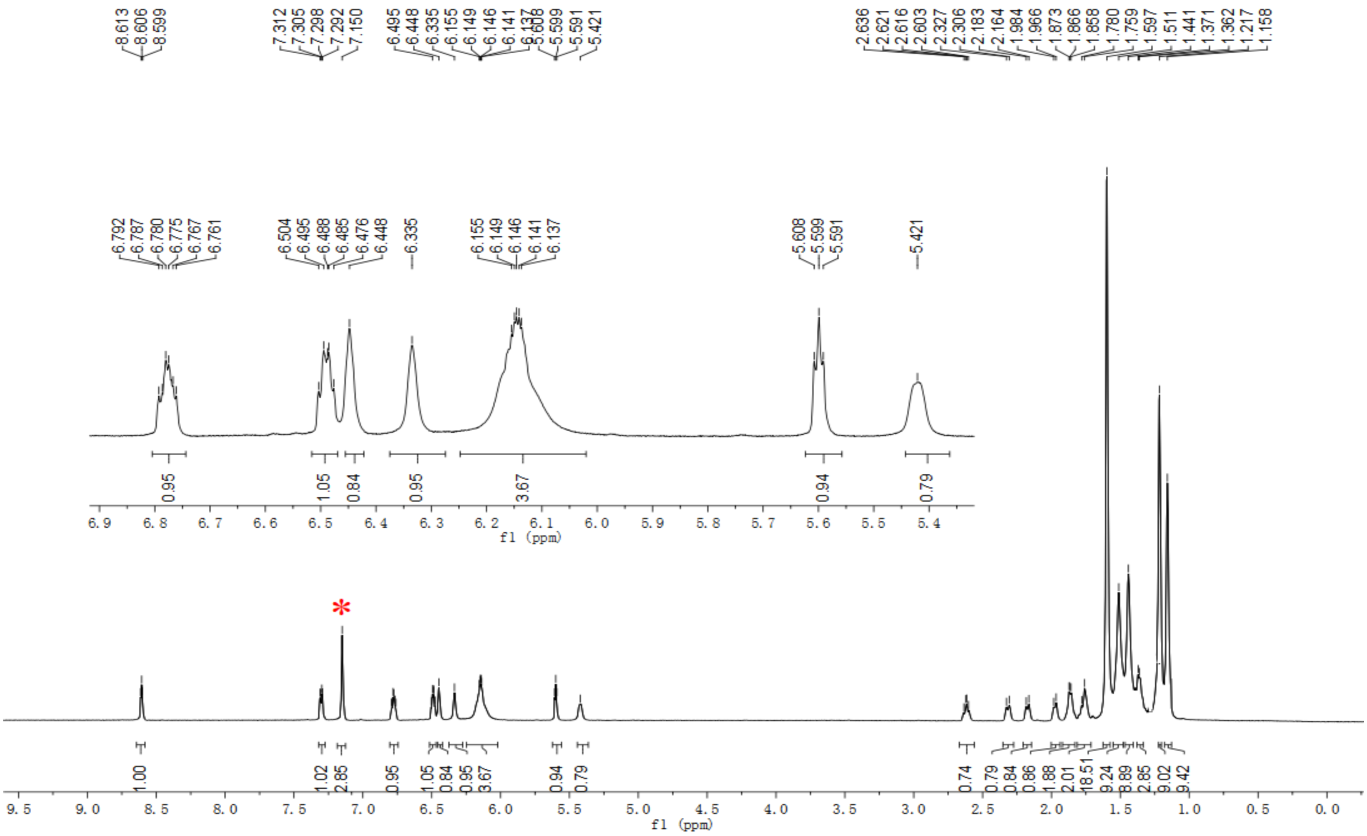

Figure S42. ^{1}H NMR spectrum for compound **18** (* solvent).

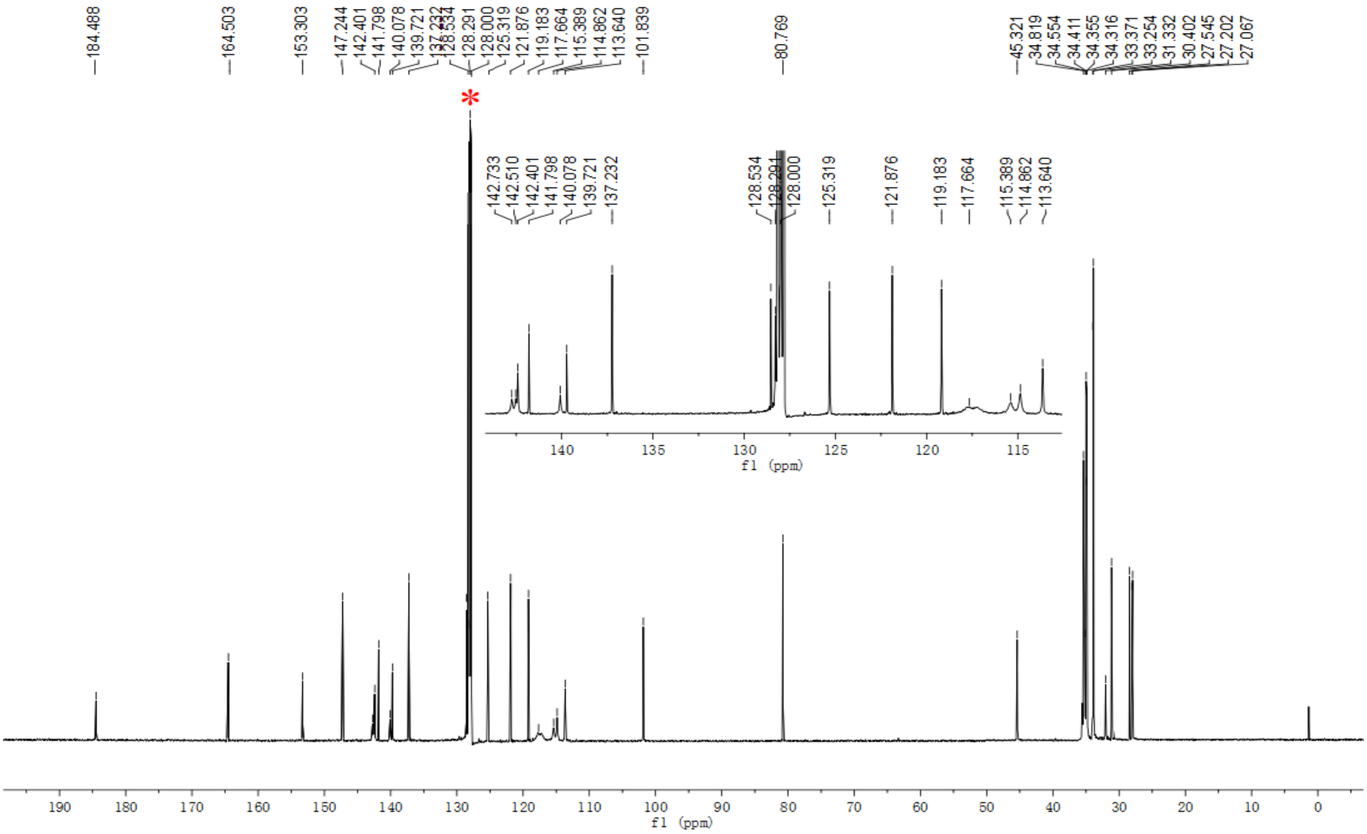

Figure S43. $^{13}C\{^{1}H\}$ NMR spectrum for compound **18** (* solvent).

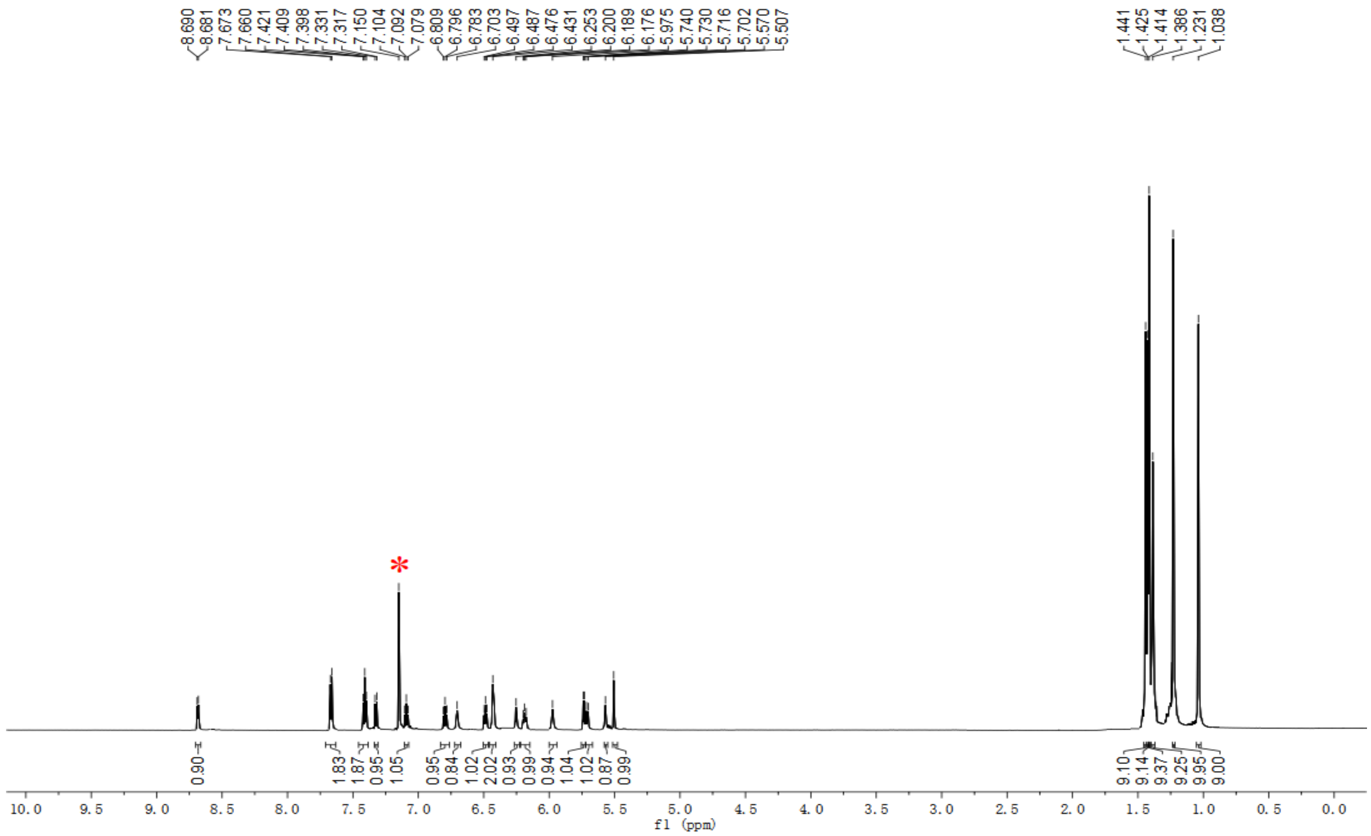

Figure S44. ^{1}H NMR spectrum for compound **19** (* solvent).

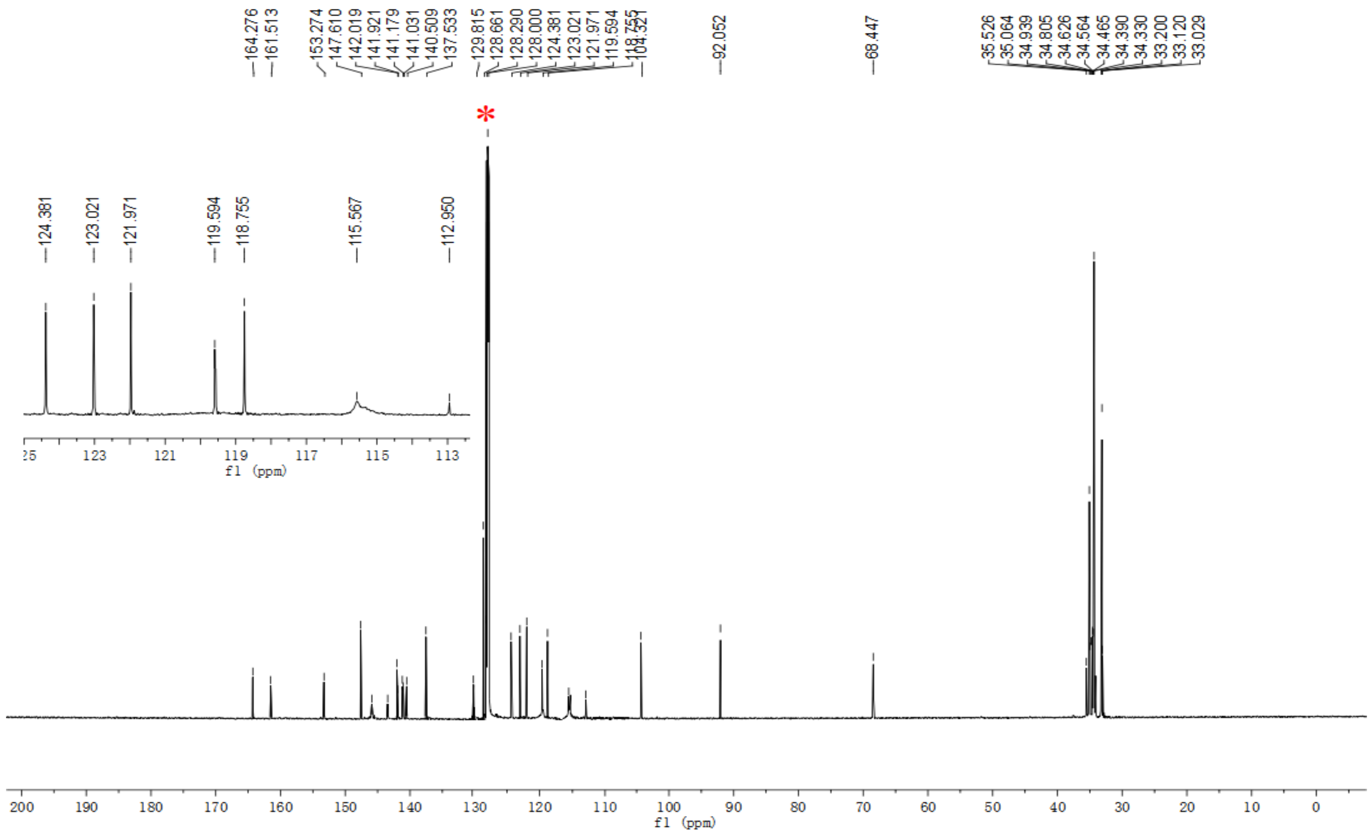

Figure S45. $^{13}C\{^{1}H\}$ NMR spectrum for compound **19** (* solvent).

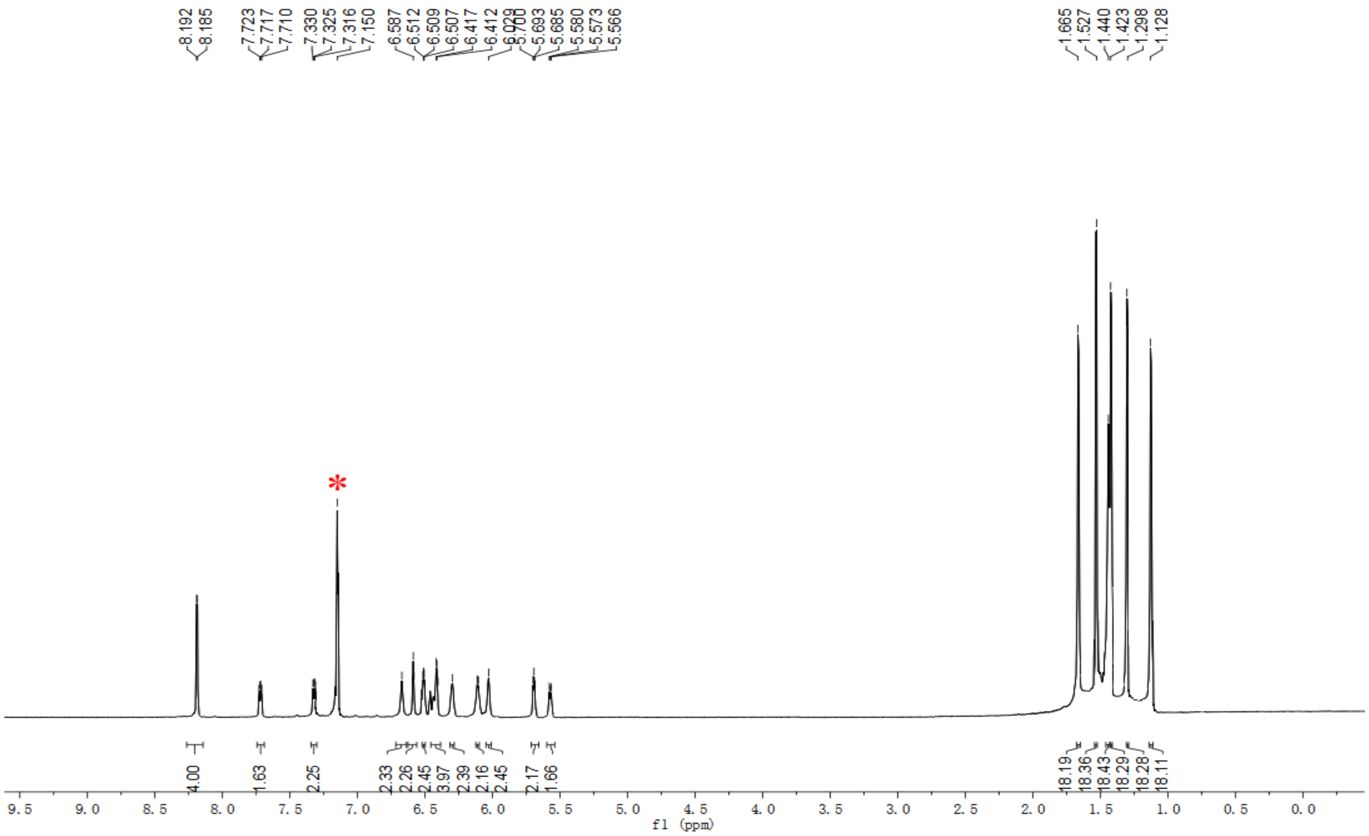

Figure S46. ^{1}H NMR spectrum for compound **20** (* solvent).

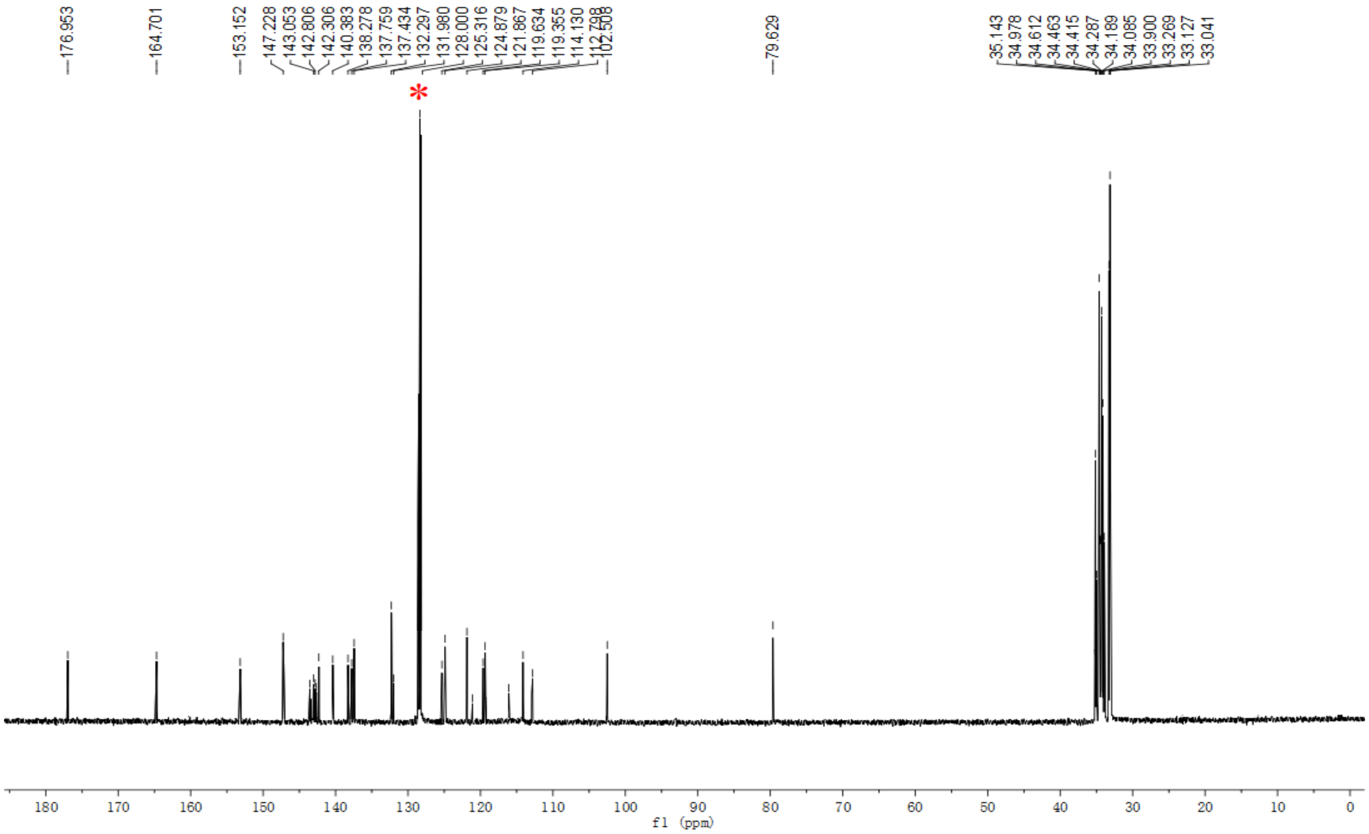

Figure S47. $^{13}C\{^{1}H\}$ NMR spectrum for compound **20** (* solvent).

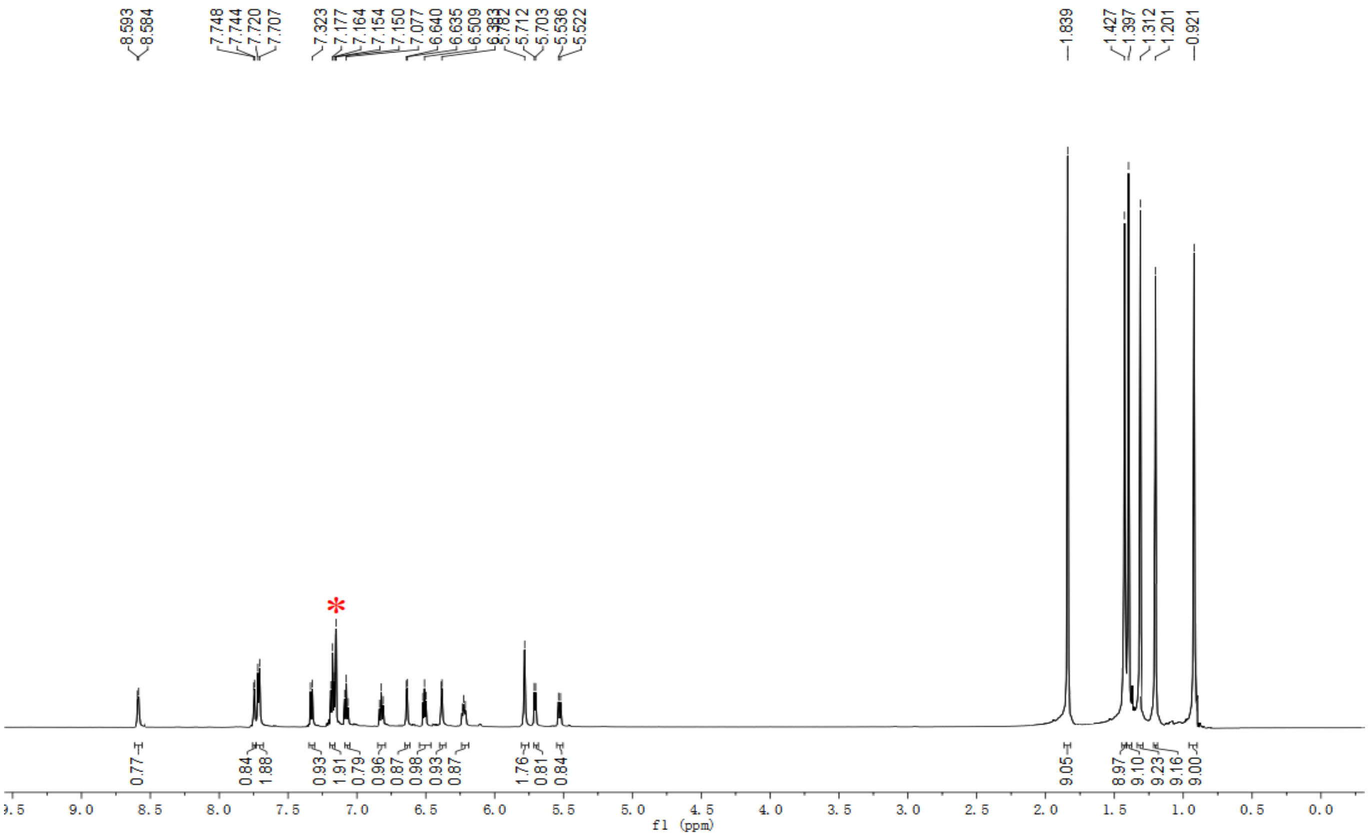

Figure S48. ^{1}H NMR spectrum for compound **21** (* solvent).

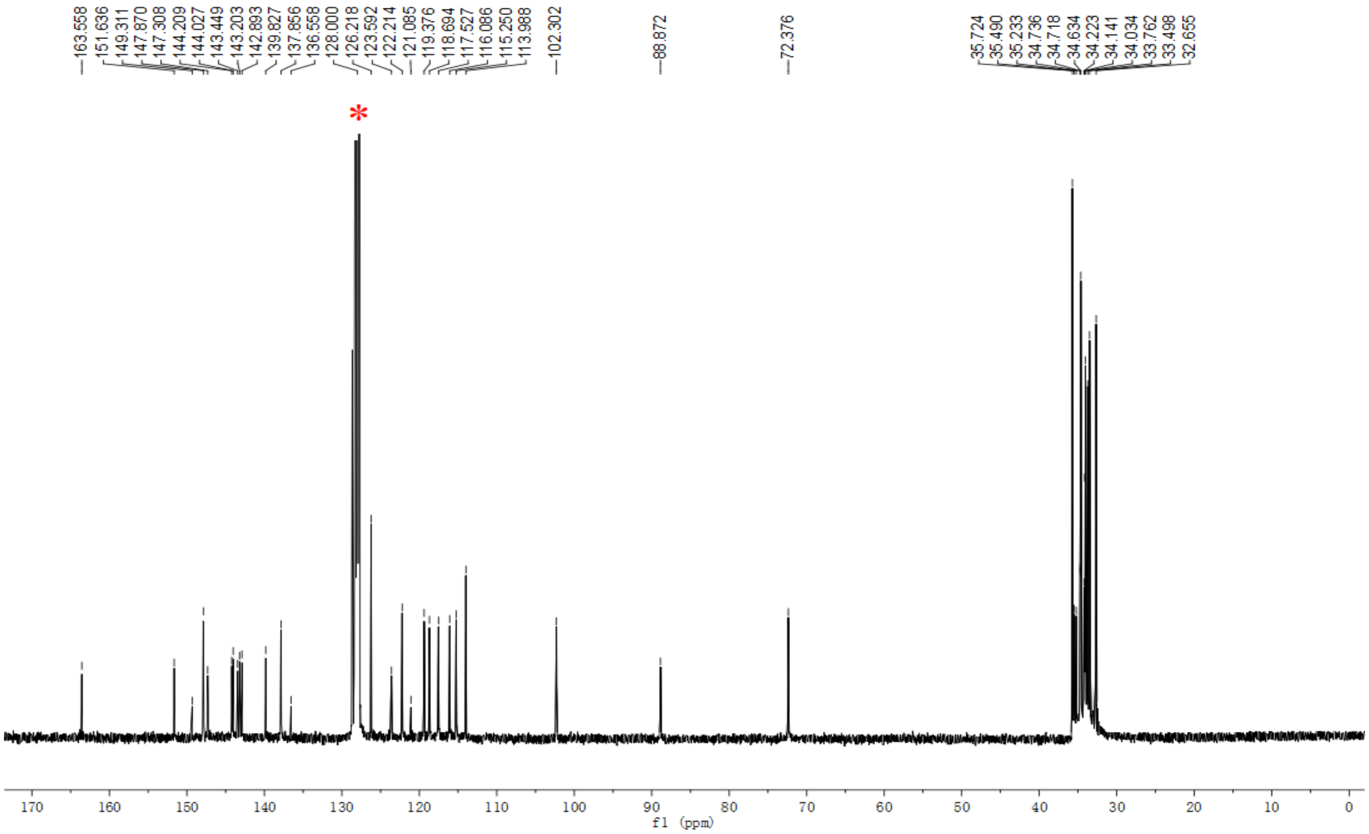

Figure S49. $^{13}C\{^{1}H\}$ NMR spectrum for compound **21** (* solvent).

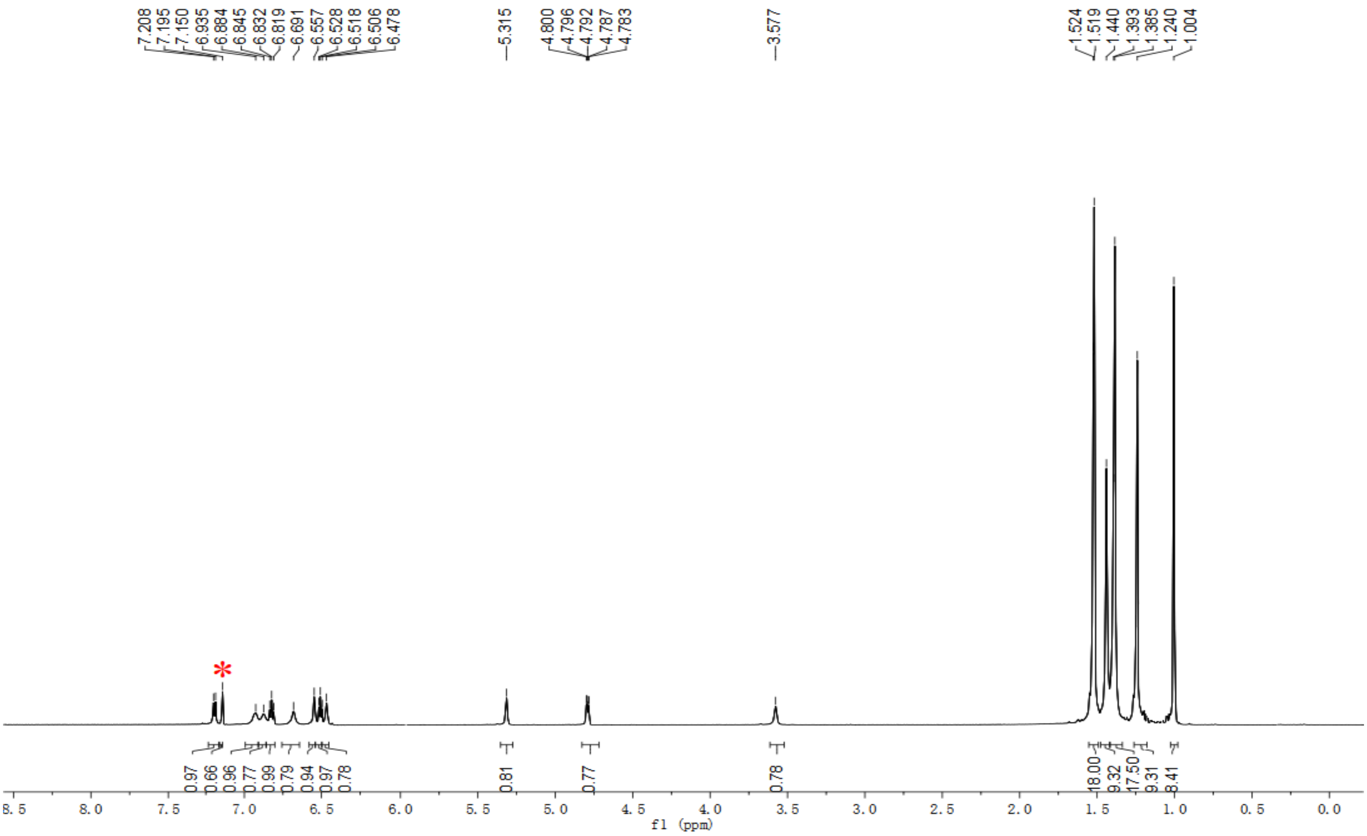

Figure S50. ^{1}H NMR spectrum for compound **22** (* solvent).

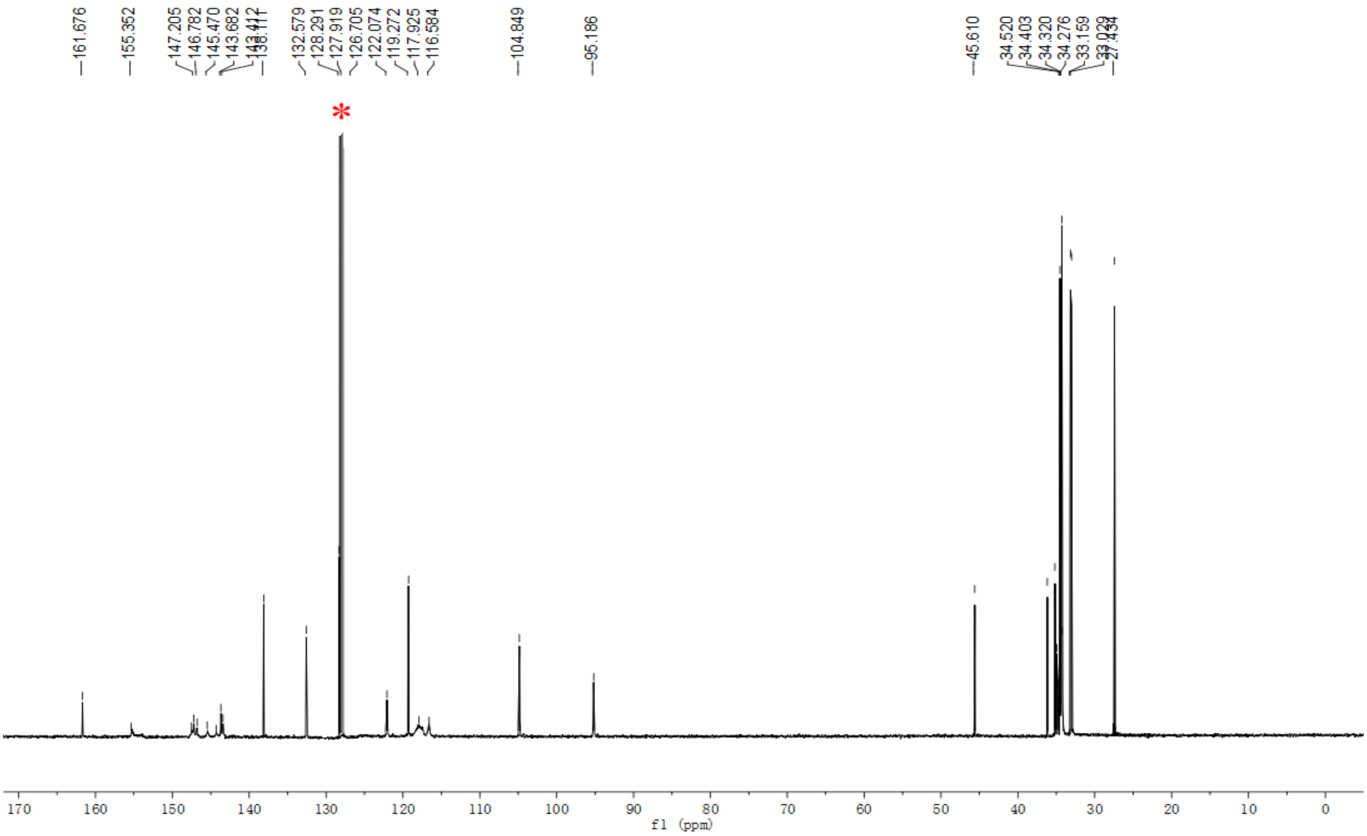

Figure S51. $^{13}C\{^{1}H\}$ NMR spectrum for compound **22** (* solvent).

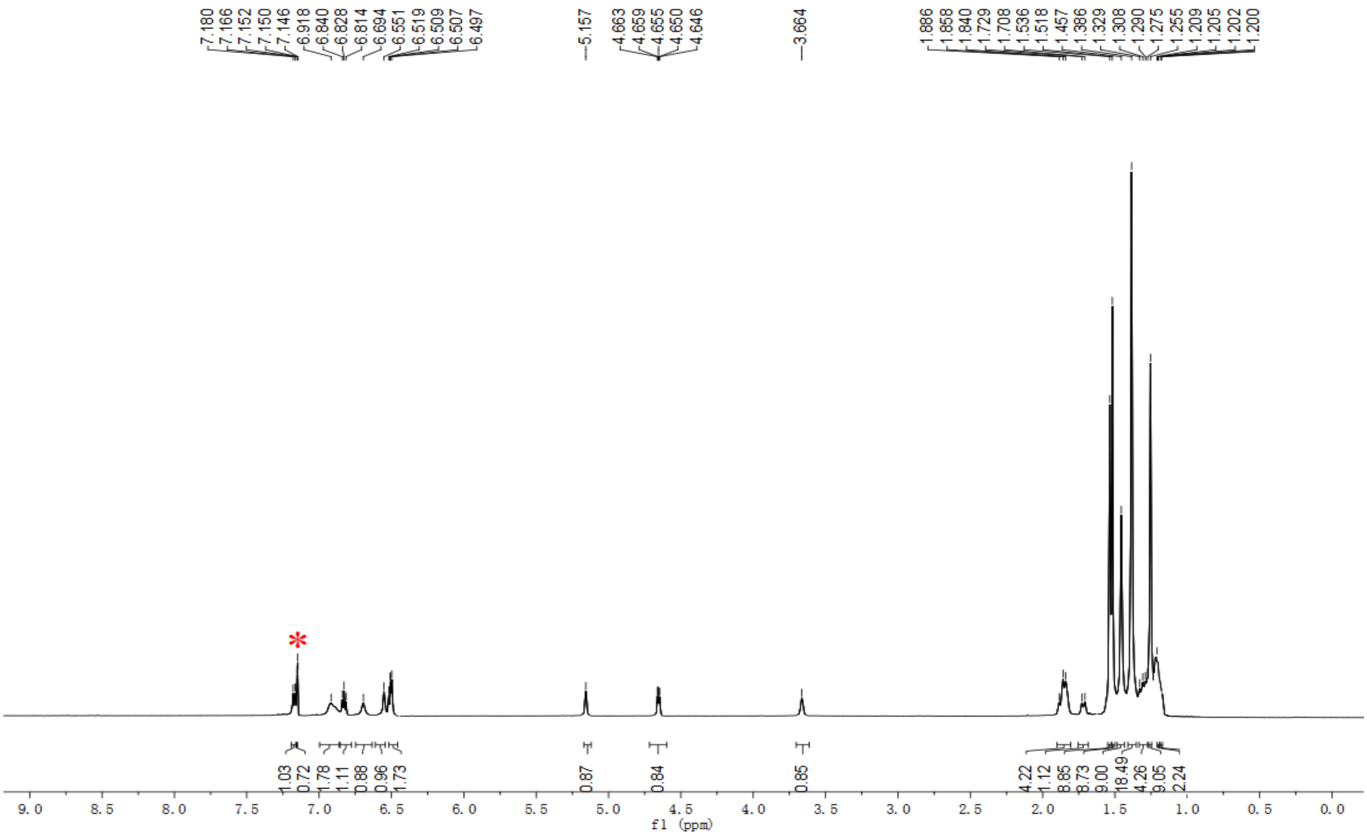

Figure S52. ^{1}H NMR spectrum for compound **23** (* solvent).

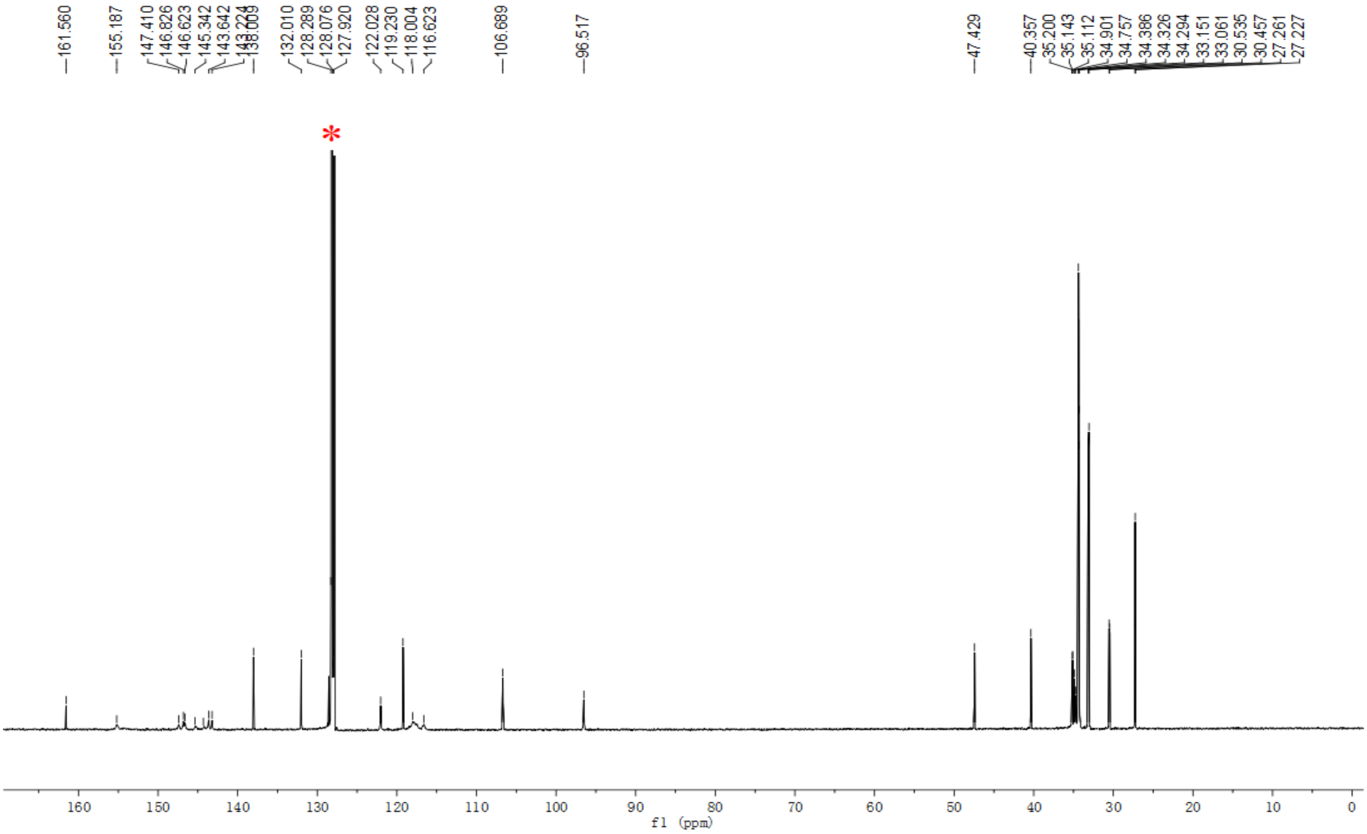

Figure S53. ^{13}C{^{1}H} NMR spectrum for compound **23** (* solvent).

5. References

(1) Frisch, M. J.; Trucks, G. W.; Schlegel, H. B.; Scuseria, G. E.; Robb, M. A.; Cheeseman, J. R.; Scalmani, G.; Barone, V.; Mennucci, B.; Petersson, G. A.; Nakatsuji, H.; Caricato, M.; Li, X.; Hratchian, H. P.; Izmaylov, A. F.; Bloino, J.; Zheng, G.; Sonnenberg, J. L.; Hada, M.; Ehara, M.; Toyota, K.; Fukuda, R.; Hasegawa, J.; Ishida, M.; Nakajima, T.; Honda, Y.; Kitao, O.; Nakai, H.; Vreven, T.; Montgomery, J. A. Jr.; Peralta, J. E.; Ogliaro, F.; Bearpark, M.; Heyd, J. J.; Brothers, E.; Kudin, K. N.; Staroverov, V. N.; Kobayashi, R.; Normand, J.; Raghavachari, K.; Rendell, A.; Burant, J. C.; Iyengar, S. S.; Tomasi, J.; Cossi, M.; Rega, N.; Millam, J. M.; Klene, M.; Knox, J. E.; Cross, J. B.; Bakken, V.; Adamo, C.; Jaramillo, J.; Gomperts, R.; Stratmann, R. E.; Yazyev, O.; Austin, A. J.; Cammi, R.; Pomelli, C.; Ochterski, J. W.; Martin, R. L.; Morokuma, K.; Zakrzewski, V. G.; Voth, G. A.; Salvador, P.; Dannenberg, J. J.; Dapprich, S.; Daniels, A. D.; Farkas, O.; Foresman, J. B.; Ortiz, J. V.; Cioslowski, J.; Fox, D. J. *Gaussian 09*, Revision A.02, Gaussian, Inc.: Wallingford CT, 2009.

(2) Küchle, W.; Dolg, M.; Stoll, H.; Preuss, H. Energy-adjusted pseudopotentials for the actinides. Parameter sets and test calculations for thorium and thorium monoxide. *J. Chem. Phys.* **1994**, *100*, 7535-7542.

(3) Cao, X.; Dolg, M.; Stoll, H. Valence basis sets for relativistic energy-consistent small-core actinide pseudopotentials. *J. Chem. Phys.* **2003**, *118*, 487-496.

(4) Cao, X.; Dolg, M. Segmented contraction scheme for small-core actinide pseudopotential basis sets. *J. Mol. Struct. (THEOCHEM)* **2004**, *673*, 203-209.
